# Hippocampal dentate gyri proteomics reveals Wnt signaling involvement in the behavioral impairment in the THRSP-overexpressing ADHD mouse model

**DOI:** 10.1038/s42003-022-04387-5

**Published:** 2023-01-16

**Authors:** Raly James Perez Custodio, Hee Jin Kim, Jiyeon Kim, Darlene Mae Ortiz, Mikyung Kim, Danilo Buctot, Leandro Val Sayson, Hyun Jun Lee, Bung-Nyun Kim, Eugene C. Yi, Jae Hoon Cheong

**Affiliations:** 1 Department of Ergonomics, Leibniz Research Centre for Working Environment and Human Factors - IfADo, Ardeystr. 67, 44139 Dortmund, Germany; 2 Uimyung Research Institute for Neuroscience, Department of Pharmacy, Sahmyook University, 815 Hwarangro, Nowon-gu Seoul, 01795 Republic of Korea; 3 Institute for New Drug Development, College of Pharmacy, Jeonbuk National University, 567 Baekje-daero, Deokjin-gu, Jeonju-si Jeollabuk-do, 54896 Republic of Korea; 4 Department of Molecular Medicine and Biopharmaceutical Sciences, Graduate School of Convergence Science and Technology and College of Medicine, Seoul National University, Seoul, 03080 Republic of Korea; 5 Department of Chemistry & Life Science, Sahmyook University, 815 Hwarangro, Nowon-gu Seoul, 01795 Republic of Korea; 6 Department of Psychiatry and Behavioral Science, College of Medicine, Seoul National University, 101 Daehakro, Jongno-gu Seoul, 03080 Republic of Korea

**Keywords:** Disease model, ADHD, Hippocampus, ADHD, Epigenetics and behaviour

## Abstract

Children with attention-deficit/hyperactivity disorder (ADHD) often struggle with impaired executive function, temporal processing, and visuospatial memory, hallmarks of the predominantly inattentive presentation (ADHD-PI), subserved by the hippocampus. However, the specific genes/proteins involved and how they shape hippocampal structures to influence ADHD behavior remain poorly understood. As an exploratory tool, hippocampal dentate gyri tissues from thyroid hormone-responsive protein overexpressing (THRSP OE) mice with defining characteristics of ADHD-PI were utilized in proteomics. Integrated proteomics and network analysis revealed an altered protein network involved in Wnt signaling. Compared with THRSP knockout (KO) mice, THRSP OE mice showed impaired attention and memory, accompanied by dysregulated Wnt signaling affecting hippocampal dentate gyrus cell proliferation and expression of markers for neural stem cell (NSC) activity. Also, combined exposure to an enriched environment and treadmill exercise could improve behavioral deficits in THRSP OE mice and Wnt signaling and NSC activity. These findings show new markers specific to the ADHD-PI presentation, converging with the ancient and evolutionary Wnt signaling pathways crucial for cell fate determination, migration, polarity, and neural patterning during neurodevelopment. These findings from THRSP OE mice support the role of Wnt signaling in neurological disorders, particularly ADHD-PI presentation.

## Abstract (web summary)

Proteomics in the hippocampal dentate gyri reveal that behavioural impairments in THRSP-overexpressing mice could be regulated by Wnt signalling.

## Introduction

Attention-deficit/hyperactivity disorder (ADHD) is a heterogeneous neurodevelopmental condition characterized by a ubiquitous array of inattention, impulsivity, and hyperactivity behaviors typically diagnosed in children^1^ and often persist into adulthood^2^. According to global estimates, ADHD affects 8–12% of children^3^ and 2–6% of adults^4^. Moreover, twin and adoption studies have indicated that ADHD is a highly heritable disorder, with heritability estimated between 77% and 90%^5^, suggesting the substantial role of genetics in the etiology of ADHD. However, no single gene can predict this disorder. Conversely, numerous studies have indicated that ~33% of ADHD heritability may be caused by polygenic factors comprising several common variants, each with a small effect size^6^. Furthermore, considerable evidence supports the interaction between genetic (polygenic) and environmental factors during early development^7^.

Studies have implicated widespread changes in the brain macro- and microstructure, including the frontal, basal ganglia, anterior cingulate, temporal, and parietal regions^8^, with high associations in the left parahippocampal gyrus^9^ and hippocampus (HPC)^10^. Children diagnosed with ADHD frequently struggle with deficits in executive function, temporal processing, and visuospatial memory, the defining hallmarks of predominantly inattentive presentation (ADHD-PI)^11^, considered to be subserved by the hippocampal region.

The HPC comprises two layers of neurons, pyramidal neurons in the cornu ammonis (CA) and granule neurons in the dentate gyrus (DG) fields^12^. This brain region is involved in consolidating emotional memory and episodic memory formation. Interestingly, the hippocampal DG can generate new neurons throughout life in humans^13^ and rodents^14^, termed adult neurogenesis^15^ and believed to play a crucial role in cognitive plasticity^16^. Interestingly, alterations during neurogenesis and plasticity of the developing brain and later in life are often discussed as vulnerability factors for developing psychiatric disorders such as ADHD^17^. Recently, personalized fingerprinting of the brain functional connectome has revealed sensitive time points in brain maturation and plasticity that differed from those with symptoms of ADHD^18^. Evidence indicates that candidate risk genes in ADHD are enriched in pathways such as neurite outgrowth, axon guidance, cell trafficking, brain development, and neurogenesis^19^, all of which are regulated by Wnt signaling^20,21^.

Extracellular Wnt signaling stimulates numerous intracellular signaling cascades that regulate crucial aspects of neural stem cell (NSC) fate determination, cell migration, cell polarity, neural patterning, and organogenesis^22^ during embryonic and adult brain development^23^ via the transcriptional coactivator catenin beta-1 (β-catenin; *Ctnnb1*), which forms the Wnt/β-catenin signaling pathway. Given this extensive range of roles, dysregulation of Wnt signaling could have pathological effects on neurodevelopment^24^. Previous findings have afforded new hypotheses: Apart from the well-recognized multifactorial ADHD etiological factors, recent evidence proposes that the interface between genetic and environmental factors and particularly Wnt signaling pathways might significantly contribute to ADHD pathophysiology^25^.

Brain processes such as executive functions (i.e., response inhibition, working memory) are involved in monitoring and regulating behavior often compromised in ADHD. The development of executive function skills occurs from infancy into young adulthood, and there has been increased interest in interventions designed to target these skills. Physical activity and environmental enrichment have been linked to neurocognitive development and healthy brain functioning, leading some researchers to consider their potential benefits for fostering executive function skill development, specifically for individuals with ADHD^26^.

In an extension of our previous studies and for preclinical exploration of the genetic underpinnings of ADHD, this study performed proteomic analysis of the Hippocampal DG of a thyroid hormone-responsive protein (THRSP)-overexpressing (OE) mouse model of predominantly inattentive ADHD (ADHD-PI) presentation. This “bottom-up” experimental approach is intended to screen and identify enriched pathways, particularly those involved in Wnt signaling, which could further elucidate the ADHD-PI endophenotype observed in THRSP OE mice. We also evaluated the effects of environmental enrichment on behavior and gene expression.

## Results

### Hippocampal DG proteomic analysis in an ADHD-PI mouse model

The inheritance of ADHD is deemed a complex phenomenon involving polygenic factors, accompanied by the development of distinct behavioral characteristics. Studies have shown that targeting a specific gene through genetic manipulation, as observed in transgenic models, intrinsically alters alternate genes by either upregulating or downregulating their expression levels, potentially inducing symptoms such as inattention, hyperactivity, and impulsivity. Previously, we have reported that THRSP overexpression in mice can produce attention and memory impairment related to dopaminergic^27^ and thyroid hormones^28^ aberrations, although other signaling mechanisms may also play a potential role. We conducted a proteomics analysis to provide a comprehensive representation of structural and functional data of the hippocampal DG, which plays a predominant role in neurogenesis and cognitive processing, as well as the response mechanisms concerning genetic manipulations of THRSP in THRSP OE and KO mice.

**Fig. 1:**
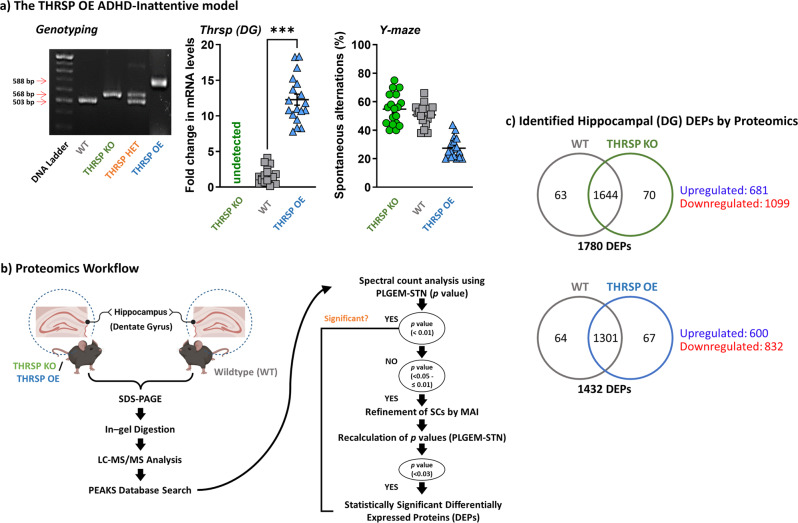
Proteomics workflow in THRSP transgenic mice. **a** Confirmation of transgenic backgrounds in mice was performed via DNA electrophoresis (Genotyping) and qRT-PCR (Thrsp mRNA expression) analyses (*n* = 18 mice/group, two-tailed, paired *t* test: (*t* = 11.9, df = 17, *P* < 0.001)). Thrsp mRNA was not detected in THRSP KO mice. Subsequently, mice were exposed to the Y-maze test to reconfirm inattention in THRSP OE mice when compared to WT and THRSP KO mice (*n* = 18 mice/group: one-way ANOVA, *F* (_2, 51_) = 51.7, *P* < 0.001). THRSP OE mice show sustained inattention and memory impairments. Values are presented as the mean ± standard error of the mean (SEM). **b** Following the confirmation of behavior in mice, hippocampal DG were prepared for subsequent proteomics analysis. **c** Summary of identified DEPs in mouse hippocampal DG. THRSP/Thrsp thyroid hormone-responsive protein, OE overexpressing, DG dentate gyrus, DEPs differentially expressed proteins. The images used in (**b**) was created with BioRender.com.

**Fig. 2:**
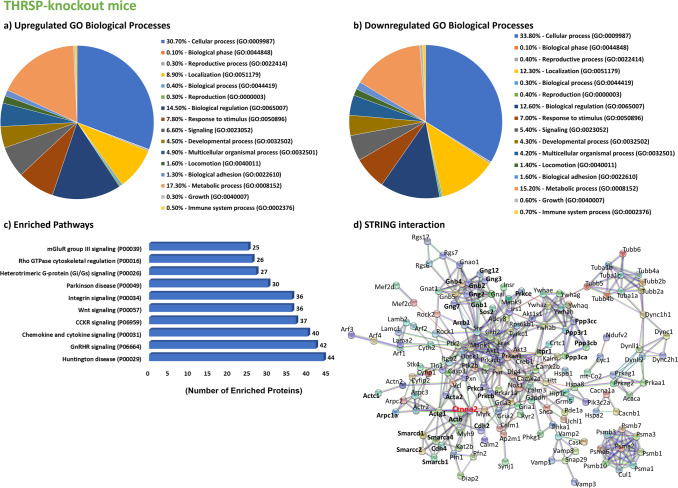
Proteomics analysis of the hippocampal DG of THRSP KO mice. **a**, **b** The Gene Ontology biological processes of upregulated and downregulated proteins in the hippocampal DG of adult THRSP KO mice (*n* = 6/group). In total, 681 and 1099 proteins are upregulated and downregulated by 0.8-fold, respectively. **c** Reactome pathway enrichment analysis using upregulated and downregulated proteins identified from the proteomics screen. **d** STRING protein–protein interaction network analysis indicates that Ctnna2 (highlighted in red), the only gene directly related to Wnt signaling, is highly interacting. Although, other Wnt signaling-related proteins were identified to exhibit an interaction. THRSP thyroid hormone-responsive protein, OE overexpressing, DG dentate gyrus, STRING Search Tool for the Retrieval of Interacting Genes/Proteins, Ctnna2 Catenin alpha-2.

**Fig. 3:**
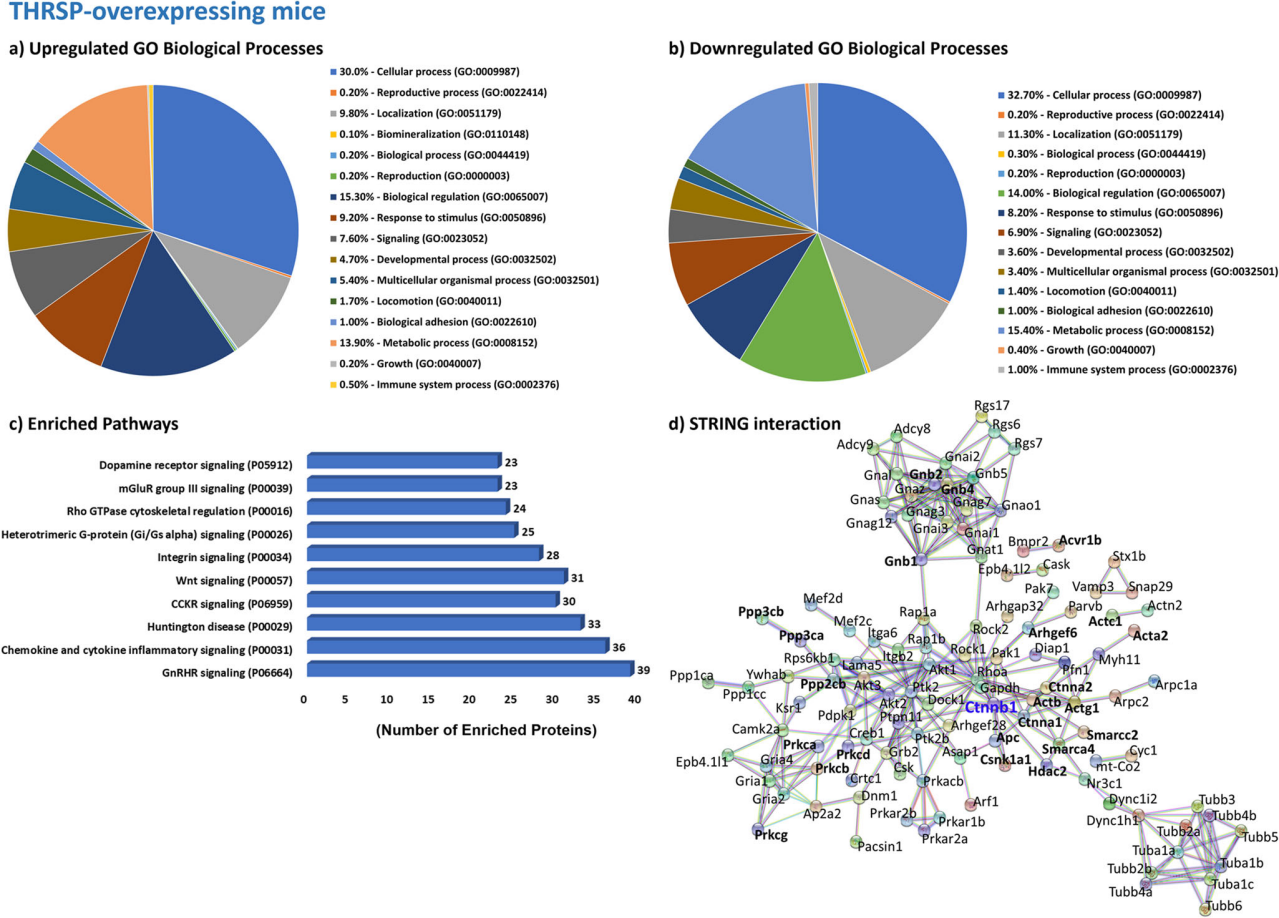
Proteomics analysis of the hippocampal DG of THRSP OE mice. **a**, **b** The Gene Ontology biological processes of upregulated and downregulated proteins in the hippocampal DG of adult THRSP OE mice (*n* = 6/group). In total, 600 proteins are upregulated by at least 0.8-fold, whereas 832 proteins are downregulated by 0.5-fold. **c** Reactome pathway enrichment analysis using upregulated and downregulated proteins identified from the proteomics screen. **d** STRING protein–protein interaction network analysis indicates enriched interactions indicate Ctnnb1 (highlighted in blue) as the most highly interacting protein, although other Wnt signaling-related proteins were identified. THRSP thyroid hormone-responsive protein, OE overexpressing, DG dentate gyrus, STRING Search Tool for the Retrieval of Interacting Genes/Proteins, Ctnnb1 Catenin beta-1.

The results revealed a total of 1780 differentially expressed proteins (DEPs) (Fig. 1c and Supplementary Tables 1 and 2) between THRSP KO and WT mice, with 681 and 1099 proteins upregulated and downregulated by 0.8-fold, respectively. Moreover, 1432 DEPs were observed between THRSP OE and WT (Fig. 1c and Supplementary Tables 3 and 4), where 600 proteins were upregulated by at least 0.8-fold and 832 were downregulated by 0.5-fold. Using this information, we applied the GENEONTOLOGY/PANTHER classification system to identify the GO biological processes involved in upregulated and downregulated proteins, and the total DEPs were analyzed to determine enriched pathways. GO analyses of DEPs in THRSP KO and OE mice identified variations in cellular processes, including localization, adhesion, and response to stimuli, as well as in metabolic and developmental processes (Figs. 2a, b and 3a, b). These biological processes may indicate the participation of NSC activity, which could be associated with hippocampal-dependent behaviors of these mice in the Y-maze test (Fig. 1a). Our previous and present data demonstrate that THRSP KO mice exhibit a normal to high percentage of spontaneous alternations^28^, whereas THRSP OE mice (the ADHD-PI model) exhibited a low percentage of spontaneous alternations^27,28^, indicating a significant difference in their behavior probably attributed to functional deletion and overexpression of THRSP, respectively. However, whether NSCs are activated in response to their transgenic nature needs to be examined. Therefore, we conducted GO enrichment analysis and identified an enriched Wnt signaling pathway (Figs. 2c and 3c), where 36 genes from THRSP KO mice (Table 1) and 31 genes from THRSP OE mice (Table 2) demonstrated direct involvement in this signaling pathway. The data demonstrated the involvement of NSC activity in these THRSP transgenic mice, revealing that alterations in THRSP gene expression induce an inherent change in the expression of hippocampal DG Wnt-related proteins. Furthermore, we identified the enrichment of a pathway related to Rho-GTPase cytoskeletal regulation (Figs. 2c and 3c), previously identified as a key mediator of Wnt signaling^29^. Interestingly, THRSP OE mice presented variations in genes enriched in dopamine receptor signaling, supporting our previous findings that dopaminergic signaling is a factor underlying inattentive behavior^27^.

**Table 1:** DEPs in THRSP KO mice involved in Wnt signaling.

Expression	Mapped IDs	Name/Symbo006C	Fold change	*P* value
Upregulated	Gnb4	Guanine nucleotide-binding protein subunit beta-4; GNB4; ortholog	1.7868	0.0011
	Arrb1	Beta-arrestin-1; ARRB1; ortholog	1.3168	0.0040
	Gnb1	Guanine nucleotide-binding protein G(I)/G(S)/G(T) subunit beta-1; GNB1; ortholog	1.3159	0.0041
	Tbl1xr1	F-box-like/WD repeat-containing protein TBL1XR1; TBL1XR1; ortholog	1.2978	0.0055
	Gnb2	Guanine nucleotide-binding protein G(I)/G(S)/G(T) subunit beta-2; GNB2; ortholog	1.2958	0.0055
	Cdh2	Cadherin-2; CDH2; ortholog	1.2180	0.0069
	Adss1	Adenylosuccinate synthetase isozyme 1; ADSS1; ortholog	1.1574	0.0094
	Smarcc2	SWI/SNF complex subunit SMARCC2; SMARCC2; ortholog	1.1382	0.0100
	Csnk2a2	Casein kinase II subunit alpha’; CSNK2A2; ortholog	1.0677	0.0139
	Smarcd1	SWI/SNF-related matrix-associated actin-dependent regulator of chromatin subfamily D member 1; SMARCD1; ortholog	1.0421	0.0154
	Myh7b	Myosin-7B; MYH7B; ortholog	0.9661	0.0216
	Ctbp1	C-terminal-binding protein 1; CTBP1; ortholog	0.9419	0.0238
	Cdh4	Cadherin-4; CDH4; ortholog	0.8791	0.0287
	Smarcb1	SWI/SNF-related matrix-associated actin-dependent regulator of chromatin subfamily B member 1; SMARCB1; ortholog	0.8791	0.0287
Downregulated	Adss2	Adenylosuccinate synthetase isozyme 2; ADSS2; ortholog	–0.9112	0.0249
	Ppp3r1	Calcineurin subunit B type 1; PPP3R1; ortholog	–0.9115	0.0248
	Ctnna2	Catenin alpha-2; CTNNA2; ortholog	–0.9199	0.0244
	Prkcb	Protein kinase C beta type; PRKCB; ortholog	–1.0276	0.0153
	Smarca4	Transcription activator BRG1; SMARCA4; ortholog	–1.0439	0.0147
	Itpr1	Inositol 1,4,5-trisphosphate receptor type 1; ITPR1; ortholog	–1.0551	0.0139
	Pcdh19	Protocadherin-19;PCDH19;ortholog	–1.1412	0.0093
	Gng2	Guanine nucleotide-binding protein G(I)/G(S)/G(O) subunit gamma-2; GNG2; ortholog	–1.2217	0.0063
	Csnk1g1	Casein kinase I isoform gamma-1; CSNK1G1; ortholog	–1.2269	0.0062
	Ppp3cc	Serine/threonine-protein phosphatase 2B catalytic subunit gamma isoform; PPP3CC; ortholog	–2.0380	0.0005
	Prkce	Protein kinase C epsilon type; PRKCE; ortholog	–2.2312	0.0003
	Prkca	Protein kinase C alpha type; PRKCA; ortholog	–2.3693	0.0002
	Gng12	Guanine nucleotide-binding protein G(I)/G(S)/G(O) subunit gamma-12; GNG12; ortholog	–2.6507	0.0001
	Acta2	Actin, aortic smooth muscle; ACTA2; ortholog	–2.8117	0.0001
	Gng3	Guanine nucleotide-binding protein G(I)/G(S)/G(O) subunit gamma-3; GNG3; ortholog	–2.8255	0.0001
	Ppp3ca	Serine/threonine-protein phosphatase 2B catalytic subunit alpha isoform; PPP3CA; ortholog	–2.8295	0.0001
	Ppp3cb	Serine/threonine-protein phosphatase 2B catalytic subunit beta isoform; PPP3CB; ortholog	–3.6034	0.0000
	Gng7	Guanine nucleotide-binding protein G(I)/G(S)/G(O) subunit gamma-7; GNG7; ortholog	–3.6163	0.0000
	Actc1	Actin, alpha cardiac muscle 1; ACTC1; ortholog	–5.3626	0.0000
	Actg1	Actin, cytoplasmic 2; ACTG1; ortholog	–5.5769	0.0000
	Actb	Actin, cytoplasmic 1; ACTB; ortholog	–5.7398	0.0000

**Table 2:** DEPs in THRSP OE mice involved in Wnt signaling.

Expression	Mapped IDs	Name/symbol	Fold change	*P* value
Upregulated	Ctnna1	Catenin alpha-1; CTNNA1; ortholog	1.6630	0.0016
	Smarcc2	SWI/SNF complex subunit SMARCC2; SMARCC2; ortholog	1.6549	0.0016
	Acvr1b	Activin receptor type-1B; ACVR1B; ortholog	1.5138	0.0021
	Ctnna2	Catenin alpha-2; CTNNA2; ortholog	1.4648	0.0024
	Prkcd	Protein kinase C delta type; PRKCD; ortholog	1.2855	0.0045
	Ctnnb1	Catenin beta-1; CTNNB1; ortholog	1.2173	0.0057
	Hdac2	Histone deacetylase 2; HDAC2; ortholog	1.1406	0.0085
	Apc	Adenomatous polyposis coli protein; APC; ortholog	0.9016	0.0239
	Myh7b	Myosin-7B; MYH7B; ortholog	0.8628	0.0287
Downregulated	Acta2	Actin, aortic smooth muscle; ACTA2; ortholog	–0.5124	0.0125
	Arhgef6	Rho guanine nucleotide exchange factor 6; ARHGEF6; ortholog	–0.8640	0.0281
	Gng3	Guanine nucleotide-binding protein G(I)/G(S)/G(O) subunit gamma-3; GNG3; ortholog	–1.0286	0.0137
	Ppp2cb	Serine/threonine-protein phosphatase 2A catalytic subunit beta isoform; PPP2CB; ortholog	–1.0362	0.0135
	Csnk1g1	Casein kinase I isoform gamma-1; CSNK1G1; ortholog	–1.0397	0.0133
	Gng7	Guanine nucleotide-binding protein G(I)/G(S)/G(O) subunit gamma-7; GNG7; ortholog	–1.0665	0.0122
	Smarca4	Transcription activator BRG1; SMARCA4; ortholog	–1.1084	0.0094
	Prkcb	Protein kinase C beta type; PRKCB; ortholog	–1.1734	0.0076
	Gng12	Guanine nucleotide-binding protein G(I)/G(S)/G(O) subunit gamma-12; GNG12; ortholog	–1.1861	0.0071
	Gnb2	Guanine nucleotide-binding protein G(I)/G(S)/G(T) subunit beta-2; GNB2; ortholog	–1.3393	0.0037
	Smarcal1	SWI/SNF-related matrix-associated actin-dependent regulator of chromatin subfamily A-like protein 1; SMARCAL1; ortholog	–1.3419	0.0037
	Gnb1	Guanine nucleotide-binding protein G(I)/G(S)/G(T) subunit beta-1; GNB1; ortholog	–1.4755	0.0024
	Actb	Actin, cytoplasmic 1; ACTB; ortholog	–1.5119	0.0022
	Gnb4	Guanine nucleotide-binding protein subunit beta-4; GNB4; ortholog	–1.5291	0.0021
	Cdh9	Cadherin-9; CDH9; ortholog	–1.5912	0.0018
	Actg1	Actin, cytoplasmic 2; ACTG1; ortholog	–1.6339	0.0017
	Csnk1a1	Casein kinase I isoform alpha; CSNK1A1; ortholog	–1.8498	0.0011
	Ppp3ca	Serine/threonine-protein phosphatase 2B catalytic subunit alpha isoform; PPP3CA; ortholog	–1.9689	0.0009
	Prkca	Protein kinase C alpha type; PRKCA; ortholog	–1.9847	0.0009
	Prkcg	Protein kinase C gamma type; PRKCG; ortholog	–2.1606	0.0007
	Actc1	Actin, alpha cardiac muscle 1; ACTC1; ortholog	–2.4216	0.0004
	Ppp3cb	Serine/threonine-protein phosphatase 2B catalytic subunit beta isoform; PPP3CB; ortholog	–2.6280	0.0003

The total DEPs used to produce GO biological processes were analyzed using STRING, presenting protein–protein interaction networks restricted to high-confidence (0.9) interaction thresholds (Figs. 2d and 3d) only. The protein network of DEPs from THRSP KO mice revealed the high interaction of catenin alpha-2 (CTNNA2) (Fig. 2d, highlighted in red). In contrast, DEPs in THRSP OE mice showed catenin beta-1 (CTNNB1) (Fig. 3d, highlighted in blue) to be among the most highly interacting proteins in the network. These critical findings provide evidence of differences between the two transgenic mice. However, THRSP OE mice are of particular interest, given the differential expression of CTNNB1 (upregulated), the key regulatory protein primarily involved in the formation of canonical Wnt/β-catenin signaling^30^, in this inattentive and memory-impaired transgenic ADHD-PI model. It should be noted that Wnt/β-catenin signaling is speculated to control the balance of NSC proliferation and differentiation during brain development and adult neurogenesis^22,23^. Disruption of Wnt signaling may result in developmental defects and neurological disorders^24^, such as ADHD^31^. These observations showed an association between the upregulated expression of CTNNB1 and the ADHD-PI endophenotype in THRSP OE mice. Other important regulatory proteins were also found to be directly involved in canonical Wnt/β-catenin signaling, including adenomatous polyposis coli (APC) and casein kinase 1 alpha-1 (CSNK1A1), which are innately dysregulated in THRSP OE mice (Table 3). The current focus is on understanding how the upregulation of CTNNB1 affects the regulation of Wnt signaling and its targets and evaluating its contributory effects on hippocampal-dependent behavioral impairment of attention and memory in THRSP OE mice.

**Table 3:** Common upregulated and downregulated proteins in THRSP KO and OE mice involved in Wnt signaling.

Mapped IDs	Gene ID	Protein class	THRSP KO	THRSP OE
Acta2	HUMAN | HGNC = 9393|UniProtKB=P17252	Actin and actin-related protein (PC00039)	–2.8117	–0.5124
Actb	HUMAN | HGNC = 29529|UniProtKB=Q9BZK7	Actin and actin-related protein (PC00039)	–5.7398	–1.5119
Actc1	HUMAN | HGNC = 11105|UniProtKB=Q8TAQ2	Actin and actin-related protein (PC00039)	–5.3626	–2.4216
Actg1	HUMAN | HGNC = 11106|UniProtKB=Q96GM5	Actin and actin-related protein (PC00039)	–5.5769	–1.6339
Acvr1b	HUMAN | HGNC = 9399|UniProtKB=Q05655	Serine/threonine-protein kinase receptor (PC00205)	–	1.5138
Apc	HUMAN | HGNC = 2514|UniProtKB=P35222		–	0.9016
Arhgef6	HUMAN | HGNC = 4853|UniProtKB=Q92769	Guanyl-nucleotide exchange factor (PC00113)	–	–0.8640
Arrb1	HUMAN | HGNC = 132|UniProtKB=P60709	Scaffold/adapter protein (PC00226)	1.3168	–
Cdh2	HUMAN | HGNC = 292|UniProtKB=P30520	Cadherin (PC00057)	1.2180	–
Cdh4	HUMAN | HGNC = 2510|UniProtKB=P26232	Cadherin (PC00057)	0.8791	–
Csnk1a1	HUMAN | HGNC = 15906|UniProtKB=A7E2Y1	Non-receptor serine/threonine-protein kinase (PC00167)	–	–1.8498
Ctnna1	HUMAN | HGNC = 685|UniProtKB=Q15052	Non-motor actin-binding protein (PC00165)	–	1.6630
Ctnna2	HUMAN | HGNC = 4405|UniProtKB=P63215	Non-motor actin-binding protein (PC00165)	–0.9199	1.4648
Ctnnb1	HUMAN | HGNC = 9300|UniProtKB=P62714		–	1.2173
Gnb1	HUMAN | HGNC = 2454|UniProtKB=Q9HCP0	Heterotrimeric G-protein (PC00117)	1.3159	–1.4755
Gnb2	HUMAN | HGNC = 4410|UniProtKB=O60262	Heterotrimeric G-protein (PC00117)	1.2958	–1.3393
Gnb4	HUMAN | HGNC = 11100|UniProtKB=P51532	Heterotrimeric G-protein (PC00117)	1.7868	–1.5291
Gng12	HUMAN | HGNC = 9395|UniProtKB=P05771	Heterotrimeric G-protein (PC00117)	–2.6507	–1.1861
Gng2	HUMAN | HGNC = 15906|UniProtKB=A7E2Y1	Heterotrimeric G-protein (PC00117)	–1.2217	–
Gng3	HUMAN | HGNC = 9395|UniProtKB=P05771	Heterotrimeric G-protein (PC00117)	–2.8255	–1.0286
Gng7	HUMAN | HGNC = 11103|UniProtKB=Q12824	Heterotrimeric G-protein (PC00117)	–3.6163	–1.0665
Hdac2	HUMAN | HGNC = 11102|UniProtKB=Q9NZC9	Histone modifying enzyme (PC00261)	–	1.1406
Itpr1	HUMAN | HGNC = 4410|UniProtKB=O60262	Ligand-gated ion channel (PC00141)	–1.0551	–
Ppp2cb	HUMAN | HGNC = 132|UniProtKB=P60709	Protein phosphatase (PC00195)	–	–1.0362
Ppp3ca	HUMAN | HGNC = 20731|UniProtKB=Q9HAV0	Protein phosphatase (PC00195)	–2.8295	–1.9689
Ppp3cb	HUMAN | HGNC = 1768|UniProtKB=Q9ULB4	Protein phosphatase (PC00195)	–3.6034	–2.6280
Ppp3cc	HUMAN | HGNC = 9314|UniProtKB=Q08209	Protein phosphatase (PC00195)	–2.0380	–
Ppp3r1	HUMAN | HGNC = 20731|UniProtKB=Q9HAV0		–0.9115	–
Prkca	HUMAN | HGNC = 9316|UniProtKB=P48454	Non-receptor serine/threonine-protein kinase (PC00167)	–2.3693	–1.9847
Prkcb	HUMAN | HGNC = 4404|UniProtKB=P59768	Non-receptor serine/threonine-protein kinase (PC00167)	–1.0276	–1.1734
Prkcd	HUMAN | HGNC = 9314|UniProtKB=Q08209	Non-receptor serine/threonine-protein kinase (PC00167)	–	1.2855
Prkce	HUMAN | HGNC = 9315|UniProtKB=P16298	Non-receptor serine/threonine-protein kinase (PC00167)	–2.2312	–
Prkcg	HUMAN | HGNC = 9393|UniProtKB=P17252	Non-receptor serine/threonine-protein kinase (PC00167)	–	–2.1606
Smarca4	HUMAN | HGNC = 4405|UniProtKB=P63215	DNA helicase (PC00011)	–1.0439	–1.1084
Smarcb1	HUMAN | HGNC = 4396|UniProtKB=P62873	DNA metabolism protein (PC00009)	0.8791	–
Smarcc2	HUMAN | HGNC = 1759|UniProtKB=P19022	chromatin/chromatin-binding, or -regulatory protein (PC00077)	1.1382	1.6549
Smarcd1	HUMAN | HGNC = 2454|UniProtKB=Q9HCP0	chromatin/chromatin-binding, or -regulatory protein (PC00077)	1.0421	–

### Aberrant Wnt-related genes in THRSP OE mice

**Fig. 4:**
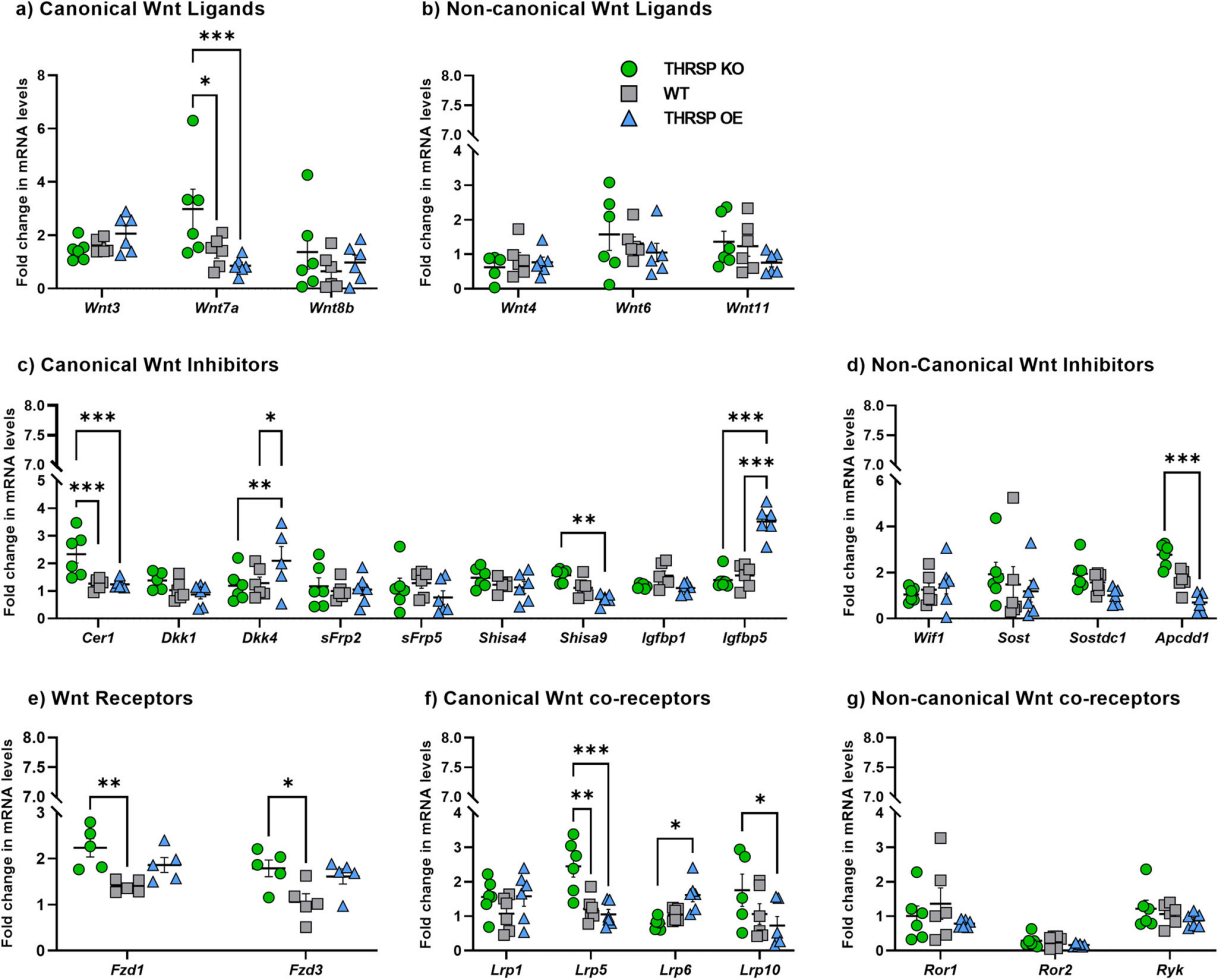
Canonical and noncanonical Wnt pathway elements expressions in the mouse hippocampal DG. qRT-PCR analyses of **a** canonical and **b** noncanonical Wnt ligands (*n* = 6 mice/group; **a** two-way ANOVA, Strain: *F* (_2, 45_) = 3.24, *P* = 0.048; Gene targets: *F* (2, 45) = 3.58, *P* = 0.036; Strain × Gene targets: *F* (_4, 45_) = 3.41, *P* = 0.016; **b** two-way ANOVA, Strain: *F* (_2, 44_) = 1.35, *P* = 0.269; Gene targets: *F* (_2, 44_) = 3.53, *P* = 0.038; Strain × Gene targets: *F* (_4, 44_) = 0.626, *P* = 0.647). The mRNA expression levels of **c** canonical and **d** noncanonical Wnt inhibitors (*n* = 6 mice/group; **c** two-way ANOVA, Strain: *F* (_2, 133_) = 1.70, *P* = 0.186; Gene targets: *F* (_8, 133_)  = 9.61, *P* < 0.001; Strain × Gene targets: *F* (_16, 133_) = 7.31, *P* < 0.001; **d** two-way ANOVA, Strain: *F* (_2, 60_) = 5.16, *P* = 0.009; Gene targets: *F* (_3, 60_) = 0.754, *P* = 0.524; Strain × Gene targets: *F* (_6, 60_) = 1.99, *P* = 0.081). mRNA levels of **e** Wnt receptors, **f** canonical Wnt co-receptors, and **g** noncanonical Wnt co-receptors. **e** Two-way ANOVA, Strain: *F* (_2, 24_) = 11.18, *P* < 0.001; Gene targets: *F* (_1, 24_) = 6.67, *P* = 0.0.16; Strain × Gene targets: *F* (_2, 24_) = 0.182, *P* = 0.835; **f** two-way ANOVA, Strain: *F* (_2, 59_) = 5.33, *P* = 0.007; Gene targets: *F* (_3, 59_) = 2.10, *P* = 0.110; Strain × Gene targets: *F* (_6, 59_) = 4.76, *P* < 0.001; **g** two-way ANOVA, Strain: *F* (_2, 45_) = 1.64, *P* = 0.205; Gene targets: *F* (_2, 45_) =  15.2, *P* < 0.001; Strain × Gene targets: *F* (_4, 45_) = 0.546, *P* = 0.703). Values are presented as the mean ± standard error of the mean (SEM). Data show differential expression of canonical and noncanonical Wnt pathway elements. DG dentate gyrus, qRT-PCR real-time quantitative reverse transcription PCR.

We evaluated Wnt signaling-related markers upstream of CTNNB1, including the gene expression of hippocampal DG Wnt ligands, inhibitors, receptors, and co-receptors. Although CTNNB1 belongs to the canonical Wnt signaling, we also analyzed genes involved in the noncanonical pathway for an in-depth understanding of genetic changes in our THRSP OE mice, during which we also compared expressions of gene targets with that of THRSP KO and WT mice. Among the Wnt ligands measured, a significant reduction in *Wnt7a* gene expression was observed in THRSP OE mice when compared with both THRSP KO and WT mice (Fig. 4a, b). Other genes, including the canonical *Wnt3* and *Wnt8b* ligands and noncanonical *Wnt4*, *Wnt6*, and *Wnt11* ligands, demonstrated insignificant expression changes, although upward/downward trends were observed. Interestingly, the expression of Wnt inhibitors *Cer1*, *Shisa9*, and *Apcdd1* was significantly higher in THRSP KO mice than in WT or THRSP OE mice. However, Wnt inhibitors such as *Dkk4* and *Igfbp5* were upregulated in THRSP OE mice.

Moreover, Wnt receptors *Fzd1* and *Fzd3* were increased in THRSP KO mice, whereas an upward trend was observed in THRSP OE mice when compared with WT mice. Wnt co-receptors of canonical signaling were deregulated and showed increased *Lrp5* and *Lrp10* expression in THRSP KO mice, whereas enhanced *Lrp6* expression was observed in THRSP OE mice. Overall, these findings suggested that Wnt ligands, inhibitors, receptors, and co-receptors were deregulated in THRSP KO and THRSP OE mice without significantly enhancing Wnt ligands. In addition, upregulated *DKk4* and *Igfbp5* Wnt inhibitors present in THRSP OE mice could have affected the imbalance in Wnt receptors and co-receptors. This may also apply to observations from THRSP KO mice, demonstrating that the activation of Wnt ligands triggers altered expression of Wnt inhibitors, receptors, and co-receptors.

### THRSP overexpression enhances some genes involved in (Wnt) multiprotein complex

**Fig. 5:**
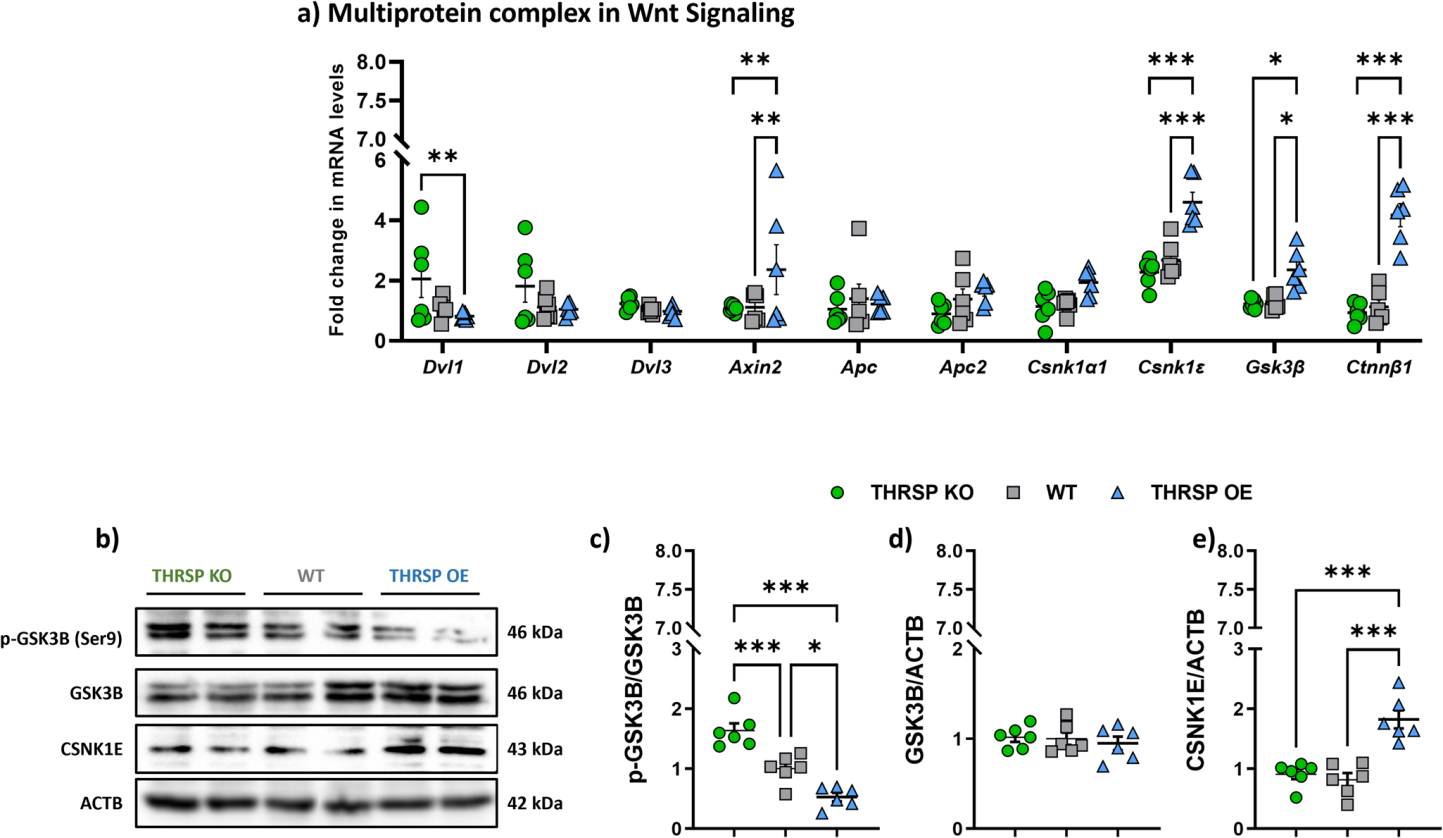
Differential expressions of Wnt signaling multiprotein complex in the mouse hippocampal DG. **a** qRT-PCR analyses of multiprotein complexes. **b** Representative western blots, and corresponding protein levels of **c** p-GSK3B, **d** GSK3B, and **e** CSNK1E, the primary mediators of β-catenin phosphorylation. (*n* = 6 mice/group; **a** two-way ANOVA, Strain: *F* (_2, 150_) = 22.7, *P* < 0.001; Gene targets: *F* (_9, 150_) = 13.7, *P* < 0.001; Strain × Gene targets: *F* (_18, 150_) = 6.47, *P* < 0.001). **c** One-way ANOVA, *F* (_2, 15_) = 32.3, *P* < 0.001; **d** one-way ANOVA, *F* (_2, 15_) = 0.294, *P* = 0.750; **e** one-way ANOVA, *F* (_2, 15_) = 22.5, *P* < 0.001). Values are presented as the mean ±  standard error of the mean (SEM). Data indicate low expression of phosphorylated GSK3B (Ser9) in THRSP OE mice, confirming basal hyperactivity of GSK3B that could subsequently affect β-catenin. OE overexpressing, THRSP thyroid hormone-responsive protein.

The multiprotein complex in Wnt signaling regulates glycogen synthase kinase 3 (GSK3) activity by physically displacing complexed GSK3 from its original regulatory binding partners, APC and casein kinase 1 (CSNK1), and more recently, axin (AXIN), in the so-called destruction complex, consequently triggering the phosphorylation or degradation of β-catenin^32^. β-catenin is phosphorylated by the serine/threonine kinases CSNK1 and GSK3B and targeted for ubiquitination by the β-transducin repeat-containing protein (βTrCP) proteasomal degradation^33^. Following confirmation, in the absence of significant upregulation of Wnt ligands examined in THRSP OE mice, we subsequently assessed the multiprotein complex or the so-called destruction complex that further initiates either augmentation or degradation of β-catenin, deemed necessary for Wnt/β-catenin signaling. Herein, we observed that *Axin2*, *Csnk1ɛ*, and *Gsk3β* were all enhanced in addition to the increased *Ctnnβ1* and decreased *Dvl1* gene expression levels (Fig. 5a). Moreover, the expression of hippocampal DG GSK3B and CSNK1E proteins was enhanced, confirming their enhanced gene expression. Additionally, GSK3B is considered to be constitutively inactivated by phosphorylation at Ser9 and activated by autophosphorylation at Tyr216^34^. This finding indicates that the low phosphorylated GSK3B (Ser9) expression in THRSP OE mice confirms its high activity in the basal form, thereby impacting β-catenin expression.

### THRSP OE mice exhibit reduced BrdU, GFAP, and NEU-N immunoreactivity

**Fig. 6:**
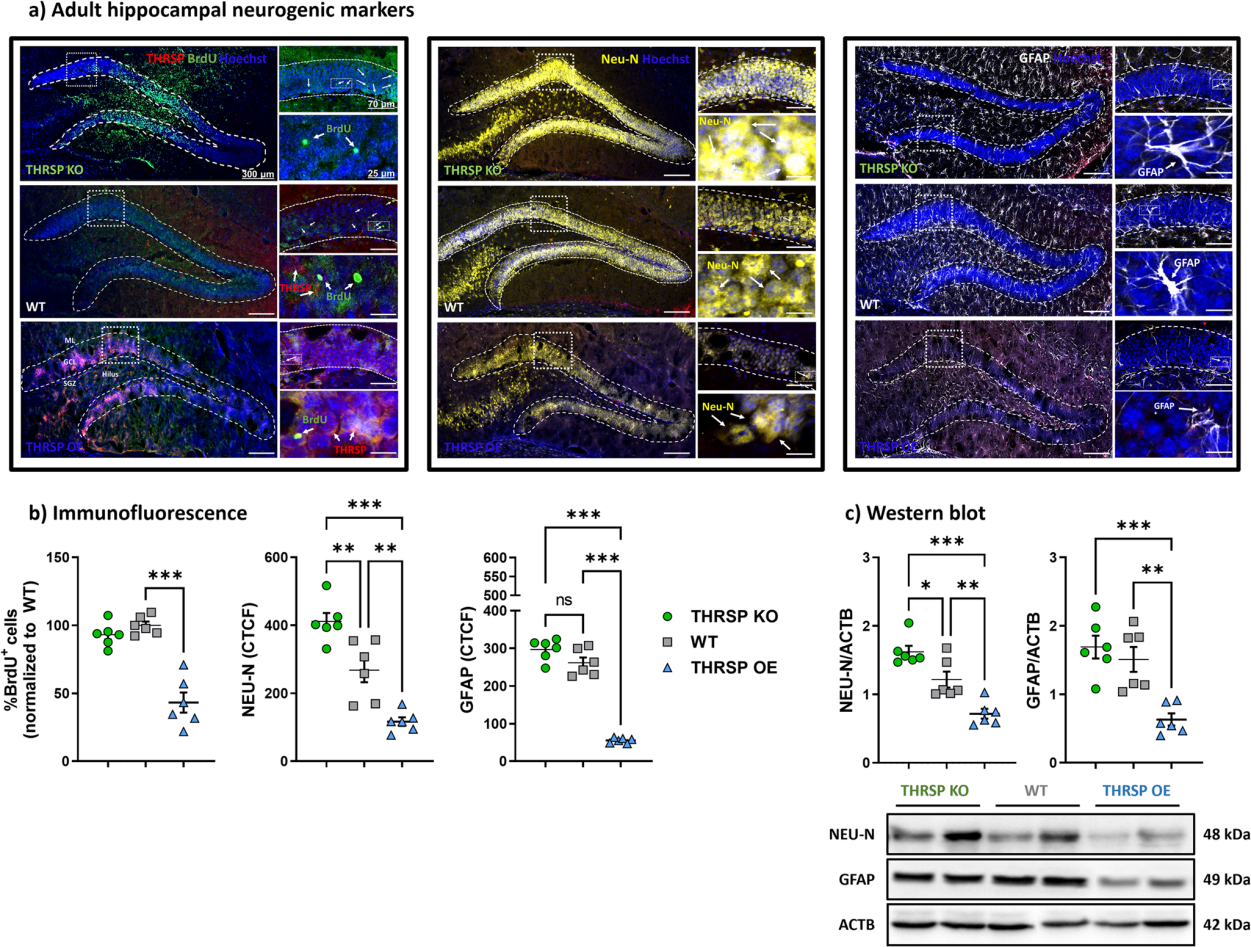
Hippocampal DG immunoreactivity of BrdU, NEU-N, and GFAP. **a** Representative fluorescence immunoreactivity of BrdU, NEU-N, and GFAP in mouse hippocampal DG, and their (**b**) corresponding analyses, and separate protein analyses using (**c**) western blotting. (*n* = 6 mice/group; **b** one-way ANOVA, %BrdU+ cells: *F* (_2, 15_) = 38.9, *P* < 0.001; NEU-N (CTCF): *F* (_2, 15_) = 32.0, *P* < 0.001; GFAP (CTCF): *F* (_2, 15_) = 147, *P* < 0.001). **c** One-way ANOVA, NEU-N/ACTB: *F* (_2, 15_) = 23.6, *P* < 0.001; GFAP/ACTB, *F* (_2, 15_) = 14.0, *P* < 0.001). Values are presented as the mean ± standard error of the mean (SEM). Data show impaired neural stem cell proliferation in THRSP OE mice. DG dentate gyrus, OE overexpressing, THRSP hyroid hormone-responsive protein.

Wnt signaling regulates crucial aspects of NSC activity. Accordingly, we determined whether dysregulation of Wnt signaling in THRSP OE mice can impact the expression of select neurogenic markers in the HPC, particularly in the DG, which participates in the NSC neurogenic process in the adult brain^35^. Immunofluorescence of HPC in THRSP OE mice presented a reduced percentage of DG cells expressing BrdU, along with decreased NEU-N and GFAP reactivity (Fig. 6a, b) when compared with that in THRSP KO and WT mice. Western blot analysis confirmed the reduced expression of NEU-N and GFAP (Fig. 6c) in THRSP OE mice, suggesting a possible maturational delay in NSCs and radial glial progenitor cells that could affect neuronal and glial formation in the nervous system during adult neurogenesis. In addition, these maturational delays in NSCs could have impacted the hippocampal-dependent behavioral performance of THRSP OE mice, resulting in inattention and memory impairment. Overall, the overexpression of THRSP in mice profoundly impacted the immunoreactivity and expression of neurogenic markers in the DG, implicated by impairments of Wnt/β-catenin signaling in THRSP OE mice.

### EE and treadmill exercise ameliorate behavioral deficits in THRSP OE mice accompanied by improvements in Wnt signaling and NSC activity

**Fig. 7:**
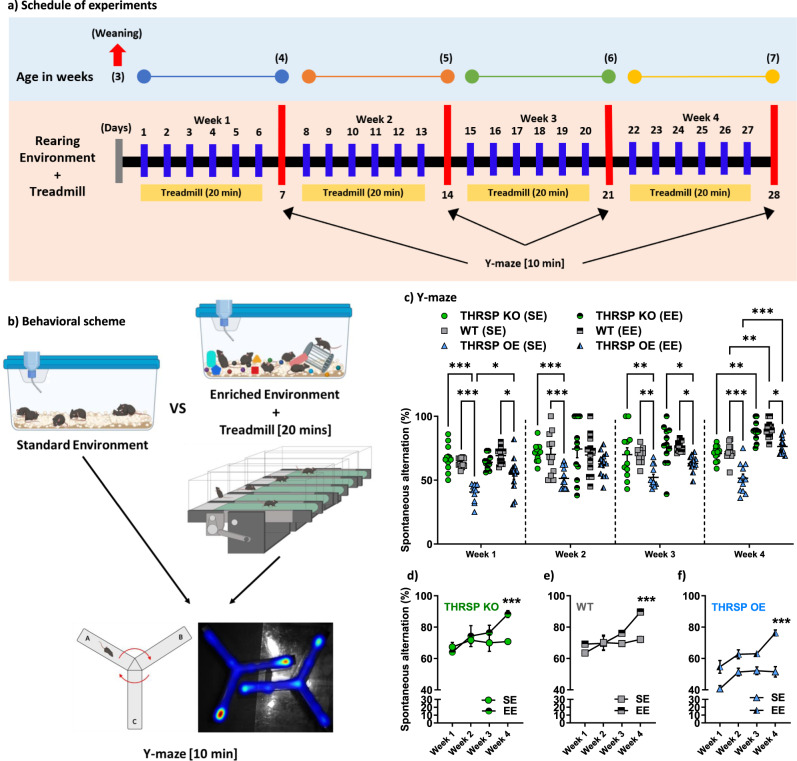
Effects of combined environmental enrichment and treadmill exercises on the ADHD-PI-like behavior in THRSP OE mice. **a** Experimental schedule showing age in weeks and the commencement of rearing environment + treadmill exercises in mice. **b** Behavioral scheme showing the representative image, with cages where mice were exposed (standard vs. enriched environment), treadmill, and representative Y-maze heatmap image. **c**, **d** The behavior of mice exposed to SE and EE throughout the 4-week exposure to rearing environment + treadmill exercises. (*n* = 12 mice/group; **c** one-way ANOVA, *F* (_5, 18_) = 7.62, *P* < 0.001); **d** two-way ANOVA (THRSP KO), Weeks: *F* (_1, 22_) =  6.32, *P* = 0.020; SE/EE exposure per strain: *F* (1.72, 37.8) = 3.99, *P* = 0.032; Weeks × SE/EE exposure per strain: *F* (_3, 66_) = 2.37, *P* = 0.078); **e** two-way ANOVA (THRSP WT), Weeks: *F* (_1, 44_) = 15.8, *P* < 0.001; SE/EE exposure per strain: *F* (_3, 44_) = 9.69, *P* < 0.001; Weeks ×  SE/EE exposure per strain: *F* (_3, 44_) = 4.16, *P* = 0.011); **f** two-way ANOVA (THRSP OE), Weeks: *F* (_1, 44_) = 46.5, *P* < 0.001; SE/EE exposure per strain: *F* (_3, 44_) = 19.5, *P* < 0.001; Weeks × SE/EE exposure per strain: *F* (_3, 44_) = 2.14, *P* = 0.109). Values are presented as the mean ±  standard error of the mean (SEM). Environmental enrichment and treadmill exercise ameliorate behavioral deficits in THRSP OE mice. ADHD-PI attention-deficit/hyperactivity disorder predominantly inattentive, EE enriched environment, KO knockout, OE (overexpressing, SE standard environment, THRSP thyroid hormone-responsive protein. The images used in (**b**) was created with BioRender.com.

Physiological effects of physical exercise on learning and memory have been demonstrated in human and animal models^36^, where regular physical exercise improves cognitive behavior by affording neuroprotective effects^37^. Conversely, exposure to physically, mentally, and sensory stimulating (enriched) environments can improve learning and memory impairment^38^. All mice were exposed to EE combined with physical exercise for four weeks to evaluate these effects (Fig. 7a, b). Each group was exposed to a SE. Subsequently, these mice were exposed to the Y-maze, and the percentage of spontaneous alternations was analyzed, revealing an enhancement in attention and memory in mice, as evidenced by increased spontaneous alternations (Fig. 7c). Early exposure to an EE and physical activity improved cognitive behavior in mice, particularly in THRSP OE mice. This confirmed that ADHD-PI behavior could be improved by combining early environmental enrichment and physical exercise, supporting the use of non-pharmacological interventions to alleviate the signs and symptoms of ADHD^39^.

**Fig. 8:**
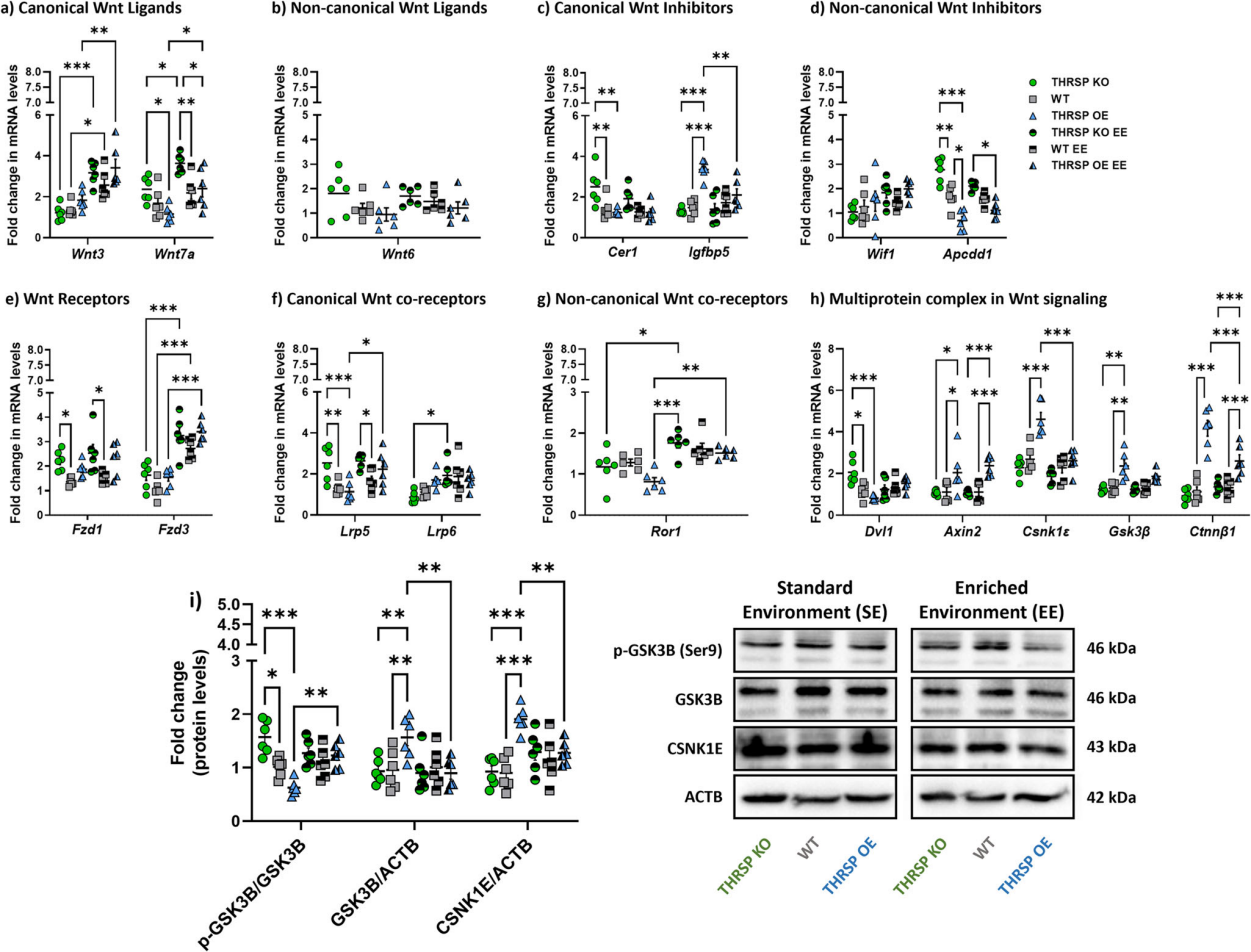
Expression of hippocampal DG Wnt pathway elements after environmental enrichment and treadmill exercises. qRT-PCR analyses of **a** canonical and **b** noncanonical Wnt ligands (*n* = 6 mice/group; **a** two-way ANOVA, Strain: *F* (_5, 60_) = 17.0, *P* < 0.001, Gene targets: *F* (_1, 60_) = 0.0245, *P* = 0.876; Strain × Gene targets: *F* (_5, 60_) = 4.51, *P* = 0.001; **b** one-way ANOVA, *F* (_5, 30_) = 1.82, *P* = 0.139), the **c** canonical and **d** noncanonical Wnt inhibitors (*n* = 6 mice/group; **a** two-way ANOVA, Strain: *F* (_5, 60_) = 4.35, *P* = 0.002, Gene targets: *F* (_1, 60_) = 5.72, *P* = 0.020; Strain × Gene targets: *F* (_5, 60_) = 4.51, *P* < 0.001; **b** two-way ANOVA, Strain: *F* (_5, 60_) = 5.18, *P* < 0.001, Gene targets: *F* (_1, 60_) = 1.63, *P* = 0.207; Strain × Gene targets: *F* (_5, 60_) = 11.1, *P* < 0.001), and the mRNA expression levels of **e** Wnt receptors, **f** canonical, **g** noncanonical Wnt co-receptors, and **h** the Wnt signaling multiprotein complex (*n* = 6 mice/group; **e** two-way ANOVA, Strain: *F* (_5, 60_) = 20.6, *P* < 0.001, Gene targets: *F* (_1, 60_) = 6.38, *P* = 0.014; Strain × Gene targets: *F* (_5, 60_) = 7.70, *P* < 0.001; **f** two-way ANOVA, Strain: *F* (_5, 60_) = 5.52, *P* < 0.001, Gene targets: *F* (_1, 60_) = 8.80, *P* = 0.004; Strain × Gene targets: *F* (_5, 60_)  = 5.81, *P* < 0.001); **g** one-way ANOVA, *F* (_5, 30_) = 8.30, *P* < 0.001; **f** two-way ANOVA, Strain: *F* (_5, 150_) = 40.6, *P* < 0.001, Gene targets: *F* (_4, 150_) = 45.6, *P* < 0.001; Strain × Gene targets: *F* (_20, 150_) = 9.67, *P* < 0.001). Confirmation of protein levels of some (**i**) mediators in the multiprotein complex (p-GSK3B, GSK3B, CSNK1E) (*n* = 6 mice/group; two-way ANOVA, SE/E exposure per strain: *F* (_5, 90_) = 3.58, *P* = 0.005, Gene targets: *F* (_2, 90_)  = 4.01, *P* = 0.022; SE/E exposure per Strain × Gene targets: *F* (_10, 90_) = 8.34, *P* < 0.001). Values are presented as the mean ± standard error of the mean (SEM). Environmental enrichment and treadmill exercise improve the Wnt signaling in mice. EE enriched environment, SE standard environment.

**Fig. 9:**
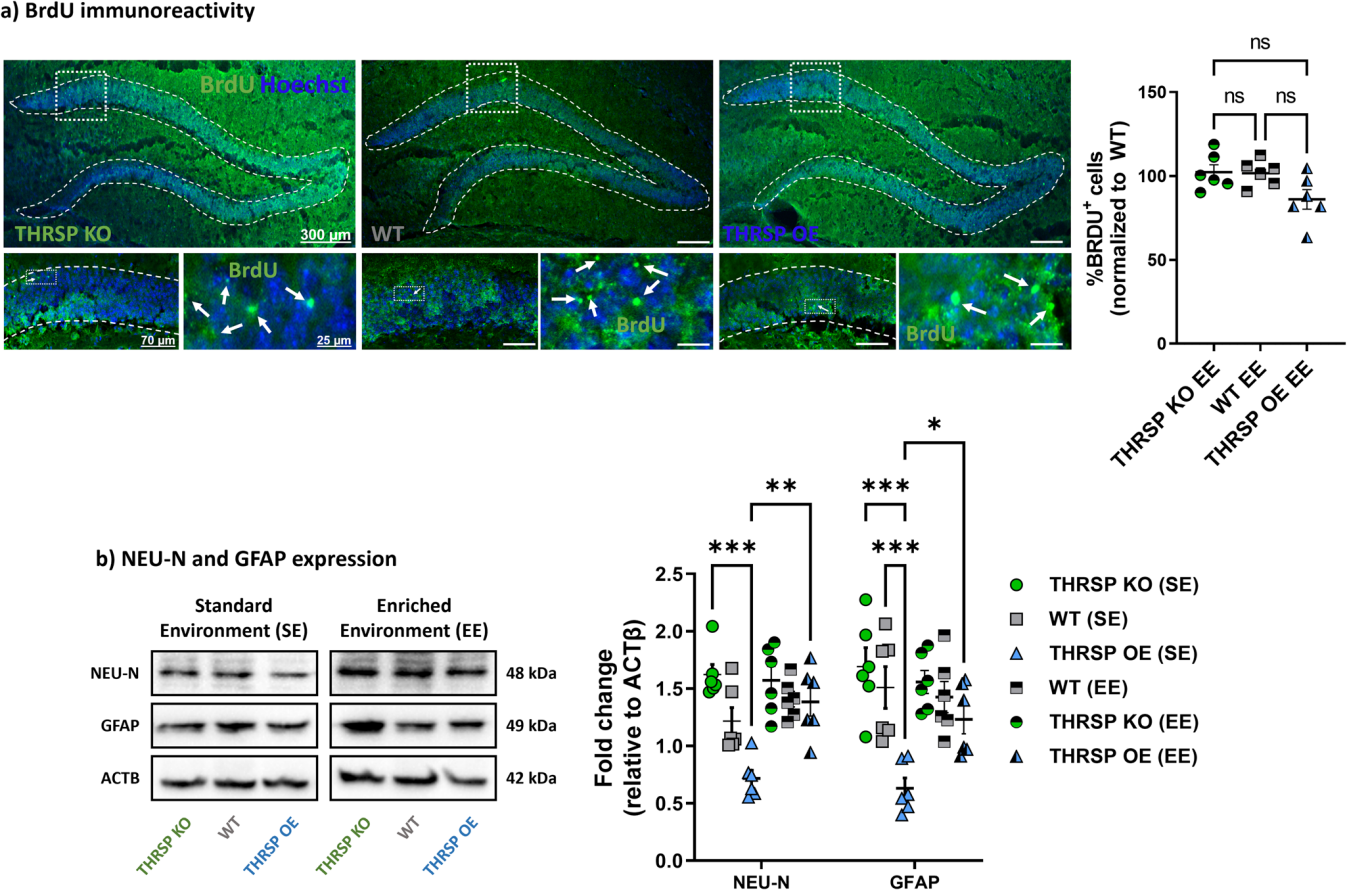
The BrdU, NEU-N, and GFAP immunoreactivity in mouse hippocampal DG after environmental enrichment and treadmill exercises. **a** BrdU immunoreactivity and **b** protein expression levels of NEU-N and GFAP in the mouse hippocampal DG. (*n* = 6 mice/group; **a** one-way ANOVA,*F* (_2, 15_) = 4.02, *P* = 0.040; **b** two-way ANOVA, SE/E exposure per strain: *F* (_5, 60_) = 16.6, *P* < 0.001, Protein targets: *F* (_1, 60_) =  0.0916, *P* = 0.763; SE/E exposure per strain × Protein targets: *F* (_5, 60_) = 0.820, *P* = 0.540). Values are presented as the mean ± SEM. Enriched environment and treadmill exercises improve in NSC activity in THRSP via the normalization of neurogenic markers. DG dentate gyrus, EE enriched environment, NSC neural stem cell, SE standard environment, THRSP thyroid hormone-responsive protein.

We identified the positive effects of combined environmental enrichment and physical exercise on attention and memory in mice, particularly in THRSP OE mice; herein, we aimed to investigate their effects on Wnt signaling and NSC activity. Compared with SE-exposed mice, groups exposed to combined EE and treadmill exercises showed improvement in the genetic markers of Wnt signaling. The expression levels of canonical *Wnt3* and *Wnt7a* ligands were improved (Fig. 8a) in all strains, with accompanying reduction in canonical and noncanonical *Cer1* and *Igfbp5* Wnt inhibitors (Fig. 8c, d) in THRSP KO and OE, respectively. Interestingly, we noted an increase in Wnt receptors (*Fzd3*) (Fig. 8e) and co-receptors (*Lrp5*, *Lrp6*) (Fig. 8f) and signs of stabilization in the multiprotein complex targets by reducing the *Csnk1ɛ*, *Gsk3β*, and *Ctnnβ1* gene and protein expression levels (Fig. 8h, i). These results indicated that Wnt signaling activity was enhanced in the hippocampal DG region of mice exposed to combined EE and physical exercise, which may be related to improved attention and memory in mice during the Y-maze test. Furthermore, we detected the normalization of BrdU immunoreactivity (Fig. 9a) in THRSP OE mice, showing comparable percentages of DG cells expressing BrdU. Interestingly, NEU-N and GFAP expression improved (Fig. 9b), particularly in THRSP OE mice. Collectively, these findings suggested a relationship between the inherent impairment of Wnt signaling, reduced NSC activity, inattention, and memory impairment in THRSP OE mice, which were improved by a combination of EE and treadmill exercises.

## Discussion

It is interesting to note how functional overexpression and deletion of a certain gene, in this case, THRSP, would induce global differences not only in the behavior of mice but also in their molecular make-up. However, given that little is known regarding the function of THRSP in behavior, it remains unclear how genetic modifications in THRSP expression, particularly overexpression, but not KO, can induce ADHD-PI behavior. Two factors have been identified in our previous studies, i.e., dopaminergic^27^ and thyroid hormone^28^ aberrations, which are known to trigger ADHD development. The present findings indicating the involvement of impaired Wnt signaling afford a better understanding of the role of THRSP and how it induces ADHD-PI. One distinct functional role of THRSP is lipogenesis, where it acts as a lipogenic activator that eventually regulates the expression of several lipogenic genes^40^. This finding is of particular interest, given the role of lipogenesis in NSC activity.

In a key study, de novo lipogenesis, particularly fatty acid synthase (FASN) activity, was shown to be specifically elevated in mouse NSCs when compared with differentiated neuronal cells^41^. Lipogenesis is required for stem cell proliferation and, therefore, for normal neurogenesis to proceed. In contrast, THRSP and Spot14 have been identified as repressors of NSC proliferation^41^. THRSP decreases FASN activity by dimerizing with Mid1-Interacting G12-Like protein (MIG12), a THRSP homolog and activator of acetyl-CoA carboxylase (ACC), thereby inhibiting its function and decreasing ACC-mediated malonyl-CoA production, leading to a reduction in fatty acid synthesis^41^. Notably, cholesterol biosynthesis, known to occur through an independent pathway, is also required for NSC self-renewal and maintenance in the developing mouse forebrain, given that NSCs with mutations in enzymes involved in this pathway exhibit premature differentiation into neurons, thereby exhausting the stem cell pool^42^.

Using in situ hybridization, THRSP-expressing cells were shown to constitute neural stem progenitor cells^41,43^, confined to the adult brain^44^ such as the sub-granular zone of the hippocampal DG, which is highly neurogenic. Some progenitor cells are highly restricted to regions of the adult brain, such as the DG, which plays a pivotal role in ensuring lifelong neurogenesis in the mammalian brain, necessary for improved learning, memory, and overall cognitive abilities^45^. Indeed, changes in the production, growth, and overall regulation of new neurons in neurogenic brain regions have been associated with neuropsychiatric disorders, including ADHD^46,47^. Remarkably, retroviral THRSP overexpression reduced hippocampal neural stem progenitor cell proliferation when compared with that induced by THRSP knockdown, indicating that THRSP plays a role in NSC modulation essential for neurogenesis. Therefore, THRSP overexpression and KO in mice potentially influence the expression of neuronal markers, indicative of the state of NSC activity in these transgenic mice, where THRSP OE mice exhibit reduced BrdU immunoreactivity with low NEU-N and GFAP protein expression when compared with THRSP KO mice with enhanced BrdU+ cells and high NEU-N and GFAP expression. These effects might lead to altered or improved inattention or memory, which is evident in THRSP transgenic mice. In addition, THRSP is highly responsive to thyroid hormones, such as the neurogenic hippocampal DG.

Moreover, recent evidence has revealed that Wnt/β-catenin signaling participates in the regulation of thyroid hormone receptors, deiodinases, and transporter expression in target tissues, thereby affecting the transcriptional mechanisms^48^. Therefore, altered THRSP expression can induce aberrations, such as low thyroid hormone T3 levels. We have previously documented reduced T3 levels due to innately low monocarboxylate transporter 8 (MCT8) in these transgenic mice^28^, improved by thyroid hormone replacement (i.e., triiodothyronine/T3 [10 mg kg^−1^], levothyroxine/T4 [10 mg kg^−1^]), as evidenced by improvements in attention and memory behavior and theta wave normalization. Subsequently, this can alter the regulation of neuronal markers, as detected in the present study.

Our study is not the first to identify the role of impaired Wnt signaling in ADHD. However, to the best of our knowledge, this is the first study to use a specific animal model that could potentially improve our understanding of ADHD-PI. However, caution should be used when interpreting the observed findings, as only the hippocampal DG proteomes were evaluated, which may result in discrepant findings. Nonetheless, a few findings using in vitro, preclinical, and clinical samples have been previously reported, which can support and corroborate our findings. Among these, enhanced neuronal differentiation in cell models (i.e., murine neural stem, rat PC12, and human SH-SY5Y cells) has been associated with activation of Wnt signal transduction pathways^17^, induced by methylphenidate (Ritalin), a commonly used ADHD medication. We have previously reported that treatment with methylphenidate (5 mg kg^−1^) could improve inattention during the Y-maze and novel-object recognition in THRSP OE mice^27^ This improved behavior in THRSP OE mice following methylphenidate injection could be attributed to Wnt signaling, as the classical G-protein-coupled receptors and canonical Wnt pathways interact with each other by sharing several intermediate signaling components. Recent in vivo studies have revealed that antipsychotic drugs, known to dopamine D2-like receptors, increase cellular levels of downstream signaling components of canonical Wnt pathways, such as *Dvl*, *Gsk3β*, and *Ctnnβ1*, indicating functional interactions between the Wnt pathway and D2-like receptors^49^. Interestingly, these genetic markers were altered in THRPS-OE mice.

In addition, the striatal transcriptomic analysis of an ADHD mouse model displaying hyperactivity and motor impulsivity revealed a pattern of synaptic remodeling. Subsequently, multiple genes were predicted to downregulate canonical Wnt pathways^50^. Lastly, an association study and meta-analysis have evaluated the involvement of canonical Wnt signaling LRP5 and LRP6 receptor gene variants in ADHD^31^. Interestingly, genetic variations in LRP5 intronic rs4988319 and rs3736228 (Ala1330Val) were observed among young females, whereas LRP6 rs2302685 (Val1062Ile) variations were observed in young males, indicating a potential sex-specific link between LRP5 and LRP6 gene variants in ADHD. In general, these recent findings support the hypothesis that genes involved in Wnt signaling might affect the physiology and predispose THRSP OE mice to ADHD-like symptoms. However, the degree to which the transgenic nature in THRSP OE mice influenced the eventual Wnt signaling impairment and ADHD-PI-like endophenotype, and the lack thereof in THRSP KO, was not fully examined. However, based on the findings obtained, we can deduce that the proteomics changes, particularly those involved in Wnt signaling, may have been innately caused by the genetic overexpression of THRSP in THRSP OE mice that subsequently affected their behavior in the Y-maze test.

Previous studies have shown that an active lifestyle involving regular exercise improves brain function, in which both synaptic plasticity and neurogenesis are modulated. In the mature brain, the canonical Wnt signaling pathway has been implicated in neuroprotection and synaptic plasticity. Herein, Wnt signaling was enhanced in THRSP OE mice, as evidenced by improvements in the expression of Wnt signaling-related markers that were previously altered. Moreover, long-term environmental enrichment accompanied by treadmill exercise improved the attention and memory of THRSP OE mice, concomitant with an improvement in Wnt signaling. Mice in the SE group showed lower expression levels of canonical Wnt ligands (i.e., *Wnt3* and *Wnt4*) than the EE + treadmill groups, which exhibited higher expression levels. In addition, we noted improvements in the expression of Wnt receptors and co-receptors, particularly *Fzd3* and *Lrp5* mRNA levels, respectively, with low levels of Wnt inhibitors (i.e., *Igfbp5, Apcdd1*). Analysis of some components of the multiprotein complex implicated in the phosphorylation of β-catenin, resulting in either its proteasomal degradation or activation, exhibited low levels or reduced trends in *Dvl1*, *Axin2*, *Csnk1ε*, *Gsk3β*, and *Ctnnβ1* mRNA in mice exposed to EE and treadmill exercise when compared with SE-exposed mice. Western blot analyses of p-GSK3B (Ser9) against basal GSK3B confirmed the high activity of the GSK3B basal form in THRSP OE SE-exposed mice; this was reversed by environmental enrichment and treadmill exercises, subsequently affecting the expression of β-catenin. Typically, Wnt ligands bind to cell surface-FZD/LRP5-6 receptor complexes, which then bind to *Dvl*, causing activation of the multiprotein complex or “destruction complex”; this, in turn, displaces GSK3B, preventing the phosphorylation and degradation of CTNNB1. Thus, our results suggest that activation of the Wnt signaling pathway induced by environmental enrichment and treadmill exercises enhances the behavior of THRSP OE mice, which is corroborated by a previous study^51^.

Compounding the difficulty of selecting an ideal model to study specific presentations of ADHD is a simple fact that our knowledge regarding ADHD neurobiology is insufficient. Accordingly, the current experiment has explored the potential genetic/protein changes innate in THRSP OE mice, a potential animal model of ADHD-PI, that shows an acceptable *face*, *predictive*, and *construct* validity. The Thrsp gene could be a potential biomarker for ADHD-PI presentation, and THRSP OE mice could represent a useful animal model for studying this distinct presentation of ADHD. We believe that using THRSP OE mice may provide an understanding of the underlying pathological mechanism of ADHD-PI and could stimulate the development of more tailored intervention strategies. Also, as observed recently, the shared thyroid hormones and dopamine impairment is influenced by THRSP overexpression, which may be central to its behavioral impairment. However, additional investigations are required to confirm this. Ultimately, to date, there have been no relevant genome-wide association studies. The occurrence of *THRSP* single-nucleotide polymorphisms in ADHD patients should be assessed to further verify the utility of *THRSP* as a biomarker for this disorder and to validate the contribution of THRSP OE mice as an animal model for the ADHD-PI presentation. Nevertheless, the overexpression and knockout of the THRSP gene in mice innately altered some of the genes involved in signaling pathways, such as those involved in Wnt signaling, previously found to be impaired in ADHD.

### Conclusions

Our study provides significant findings that could be valuable in advancing ADHD research. We identified hippocampal DG molecular signatures in an ADHD-PI mouse model that overexpressed THRSP rather than typical “culprits”, such as the dopaminergic hypothesis in ADHD or thyroid hormone dysfunction, also identified in our previous studies. Our current results converge with the ancient and evolutionary Wnt signaling pathways crucial for cell fate determination, migration, polarity, and neural patterning during neurodevelopment. These findings support the role of Wnt signaling in neurological disorders, particularly ADHD. Moreover, the integration of environmental enrichment with treadmill exercise has proven effective in improving not only behavior but also some altered molecular aspects in the hippocampal DG of THRSP OE mice, supporting the benefits conferred by non-pharmacological interventions, such as environmental modifications and exercise, in improving the signs and symptoms of ADHD.

Overall, our previous and present findings provide a clearer understanding of the validity of THRSP OE mice as an animal model for ADHD-PI presentation showing *face* (similarity in symptoms), *predictive* (similarity in response to treatment or medications), and *construct* (similarity in etiology or underlying pathophysiological mechanism) validity^52^.

## Methods

### Animals

Herein, we used two THRSP transgenic male mouse lines, OE and knockout (KO) mice^28^, excluding the THRSP heterozygous (THRSP HET) mice, and their wild-type (WT) counterparts (Fig. 1a), confirmed using DNA electrophoresis (genotyping). These lines were continuously bred and maintained at the Uimyung Research Institute for Neuroscience (Laboratory of Pharmacology, Sahmyook University)^27,28^ animal facility until the appropriate experimental age was reached. In addition, non-transgenic littermates were used as WT counterparts. Briefly, mice were housed in a temperature- and humidity-controlled environment (temperature, 22 ± 2 °C; relative humidity, 55 ± 5%) under a 12/12 h light/dark (07:00–19:00 h light) cycle. All standard animal care and procedures were performed following the Principles of Laboratory Animal Care (NIH Publication No. 85-23, revised 1985), the Animal Ethics Review Board of Sahmyook University, South Korea (SYUIACUC2020-010), and in compliance with the 3Rs framework^53^ and ARRIVE guidelines^54^ recommended by Communications Biology.

As presented, all mice used in the experiment were male. Unfortunately, we did not use female samples in our experiments for two reasons also presented in our previous studies^27,28^. First, this study focused only on male mice, given the clear sex differences in ADHD subtypes, prevalence, and specific DSM-5 ADHD symptoms showing that males are more prevalent to be diagnosed with ADHD than females^55^, hence the use of only male mice. Nevertheless, it would be valuable to identify sex differences in the future, considering the potential variations in the thyroid hormone levels between male and female mice, particularly those of transgenic nature^27,28^. Second, female mice of each strain followed continuous breeding to maintain the transgenic lines and preserve significant samples for our experiments; hence, male mice were utilized for all succeeding experiments. Furthermore, all mice for each strain (KO, OE, WT) tested were of the same number per group. Regarding the litter size, three weeks before the experiments, genotyping was conducted to determine transgenic mice and separate them from their non-transgenic littermates. Depending on the size of the litter and the resultant number of transgenic and non-transgenic (WT) mice within the litter, we may use mice from different litters, between 1 and 3 litters of the same strain and age, for the same experiment.

### EXPERIMENT 1: Evaluation of potential genetic targets affecting ADHD-PI behavior

#### Y-maze

Prior to proteomic and other molecular analyses of the hippocampal DG, 7-week-old mice (between postnatal [PND] 49 or 50) (*n* = 18) were examined in the Y-maze test to confirm the impaired behavior (i.e., inattention, impaired memory) in adult THRSP OE and the absence thereof in THRSP KO mice, compared with WT mice, which were previously observed^27,28^. The use of 7-week-old or PND 49 or 50 mice was based on a previous study that identified the chronometry of species, indicating full body growth completion by PND 50^56^. Accordingly, we employed an arbitrary date of PND 49 or 50 as a reasonable age for an adult mouse.

Briefly, each mouse was placed in one arm of the Y-maze (45 × 10 × 20 cm), allowed to explore freely for 10 min, and recorded using Ethovison XT (RRID: SCR_000441; Noldus, Netherlands). “Arm entry” was defined as the entry of all four paws (mice) into an arm and the ‘alternation behavior’ (actual alternations) as a consecutive entry into three arms. The percentage of spontaneous alternation was calculated as the ratio of actual alternations to the maximum number of alternations (total number of arm entries minus two) multiplied by 100 (% alternation = [(number of alternations)/(total arm entries − 2)] × 100).

#### Brain extraction

Herein, brain samples (*n* = 18) (Fig. 1a) were randomly assigned for use in proteomics analysis, quantitative reverse transcription-polymerase chain reaction (qRT-PCR), western blotting, and immunofluorescence. For the first six samples, left and right hippocampal DG were snap-frozen, enclosed in a dry ice-filled box, and transported to the SNU laboratory for proteomic analysis. Next, we again divided the second set of hippocampal DG samples into left and right slices for qRT-PCR and western blotting (*n* = 6), respectively, while the remaining brain samples were utilized for immunofluorescence (*n* = 6) targeting the hippocampal DG. (Note: hippocampal DG utilized in proteomics, qRT-PCR, and western blotting were isolated following the Allen mouse brain atlas coordinates^57^).

#### Proteomic analysis

Proteins (Fig. 1b) were extracted from mouse hippocampal DG brain tissues using radioimmunoprecipitation assay (RIPA) buffer and quantified using the bicinchoninic acid protein assay. Briefly, 200 µg of each sample were resolved by sodium dodecyl sulfate (SDS)-polyacrylamide gel electrophoresis under reducing conditions and stained with Instant Blue Coomassie protein stain. Each lane was divided into ten individually processed segments for in-gel protein digestion. Stained gel fragments were cut into small pieces, washed with 100 mM ammonium bicarbonate (NH_4_HCO_3_), and dehydrated with 50% (v/v) acetonitrile (ACN) in 25 mM ammonium bicarbonate. The reduction was performed by incubating samples with 20 mM dithiothreitol (DTT) for 1 h at 60 °C, followed by alkylation with 55 mM iodoacetamide for 45 min in the dark. After washing and dehydration with acetonitrile, gel pieces were covered with 12.5 ng/μL trypsin in 50 mM NH_4_HCO_3_, and digestion was performed overnight at 37 °C. Peptide extraction was carried out by incubation at 37 °C with 10% formic acid (FA) and further incubation with 50% ACN in 0.1% FA and 80% ACN in 0.1% FA. Eluted peptides were dried in a SpeedVac and stored at −20 °C until further use.

#### Liquid chromatography with tandem mass spectrometry (LC/MS/MS) and data analysis

Peptides (Fig. 1b) were resuspended in solvent A (0.1% FA), loaded into an analytical column, and separated with a linear grade 5–35% solvent B (0.1% FA in 98% ACN) for 95 min at a flow rate of 300 nL/min. The MS spectra were recorded on a Q-Exactive plus hybrid quadrupole-orbitrap MS coupled with an Ultimate 3000 HPLC system. The standard mass spectrometric condition of the spray voltage was set to 2.0 kV, and the temperature of the heated capillary was set to 250 °C. Full scans were acquired in the range of 400–1400 *m/z* with 70,000 resolutions, and the normalized collision energy was 27% and 17,500 resolutions for high-energy collision dissociation fragmentation. The data-dependent acquisition was performed with a single survey MS scan, followed by 10 MS/MS scans with a dynamic exclusion time of 30 s. The mass spectrometry proteomics data have been deposited to the ProteomeXchange Consortium via the PRIDE^58^ partner repository with the dataset identifier PXD038525 and 10.6019/PXD038525. The collected MS/MS raw data were converted into mzXML files using engine-based PEAKS Studio. Protein identification was performed using the UniProt-Musculus database, setting the precursor mass tolerance to 10 ppm and fragment mass tolerance to 0.8 Da. Oxidized methionine was considered a variable amino acid modification, and carbamidomethylating of cysteine was deemed a fixed modification. Trypsin was selected as the enzyme, allowing up to two missed cleavages. Peptide and protein identifications were further filtered to <1% FDR, as measured using a concatenated target-decoy database search strategy. Relative protein quantitation samples were analyzed using the Power Law Global Error Model (PLGEM) (http://www.bioconductor.org) package within the R program (version 3.4.2). PLGEM can control datasets to distinguish statistically significant DEPs and calculate expression level changes by *P* value and signal-to-noise ratio. Ingenuity pathway analysis was performed using all significantly altered proteins (Fig. 1c, Tables 1–3, and Supplementary Tables 1–4).

#### Gene ontology (GO) analysis

Analyses of GO biological processes and enriched pathways were performed using the GENEONTOLOGY/PANTHER classification system (v.17)^59^. We used upregulated and downregulated DEPs from THRSP KO (Fig. 2a, b) and OE (Fig. 3a, b) mice relative to WT mice to analyze GO biological processes. In contrast, the total DEPs (upregulated and downregulated) were used for GO enrichment analysis (Fig. 2c).

#### Search tool for the retrieval of interacting genes/proteins (STRING) analysis

To investigate the biological relevance of identified DEPs, protein–protein interaction networks were generated using STRING (v.11.5)^60^. The graphical representation of protein networks in THRSP KO (Fig. 2d) and OE (Fig. 3d) mice was restricted to high-confidence interactions with a score interaction threshold of 0.9, excluding non-interacting proteins.

#### RNA extraction and real-time quantitative reverse transcription PCR (qRT-PCR)

Total RNA was isolated using TRIzol™ Reagent (Invitrogen, Carlsbad, CA, USA). A Hybrid-RTM Kit (Geneall Biotechnology, Seoul, Korea) was used for RNA purification. The total RNA concentration was determined with a Colibri Microvolume Spectrometer (Titertek-Berthold, Pforzheim, Germany).

qRT-PCR was used to measure mRNA expression levels of *Thrsp* (Fig. 1a) and those involved in Wnt signaling (upstream/downstream) (Figs. 4a–g and 5a), based on our previous report^61^. One microgram (µg) of total RNA was reverse transcribed into cDNA using AccuPower CycleScript RT Premix (Bioneer, Seoul, Korea). The cDNA amplification was performed using custom-made sequence-specific primers (Cosmogenetech, Seoul, Korea) (thyroid hormone-responsive protein (*Thrsp*), F: 5’-ATGCAAGTGCTAACGAAACGC-3’, R: 5’-CCTGCCATTCCTCCCTTGG-3’; Wnt family member 3 (*Wnt3*), F: 5’-CTCGCTGGCTACCCAATTTG-3’, R: 5’-CTTCACACCTTCTGCTACGCT-3’; Wnt family member 4 (*Wnt4*), F: 5’-AGACGTGCGAGAAACTCAAAG-3’, R: 5’-GGAACTGGTATTGGCACTCCT-3’; Wnt family member 6 (*Wnt6*), F: 5’-GCAAGACTGGGGGTTCGAG-3’, R: 5’-CCTGACAACCACACTGTAGGAG-3’; Wnt family member 7a (*Wnt7a*), F: 5’-TCAGTTTCAGTTCCGAAATGGC-3’, R: 5’- CCCGACTCCCCACTTTGAG-3’; Wnt family member 8b (*Wnt8b*), F: 5’-CCCGTGTGCGTTCTTCTAGTC-3’, R: 5’-AGTAGACCAGGTAAGCCTTTGG-3’; Wnt family member 11 (Wnt11), F: 5’-GCTGGCACTGTCCAAGACTC-3’, R: 5’-CTCCCGTGTACCTCTCTCCA-3’; Cerberus 1 (*Cer1*), F: 5’-CTCTGGGGAAGGCAGACCTAT-3’, R: 5’-CCACAAACAGATCCGGCTT-3’; Dickkopf Wnt signaling pathway inhibitor 1 (*Dkk1*), F: 5’-CTCATCAATTCCAACGCGATCA-3’, R: 5’-GCCCTCATAGAGAACTCCCG-3’; Dickkopf Wnt signaling pathway inhibitor 4 (*Dkk4*), F: 5’-GTACTGGTGACCTTGCTTGGA-3’, R: 5’-CCGTTCATCGTGAAACGCTAAG-3’; Secreted frizzled related protein 2 (*sFrp2*), F: 5’-CGTGGGCTCTTCCTCTTCG-3’, R: 5’-ATGTTCTGGTACTCGATGCCG-3’; Secreted frizzled related protein 5 (*sFrp5*), F: 5’-CACTGCCACAAGTTCCCCC-3’, R: 5’-TCTGTTCCATGAGGCCATCAG-3’; Shisa family member 4 (*Shisa4*), F: 5’-GGACTGCTTGTGGTATCTGGA-3’, R: 5’-CGGTGATGAGTAAGGTCAGGT-3’; Shisa family member 9 (*Shisa9*), F: 5’-CTCCTGTCGGGGCTACTTC-3’, R: 5’-CCGCTTCTTAAACGTGCAGC-3’; insulin-like growth factor binding protein 1 (*Igfbp1*), F: 5’-ATCAGCCCATCCTGTGGAAC-3’, R: 5’-TGCAGCTAATCTCTCTAGCACTT-3’; Insulin-like growth factor binding protein 1 (*Igfbp5*), F: 5’-CCCTGCGACGAGAAAGCTC-3’, R: 5’-GCTCTTTTCGTTGAGGCAAACC-3’; Wnt inhibitory factor 1 (*Wif1*), F: 5’-TCTGGAGCATCCTACCTTGC-3’, R: 5’-ATGAGCACTCTAGCCTGATGG-3’; Sclerostin (*Sost*), F: 5’-AGCCTTCAGGAATGATGCCAC-3’, R: 5’-CTTTGGCGTCATAGGGATGGT-3’; sclerostin domain containing 1 (*Sostdc1*), F: 5’-CCTGCCATTCATCTCTCTCTCA-3’, R: 5’-CCGGGACAGGTTTAACCACA-3’; adenomatosis polyposis coli downregulated 1 (*Apcdd1*), F: 5’-CTTCACGGCGTCCAAGTCAT-3’, R: 5’-GCAAGTTCGGTTCACCAGTC-3’; frizzled class receptor 1 (*Fzd1*), F: 5’-CAGCAGTACAACGGCGAAC-3’, R: 5’-GTCCTCCTGATTCGTGTGGC-3’; frizzled class receptor 3 (*Fzd3*), F: 5’-ATGGCTGTGAGCTGGATTGTC-3’, R: 5’-GGCACATCCTCAAGGTTATAGGT-3’; low-density lipoprotein receptor-related protein 1 (*Lrp1*), F: 5’-ACTATGGATGCCCCTAAAACTTG-3’, R: 5’-GCAATCTCTTTCACCGTCACA-3’; low-density lipoprotein receptor-related protein 5 (*Lrp5*), F: 5’-AAGGGTGCTGTGTACTGGAC-3’, R: 5’-AGAAGAGAACCTTACGGGACG-3’; low-density lipoprotein receptor-related protein 6 (*Lrp6*), F: 5’-TTGTTGCTTTATGCAAACAGACG-3’, R: 5’-GTTCGTTTAATGGCTTCTTCGC-3’; low-density lipoprotein receptor-related protein 10 (*Lrp10*), F: 5’-GGATCACTTTCCCACGTTCTG-3’, R: 5’-GAGTGCAGGATTAAATGCTCTGA-3’; receptor tyrosine kinase-like orphan receptor 1 (*Ror1*), F: 5’-TGAGCCGATGAATAACATCACAA-3’, R: 5’-CAGGTGCATCATTCTTGAACCA-3’; receptor tyrosine kinase-like orphan receptor 2 (*Ror2*), F: 5’-ATCGACACCTTGGGACAACC-3’, R: 5’-AGTGCAGGATTGCCGTCTG-3’; receptor like tyrosine kinase (*Ryk*), F: 5’-GGTCTTGATGCAGAGCTTTACT-3’, R: 5’-CCCATAGCCACAAAGTTGTCTAC-3’; disheveled segment polarity protein 1 (*Dvl1*), F: 5’-ATGAGGAGGACAATACGAGCC-3’, R: 5’-GCATTTGTGCTTCCGAACTAGC-3’; disheveled segment polarity protein 2 (*Dvl2*), F: 5’-GGTGTAGGCGAGACGAAGG-3’, R: 5’-GCTGCAAAACGCTCTTGAAATC-3’; disheveled segment polarity protein 3 (*Dvl3*), F: 5’-GTCACCTTGGCGGACTTTAAG-3’, R: 5’-AAGCAGGGTAGCTTGGCATTG-3’; axin 2 (*Axin2*), F: 5’-TGACTCTCCTTCCAGATCCCA-3’, R: 5’-TGCCCACACTAGGCTGACA-3’; adenomatous polyposis coli regulator of Wnt signaling pathway (*Apc*), F: 5’-CTTGTGGCCCAGTTAAAATCTGA-3’, R: 5’-CGCTTTTGAGGGTTGATTCCT-3’; Apc regulator of Wnt signaling pathway 2 (*Apc2*), F: 5’-CACACAGTTTGACCATCGTGA-3’, R: 5’-GTGGACGAGGTTGCGTAGC-3’; casein kinase 1 alpha-1 (*Csnk1α1*), F: 5’-TCCAAGGCCGAATTTATCGTC-3’, R: 5’-ACTTCCTCGCCATTGGTGATG-3’; casein kinase 1 epsilon (*Csnk1ε*), F: 5’-ATGGAGTTGCGTGTGGGAAAT-3’, R: 5’-ACATTCGAGCTTGATGGCTACT-3’; glycogen synthase kinase 3 beta (*Gsk3β*), F: 5’-TGGCAGCAAGGTAACCACAG-3’, R: 5’-CGGTTCTTAAATCGCTTGTCCTG-3’; catenin beta-1 (*Ctnnβ1*), F: 5’-ATGGAGCCGGACAGAAAAGC-3’, R: 5’-CTTGCCACTCAGGGAAGGA-3’;) and was detected with SYBR Green (Solgent, Korea).

qRT-PCR analysis was performed in duplicate, and values were normalized to mRNA levels of the housekeeping gene Actin beta (*Actb*), F:5’- GGCTGTATTCCCCTCCATCG-3’, R:5’-CCAGTTGGTAACAATGCCATGT-3’]. The relative expression levels were calculated using the 2-^ΔΔCt^ method.

#### Western blotting

Western blotting was performed to evaluate the expression levels of specific Wnt signaling targets and neuronal markers in the mouse hippocampal DG region (Figs. 5b–e and 6c). Western blotting protocols were performed in accordance with those utilized in our previous studies^27,62^. Briefly, 30 μg of protein lysates were loaded and electrophoresed on 10% sodium dodecyl sulfate and polyacrylamide gels and subsequently transferred onto nitrocellulose membranes. Membranes were blocked with 5% bovine serum albumin in Tris-buffered saline with Tween-20 (TBST) for 1 h and incubated overnight with the following primary antibodies: rabbit polyclonal anti-phosphorylated-GSK3B (1/1000; Cell Signaling Technology Cat# 9336, RRID:AB_331405), rabbit monoclonal anti-GSK3B (1/1000; Cell Signaling Technology Cat# 9336, RRID:AB_331405), rabbit polyclonal anti-CSNK1E (1/1000; Cell Signaling Technology Cat# 12448, RRID:AB_2797919), rabbit polyclonal anti-GFAP (1/1000; LSBio, catalog number: LS-B15993), mouse monoclonal anti-NEU-N (1/1000; LSBio [LifeSpan] Cat# LS-C312122, RRID:AB_2827517). The standard control was performed using mouse monoclonal anti-ACTB (1/5000; Sigma-Aldrich Cat# A5441, RRID:AB_476744). Subsequently, the blots were washed in TBST and incubated with the appropriate HRP-conjugated anti-rabbit (1/3000; Bio-Rad Cat# 170-6515, RRID:AB_11125142) and anti-mouse (1/5000; Bio-Rad Cat# 170-6516, RRID:AB_11125547) secondary antibodies for 1 h. After three final washes with TBST, blots were visualized using enhanced chemiluminescence (Clarity Western ECL; Bio-Rad Laboratories) in a ChemiDoc Imaging System (Bio-Rad ChemiDoc MP Imaging System, RRID:SCR_019037). Original blots are shown in Supplementary Fig. 1.

#### 5-BrdU injection

To label cell proliferation or mitotic cells, all groups were injected with 50 mg/kg 5-Bromo-2-deoxyuridine (5-BrdU) (Cat# HY-15910 MedChemExpress, Monmouth Junction, NJ, USA) once daily for 6 days. On the last day(day 7), all groups were injected with 100 mg/kg 5-BrdU after behavioral experiments or 3 h prior to brain extraction. 5-BrdU was dissolved in 0.7% dimethyl sulfoxide (DMSO) and 1% Tween-80 in 0.9% saline solution and administered intraperitoneally (i.p.).

#### Immunofluorescence

Briefly, 5-BrdU-injected mice (*n* = 6/group) were sacrificed immediately after the last day of the respective experiments. Standard protocols for brain fixation were followed^63^. Briefly, mice were anesthetized using tiletamine/zolazepam (50 mg mL^−1^); Zoletil; Vibrac Laboratories, Carros, France) and xylazine (100 mg mL^−1^). Using the intracardiac route, mice were perfused with a perfusion solution (0.05 M phosphate-buffered saline [PBS] and perfusate [4% paraformaldehyde; PFA] in 0.1 M phosphate buffer [PB]). Subsequently, mouse brains were carefully isolated, placed in a PFA solution-filled container, and stored at 4 °C. The following day, brain samples were washed with PBS to remove the excess PFA, placed in a 30% sucrose solution, and stored at 4 °C until use.

Brain samples were sectioned using a cryostat (Leica CM1850; Wetzlar, Germany) adjusted to 40-μm thickness, following stereotaxic coordinates of the mouse brain^64^. The brain slices were placed in a 0.2 M PB:distilled water:ethylene glycol:glycerin (1:3:3:3) storage solution and stored at 4 °C (short-term storage) or −20 °C (long-term storage).

The hippocampal region, specifically the DG, was selected, given its neurogenic role in the adult brain^35^. Brain slices were carefully washed thrice in a 24-well plate filled with 1× PBS. Subsequently, samples were incubated in a protein-blocking solution (5% goat serum and 0.3% Triton™ X-100 in 1× PBS) for 1 h at room temperature. Thereafter, brain slices were incubated (free-floating) with primary antibodies (dilution, 1/250) dissolved in a protein-blocking solution for 3 days. The primary antibodies used were mouse monoclonal anti-BrdU (Thermo Fisher Scientific, Cat# MA3-071, RRID:AB_10986341), mouse monoclonal anti-NEU-N (LSBio, Cat# LS-C312122, RRID:AB_2827517), and rabbit polyclonal anti-GFAP (LSBio; catalog number: LS-B15993). After thrice washing with 1× PBS, the samples were incubated with either goat-anti-rabbit Alexa Fluor™-555 or goat-anti-mouse Alexa Fluor™-488 dyes (1/250) overnight at room temperature. The samples were thrice washed in 1× PBS and then incubated for 10 min with Hoechst 33342 (Thermo Scientific, MA, USA; catalog number:62249) in 1× PBS (1/1000). After three final washes with 1× PBS, the brain slices were mounted on 25 × 75 × 1 mm clean positively charged microscope slides (Walter Products Inc., MI, USA; catalog number: C17090W) and cured with Fisher Chemical™ Permount™ Mounting Medium (Fisher Scientific, NH, USA; catalog number: SP15-100). The prepared slides were then covered with 24 × 50 mm microscope cover glasses (Marienfield Laboratory Glassware, Germany; catalog number:0101222) and observed under a confocal laser-scanning microscope (Leica TCS SP8). The number of cells expressing BrdU in the DG was counted, and corrected cell fluorescence levels for NEU-N and GFAP in the DG were analyzed using ImageJ software (RRID: SCR_003070), as described previously^63,65,66^ (Fig. 6a, b).

### EXPERIMENT 2: Outcomes of non-pharmacological management in ADHD-PI genetic targets and behavior

#### Rearing environment

From week 3 (PND 21) to 7 (PND 49-50), six mice of each strain were maintained in either the standard environment (SE) or EE (n = 12/group) (Fig. 7a, b). The onset and duration of the rearing environment were based on previous studies that reported improved neural and behavioral outcomes in spontaneously hypertensive rats (SHR/NCrl)^67^, the most validated animal model of ADHD. The overall cage size for both SE and EE measurements was 430 × 290 × 201 mm (L × B × H), with corncob bedding and a wire mesh cover. In addition, the EE cage contained a variety of stimulating, well-proportioned objects (i.e., colorful plastic tubes, wooden blocks, glass marbles, and small cylindrical-shaped wire cage platforms) (Fig. 7b) to initiate interest and allow exploratory behavior, affording improvements in memory and cognitive functions^68^. Cages were cleaned twice weekly to ensure a hygienic environment, and at the same time, objects in the EE group were rearranged into a novel configuration to ensure reinterest and re-exploration.

#### Treadmill exercise

The use of treadmill exercise allows an aerobic form of workout in mice, initiated in combination with EE, previously shown to improve memory and cognitive functions in rodents^69^. Mice exposed to EE were allowed to run on a treadmill platform six times weekly (20 min/day) for four weeks in a rearing environment (Fig. 7a, b).

Before the actual treadmill exercise, mice from the EE group were allowed a one-week adaptation period to familiarize themselves with the treadmill (Daejong Lab, Seoul, Republic of Korea)^70^. In this study, we prevented the use of shock to stimulate running, as it may induce stress in mice, eventually altering their behavior. Instead, tapping using the experimenter’s finger(s) or with a small blunt object or tail tickling was used to stimulate mice to continuously walk/run^71^. We maintained treadmill exercise for 20 min every 6 days, while the speed was gradually increased weekly to ensure continuous training and to stimulate running at the following speeds: week 0/habituation (10 m/20 min), week 1 (15 m/20 min), week 2 (20 m/min), week 3 (25 m/min), and week 4 (30 m/min). A sample mouse treadmill experiment is supplemented (Supplementary Video 1). Mice experiencing early signs of fatigue (i.e., remaining still) were allowed to rest and were eventually reintroduced to the platform to continue the remaining treadmill exercise period.

#### Y-maze

Mice (THRSP OE, KO, WT) (*n* = 12) maintained in either SE (6 per cage) or EE (6 per cage) (Fig. 7a, b) were subjected to Y-maze tests at a frequency of four tests conducted on days 7, 14, 21, and 28 of rearing enrichment (Fig. 7c). The experimental protocols used were the same as those employed in “Experiment 1” unless otherwise indicated.

#### Brain extraction

For mice (*n* = 12) exposed to either SE or EE (Fig. 7a, b), brains were extracted and randomly assigned to two sets. The first set of six hippocampal DG samples was divided into left and right sections for subsequent use in qRT-PCR (left) and western blotting (right) (*n* = 6), respectively, whereas the last sets of brain samples were used for immunofluorescence (Figs. 8 and 9) (*n* = 6).

#### RNA extraction and qRT-PCR

The left hippocampal DG isolated from the first set of mice (*n* = 6) exposed to either SE or EE was used for further RNA extraction and subsequent qRT-PCR analysis, using the same protocols as those in “Experiment 1”, unless otherwise indicated. In this experiment, we only measured and analyzed genes that were found to be altered during Experiment 1 or those showing up/downward trends. However, some genes were measured considering representation for each target belonging to ligands, inhibitors, receptors, co-receptors, and multiprotein complexes involved in canonical/noncanonical Wnt signaling (Fig. 8a–h). The gene targets, including *Wnt3*, *Wnt6*, *Wnt7a*, *Cer1*, *Igfbp5*, *Wif1*, *Apcdd1*, *Fzd1*, *Fzd3*, *Lrp5*, *Lrp6*, *Ror1*, *Dvl1*, *Axin2*, *Csnk1ɛ*, *Gsk3β*, and *Ctnnβ1* were normalized to *Actb* as the housekeeping gene (refer to the “EXPERIMENT 1” for the primer information of the above-mentioned target genes).

#### Western blotting

Western blotting was performed on the right hippocampal DG isolated from the first set of mice (*n* = 6) exposed to the SE or EE to examine specific Wnt signaling targets (i.e., p-GSK3B, GSK3B, CSNK1E) (Fig. 8i) and neuronal markers (i.e., NEU-N, GFAP) (Fig. 9b) in the mouse hippocampal DG region using the same protocols as those used in Experiment 1. Original blots are shown in Supplementary Fig. 1.

#### 5-BrdU injection

Mice were injected with 50 mg/kg 5-BrdU for 6 days during the last week of environmental enrichment and treadmill exercises (days 22–27), followed by a 100 mg/kg injection on day 28 after the behavioral experiments, or 3 h prior to brain extraction. This labeling technique targets proliferating cells in response to combined EE and treadmill exercise.

#### Immunofluorescence

Mice were sacrificed immediately after the last behavioral experiment and 5-BrdU injection. The protocols were the same as those used in Experiment 1, primarily targeting BrdU immunoreactivity (Fig. 9a) in the hippocampal DG region.

### Statistics and reproducibility

Statistical analyses were performed using GraphPad Prism v9.4.1 (681) (GraphPad Software, Inc., La Jolla, CA, USA). For graphical purposes, data are presented as mean ± standard error of the mean, and all statistical analyses were conducted on raw data tested for normal (Gaussian) distribution using the D’Agostino–Pearson omnibus. The animal numbers and recorded data points are indicated in all figures. The results were analyzed using either one-way or two-way analysis of variance with or without repeated measures, followed by Tukey’s multiple comparison test. A level of probability of *P* ≤ 0.05 was defined as the threshold for statistical significance. Experiments were replicated at least three times.

### Reporting summary

Further information on research design is available in the Nature Portfolio Reporting Summary linked to this article.

## Supplementary information

Supplementary Information

Description of Additional Supplementary Files

Supplementary Video 1

Reporting Summary

## Data Availability

Datasets generated and/or analyzed during the current study are available from the corresponding author upon reasonable request. Original western blots are available as a supplementary file (Supplementary Fig. 1).
