# Supplementary material for: Hippocampal dentate gyri proteomics reveals Wnt signaling involvement in the behavioral impairment in the THRSP-overexpressing ADHD mouse model
Source: Commun Biol. 2023 Jan 16;6:55. doi: 10.1038/s42003-022-04387-5 (PMC9842619; doi:10.1038/s42003-022-04387-5)
Supplement: Supplementary file 1 — Supplementary Information [file 42003_2022_4387_MOESM1_ESM.pdf]

Hippocampal dentate gyri proteomics reveals Wnt signaling involvement in the behavioral impairment in the THRSP-overexpressing ADHD mouse model

Raly James Perez Custodio [1, 2, 3, ‡], Hee Jin Kim [2, ‡], Jiyeon Kim [4], Darlene Mae Ortiz [2], Mikyung Kim [2, 5], Danilo Buctot [2], Leandro Val Sayson [2], Hyun Jun Lee [2], Bung-Nyun Kim [6] Eugene C. Yi [4, **], and Jae Hoon Cheong [3,*]

[1]*Department of Ergonomics, Leibniz Research Centre for Working Environment and Human Factors - IfADo, Ardeystr. 67, 44139 Dortmund, Germany*

[2]*Uimyung Research Institute for Neuroscience, Department of Pharmacy, Sahmyook University, 815 Hwarangro, Nowon-gu, Seoul 01795, Republic of Korea*

[3]*Institute for New Drug Development, College of Pharmacy, Jeonbuk National University, 567 Baekje-daero, Deokjin-gu, Jeonju-si, Jeollabuk-do 54896, Republic of Korea*

[4]*Department of Molecular Medicine and Biopharmaceutical Sciences, Graduate School of Convergence Science and Technology and College of Medicine, Seoul National University, Seoul 03080, Republic of Korea*

[5]*Department of Chemistry & Life Science, Sahmyook University, 815 Hwarangro, Nowon-gu, Seoul 01795, Republic of Korea*

[6]*Department of Psychiatry and Behavioral Science, College of Medicine, Seoul National University, 101 Daehakro, Jongno-gu, Seoul, 03080, Republic of Korea*

[‡] Equal Contributions

[*]Corresponding author: Jae Hoon Cheong (cheongjh@jbnu.ac.kr)

[**]Co-corresponding author: Eugene C. Yi (euyi@snu.ac.kr)

Supplementary figure 1: Original Western Blots

Figure 5b/Figure 6c

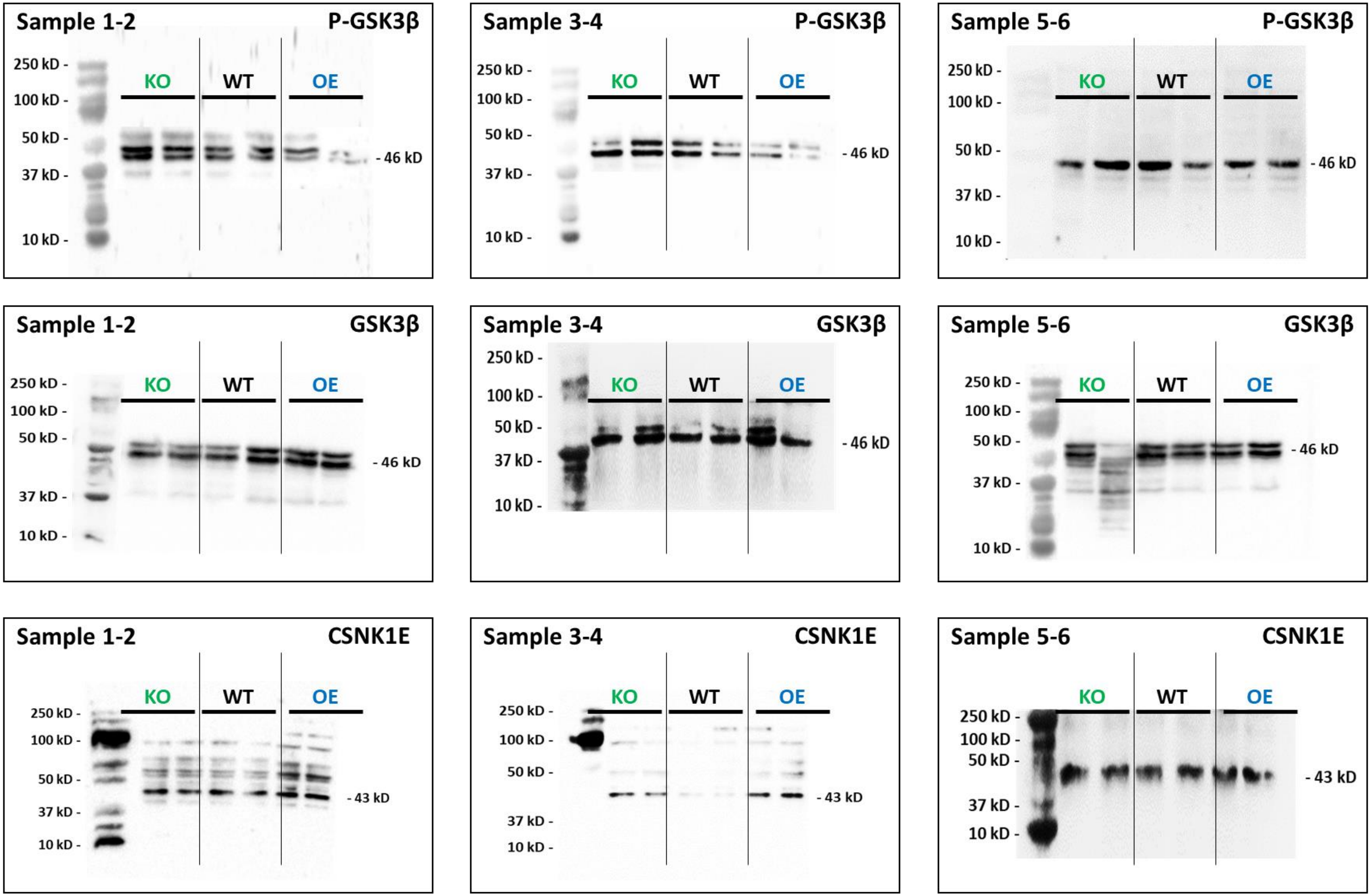

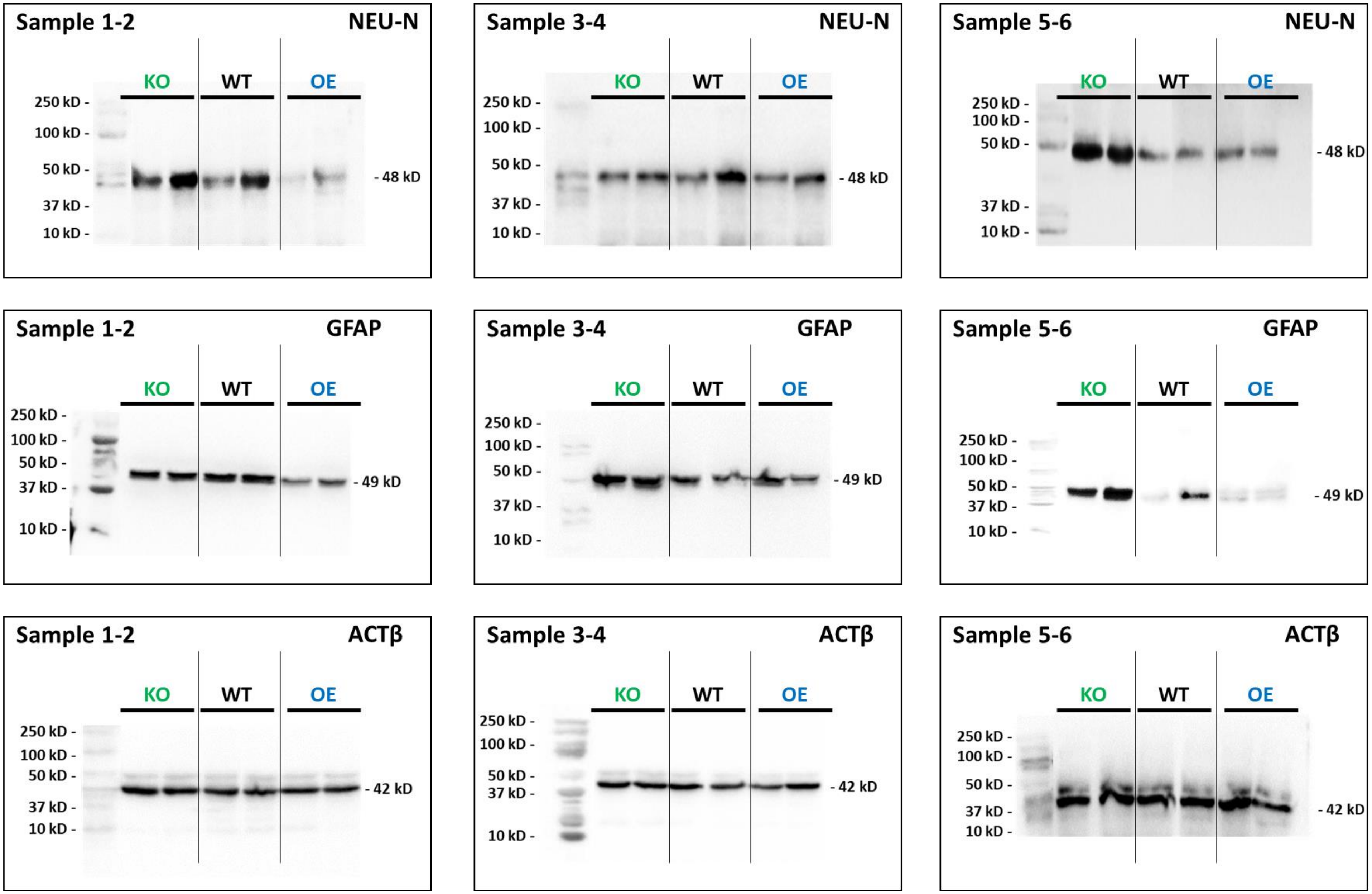
Sample 1-2
NEU-N
KO
WT
OE
250 kD -
100 kD -
50 kD -
37 kD -
10 kD -
- 48 kD
Sample 3-4
NEU-N
KO
WT
OE
250 kD -
100 kD -
50 kD -
37 kD -
10 kD -
- 48 kD
Sample 5-6
NEU-N
KO
WT
OE
250 kD -
100 kD -
50 kD -
37 kD -
10 kD -
- 48 kD
Sample 1-2
GFAP
KO
WT
OE
250 kD -
100 kD -
50 kD -
37 kD -
10 kD -
- 49 kD
Sample 3-4
GFAP
KO
WT
OE
250 kD -
100 kD -
50 kD -
37 kD -
10 kD -
- 49 kD
Sample 5-6
GFAP
KO
WT
OE
250 kD -
100 kD -
50 kD -
37 kD -
10 kD -
- 49 kD
Sample 1-2
ACTβ
KO
WT
OE
250 kD -
100 kD -
50 kD -
37 kD -
10 kD -
- 42 kD
Sample 3-4
ACTβ
KO
WT
OE
250 kD -
100 kD -
50 kD -
37 kD -
10 kD -
- 42 kD
Sample 5-6
ACTβ
KO
WT
OE
250 kD -
100 kD -
50 kD -
37 kD -
10 kD -
- 42 kD

Figure 8i/Figure 9b

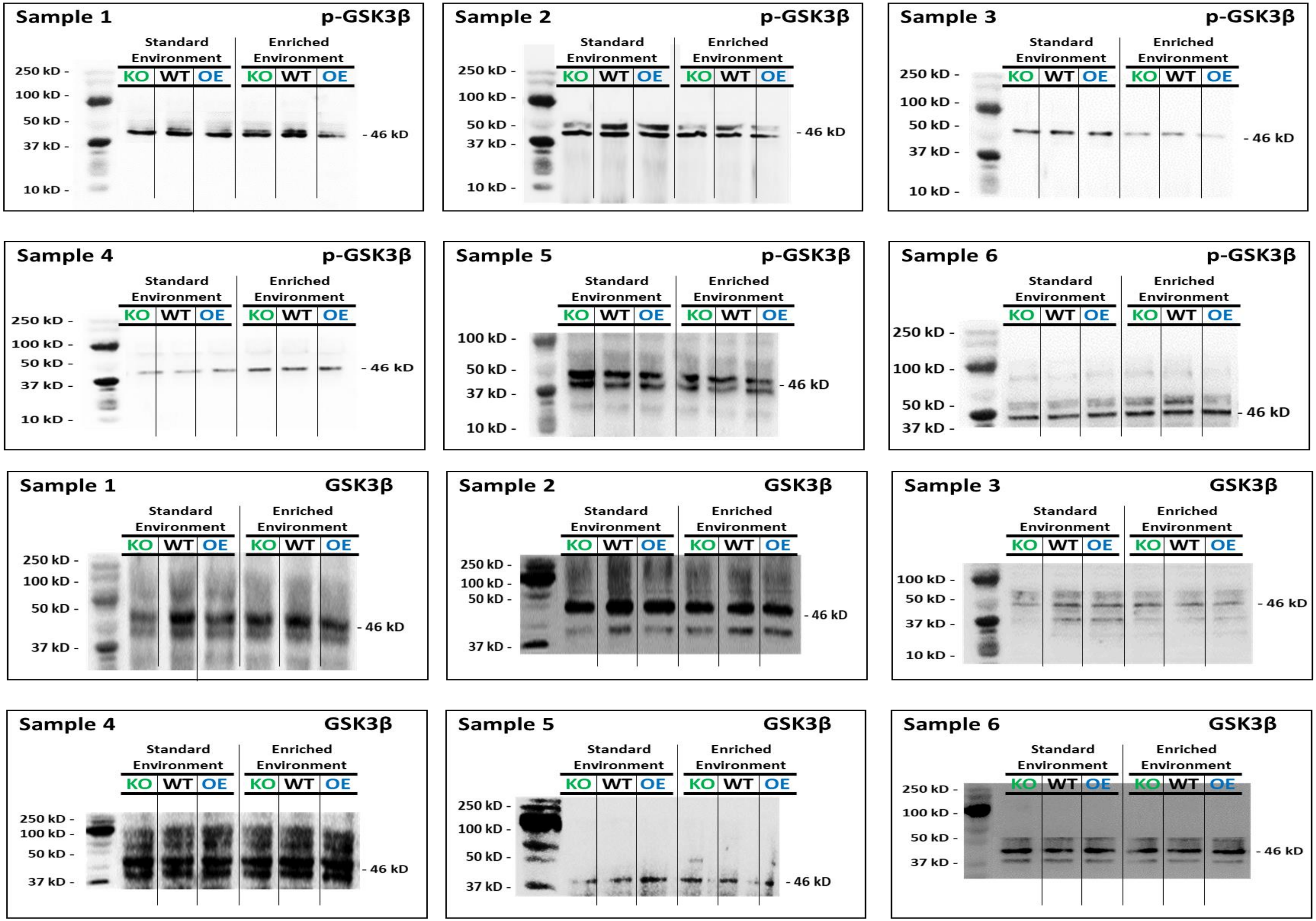

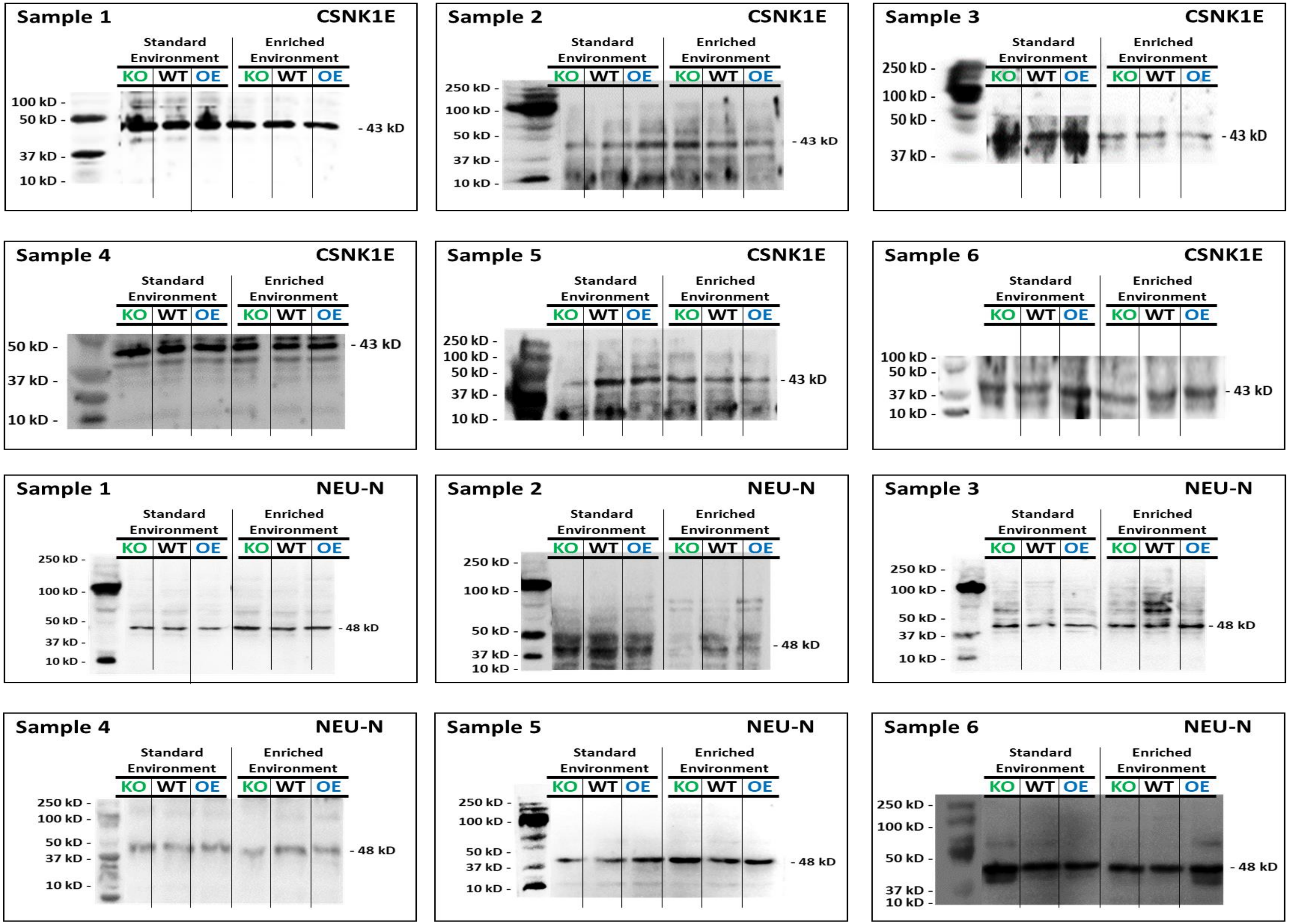
Sample 1
CSNK1E
Standard Environment
Enriched Environment
KO
WT
OE
KO
WT
OE
100 kD -
50 kD -
37 kD -
10 kD -
- 43 kD
Sample 2
CSNK1E
Standard Environment
Enriched Environment
KO
WT
OE
KO
WT
OE
250 kD -
100 kD -
50 kD -
37 kD -
10 kD -
- 43 kD
Sample 3
CSNK1E
Standard Environment
Enriched Environment
KO
WT
OE
KO
WT
OE
250 kD -
100 kD -
50 kD -
37 kD -
- 43 kD
Sample 4
CSNK1E
Standard Environment
Enriched Environment
KO
WT
OE
KO
WT
OE
50 kD -
37 kD -
10 kD -
- 43 kD
Sample 5
CSNK1E
Standard Environment
Enriched Environment
KO
WT
OE
KO
WT
OE
250 kD -
100 kD -
50 kD -
37 kD -
10 kD -
- 43 kD
Sample 6
CSNK1E
Standard Environment
Enriched Environment
KO
WT
OE
KO
WT
OE
100 kD -
50 kD -
37 kD -
10 kD -
- 43 kD
Sample 1
NEU-N
Standard Environment
Enriched Environment
KO
WT
OE
KO
WT
OE
250 kD -
100 kD -
50 kD -
37 kD -
10 kD -
- 48 kD
Sample 2
NEU-N
Standard Environment
Enriched Environment
KO
WT
OE
KO
WT
OE
250 kD -
100 kD -
50 kD -
37 kD -
10 kD -
- 48 kD
Sample 3
NEU-N
Standard Environment
Enriched Environment
KO
WT
OE
KO
WT
OE
250 kD -
100 kD -
50 kD -
37 kD -
10 kD -
- 48 kD
Sample 4
NEU-N
Standard Environment
Enriched Environment
KO
WT
OE
KO
WT
OE
250 kD -
100 kD -
50 kD -
37 kD -
10 kD -
- 48 kD
Sample 5
NEU-N
Standard Environment
Enriched Environment
KO
WT
OE
KO
WT
OE
250 kD -
100 kD -
50 kD -
37 kD -
10 kD -
- 48 kD
Sample 6
NEU-N
Standard Environment
Enriched Environment
KO
WT
OE
KO
WT
OE
250 kD -
100 kD -
50 kD -
37 kD -
10 kD -
- 48 kD

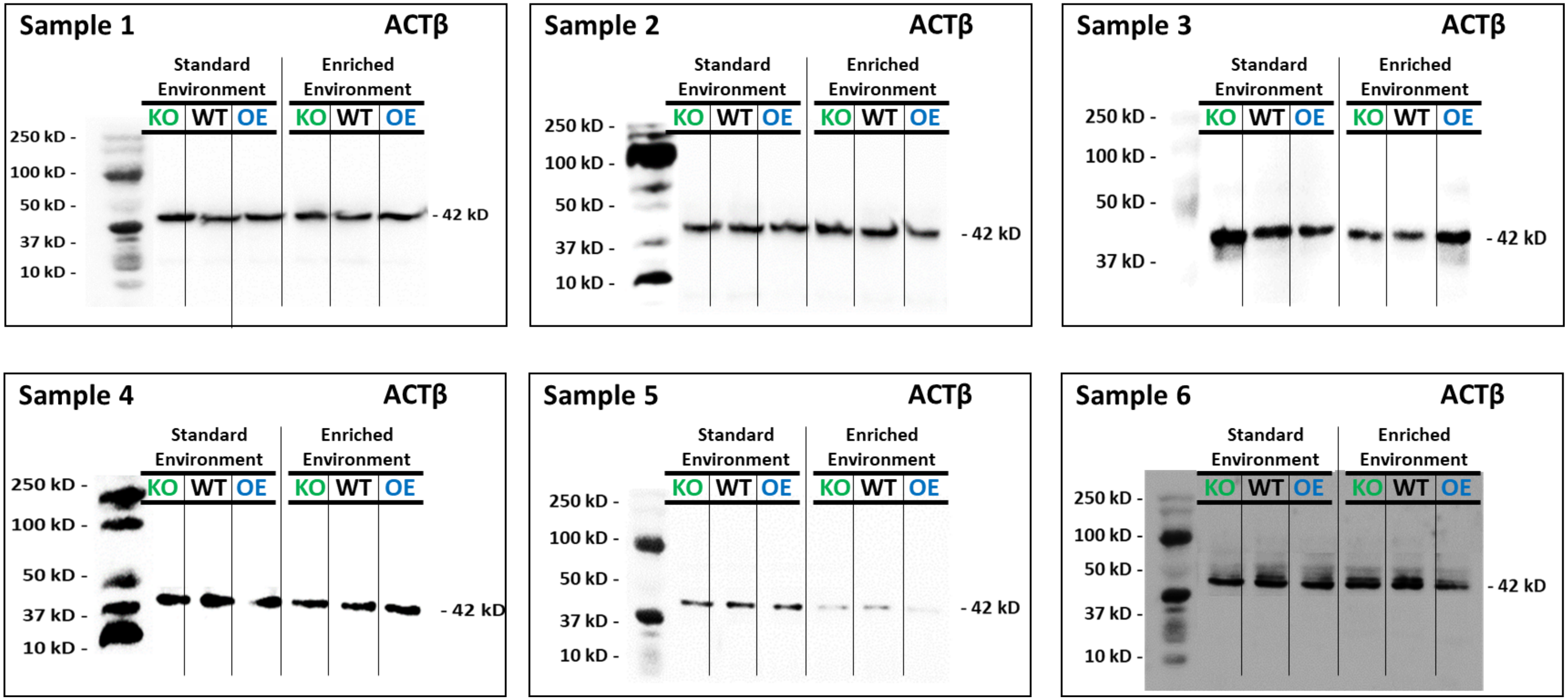
Sample 1
ACTβ
Standard Environment
Enriched Environment
KO WT OE KO WT OE
250 kD -
100 kD -
50 kD -
37 kD -
10 kD -
- 42 kD
Sample 2
ACTβ
Standard Environment
Enriched Environment
KO WT OE KO WT OE
250 kD -
100 kD -
50 kD -
37 kD -
10 kD -
- 42 kD
Sample 3
ACTβ
Standard Environment
Enriched Environment
KO WT OE KO WT OE
250 kD -
100 kD -
50 kD -
37 kD -
- 42 kD
Sample 4
ACTβ
Standard Environment
Enriched Environment
KO WT OE KO WT OE
250 kD -
100 kD -
50 kD -
37 kD -
10 kD -
- 42 kD
Sample 5
ACTβ
Standard Environment
Enriched Environment
KO WT OE KO WT OE
250 kD -
100 kD -
50 kD -
37 kD -
10 kD -
- 42 kD
Sample 6
ACTβ
Standard Environment
Enriched Environment
KO WT OE KO WT OE
250 kD -
100 kD -
50 kD -
37 kD -
10 kD -
- 42 kD

Supplementary Table 1.

Number	Accession	Protein Name	Gene Name	PLGEM-STN	*p* Value
1	O70340	Neuronal pentraxin-2	Nptx2	4.2109	0.0000
2	Q9WV92	Band 4.1-like protein 3	Epb41l3	3.3043	0.0000
3	Q9Z2T6	Keratin type II cuticular Hb5	Krt85	3.2156	0.0000
4	Q6P5D3	Putative ATP-dependent RNA helicase DHX57	Dhx57	3.1513	0.0000
5	Q4FZC9	Nesprin-3	Syne3	3.0656	0.0001
6	Q8C1B7	Septin-11	Septin11	2.8961	0.0001
7	Q8CGI1	Protein FAM193A	Fam193a	2.8821	0.0001
8	O89053	Coronin-1A	Coro1a	2.8326	0.0001
9	P14733	Lamin-B1	Lmnb1	2.8135	0.0001
10	Q7TSJ2	Microtubule-associated protein 6	Map6	2.7530	0.0001
11	Q7TQG1	Pleckstrin homology domain-containing family A member 6	Plekha6	2.6776	0.0001
12	P28665	Murinoglobulin-1	Mug1	2.6599	0.0001
13	Q9QWI6	SRC kinase signaling inhibitor 1	Srcin1	2.6125	0.0001
14	Q811Q9	Choline-phosphate cytidylyltransferase B	Pcyt1b	2.6039	0.0001
15	P49025	Citron Rho-interacting kinase	Cit	2.5870	0.0001
16	Q8CFN5	Myocyte-specific enhancer factor 2C	Mef2c	2.5778	0.0001
17	Q99K48	Non-POU domain-containing octamer-binding protein	Nono	2.5577	0.0002
18	Q8CHS8	Vacuolar protein sorting-associated protein 37A	Vps37a	2.5471	0.0002
19	B1AZI6	THO complex subunit 2	Thoc2	2.5398	0.0002
20	Q61072	Disintegrin and metalloproteinase domain-containing protein 9	Adam9	2.4580	0.0002
21	Q62407	Striated muscle-specific serine/threonine-protein kinase	Speg	2.4520	0.0002
22	Q80TR8	DDB1- and CUL4-associated factor 1	Dcaf1	2.4350	0.0002
23	P50608	Fibromodulin	Fmod	2.4120	0.0002
24	Q80U72	Protein scribble homolog	Scrib	2.3760	0.0002
25	Q80TK0	AP2-interacting clathrin-endocytosis protein	Kiaa1107	2.3626	0.0002
26	Q6VNB8	WD repeat and FYVE domain-containing protein 3	Wdfy3	2.3122	0.0003
27	Q8BRJ3	RELT-like protein 2	Rell2	2.3084	0.0003
28	Q8BUR4	Dedicator of cytokinesis protein 1	Dock1	2.2389	0.0003
29	A2CG49	Kalirin	Kalrn	2.2232	0.0003
30	Q9JI10	Serine/threonine-protein kinase 3	Stk3	2.2205	0.0003
31	Q6QD59	Vesicle transport protein SEC20	Bnip1	2.2194	0.0003
32	Q9ERE2	Keratin type II cuticular Hb1	Krt81	2.2095	0.0003
33	P97861	Keratin type II cuticular Hb6	Krt86	2.2095	0.0003
34	P31750	RAC-alpha serine/threonine-protein kinase	Akt1	2.2082	0.0003
35	Q9WTX6	Cullin-1	Cul1	2.2043	0.0003
36	Q3TVA9	Coiled-coil domain-containing protein 136	Ccdc136	2.1942	0.0003
37	Q63943	Myocyte-specific enhancer factor 2D	Mef2d	2.1848	0.0004
38	Q8CC35	Synaptopodin	Synpo	2.1771	0.0004
39	Q569Z6	Thyroid hormone receptor-associated protein 3	Thrap3	2.1733	0.0004
40	Q80YN3	Breast carcinoma-amplified sequence 1 homolog	Bcas1	2.1689	0.0004
41	Q8BTI8	Serine/arginine repetitive matrix protein 2	Srrm2	2.1629	0.0004
42	D3YVF0	A-kinase anchor protein 5	Akap5	2.1456	0.0004
43	Q5SVL6	Rap1 GTPase-activating protein 2	Rap1gap2	2.1313	0.0005
44	Q8VHK9	ATP-dependent DNA/RNA helicase DHX36	Dhx36	2.1266	0.0005
45	B2RR83	3'-5' RNA helicase YTHDC2	Ythdc2	2.1012	0.0005
46	Q3UJB9	Enhancer of mRNA-decapping protein 4	Edc4	2.0836	0.0005
47	O88569	Heterogeneous nuclear ribonucleoproteins A2/B1	Hnrnpa2b1	2.0726	0.0005
48	Q9JJK2	LanC-like protein 2	Lancl2	2.0418	0.0006
49	Q9D394	Protein RUFY3	Rufy3	2.0391	0.0006
50	P16858	Glyceraldehyde-3-phosphate dehydrogenase	Gapdh	2.0329	0.0006
51	Q8BYW1	Rho GTPase-activating protein 25	Arhgap25	2.0145	0.0006
52	Q5XJV6	Serine/threonine-protein kinase LMTK3	Lmtk3	2.0073	0.0006
53	Q922P8	Transmembrane protein 132A	Tmem132a	2.0065	0.0006
54	Q68EF4	Metabotropic glutamate receptor 4	Grm4	2.0065	0.0006
55	Q8BZZ3	NEDD4-like E3 ubiquitin-protein ligase WWP1	Wwp1	2.0056	0.0006
56	Q62383	Transcription elongation factor SPT6	Supt6h	2.0056	0.0006
57	Q8R146	Acylamino-acid-releasing enzyme	Apeh	1.9919	0.0006
58	Q5DU31	Interactor protein for cytohesin exchange factors 1	Ipcef1	1.9764	0.0006
59	Q9WVR4	Fragile X mental retardation syndrome-related protein 2	Fxr2	1.9711	0.0006
60	Q3TCJ1	BRISC complex subunit Abraxas 2	Abraxas2	1.9647	0.0006
61	Q07409	Contactin-3	Cntn3	1.9647	0.0006
62	Q8VDL4	ADP-dependent glucokinase	Adpgk	1.9510	0.0007

63	P84091	AP-2 complex subunit mu	Ap2m1	1.9462	0.0007
64	E9PZ19	Protein turtle homolog B	Igsf9b	1.9305	0.0007
65	Q8BH60	Golgi-associated PDZ and coiled-coil motif-containing protein	Gopc	1.9298	0.0007
66	Q9Z1K6	E3 ubiquitin-protein ligase ARIH2	Arih2	1.9298	0.0007
67	Q8C525	Protein MB21D2	Mb21d2	1.9298	0.0007
68	Q62413	Ephrin type-A receptor 6	Epha6	1.9138	0.0007
69	P48678	Prelamin-A/C	Lmna	1.8907	0.0008
70	Q80U57	Regulating synaptic membrane exocytosis protein 3	Rims3	1.8804	0.0008
71	Q60634	Flotillin-2	Flot2	1.8722	0.0008
72	Q9CPU4	Microsomal glutathione S-transferase 3	Mgst3	1.8631	0.0008
73	Q8VD12	Zinc finger protein 385A	Znf385a	1.8594	0.0009
74	Q6ZQH8	Nucleoporin NUP188	Nup188	1.8282	0.0009
75	Q9QXT8	Calsenilin	Kcnip3	1.8268	0.0009
76	O35129	Prohibitin-2	Phb2	1.8173	0.0009
77	Q9QYI3	DnaJ homolog subfamily C member 7	Dnajc7	1.8012	0.0010
78	O70400	PDZ and LIM domain protein 1	Pdlim1	1.7940	0.0010
79	Q99M74	Keratin type II cuticular Hb2	Krt82	1.7886	0.0011
80	Q9JKK8	Serine/threonine-protein kinase ATR	Atr	1.7877	0.0011
81	P29387	Guanine nucleotide-binding protein subunit beta-4	Gnb4	1.7868	0.0011
82	Q8BZ98	Dynamin-3	Dnm3	1.7835	0.0011
83	Q9R0A0	Peroxisomal membrane protein PEX14	Pex14	1.7754	0.0011
84	Q9JJV5	Voltage-dependent calcium channel gamma-3 subunit	Cacng3	1.7723	0.0011
85	Q8C3F2	Constitutive coactivator of PPAR-gamma-like protein 2	Fam120c	1.7698	0.0011
86	Q8BLE7	Vesicular glutamate transporter 2	Slc17a6	1.7665	0.0011
87	O08919	Numb-like protein	Numbl	1.7370	0.0012
88	Q6GSS7	Histone H2A type 2-A	Hist2h2aa1	1.7364	0.0012
89	Q64523	Histone H2A type 2-C	H2ac20	1.7364	0.0012
90	Q6QI06	Rapamycin-insensitive companion of mTOR	Rictor	1.7339	0.0012
91	Q8C015	Serine/threonine-protein kinase PAK 5	Pak5	1.7339	0.0012
92	Q64467	Glyceraldehyde-3-phosphate dehydrogenase testis-specific	Gapdhs	1.7330	0.0012
93	P26645	Myristoylated alanine-rich C-kinase substrate	Marcks	1.7311	0.0012
94	Q8K4Q0	Regulatory-associated protein of mTOR	Rptor	1.7259	0.0012
95	Q99P47	Contactin-associated protein-like 4	Cntnap4	1.7244	0.0012
96	Q8K010	5-oxoprolinase	Oplah	1.7237	0.0012
97	Q6P4S6	Serine/threonine-protein kinase SIK3	Sik3	1.7216	0.0012
98	Q9WTS2	Alpha-(1 6)-fucosyltransferase	Fut8	1.7191	0.0012
99	Q8C7D2	Protein cereblon	Crbn	1.7191	0.0012
100	Q61686	Chromobox protein homolog 5	Cbx5	1.7191	0.0012
101	Q9EQS9	Immunoglobulin superfamily DCC subclass member 4	Igdcc4	1.7191	0.0012
102	E9Q4S1	High affinity cAMP-specific and IBMX-insensitive 3' 5'-cyclic phosphodiesterase 8B	Pde8b	1.7191	0.0012
103	O08644	Ephrin type-B receptor 6	Ephb6	1.7163	0.0012
104	Q9WUA6	RAC-gamma serine/threonine-protein kinase	Akt3	1.7025	0.0013
105	P07310	Creatine kinase M-type	Ckm	1.6964	0.0013
106	Q8JZK9	Hydroxymethylglutaryl-CoA synthase cytoplasmic	Hmgcs1	1.6877	0.0013
107	Q99KI0	Aconitate hydratase mitochondrial	Aco2	1.6753	0.0013
108	Q149F3	Eukaryotic peptide chain release factor GTP-binding subunit ERF3B	Gspt2	1.6646	0.0014
109	Q91WM1	Spermatid perinuclear RNA-binding protein	Strbp	1.6570	0.0014
110	P08775	DNA-directed RNA polymerase II subunit RPB1	Polr2a	1.6466	0.0014
111	Q4VAA2	Protein CDV3	Cdv3	1.6421	0.0014
112	Q8K019	Bcl-2-associated transcription factor 1	Bclaf1	1.6353	0.0015
113	Q8BIV3	Ran-binding protein 6	Ranbp6	1.6350	0.0015
114	Q80YE7	Death-associated protein kinase 1	Dapk1	1.6316	0.0015
115	Q9DBS9	Oxysterol-binding protein-related protein 3	Osbpl3	1.6276	0.0015
116	P50096	Inosine-5'-monophosphate dehydrogenase 1	Impdh1	1.6276	0.0015
117	Q5DU25	IQ motif and SEC7 domain-containing protein 2	Iqsec2	1.6259	0.0015
118	Q8BH66	Atlastin-1	Atl1	1.6219	0.0015
119	Q9DBE8	Alpha-1 3/1 6-mannosyltransferase ALG2	Alg2	1.6192	0.0015
120	O55131	Septin-7	Septin7	1.6171	0.0015
121	Q811P8	Rho GTPase-activating protein 32	Arhgap32	1.6138	0.0015
122	Q91V12	Cytosolic acyl coenzyme A thioester hydrolase	Acot7	1.6052	0.0016
123	A2A690	Protein TANC2	Tanc2	1.6045	0.0016
124	Q09M02	Cytosolic carboxypeptidase-like protein 5	Agbl5	1.6040	0.0016
125	Q8CAL5	Glypican-5	Gpc5	1.6009	0.0016
126	Q9D486	C-Maf-inducing protein	Cmip	1.6009	0.0016

127	P12023	Amyloid-beta A4 protein	App	1.6006	0.0016
128	O88811	Signal transducing adapter molecule 2	Stam2	1.6002	0.0016
129	Q9D6I9	Leucine rich adaptor protein 1	Lurap1	1.6002	0.0016
130	Q9CY27	Very-long-chain enoyl-CoA reductase	Tecr	1.5917	0.0016
131	Q6PFQ7	Ras GTPase-activating protein 4	Rasa4	1.5917	0.0016
132	P06745	Glucose-6-phosphate isomerase	Gpi	1.5795	0.0017
133	Q8CHT1	Ephexin-1	Ngef	1.5740	0.0017
134	P22723	Gamma-aminobutyric acid receptor subunit gamma-2	Gabrg2	1.5722	0.0017
135	Q61189	Methylosome subunit pICln	Clns1a	1.5688	0.0017
136	Q5DTM8	E3 ubiquitin-protein ligase BRE1A	Rnf20	1.5600	0.0019
137	Q08890	Iduronate 2-sulfatase	Ids	1.5600	0.0019
138	Q9JI11	Serine/threonine-protein kinase 4	Stk4	1.5586	0.0019
139	P60670	Nuclear protein localization protein 4 homolog	Nploc4	1.5486	0.0019
140	Q8K2B3	Succinate dehydrogenase [ubiquinone] flavoprotein subunit mitochondrial	Sdha	1.5465	0.0019
141	P52479	Ubiquitin carboxyl-terminal hydrolase 10	Usp10	1.5446	0.0019
142	Q9QZB0	Regulator of G-protein signaling 17	Rgs17	1.5446	0.0019
143	Q80YV4	4'-phosphopantetheine phosphatase	Pank4	1.5411	0.0020
144	Q6A0A2	La-related protein 4B	Larp4b	1.5398	0.0020
145	Q6ZWR6	Nesprin-1	Syne1	1.5363	0.0020
146	Q9QZB7	Actin-related protein 10	Actr10	1.5291	0.0021
147	Q8CIN6	CUGBP Elav-like family member 3	Celf3	1.5264	0.0021
148	Q8R526	Polycystic kidney disease protein 1-like 1	Pkd1l1	1.5264	0.0021
149	Q99LI2	Chloride channel CLIC-like protein 1	Clcc1	1.5196	0.0021
150	P18581	Cationic amino acid transporter 2	Slc7a2	1.5196	0.0021
151	P97386	DNA ligase 3	Lig3	1.5196	0.0021
152	P34152	Focal adhesion kinase 1	Ptk2	1.5095	0.0022
153	Q6A065	Centrosomal protein of 170 kDa	Cep170	1.5090	0.0022
154	Q8VEE1	LIM and cysteine-rich domains protein 1	Lmcd1	1.5086	0.0022
155	P48193	Protein 4.1	Epb41	1.5086	0.0022
156	Q9EPX2	Papilin	Papln	1.5052	0.0022
157	Q60598	Src substrate cortactin	Cttn	1.5034	0.0022
158	P36993	Protein phosphatase 1B	Ppm1b	1.5012	0.0022
159	Q80SW1	S-adenosylhomocysteine hydrolase-like protein 1	Ahcyl1	1.5009	0.0022
160	Q99NE5	Regulating synaptic membrane exocytosis protein 1	Rims1	1.4859	0.0022
161	P18572	Basigin	Bsg	1.4844	0.0023
162	Q8R1M2	Histone H2A.J	H2aj	1.4805	0.0023
163	Q80U49	Centrosomal protein of 170 kDa protein B	Cep170b	1.4795	0.0023
164	P60882	Multiple epidermal growth factor-like domains protein 8	Megf8	1.4771	0.0024
165	P97445	Voltage-dependent P/Q-type calcium channel subunit alpha-1A	Cacna1a	1.4660	0.0025
166	E9Q912	Rap1 GTPase-GDP dissociation stimulator 1	Rap1gds1	1.4584	0.0025
167	Q8BJ42	Disks large-associated protein 2	Dlgap2	1.4549	0.0027
168	P54731	FAS-associated factor 1	Faf1	1.4541	0.0027
169	P97490	Adenylate cyclase type 8	Adcy8	1.4534	0.0027
170	Q5SUE8	Ankyrin repeat domain-containing protein 40	Ankrd40	1.4470	0.0028
171	Q3USL1	Kelch domain-containing protein 9	Klhdc9	1.4470	0.0028
172	Q3UQN2	F-BAR domain only protein 2	Fcho2	1.4470	0.0028
173	Q8BHT6	Beta-1 3-glucosyltransferase	B3glct	1.4470	0.0028
174	Q9JLC4	VPS10 domain-containing receptor SorCS1	Sorcs1	1.4470	0.0028
175	Q9Z0W3	Nuclear pore complex protein Nup160	Nup160	1.4470	0.0028
176	B2RY04	Dedicator of cytokinesis protein 5	Dock5	1.4470	0.0028
177	A2A8U2	Transmembrane protein 201	Tmem201	1.4470	0.0028
178	Q9D968	Host cell factor 2	Hcfc2	1.4470	0.0028
179	Q91YT2	E3 ubiquitin-protein ligase RNF185	Rnf185	1.4470	0.0028
180	Q8BL43	Ras association domain-containing protein 10	Rassf10	1.4470	0.0028
181	Q99MD9	Nuclear autoantigenic sperm protein	Nasp	1.4470	0.0028
182	Q9Z2G1	Protein fem-1 homolog A-A	Fem1aa	1.4470	0.0028
183	P62881	Guanine nucleotide-binding protein subunit beta-5	Gnb5	1.4455	0.0028
184	Q80TT8	Cullin-9	Cul9	1.4386	0.0028
185	Q8CGF7	Transcription elongation regulator 1	Tcerg1	1.4334	0.0029
186	O08599	Syntaxin-binding protein 1	Stxbp1	1.4331	0.0029
187	P47738	Aldehyde dehydrogenase mitochondrial	Aldh2	1.4251	0.0029
188	Q9R1Q9	V-type proton ATPase subunit S1	Atp6ap1	1.4209	0.0030
189	P63085	Mitogen-activated protein kinase 1	Mapk1	1.4185	0.0030
190	Q5U4F6	Cytoplasmic dynein 2 intermediate chain 2	Dync2i2	1.4028	0.0031

191	Q8BFW7	Lipoma-preferred partner homolog	Lpp	1.4005	0.0031
192	Q9DBD5	Proline- glutamic acid- and leucine-rich protein 1	Pelp1	1.4005	0.0031
193	Q8R1F1	Protein Niban 2	Niban2	1.4005	0.0031
194	Q99PV0	Pre-mRNA-processing-splicing factor 8	Prpf8	1.3992	0.0031
195	Q8BGW1	Alpha-ketoglutarate-dependent dioxygenase FTO	Fto	1.3990	0.0031
196	Q9JLT4	Thioredoxin reductase 2 mitochondrial	Txnrd2	1.3989	0.0031
197	Q8K0T4	Katanin p60 ATPase-containing subunit A-like 1	Katnal1	1.3986	0.0031
198	O35954	Membrane-associated phosphatidylinositol transfer protein 1	Pitpnm1	1.3976	0.0031
199	Q9D6F4	Gamma-aminobutyric acid receptor subunit alpha-4	Gabra4	1.3943	0.0031
200	Q8BH24	Transmembrane 9 superfamily member 4	Tm9sf4	1.3943	0.0031
201	Q80W47	WD repeat domain phosphoinositide-interacting protein 2	Wipi2	1.3846	0.0032
202	Q8CJH3	Plexin-B1	Plxnb1	1.3834	0.0032
203	Q8C2Q3	RNA-binding protein 14	Rbm14	1.3816	0.0033
204	Q8R4C2	RUN and FYVE domain-containing protein 2	Rufy2	1.3810	0.0033
205	Q9QZS3	Protein numb homolog	Numb	1.3796	0.0033
206	Q9JKY5	Huntingtin-interacting protein 1-related protein	Hip1r	1.3725	0.0033
207	Q99KP6	Pre-mRNA-processing factor 19	Prpf19	1.3710	0.0033
208	Q4VA61	Down syndrome cell adhesion molecule-like protein 1 homolog	Dscaml1	1.3666	0.0034
209	Q5DTL9	Sodium-driven chloride bicarbonate exchanger	Slc4a10	1.3660	0.0034
210	Q64442	Sorbitol dehydrogenase	Sord	1.3624	0.0035
211	Q8BIJ7	RUN and FYVE domain-containing protein 1	Rufy1	1.3554	0.0035
212	A2AQ25	Sickle tail protein	Skt	1.3443	0.0037
213	Q4VBD2	Transmembrane anterior posterior transformation protein 1	Tapt1	1.3412	0.0037
214	Q8C1A5	Thimet oligopeptidase	Thop1	1.3399	0.0038
215	Q91XU3	Phosphatidylinositol 5-phosphate 4-kinase type-2 gamma	Pip4k2c	1.3375	0.0038
216	Q8BG81	Polymerase delta-interacting protein 3	Poldip3	1.3371	0.0038
217	Q64444	Carbonic anhydrase 4	Ca4	1.3365	0.0038
218	E9PUL5	Proline-rich transmembrane protein 2	Prrt2	1.3361	0.0038
219	Q8K4B0	Metastasis-associated protein MTA1	Mta1	1.3354	0.0038
220	Q8C4B4	Protein unc-119 homolog B	Unc119b	1.3295	0.0039
221	P10852	4F2 cell-surface antigen heavy chain	Slc3a2	1.3285	0.0039
222	Q9EQZ7	Regulating synaptic membrane exocytosis protein 2	Rims2	1.3252	0.0039
223	Q6ZQ93	Ubiquitin carboxyl-terminal hydrolase 34	Usp34	1.3243	0.0040
224	Q8CFE4	SCY1-like protein 2	Scyl2	1.3171	0.0040
225	P28063	Proteasome subunit beta type-8	Psmb8	1.3170	0.0040
226	P58158	Galactosylgalactosylxylosylprotein 3-beta-glucuronosyltransferase 3	B3gat3	1.3170	0.0040
227	Q3U1Y4	DENN domain-containing protein 4B	Dennd4b	1.3170	0.0040
228	Q6PDH0	Pleckstrin homology-like domain family B member 1	Phldb1	1.3170	0.0040
229	Q8VDI7	Ubiquitin-associated domain-containing protein 1	Ubac1	1.3170	0.0040
230	Q8BWG8	Beta-arrestin-1	Arrb1	1.3168	0.0040
231	P62874	Guanine nucleotide-binding protein G(I)/G(S)/G(T) subunit beta-1	Gnb1	1.3159	0.0041
232	Q61165	Sodium/hydrogen exchanger 1	Slc9a1	1.3133	0.0041
233	P14148	60S ribosomal protein L7	Rpl7	1.3117	0.0042
234	Q8K2K6	Arf-GAP domain and FG repeat-containing protein 1	Agfg1	1.3095	0.0042
235	A2ALS5	Rap1 GTPase-activating protein 1	Rap1gap	1.3094	0.0042
236	O54865	Guanylate cyclase soluble subunit beta-1	Gucy1b1	1.3045	0.0042
237	Q3V1L4	Cytosolic purine 5'-nucleotidase	Nt5c2	1.3017	0.0042
238	Q8BLQ9	Cell adhesion molecule 2	Cadm2	1.3002	0.0054
239	Q14C51	Pentatricopeptide repeat domain-containing protein 3 mitochondrial	Ptcd3	1.2991	0.0055
240	O88602	Voltage-dependent calcium channel gamma-2 subunit	Cacng2	1.2990	0.0055
241	Q9Z275	Retinaldehyde-binding protein 1	Rlbp1	1.2982	0.0055
242	P51859	Hepatoma-derived growth factor	Hdgf	1.2982	0.0055
243	Q8BHJ5	F-box-like/WD repeat-containing protein TBL1XR1	Tbl1xr1	1.2978	0.0055
244	Q8VDZ4	Palmitoyltransferase ZDHHC5	Zdhhc5	1.2978	0.0055
245	A2RSQ0	DENN domain-containing protein 5B	Dennd5b	1.2978	0.0055
246	Q9D1F4	Proline-rich AKT1 substrate 1	Akt1s1	1.2978	0.0055
247	Q923D5	WW domain-binding protein 11	Wbp11	1.2978	0.0055
248	Q9R0U0	Serine/arginine-rich splicing factor 10	Srsf10	1.2978	0.0055
249	Q5KU39	Vacuolar protein sorting-associated protein 41 homolog	Vps41	1.2967	0.0055
250	Q8CGP0	Histone H2B type 3-B	H2bu1	1.2961	0.0055
251	Q9D2U9	Histone H2B type 3-A	Hist3h2ba	1.2961	0.0055
252	P62880	Guanine nucleotide-binding protein G(I)/G(S)/G(T) subunit beta-2	Gnb2	1.2958	0.0055
253	Q9CZX8	40S ribosomal protein S19	Rps19	1.2944	0.0055
254	A2AHC3	Calmodulin-regulated spectrin-associated protein 1	Camsap1	1.2922	0.0055

255	Q76LS9	Ubiquitin carboxyl-terminal hydrolase MINDY-1	Mindy1	1.2899	0.0056
256	P47857	ATP-dependent 6-phosphofructokinase muscle type	Pfkm	1.2859	0.0056
257	Q9Z2I0	Mitochondrial proton/calcium exchanger protein	Letm1	1.2846	0.0056
258	Q60780	Growth arrest-specific protein 7	Gas7	1.2826	0.0057
259	O54829	Regulator of G-protein signaling 7	Rgs7	1.2815	0.0057
260	B9EJ80	PDZ domain-containing protein 8	Pdzd8	1.2812	0.0057
261	P54285	Voltage-dependent L-type calcium channel subunit beta-3	Cacnb3	1.2785	0.0057
262	Q62172	RalA-binding protein 1	Ralbp1	1.2764	0.0057
263	P07934	Phosphorylase b kinase gamma catalytic chain skeletal muscle/heart isoform	Phkg1	1.2734	0.0058
264	P40124	Adenylyl cyclase-associated protein 1	Cap1	1.2710	0.0058
265	Q02357	Ankyrin-1	Ank1	1.2707	0.0058
266	Q9CZC8	Secernin-1	Scrn1	1.2684	0.0059
267	Q80YF9	Rho GTPase-activating protein 33	Arhgap33	1.2665	0.0060
268	P27659	60S ribosomal protein L3	Rpl3	1.2654	0.0060
269	Q61161	Mitogen-activated protein kinase kinase kinase kinase 2	Map4k2	1.2635	0.0060
270	O70551	SRSF protein kinase 1	Srpk1	1.2635	0.0060
271	Q7TSH4	Centriolar coiled-coil protein of 110 kDa	Ccp110	1.2635	0.0060
272	Q99LH2	Phosphatidylserine synthase 1	Ptdss1	1.2635	0.0060
273	Q80SY6	Adenosine deaminase-like protein	Adal	1.2635	0.0060
274	Q61324	Aryl hydrocarbon receptor nuclear translocator 2	Arnt2	1.2635	0.0060
275	Q6A058	Armadillo repeat-containing X-linked protein 2	Armcx2	1.2635	0.0060
276	Q8K4P0	pre-mRNA 3' end processing protein WDR33	Wdr33	1.2635	0.0060
277	Q8R216	NAD-dependent protein lipoamidase sirtuin-4 mitochondrial	Sirt4	1.2635	0.0060
278	P37242	Thyroid hormone receptor beta	Thrb	1.2635	0.0060
279	Q91VB2	Calcium/calmodulin-dependent protein kinase type 1G	Camk1g	1.2635	0.0060
280	Q3U3E2	Protein FAM117B	Fam117b	1.2635	0.0060
281	Q60603	Potassium voltage-gated channel subfamily H member 1	Kcnh1	1.2635	0.0060
282	Q64735	Complement component receptor 1-like protein	Cr1l	1.2617	0.0060
283	Q80XS7	Protein FAM83E	Fam83e	1.2608	0.0060
284	Q9DCS3	Enoyl-[acyl-carrier-protein] reductase mitochondrial	Mecr	1.2595	0.0061
285	Q9WTX2	Interferon-inducible double-stranded RNA-dependent protein kinase activator A	Prkra	1.2575	0.0062
286	Q8VD65	Phosphoinositide 3-kinase regulatory subunit 4	Pik3r4	1.2541	0.0062
287	Q8CGP5	Histone H2A type 1-F	Hist1h2af	1.2535	0.0062
288	Q8CGP6	Histone H2A type 1-H	H2ac12	1.2535	0.0062
289	C0HKE8	Histone H2A type 1-O	Hist1h2ao	1.2535	0.0062
290	C0HKE4	Histone H2A type 1-E	H2ac8	1.2535	0.0062
291	C0HKE2	Histone H2A type 1-C	H2ac6	1.2535	0.0062
292	C0HKE1	Histone H2A type 1-B	H2ac4	1.2535	0.0062
293	C0HKE9	Histone H2A type 1-P	Hist1h2ap	1.2535	0.0062
294	C0HKE3	Histone H2A type 1-D	H2ac7	1.2535	0.0062
295	Q8BFU2	Histone H2A type 3	H2aw	1.2535	0.0062
296	C0HKE5	Histone H2A type 1-G	H2ac11	1.2535	0.0062
297	C0HKE6	Histone H2A type 1-I	H2ac13	1.2535	0.0062
298	C0HKE7	Histone H2A type 1-N	Hist1h2an	1.2535	0.0062
299	Q8CGP7	Histone H2A type 1-K	H2ac15	1.2535	0.0062
300	Q8BYM8	Probable cysteine--tRNA ligase mitochondrial	Cars2	1.2526	0.0063
301	Q02819	Nucleobindin-1	Nucb1	1.2526	0.0063
302	Q8BVQ5	Protein phosphatase methylesterase 1	Ppme1	1.2526	0.0063
303	Q9QXB9	Developmentally-regulated GTP-binding protein 2	Drg2	1.2461	0.0063
304	A2ADY9	Protein DDI1 homolog 2	Ddi2	1.2440	0.0064
305	Q9JJ43	RNA binding protein fox-1 homolog 1	Rbfox1	1.2440	0.0064
306	Q80UG5	Septin-9	Septin9	1.2401	0.0065
307	Q80TL4	PHD finger protein 24	Phf24	1.2373	0.0065
308	Q8VDP4	Cell cycle and apoptosis regulator protein 2	Ccar2	1.2343	0.0065
309	P53811	Phosphatidylinositol transfer protein beta isoform	Pitpnb	1.2340	0.0065
310	Q8K310	Matrin-3	Matr3	1.2312	0.0066
311	Q64524	Histone H2B type 2-E	H2bc21	1.2269	0.0067
312	Q9EPE9	Endoplasmic reticulum transmembrane helix translocase	Atp13a1	1.2241	0.0067
313	Q8R0A7	Uncharacterized protein KIAA0513	Kiaa0513	1.2234	0.0067
314	Q9R1V6	Disintegrin and metalloproteinase domain-containing protein 22	Adam22	1.2211	0.0068
315	Q3UXZ6	Protein FAM81A	Fam81a	1.2207	0.0068
316	Q8R574	Phosphoribosyl pyrophosphate synthase-associated protein 2	Prpsap2	1.2183	0.0069
317	Q8CGK7	Guanine nucleotide-binding protein G(olf) subunit alpha	Gnal	1.2181	0.0069
318	P15116	Cadherin-2	Cdh2	1.2180	0.0069

319	Q8CBF3	Ephrin type-B receptor 1	Ephb1	1.2175	0.0069
320	Q6ZQ58	La-related protein 1	Larp1	1.2078	0.0072
321	E9Q7G0	Nuclear mitotic apparatus protein 1	Numa1	1.2071	0.0072
322	Q9Z2E1	Methyl-CpG-binding domain protein 2	Mbd2	1.2028	0.0073
323	Q8CGQ8	Sodium/potassium/calcium exchanger 4	Slc24a4	1.2028	0.0073
324	Q9EPW0	Inositol polyphosphate-4-phosphatase type I A	Inpp4a	1.2017	0.0073
325	Q61329	Zinc finger homeobox protein 3	Zfhx3	1.1979	0.0074
326	Q9R0L6	Pericentriolar material 1 protein	Pcm1	1.1953	0.0075
327	Q68FE2	Autophagy-related protein 9A	Atg9a	1.1953	0.0075
328	Q8K004	Spermatogenesis-associated protein 2	Spata2	1.1946	0.0075
329	Q6PEE2	CBP80/20-dependent translation initiation factor	Ctif	1.1946	0.0075
330	Q9D706	RNA polymerase II-associated protein 3	Rpap3	1.1946	0.0075
331	Q3UY34	Protein CUST	Custos	1.1946	0.0075
332	Q8VEG4	Exonuclease 3'-5' domain-containing protein 2	Exd2	1.1946	0.0075
333	Q91YT0	NADH dehydrogenase [ubiquinone] flavoprotein 1 mitochondrial	Ndufv1	1.1932	0.0075
334	P80317	T-complex protein 1 subunit zeta	Cct6a	1.1921	0.0075
335	O55222	Integrin-linked protein kinase	Ilk	1.1910	0.0075
336	P30275	Creatine kinase U-type mitochondrial	Ckmt1	1.1907	0.0076
337	Q9JM76	Actin-related protein 2/3 complex subunit 3	Arpc3	1.1886	0.0076
338	Q7TSH8	Transmembrane protein 94	Tmem94	1.1877	0.0076
339	A2ARP1	Inositol hexakisphosphate and diphosphoinositol-pentakisphosphate kinase 1	Ppip5k1	1.1873	0.0078
340	Q9WUA5	Laforin	Epm2a	1.1873	0.0078
341	Q61137	Astrotactin-1	Astn1	1.1851	0.0079
342	Q3U0V1	Far upstream element-binding protein 2	Khsrp	1.1808	0.0079
343	Q9WUA3	ATP-dependent 6-phosphofructokinase platelet type	Pfkp	1.1760	0.0081
344	Q6P5E4	UDP-glucose:glycoprotein glucosyltransferase 1	Uggt1	1.1667	0.0092
345	O70481	E3 ubiquitin-protein ligase UBR1	Ubr1	1.1659	0.0092
346	Q9WTR1	Transient receptor potential cation channel subfamily V member 2	Trpv2	1.1655	0.0092
347	Q9Z1N5	Spliceosome RNA helicase Ddx39b	Ddx39b	1.1618	0.0093
348	P51660	Peroxisomal multifunctional enzyme type 2	Hsd17b4	1.1618	0.0093
349	P63034	Cytohesin-2	Cyth2	1.1601	0.0094
350	Q9Z2H2	Regulator of G-protein signaling 6	Rgs6	1.1600	0.0094
351	P28650	Adenylosuccinate synthetase isozyme 1	Adss1	1.1574	0.0094
352	Q8BKX6	Serine/threonine-protein kinase SMG1	Smg1	1.1559	0.0094
353	P61620	Protein transport protein Sec61 subunit alpha isoform 1	Sec61a1	1.1559	0.0094
354	Q8BWT5	Disco-interacting protein 2 homolog A	Dip2a	1.1556	0.0095
355	Q69Z98	Serine/threonine-protein kinase BRSK2	Brsk2	1.1552	0.0095
356	Q9D7N9	Adipocyte plasma membrane-associated protein	Apmap	1.1545	0.0095
357	Q6PHS6	Sorting nexin-13	Snx13	1.1535	0.0095
358	Q9D773	39S ribosomal protein L2 mitochondrial	Mrpl2	1.1502	0.0096
359	Q91VL8	Telomeric repeat-binding factor 2-interacting protein 1	Terf2ip	1.1486	0.0096
360	Q8VIJ8	GATOR complex protein NPRL3	Nprl3	1.1486	0.0096
361	Q62203	Splicing factor 3A subunit 2	Sf3a2	1.1486	0.0096
362	P55144	Tyrosine-protein kinase receptor TYRO3	Tyro3	1.1486	0.0096
363	Q6IEE6	Transmembrane protein 132E	Tmem132e	1.1486	0.0096
364	Q99PI5	Phosphatidate phosphatase LPIN2	Lpin2	1.1486	0.0096
365	Q8BKE9	Intraflagellar transport protein 74 homolog	Ift74	1.1486	0.0096
366	Q6PAL7	AT-hook DNA-binding motif-containing protein 1	Ahdc1	1.1486	0.0096
367	Q9CXG3	Peptidyl-prolyl cis-trans isomerase-like 4	Ppil4	1.1486	0.0096
368	Q8VEM1	E3 ubiquitin-protein ligase RNF130	Rnf130	1.1486	0.0096
369	D3Z4I3	RNA-binding protein 24	Rbm24	1.1486	0.0096
370	Q62176	RNA-binding protein 38	Rbm38	1.1486	0.0096
371	Q8VDG3	Poly(A)-specific ribonuclease PARN	Parn	1.1486	0.0096
372	Q6PDX6	E3 ubiquitin-protein ligase Rnf220	Rnf220	1.1486	0.0096
373	Q9JIL5	Tubby-related protein 4	Tulp4	1.1486	0.0096
374	A2AQ19	RNA polymerase-associated protein RTF1 homolog	Rtf1	1.1486	0.0096
375	O70401	Tetraspanin-6	Tspan6	1.1486	0.0096
376	Q4VC33	E3 ubiquitin-protein transferase MAEA	Maea	1.1486	0.0096
377	Q60949	TBC1 domain family member 1	Tbc1d1	1.1486	0.0096
378	Q9DAX9	Amyloid protein-binding protein 2	Appbp2	1.1486	0.0096
379	Q9JIB4	General transcription factor IIH subunit 2	Gtf2h2	1.1486	0.0096
380	Q9R171	Cerebellin-1	Cbln1	1.1486	0.0096
381	Q8BGU2	Cerebellin-2	Cbln2	1.1486	0.0096
382	Q80U38	Protein KHNYN	Khnyn	1.1486	0.0096

383	P97799	Neurensin-1	Nrsn1	1.1486	0.0096
384	O70281	Protein-tyrosine sulfotransferase 1	Tpst1	1.1486	0.0096
385	Q8BZ81	Leucine-rich repeat transmembrane neuronal protein 3	Lrrtm3	1.1486	0.0096
386	P35822	Receptor-type tyrosine-protein phosphatase kappa	Ptprk	1.1486	0.0096
387	Q9Z160	Conserved oligomeric Golgi complex subunit 1	Cog1	1.1486	0.0096
388	Q8VI93	2'-5'-oligoadenylate synthase 3	Oas3	1.1486	0.0096
389	P59644	Phosphatidylinositol 4 5-bisphosphate 5-phosphatase A	Inpp5j	1.1470	0.0097
390	Q9D554	Splicing factor 3A subunit 3	Sf3a3	1.1423	0.0098
391	Q3U2P1	Protein transport protein Sec24A	Sec24a	1.1412	0.0099
392	Q8R4U6	DNA topoisomerase I mitochondrial	Top1mt	1.1412	0.0099
393	O08842	GDNF family receptor alpha-2	Gfra2	1.1408	0.0099
394	Q00560	Interleukin-6 receptor subunit beta	Il6st	1.1408	0.0099
395	P70195	Proteasome subunit beta type-7	Psmb7	1.1408	0.0099
396	P22315	Ferrochelatase mitochondrial	Fech	1.1402	0.0099
397	Q3UHC7	Disabled homolog 2-interacting protein	Dab2ip	1.1402	0.0099
398	P62743	AP-2 complex subunit sigma	Ap2s1	1.1391	0.0100
399	Q6PDG5	SWI/SNF complex subunit SMARCC2	Smarcc2	1.1382	0.0100
400	P40142	Transketolase	Tkt	1.1379	0.0100
401	Q9Z127	Large neutral amino acids transporter small subunit 1	Slc7a5	1.1364	0.0100
402	P36552	Oxygen-dependent coproporphyrinogen-III oxidase mitochondrial	Cpox	1.1301	0.0102
403	Q99KJ8	Dynactin subunit 2	Dctn2	1.1262	0.0102
404	O70585	Dystrobrevin beta	Dtnb	1.1217	0.0106
405	Q9CYI4	Putative RNA-binding protein Luc7-like 1	Luc7l	1.1185	0.0108
406	Q9EQ20	Methylmalonate-semialdehyde dehydrogenase [acylating] mitochondrial	Aldh6a1	1.1184	0.0108
407	Q9EQH2	Endoplasmic reticulum aminopeptidase 1	Erap1	1.1153	0.0109
408	Q8R3H7	Heparan sulfate 2-O-sulfotransferase 1	Hs2st1	1.1153	0.0109
409	Q8K296	Myotubularin-related protein 3	Mtmr3	1.1153	0.0109
410	Q62132	Receptor-type tyrosine-protein phosphatase R	Ptprr	1.1153	0.0109
411	Q9R008	Mevalonate kinase	Mvk	1.1151	0.0109
412	Q9ERB0	Synaptosomal-associated protein 29	Snap29	1.1151	0.0109
413	Q3U186	Probable arginine--tRNA ligase mitochondrial	Rars2	1.1151	0.0109
414	Q99PJ0	Neurotrimin	Ntm	1.1133	0.0109
415	Q3TDK6	Protein rogdi homolog	Rogdi	1.1133	0.0109
416	Q64487	Receptor-type tyrosine-protein phosphatase delta	Ptprd	1.1123	0.0109
417	Q61655	ATP-dependent RNA helicase DDX19A	Ddx19a	1.1117	0.0110
418	Q99MR3	Solute carrier family 12 member 9	Slc12a9	1.1117	0.0110
419	Q9D5V6	Synapse-associated protein 1	Syap1	1.1116	0.0110
420	P41234	ATP-binding cassette sub-family A member 2	Abca2	1.1099	0.0111
421	Q9ET54	Palladin	Palld	1.1091	0.0111
422	P01831	Thy-1 membrane glycoprotein	Thy1	1.1081	0.0112
423	Q04736	Tyrosine-protein kinase Yes	Yes1	1.1077	0.0112
424	Q91YM2	Rho GTPase-activating protein 35	Arhgap35	1.1030	0.0113
425	Q8R2Y0	Monoacylglycerol lipase ABHD6	Abhd6	1.0986	0.0128
426	Q9JHW9	Aldehyde dehydrogenase family 1 member A3	Aldh1a3	1.0975	0.0129
427	Q2TPA8	Hydroxysteroid dehydrogenase-like protein 2	Hsdl2	1.0963	0.0130
428	O88829	Lactosylceramide alpha-2 3-sialyltransferase	St3gal5	1.0941	0.0131
429	Q9D6W8	BLOC-1-related complex subunit 6	Borcs6	1.0941	0.0131
430	Q6PDL0	Cytoplasmic dynein 1 light intermediate chain 2	Dync1li2	1.0938	0.0131
431	Q9QYB1	Chloride intracellular channel protein 4	Clic4	1.0907	0.0132
432	P07903	DNA excision repair protein ERCC-1	Ercc1	1.0853	0.0133
433	Q6DID3	SR-related and CTD-associated factor 8	Scaf8	1.0853	0.0133
434	Q8BSK8	Ribosomal protein S6 kinase beta-1	Rps6kb1	1.0831	0.0134
435	P70677	Caspase-3	Casp3	1.0802	0.0135
436	Q9QYG0	Protein NDRG2	Ndrg2	1.0761	0.0136
437	Q01147	Cyclic AMP-responsive element-binding protein 1	Creb1	1.0755	0.0136
438	Q5EBH1	Ras association domain-containing protein 5	Rassf5	1.0755	0.0136
439	Q0KK59	Protein unc-79 homolog	Unc79	1.0755	0.0136
440	Q3TBW2	39S ribosomal protein L10 mitochondrial	Mrpl10	1.0755	0.0136
441	Q9CWS0	N(G) N(G)-dimethylarginine dimethylaminohydrolase 1	Ddah1	1.0750	0.0136
442	Q68SA9	A disintegrin and metalloproteinase with thrombospondin motifs 7	Adamts7	1.0744	0.0137
443	O55091	Protein IMPACT	Impact	1.0739	0.0137
444	Q6V4S5	Protein sidekick-2	Sdk2	1.0732	0.0137
445	Q91YM4	FAST kinase domain-containing protein 4	Tbrg4	1.0731	0.0137
446	Q9CXR1	Dehydrogenase/reductase SDR family member 7	Dhrs7	1.0731	0.0137

447	Q8R3Z5	Voltage-dependent L-type calcium channel subunit beta-1	Cacnb1	1.0699	0.0138
448	O54833	Casein kinase II subunit alpha'	Csnk2a2	1.0677	0.0139
449	Q8BM13	Noelin-2	Olfm2	1.0675	0.0139
450	Q8R2V3	Zinc finger protein 445	Znf445	1.0675	0.0139
451	Q68FL4	Putative adenosylhomocysteinase 3	Ahcyl2	1.0659	0.0140
452	Q9D0L8	mRNA cap guanine-N7 methyltransferase	Rnmt	1.0651	0.0140
453	O35226	26S proteasome non-ATPase regulatory subunit 4	Psmd4	1.0603	0.0143
454	Q9WTM5	RuvB-like 2	Ruvbl2	1.0591	0.0144
455	Q91ZX7	Prolow-density lipoprotein receptor-related protein 1	Lrp1	1.0579	0.0144
456	Q6PE01	U5 small nuclear ribonucleoprotein 40 kDa protein	Snrnp40	1.0563	0.0144
457	Q0P5W1	Vacuolar protein sorting-associated protein 8 homolog	Vps8	1.0557	0.0145
458	Q61194	Phosphatidylinositol 4-phosphate 3-kinase C2 domain-containing subunit alpha	Pik3c2a	1.0556	0.0145
459	O35075	Vacuolar protein sorting-associated protein 26C	Vps26c	1.0556	0.0145
460	Q9CXY9	GPI-anchor transamidase	Pigk	1.0556	0.0145
461	Q3UHD3	Microtubule-associated tumor suppressor candidate 2 homolog	Mtus2	1.0556	0.0145
462	O70174	Neuronal acetylcholine receptor subunit alpha-4	Chrna4	1.0556	0.0145
463	Q9D3D0	Alpha-tocopherol transfer protein-like	Ttpal	1.0556	0.0145
464	Q810C1	SLIT and NTRK-like protein 1	Slitrk1	1.0556	0.0145
465	Q8K0S5	Reticulon-4 receptor-like 1	Rtn4rl1	1.0556	0.0145
466	Q640M6	Glycerophosphodiester phosphodiesterase domain-containing protein 5	Gdpd5	1.0556	0.0145
467	Q9JIK5	Nucleolar RNA helicase 2	Ddx21	1.0556	0.0145
468	Q91Y63	Solute carrier family 13 member 3	Slc13a3	1.0556	0.0145
469	O54836	Zinc finger matrin-type protein 3	Zmat3	1.0556	0.0145
470	Q8BG30	Negative elongation factor A	Nelfa	1.0556	0.0145
471	Q6ZQA6	Immunoglobulin superfamily member 3	Igsf3	1.0556	0.0145
472	Q8VCQ3	Nuclear receptor-binding factor 2	Nrbf2	1.0556	0.0145
473	Q922K7	Probable 28S rRNA (cytosine-C(5))-methyltransferase	Nop2	1.0556	0.0145
474	Q8CFE2	Histone PARylation factor 1	Hpf1	1.0556	0.0145
475	Q91VW5	Golgin subfamily A member 4	Golga4	1.0556	0.0145
476	Q3UGF1	WD repeat-containing protein 19	Wdr19	1.0556	0.0145
477	Q3UU35	Ovostatin homolog	Ovos	1.0556	0.0145
478	Q9EQB9	Zinc finger protein 287	Znf287	1.0556	0.0145
479	P08074	Carbonyl reductase [NADPH] 2	Cbr2	1.0556	0.0145
480	P22933	Gamma-aminobutyric acid receptor subunit delta	Gabrd	1.0556	0.0145
481	Q9QY40	Plexin-B3	Plxnb3	1.0549	0.0145
482	Q9EPR4	Solute carrier family 23 member 2	Slc23a2	1.0538	0.0146
483	Q6A009	E3 ubiquitin-protein ligase listerin	Ltn1	1.0488	0.0151
484	Q3UHD6	Sorting nexin-27	Snx27	1.0484	0.0151
485	Q1HFZ0	RNA cytosine C(5)-methyltransferase NSUN2	Nsun2	1.0480	0.0152
486	Q64511	DNA topoisomerase 2-beta	Top2b	1.0478	0.0152
487	P97789	5'-3' exoribonuclease 1	Xrn1	1.0477	0.0152
488	P21956	Lactadherin	Mfge8	1.0425	0.0154
489	Q8C4Y3	Negative elongation factor B	Nelfb	1.0425	0.0154
490	Q61466	SWI/SNF-related matrix-associated actin-dependent regulator of chromatin subfamily D member 1	Smarcd1	1.0421	0.0154
491	Q8CB44	GRAM domain-containing protein 4	Gramd4	1.0421	0.0154
492	Q8BYL4	Tyrosine--tRNA ligase mitochondrial	Yars2	1.0421	0.0154
493	Q9CQT1	Methylthioribose-1-phosphate isomerase	Mri1	1.0372	0.0158
494	Q3TWN3	Metal transporter CNNM2	Cnnm2	1.0372	0.0158
495	Q9Z108	Double-stranded RNA-binding protein Staufen homolog 1	Stau1	1.0372	0.0158
496	Q9ESJ4	NCK-interacting protein with SH3 domain	Nckipsd	1.0368	0.0158
497	O35926	Cyclin-dependent kinase 5 activator 2	Cdk5r2	1.0362	0.0158
498	Q6P9K8	Caskin-1	Caskin1	1.0355	0.0158
499	Q62077	1-phosphatidylinositol 4 5-bisphosphate phosphodiesterase gamma-1	Plcg1	1.0348	0.0158
500	P48318	Glutamate decarboxylase 1	Gad1	1.0348	0.0158
501	Q63912	Oligodendrocyte-myelin glycoprotein	Omg	1.0343	0.0159
502	Q60575	Kinesin-like protein KIF1B	Kif1b	1.0320	0.0159
503	P50580	Proliferation-associated protein 2G4	Pa2g4	1.0304	0.0160
504	Q9D8X2	Coiled-coil domain-containing protein 124	Ccdc124	1.0252	0.0163
505	Q8JZS0	Protein lin-7 homolog A	Lin7a	1.0246	0.0163
506	Q9JJC6	RILP-like protein 1	Rilpl1	1.0238	0.0163
507	P54822	Adenylosuccinate lyase	Adsl	1.0232	0.0166
508	Q9CT10	Ran-binding protein 3	Ranbp3	1.0205	0.0167
509	Q99LC3	NADH dehydrogenase [ubiquinone] 1 alpha subcomplex subunit 10 mitochondrial	Ndufa10	1.0196	0.0167
510	Q8BLY3	Leucine-rich repeat and fibronectin type-III domain-containing protein 3	Lrfn3	1.0118	0.0171

511	Q9QZE7	Translin-associated protein X	Tsnax	1.0074	0.0173
512	Q9CXW4	60S ribosomal protein L11	Rpl11	1.0074	0.0173
513	Q99LB2	Dehydrogenase/reductase SDR family member 4	Dhrs4	1.0053	0.0175
514	Q99J77	Sialic acid synthase	Nans	1.0041	0.0176
515	Q9Z0S1	3'(2') 5'-bisphosphate nucleotidase 1	Bpnt1	1.0038	0.0177
516	P38060	Hydroxymethylglutaryl-CoA lyase mitochondrial	Hmgcl	1.0021	0.0177
517	Q9ER73	Elongator complex protein 4	Elp4	0.9997	0.0178
518	Q9WV02	RNA-binding motif protein X chromosome	Rbmx	0.9982	0.0178
519	Q8VE33	Ganglioside-induced differentiation-associated protein 1-like 1	Gdap1l1	0.9975	0.0179
520	Q9CQ10	Charged multivesicular body protein 3	Chmp3	0.9964	0.0179
521	Q9D6Z1	Nucleolar protein 56	Nop56	0.9963	0.0179
522	Q5U458	DnaJ homolog subfamily C member 11	Dnajc11	0.9958	0.0179
523	Q8BH95	Enoyl-CoA hydratase mitochondrial	Echs1	0.9949	0.0180
524	Q8BGN3	Glycerophosphocholine cholinephosphodiesterase ENPP6	Enpp6	0.9944	0.0180
525	Q9CY64	Biliverdin reductase A	Blvra	0.9943	0.0180
526	P50149	Guanine nucleotide-binding protein G(t) subunit alpha-2	Gnat2	0.9939	0.0180
527	P18872	Guanine nucleotide-binding protein G(o) subunit alpha	Gnao1	0.9924	0.0181
528	Q04447	Creatine kinase B-type	Ckb	0.9924	0.0181
529	Q66JT5	Tubulin polyglutamylase complex subunit 2	Tpgs2	0.9920	0.0182
530	P23242	Gap junction alpha-1 protein	Gja1	0.9887	0.0183
531	Q8BZ05	Arf-GAP with Rho-GAP domain ANK repeat and PH domain-containing protein 2	Arap2	0.9868	0.0185
532	Q9ET80	Junctophilin-1	Jph1	0.9868	0.0185
533	Q80XD1	Beta-chimaerin	Chn2	0.9868	0.0185
534	Q9D8P4	39S ribosomal protein L17 mitochondrial	Mrpl17	0.9868	0.0185
535	A2A432	Cullin-4B	Cul4b	0.9861	0.0186
536	Q9CSU0	Regulation of nuclear pre-mRNA domain-containing protein 1B	Rprd1b	0.9815	0.0188
537	P68181	cAMP-dependent protein kinase catalytic subunit beta	Prkacb	0.9802	0.0188
538	Q8CCS6	Polyadenylate-binding protein 2	Pabpn1	0.9789	0.0189
539	Q99JW2	Aminoacylase-1	Acy1	0.9767	0.0190
540	Q6NS60	F-box only protein 41	Fbxo41	0.9757	0.0191
541	Q505F5	Leucine-rich repeat-containing protein 47	Lrrc47	0.9740	0.0191
542	Q810J8	Zinc finger FYVE domain-containing protein 1	Zfyve1	0.9734	0.0192
543	Q9QX11	Cytohesin-1	Cyth1	0.9716	0.0210
544	Q8CH77	Neuron navigator 1	Nav1	0.9709	0.0210
545	Q9WTS5	Teneurin-2	Tenm2	0.9699	0.0211
546	Q9CRC8	Leucine-rich repeat-containing protein 40	Lrrc40	0.9664	0.0216
547	A2AQP0	Myosin-7B	Myh7b	0.9661	0.0216
548	Q8BHA3	D-aminoacyl-tRNA deacylase 2	Dtd2	0.9655	0.0217
549	Q9DAW6	U4/U6 small nuclear ribonucleoprotein Prp4	Prpf4	0.9648	0.0217
550	Q3TDQ1	Dolichyl-diphosphooligosaccharide--protein glycosyltransferase subunit STT3B	Stt3b	0.9638	0.0219
551	P46097	Synaptotagmin-2	Syt2	0.9636	0.0219
552	Q9CS84	Neurexin-1	Nrxn1	0.9630	0.0220
553	Q8BQZ4	Ral GTPase-activating protein subunit beta	Ralgapb	0.9622	0.0221
554	P10637	Microtubule-associated protein tau	Mapt	0.9595	0.0223
555	Q9CW03	Structural maintenance of chromosomes protein 3	Smc3	0.9588	0.0223
556	Q8K448	Cholesterol transporter ABCA5	Abca5	0.9587	0.0223
557	Q7TT37	Elongator complex protein 1	Elp1	0.9567	0.0224
558	P49443	Protein phosphatase 1A	Ppm1a	0.9566	0.0224
559	P21550	Beta-enolase	Eno3	0.9553	0.0225
560	Q8BUV3	Gephyrin	Gphn	0.9527	0.0227
561	P55194	SH3 domain-binding protein 1	Sh3bp1	0.9487	0.0235
562	Q8R550	SH3 domain-containing kinase-binding protein 1	Sh3kbp1	0.9476	0.0236
563	Q99KN9	Clathrin interactor 1	Clint1	0.9454	0.0237
564	P39688	Tyrosine-protein kinase Fyn	Fyn	0.9443	0.0237
565	Q3V132	ADP/ATP translocase 4	Slc25a31	0.9433	0.0238
566	Q8C561	G-protein coupled receptor-associated protein LMBRD2	Lmbrd2	0.9431	0.0238
567	B2RQL2	Storkhead-box protein 1	Stox1	0.9431	0.0238
568	Q570Y9	DEP domain-containing mTOR-interacting protein	Deptor	0.9423	0.0238
569	O88712	C-terminal-binding protein 1	Ctbp1	0.9419	0.0238
570	Q8VEK3	Heterogeneous nuclear ribonucleoprotein U	Hnrnpu	0.9399	0.0239
571	Q3UHE1	Membrane-associated phosphatidylinositol transfer protein 3	Pitpnm3	0.9394	0.0240
572	Q8VCN9	Tubulin-specific chaperone C	Tbcc	0.9394	0.0240
573	Q64096	Guanine nucleotide exchange factor DBS	Mcf2l	0.9365	0.0243
574	Q9D1D4	Transmembrane emp24 domain-containing protein 10	Tmed10	0.9360	0.0244

575	Q5DTT2	PH and SEC7 domain-containing protein 1	Psd	0.9345	0.0245
576	E9Q5F9	Histone-lysine N-methyltransferase SETD2	Setd2	0.9327	0.0245
577	Q8R3Q2	Serine/threonine-protein phosphatase 6 regulatory subunit 2	Ppp6r2	0.9316	0.0246
578	P46096	Synaptotagmin-1	Syt1	0.9313	0.0247
579	Q91WD5	NADH dehydrogenase [ubiquinone] iron-sulfur protein 2 mitochondrial	Ndufs2	0.9311	0.0247
580	Q6RT24	Centromere-associated protein E	Cenpe	0.9304	0.0247
581	Q61481	Calcium/calmodulin-dependent 3' 5'-cyclic nucleotide phosphodiesterase 1A	Pde1a	0.9282	0.0249
582	P26039	Talin-1	Tln1	0.9280	0.0250
583	Q810B6	Rabankyrin-5	Ankfy1	0.9271	0.0250
584	Q9QYI5	DnaJ homolog subfamily B member 2	Dnajb2	0.9267	0.0251
585	Q99LR1	Lysophosphatidylserine lipase ABHD12	Abhd12	0.9267	0.0251
586	P05480	Neuronal proto-oncogene tyrosine-protein kinase Src	Src	0.9254	0.0252
587	Q64514	Tripeptidyl-peptidase 2	Tpp2	0.9242	0.0253
588	Q64133	Amine oxidase [flavin-containing] A	Maoa	0.9240	0.0253
589	Q9CZP5	Mitochondrial chaperone BCS1	Bcs1l	0.9230	0.0253
590	Q80TN4	DnaJ homolog subfamily C member 16	Dnajc16	0.9227	0.0253
591	Q8BY87	Ubiquitin carboxyl-terminal hydrolase 47	Usp47	0.9220	0.0254
592	Q8C167	Prolyl endopeptidase-like	Prepl	0.9219	0.0254
593	Q9R0N0	Galactokinase	Galk1	0.9219	0.0254
594	P16675	Lysosomal protective protein	Ctsa	0.9203	0.0256
595	P20612	Guanine nucleotide-binding protein G(t) subunit alpha-1	Gnat1	0.9184	0.0257
596	Q6A0A9	Constitutive coactivator of PPAR-gamma-like protein 1	FAM120A	0.9183	0.0257
597	P51174	Long-chain specific acyl-CoA dehydrogenase mitochondrial	Acadl	0.9178	0.0257
598	Q6NZL0	Protein SOGA3	Soga3	0.9158	0.0259
599	Q6P9R2	Serine/threonine-protein kinase R1	Oxsr1	0.9153	0.0259
600	Q8R555	Cartilage acidic protein 1	Crtac1	0.9150	0.0259
601	Q99NH0	Ankyrin repeat domain-containing protein 17	Ankrd17	0.9115	0.0261
602	Q8BIG7	Catechol O-methyltransferase domain-containing protein 1	Comtd1	0.9115	0.0261
603	Q9D662	Protein transport protein Sec23B	Sec23b	0.9115	0.0261
604	Q99MR8	Methylcrotonoyl-CoA carboxylase subunit alpha mitochondrial	Mccc1	0.9112	0.0261
605	Q80TY0	Formin-binding protein 1	Fnbp1	0.9093	0.0262
606	Q8BGT7	Survival of motor neuron-related-splicing factor 30	Smndc1	0.9089	0.0262
607	Q9Z0U1	Tight junction protein ZO-2	Tjp2	0.9085	0.0263
608	Q8BIF2	RNA binding protein fox-1 homolog 3	Rbfox3	0.9082	0.0263
609	P06837	Neuromodulin	Gap43	0.9059	0.0264
610	Q80X80	Phospholipid transfer protein C2CD2L	C2cd2l	0.9045	0.0266
611	Q80TL7	Protein MON2 homolog	Mon2	0.9014	0.0267
612	Q8BGF9	Solute carrier family 25 member 44	Slc25a44	0.9012	0.0268
613	Q9CXJ4	Mitochondrial potassium channel ATP-binding subunit	Abcb8	0.8970	0.0272
614	Q91VM5	RNA binding motif protein X-linked-like-1	Rbmxl1	0.8964	0.0272
615	P60469	Liprin-alpha-3	Ppfia3	0.8959	0.0272
616	Q6P5E8	Diacylglycerol kinase theta	Dgkq	0.8931	0.0274
617	Q60676	Serine/threonine-protein phosphatase 5	Ppp5c	0.8918	0.0275
618	Q80XI3	Eukaryotic translation initiation factor 4 gamma 3	Eif4g3	0.8906	0.0275
619	Q9ESZ8	General transcription factor II-I	Gtf2i	0.8883	0.0277
620	Q80U28	MAP kinase-activating death domain protein	Madd	0.8878	0.0278
621	Q6PDC0	RUN domain-containing protein 3B	Rundc3b	0.8876	0.0279
622	Q6DFV3	Rho GTPase-activating protein 21	Arhgap21	0.8872	0.0282
623	Q61644	Protein kinase C and casein kinase substrate in neurons protein 1	Pacsin1	0.8863	0.0284
624	O55137	Acyl-coenzyme A thioesterase 1	Acot1	0.8854	0.0284
625	Q69ZR2	E3 ubiquitin-protein ligase HECTD1	Hectd1	0.8849	0.0284
626	Q8BYI9	Tenascin-R	Tnr	0.8831	0.0285
627	P61089	Ubiquitin-conjugating enzyme E2 N	Ube2n	0.8825	0.0286
628	Q8BG05	Heterogeneous nuclear ribonucleoprotein A3	Hnrnpa3	0.8811	0.0286
629	Q9JM96	Cdc42 effector protein 4	Cdc42ep4	0.8796	0.0287
630	Q4U2R1	E3 ubiquitin-protein ligase HERC2	Herc2	0.8796	0.0287
631	Q99LC2	Cleavage stimulation factor subunit 1	Cstf1	0.8791	0.0287
632	Q8R105	Vacuolar protein sorting-associated protein 37C	Vps37c	0.8791	0.0287
633	Q923M0	Protein phosphatase 1 regulatory subunit 16A	Ppp1r16a	0.8791	0.0287
634	Q7TMW6	Cytosolic iron-sulfur assembly component 3	Ciao3	0.8791	0.0287
635	Q8CAM5	Ras-related protein Rab-36	Rab36	0.8791	0.0287
636	Q9CWN7	CCR4-NOT transcription complex subunit 11	Cnot11	0.8791	0.0287
637	P70213	Friend virus susceptibility protein 1	Fv1	0.8791	0.0287
638	Q8R1U1	Conserved oligomeric Golgi complex subunit 4	Cog4	0.8791	0.0287

639	Q8BG89	Protein ZNF365	Znf365	0.8791	0.0287
640	Q9Z2X8	Kelch-like ECH-associated protein 1	Keap1	0.8791	0.0287
641	Q91YE3	Egl nine homolog 1	Egln1	0.8791	0.0287
642	Q8BPG6	Inactive C-alpha-formylglycine-generating enzyme 2	Sumf2	0.8791	0.0287
643	P39038	Cadherin-4	Cdh4	0.8791	0.0287
644	B2RXR6	Serine/threonine-protein phosphatase 6 regulatory ankyrin repeat subunit B	Ankrd44	0.8791	0.0287
645	Q9DBX3	Sushi domain-containing protein 2	Susd2	0.8791	0.0287
646	Q9D6T0	Nitric oxide synthase-interacting protein	Nosip	0.8791	0.0287
647	Q3UHU5	Microtubule cross-linking factor 1	Mtcl1	0.8791	0.0287
648	Q7TN22	Thioredoxin domain-containing protein 16	Txndc16	0.8791	0.0287
649	Q7TQF2	F-box only protein 10	Fbxo10	0.8791	0.0287
650	Q9D4F8	Gamma-tubulin complex component 4	Tubgcp4	0.8791	0.0287
651	Q6NZA9	Transcription initiation factor TFIID subunit 9B	Taf9b	0.8791	0.0287
652	Q8VI33	Transcription initiation factor TFIID subunit 9	Taf9	0.8791	0.0287
653	Q8BHR8	UPF0705 protein C11orf49 homolog		0.8791	0.0287
654	Q6P4T1	Sorting nexin-19	Snx19	0.8791	0.0287
655	O09172	Glutamate--cysteine ligase regulatory subunit	Gclm	0.8791	0.0287
656	Q6P6M7	O-phosphoseryl-tRNA(Sec) selenium transferase	Sepsecs	0.8791	0.0287
657	Q9Z0H3	SWI/SNF-related matrix-associated actin-dependent regulator of chromatin subfamily B member 1	Smarcb1	0.8791	0.0287
658	P59113	Fermitin family homolog 1	Fermt1	0.8791	0.0287
659	Q8CCG1	Zinc finger C2HC domain-containing protein 1C	Zc2hc1c	0.8791	0.0287
660	Q6NS46	Protein RRP5 homolog	Pdcd11	0.8791	0.0287
661	Q5S006	Leucine-rich repeat serine/threonine-protein kinase 2	Lrrk2	0.8791	0.0287
662	P35569	Insulin receptor substrate 1	Irs1	0.8791	0.0287
663	Q8BM55	Transmembrane protein 214	Tmem214	0.8791	0.0287
664	Q8K2A7	Integrator complex subunit 10	Ints10	0.8791	0.0287
665	Q99JF8	PC4 and SFRS1-interacting protein	Psip1	0.8788	0.0287
666	O88685	26S proteasome regulatory subunit 6A	Psmc3	0.8781	0.0287
667	Q8JZP2	Synapsin-3	Syn3	0.8768	0.0288
668	Q7TMK9	Heterogeneous nuclear ribonucleoprotein Q	Syncrip	0.8763	0.0288
669	Q8VEH5	EPM2A-interacting protein 1	Epm2aip1	0.8737	0.0290
670	Q61598	Rab GDP dissociation inhibitor beta	Gdi2	0.8735	0.0290
671	O55229	Choline/ethanolamine kinase	Chkb	0.8728	0.0290
672	Q8R313	Exocyst complex component 6	Exoc6	0.8715	0.0293
673	P55302	Alpha-2-macroglobulin receptor-associated protein	Lrpap1	0.8714	0.0293
674	Q9JKN6	RNA-binding protein Nova-1	Nova1	0.8711	0.0296
675	Q921S7	39S ribosomal protein L37 mitochondrial	Mrpl37	0.8710	0.0296
676	Q64455	Receptor-type tyrosine-protein phosphatase eta	Ptprj	0.8709	0.0296
677	P56695	Wolframin	Wfs1	0.8708	0.0296
678	Q9JIS5	Synaptic vesicle glycoprotein 2A	Sv2a	0.8689	0.0297
679	Q9CZ44	NSFL1 cofactor p47	Nsfl1c	0.8676	0.0299
680	Q62189	U1 small nuclear ribonucleoprotein A	Snrpa	0.8661	0.0300
681	Q9DCP2	Sodium-coupled neutral amino acid transporter 3	Slc38a3	0.8648	0.0300

Supplementary Table 2.

Number	Accession	Protein Name	Gene Name	PLGEM-STN	*p* Value
1	Q9CWF2	Tubulin beta-2B chain	Tubb2b	-8.9382	0.0000
2	Q7TMM9	Tubulin beta-2A chain	Tubb2a	-8.5405	0.0000
3	P68372	Tubulin beta-4B chain	Tubb4b	-7.7526	0.0000
4	P99024	Tubulin beta-5 chain	Tubb5	-6.7467	0.0000
5	P60202	Myelin proteolipid protein	Plp1	-6.6446	0.0000
6	Q9D6F9	Tubulin beta-4A chain	Tubb4a	-6.3012	0.0000
7	Q9CPQ1	Cytochrome c oxidase subunit 6C	Cox6c	-5.9010	0.0000
8	Q62418	Drebrin-like protein	Dbnl	-5.8414	0.0000
9	P01942	Hemoglobin subunit alpha	Hba	-5.7539	0.0000
10	P60710	Actin cytoplasmic 1	Actb	-5.7398	0.0000
11	P63260	Actin cytoplasmic 2	Actg1	-5.5769	0.0000
12	P68033	Actin alpha cardiac muscle 1	Actc1	-5.3626	0.0000
13	Q9JHU4	Cytoplasmic dynein 1 heavy chain 1	Dync1h1	-5.1984	0.0000
14	Q9Z140	Copine-6	Cpne6	-5.0066	0.0000
15	Q9JJV2	Profilin-2	Pfn2	-4.9151	0.0000
16	P05784	Keratin type I cytoskeletal 18	Krt18	-4.3676	0.0000
17	P02535	Keratin type I cytoskeletal 10	Krt10	-4.3272	0.0000
18	P15533	Tripartite motif-containing protein 30A	Trim30a	-4.3204	0.0000
19	P04370	Myelin basic protein	Mbp	-4.3184	0.0000
20	P68254	14-3-3 protein theta	Ywhaq	-4.2408	0.0000
21	Q9QWL7	Keratin type I cytoskeletal 17	Krt17	-4.2240	0.0000
22	Q921I1	Serotransferrin	Tf	-4.1825	0.0000
23	P11499	Heat shock protein HSP 90-beta	Hsp90ab1	-4.1810	0.0000
24	P62806	Histone H4	H4c1	-4.1660	0.0000
25	Q61414	Keratin type I cytoskeletal 15	Krt15	-4.0963	0.0000
26	Q06185	ATP synthase subunit e mitochondrial	Atp5me	-3.9996	0.0000
27	P12246	Serum amyloid P-component	Apcs	-3.9651	0.0000
28	P59764	Dedicator of cytokinesis protein 4	Dock4	-3.8862	0.0000
29	P04104	Keratin type II cytoskeletal 1	Krt1	-3.8859	0.0000
30	E9PV24	Fibrinogen alpha chain	Fga	-3.8459	0.0000
31	Q9CQZ6	NADH dehydrogenase [ubiquinone] 1 beta subcomplex subunit 3	Ndufb3	-3.8278	0.0000
32	P19536	Cytochrome c oxidase subunit 5B mitochondrial	Cox5b	-3.7770	0.0000
33	P01027	Complement C3	C3	-3.7380	0.0000
34	P08730	Keratin type I cytoskeletal 13	Krt13	-3.6619	0.0000
35	Q61781	Keratin type I cytoskeletal 14	Krt14	-3.6578	0.0000
36	Q9Z2K1	Keratin type I cytoskeletal 16	Krt16	-3.6366	0.0000
37	Q61016	Guanine nucleotide-binding protein G(I)/G(S)/G(O) subunit gamma-7	Gng7	-3.6163	0.0000
38	P48453	Serine/threonine-protein phosphatase 2B catalytic subunit beta isoform	Ppp3cb	-3.6034	0.0000
39	P63101	14-3-3 protein zeta/delta	Ywhaz	-3.5900	0.0000
40	P62897	Cytochrome c somatic	Cycs	-3.5703	0.0000
41	P03995	Glial fibrillary acidic protein	Gfap	-3.5126	0.0000
42	Q9DB05	Alpha-soluble NSF attachment protein	Napa	-3.5106	0.0000
43	O55143	Sarcoplasmic/endoplasmic reticulum calcium ATPase 2	Atp2a2	-3.4541	0.0000
44	Q8CIQ7	Dedicator of cytokinesis protein 3	Dock3	-3.4138	0.0000
45	Q922U2	Keratin type II cytoskeletal 5	Krt5	-3.3870	0.0000
46	P19783	Cytochrome c oxidase subunit 4 isoform 1 mitochondrial	Cox4i1	-3.3805	0.0000
47	P11798	Calcium/calmodulin-dependent protein kinase type II subunit alpha	Camk2a	-3.3767	0.0000
48	Q9CQV8	14-3-3 protein beta/alpha	Ywhab	-3.3447	0.0000
49	Q6PIC6	Sodium/potassium-transporting ATPase subunit alpha-3	Atp1a3	-3.3330	0.0000
50	Q7TSC1	Protein PRRC2A	Prrc2a	-3.3318	0.0000
51	P56399	Ubiquitin carboxyl-terminal hydrolase 5	Usp5	-3.3161	0.0000
52	P01869	Ig gamma-1 chain C region membrane-bound form	Ighg1	-3.3143	0.0000
53	Q6IFZ6	Keratin type II cytoskeletal 1b	Krt77	-3.2823	0.0000
54	Q9D0M5	Dynein light chain 2 cytoplasmic	Dynll2	-3.2543	0.0000
55	P62259	14-3-3 protein epsilon	Ywhae	-3.2259	0.0000
56	Q3TTY5	Keratin type II cytoskeletal 2 epidermal	Krt2	-3.2243	0.0000
57	Q91WS0	CDGSH iron-sulfur domain-containing protein 1	Cisd1	-3.1892	0.0000
58	P01868	Ig gamma-1 chain C region secreted form	Ighg1	-3.1743	0.0000
59	Q922F4	Tubulin beta-6 chain	Tubb6	-3.1732	0.0000
60	Q9WV96	Mitochondrial import inner membrane translocase subunit Tim10 B	Timm10b	-3.1675	0.0000
61	Q9CQ69	Cytochrome b-c1 complex subunit 8	Uqcrq	-3.1444	0.0000
62	P02088	Hemoglobin subunit beta-1	Hbb-b1	-3.1278	0.0001
63	Q78IK2	ATP synthase membrane subunit K mitochondrial	Atp5mk	-3.1134	0.0001
64	P62823	Ras-related protein Rab-3C	Rab3c	-3.1129	0.0001
65	Q4FZF3	Probable ATP-dependent RNA helicase DDX49	Ddx49	-3.1124	0.0001

66	P62962	Profilin-1	Pfn1	-3.1006	0.0001
67	Q6NZC7	SEC23-interacting protein	Sec23ip	-3.0933	0.0001
68	P19001	Keratin type I cytoskeletal 19	Krt19	-3.0855	0.0001
69	P10649	Glutathione S-transferase Mu 1	Gstm1	-3.0638	0.0001
70	O55142	60S ribosomal protein L35a	Rpl35a	-3.0492	0.0001
71	P59108	Copine-2	Cpne2	-3.0289	0.0001
72	Q91YE6	Importin-9	Ipo9	-3.0154	0.0001
73	Q812A2	SLIT-ROBO Rho GTPase-activating protein 3	Srgap3	-3.0053	0.0001
74	P02089	Hemoglobin subunit beta-2	Hbb-b2	-3.0037	0.0001
75	Q8BFZ3	Beta-actin-like protein 2	Actbl2	-3.0022	0.0001
76	P11404	Fatty acid-binding protein heart	Fabp3	-2.9912	0.0001
77	Q3UX10	Tubulin alpha chain-like 3	Tubal3	-2.9804	0.0001
78	F6SEU4	Ras/Rap GTPase-activating protein SynGAP	Syngap1	-2.9573	0.0001
79	Q9QUM9	Proteasome subunit alpha type-6	Psma6	-2.9517	0.0001
80	Q9QXZ0	Microtubule-actin cross-linking factor 1	Macf1	-2.9399	0.0001
81	P07901	Heat shock protein HSP 90-alpha	Hsp90aa1	-2.9264	0.0001
82	Q9QVP9	Protein-tyrosine kinase 2-beta	Ptk2b	-2.9241	0.0001
83	Q9CQR4	Acyl-coenzyme A thioesterase 13	Acot13	-2.9032	0.0001
84	P52503	NADH dehydrogenase [ubiquinone] iron-sulfur protein 6 mitochondrial	Ndufs6	-2.8954	0.0001
85	Q9JLF6	Protein-glutamine gamma-glutamyltransferase K	Tgm1	-2.8909	0.0001
86	Q80ZJ1	Ras-related protein Rap-2a	Rap2a	-2.8685	0.0001
87	Q9R0H5	Keratin type II cytoskeletal 71	Krt71	-2.8624	0.0001
88	P35846	Folate receptor alpha	Folr1	-2.8569	0.0001
89	P63328	Serine/threonine-protein phosphatase 2B catalytic subunit alpha isoform	Ppp3ca	-2.8295	0.0001
90	P63216	Guanine nucleotide-binding protein G(I)/G(S)/G(O) subunit gamma-3	Gng3	-2.8255	0.0001
91	Q91V41	Ras-related protein Rab-14	Rab14	-2.8168	0.0001
92	Q8BPN8	DmX-like protein 2	Dmxl2	-2.8124	0.0001
93	P62737	Actin aortic smooth muscle	Acta2	-2.8117	0.0001
94	P16546	Spectrin alpha chain non-erythrocytic 1	Sptan1	-2.7844	0.0001
95	Q9CPQ8	ATP synthase subunit g mitochondrial	Atp5mg	-2.7807	0.0001
96	P13595	Neural cell adhesion molecule 1	Ncam1	-2.7493	0.0001
97	P20152	Vimentin	Vim	-2.7416	0.0001
98	P14873	Microtubule-associated protein 1B	Map1b	-2.7318	0.0001
99	Q91V16	Electron transfer flavoprotein regulatory factor 1	Etfrf1	-2.7222	0.0001
100	Q99PT1	Rho GDP-dissociation inhibitor 1	Arhgdia	-2.7190	0.0001
101	P63158	High mobility group protein B1	Hmgb1	-2.7125	0.0001
102	P21460	Cystatin-C	Cst3	-2.7097	0.0001
103	P52760	2-iminobutanoate/2-iminopropanoate deaminase	Rida	-2.7058	0.0001
104	P07309	Transthyretin	Ttr	-2.7037	0.0001
105	O35382	Exocyst complex component 4	Exoc4	-2.6968	0.0001
106	Q5SYD0	Unconventional myosin-Id	Myo1d	-2.6930	0.0001
107	Q9D855	Cytochrome b-c1 complex subunit 7	Uqcrb	-2.6868	0.0001
108	P63024	Vesicle-associated membrane protein 3	Vamp3	-2.6682	0.0001
109	Q9Z0R4	Intersectin-1	Itsn1	-2.6661	0.0001
110	Q19LI2	Alpha-1B-glycoprotein	A1bg	-2.6570	0.0001
111	Q9DAS9	Guanine nucleotide-binding protein G(I)/G(S)/G(O) subunit gamma-12	Gng12	-2.6507	0.0001
112	P39061	Collagen alpha-1(XVIII) chain	Col18a1	-2.6420	0.0001
113	Q6NXH9	Keratin type II cytoskeletal 73	Krt73	-2.6167	0.0001
114	P0C8K7	Small integral membrane protein 1	Smim1	-2.6149	0.0001
115	P70261	Paladin	Pald1	-2.6039	0.0001
116	Q61699	Heat shock protein 105 kDa	Hsph1	-2.6001	0.0001
117	P63323	40S ribosomal protein S12	Rps12	-2.5986	0.0001
118	P05532	Mast/stem cell growth factor receptor Kit	Kit	-2.5970	0.0001
119	P23492	Purine nucleoside phosphorylase	Pnp	-2.5897	0.0001
120	Q9JHS3	Ragulator complex protein LAMTOR2	Lamtor2	-2.5806	0.0001
121	Q8BHB9	Chloride intracellular channel protein 6	Clic6	-2.5704	0.0002
122	Q80YA9	Connector enhancer of kinase suppressor of ras 2	Cnksr2	-2.5650	0.0002
123	P0DP60	Ly-6/neurotoxin-like protein 1	Lynx1	-2.5538	0.0002
124	Q11011	Puromycin-sensitive aminopeptidase	Npepps	-2.5495	0.0002
125	Q9DCC8	Mitochondrial import receptor subunit TOM20 homolog	Tomm20	-2.5444	0.0002
126	Q6IFX2	Keratin type I cytoskeletal 42	Krt42	-2.5272	0.0002
127	P62307	Small nuclear ribonucleoprotein F	Snrpf	-2.4954	0.0002
128	P63011	Ras-related protein Rab-3A	Rab3a	-2.4838	0.0002
129	Q9JJU8	SH3 domain-binding glutamic acid-rich-like protein	Sh3bgrl	-2.4782	0.0002
130	P63030	Mitochondrial pyruvate carrier 1	Mpc1	-2.4757	0.0002
131	Q8K3J1	NADH dehydrogenase [ubiquinone] iron-sulfur protein 8 mitochondrial	Ndufs8	-2.4706	0.0002
132	P63044	Vesicle-associated membrane protein 2	Vamp2	-2.4703	0.0002

133	Q9WV55	Vesicle-associated membrane protein-associated protein A	Vapa	-2.4631	0.0002
134	P56391	Cytochrome c oxidase subunit 6B1	Cox6b1	-2.4435	0.0002
135	P56379	ATP synthase subunit ATP5MJ mitochondrial	Atp5mj	-2.4350	0.0002
136	Q80ZW2	Protein THEM6	Them6	-2.4349	0.0002
137	O08709	Peroxiredoxin-6	Prdx6	-2.4050	0.0002
138	P47757	F-actin-capping protein subunit beta	Capzb	-2.3956	0.0002
139	Q4KUS2	Protein unc-13 homolog A	Unc13a	-2.3955	0.0002
140	Q8R164	Valacyclovir hydrolase	Bphl	-2.3908	0.0002
141	P62702	40S ribosomal protein S4 X isoform	Rps4x	-2.3902	0.0002
142	P20444	Protein kinase C alpha type	Prkca	-2.3693	0.0002
143	P61982	14-3-3 protein gamma	Ywhag	-2.3623	0.0002
144	Q0VE82	Copine-7	Cpne7	-2.3585	0.0002
145	Q6PB66	Leucine-rich PPR motif-containing protein mitochondrial	Lrpprc	-2.3562	0.0002
146	Q61495	Desmoglein-1-alpha	Dsg1a	-2.3433	0.0003
147	Q7TSF1	Desmoglein-1-beta	Dsg1b	-2.3433	0.0003
148	Q5SXY1	Cytospin-B	Specc1	-2.3365	0.0003
149	Q8CHC4	Synaptojanin-1	Synj1	-2.3189	0.0003
150	Q9D2R6	Cytochrome c oxidase assembly factor 3 homolog mitochondrial	Coa3	-2.3143	0.0003
151	Q9WTT4	V-type proton ATPase subunit G 2	Atp6v1g2	-2.3122	0.0003
152	P17897	Lysozyme C-1	Lyz1	-2.3084	0.0003
153	P35276	Ras-related protein Rab-3D	Rab3d	-2.3083	0.0003
154	P68510	14-3-3 protein eta	Ywhah	-2.3005	0.0003
155	P51880	Fatty acid-binding protein brain	Fabp7	-2.3000	0.0003
156	P19096	Fatty acid synthase	Fasn	-2.2986	0.0003
157	E9Q3L2	Phosphatidylinositol 4-kinase alpha	Pi4ka	-2.2949	0.0003
158	P55066	Neurocan core protein	Ncan	-2.2838	0.0003
159	P31786	Acyl-CoA-binding protein	Dbi	-2.2832	0.0003
160	P15626	Glutathione S-transferase Mu 2	Gstm2	-2.2763	0.0003
161	O55042	Alpha-synuclein	Snca	-2.2724	0.0003
162	Q9EQK5	Major vault protein	Mvp	-2.2700	0.0003
163	P63038	60 kDa heat shock protein mitochondrial	Hspd1	-2.2657	0.0003
164	Q61646	Haptoglobin	Hp	-2.2601	0.0003
165	Q62261	Spectrin beta chain non-erythrocytic 1	Sptbn1	-2.2545	0.0003
166	A1L317	Keratin type I cytoskeletal 24	Krt24	-2.2443	0.0003
167	P84075	Neuron-specific calcium-binding protein hippocalcin	Hpca	-2.2368	0.0003
168	P16054	Protein kinase C epsilon type	Prkce	-2.2312	0.0003
169	O35551	Rab GTPase-binding effector protein 1	Rabep1	-2.2242	0.0003
170	Q8K0E8	Fibrinogen beta chain	Fgb	-2.2225	0.0003
171	Q3UGR5	Haloacid dehalogenase-like hydrolase domain-containing protein 2	Hdhd2	-2.2194	0.0003
172	Q6NWV3	Intraflagellar transport protein 122 homolog	Ift122	-2.2095	0.0003
173	Q80WR1	Tetraspanin-18	Tspan18	-2.2095	0.0003
174	Q5SQX6	Cytoplasmic FMR1-interacting protein 2	Cyfip2	-2.1929	0.0003
175	Q99J47	Dehydrogenase/reductase SDR family member 7B	Dhrs7b	-2.1796	0.0004
176	Q9Z2V6	Histone deacetylase 5	Hdac5	-2.1786	0.0004
177	O54983	Ketimine reductase mu-crystallin	Crym	-2.1779	0.0004
178	P48758	Carbonyl reductase [NADPH] 1	Cbr1	-2.1748	0.0004
179	Q60931	Voltage-dependent anion-selective channel protein 3	Vdac3	-2.1703	0.0004
180	Q60605	Myosin light polypeptide 6	Myl6	-2.1659	0.0004
181	P19157	Glutathione S-transferase P 1	Gstp1	-2.1486	0.0004
182	Q9QZ88	Vacuolar protein sorting-associated protein 29	Vps29	-2.1339	0.0005
183	P57746	V-type proton ATPase subunit D	Atp6v1d	-2.1334	0.0005
184	Q9CQ65	S-methyl-5'-thioadenosine phosphorylase	Mtap	-2.1198	0.0005
185	Q99LX0	Parkinson disease protein 7 homolog	Park7	-2.1140	0.0005
186	Q9R0P9	Ubiquitin carboxyl-terminal hydrolase isozyme L1	Uchl1	-2.1122	0.0005
187	P18242	Cathepsin D	Ctsd	-2.1083	0.0005
188	P48774	Glutathione S-transferase Mu 5	Gstm5	-2.1024	0.0005
189	Q9Z1T2	Thrombospondin-4	Thbs4	-2.0897	0.0005
190	Q4KMM3	Oxidation resistance protein 1	Oxr1	-2.0875	0.0005
191	Q64105	Sepiapterin reductase	Spr	-2.0813	0.0005
192	O54940	BCL2/adenovirus E1B 19 kDa protein-interacting protein 2	Bnip2	-2.0789	0.0005
193	Q9D379	Epoxide hydrolase 1	Ephx1	-2.0673	0.0005
194	Q9Z2I9	Succinate--CoA ligase [ADP-forming] subunit beta mitochondrial	Sucla2	-2.0672	0.0005
195	Q8K1X4	Nck-associated protein 1-like	Nckap1l	-2.0670	0.0005
196	P61294	Ras-related protein Rab-6B	Rab6b	-2.0626	0.0005
197	Q9JMH9	Unconventional myosin-XVIIIa	Myo18a	-2.0582	0.0005
198	Q9EPN1	Neurobeachin	Nbea	-2.0559	0.0005
199	Q9QXS1	Plectin	Plec	-2.0504	0.0005

200	P52480	Pyruvate kinase PKM	Pkm	-2.0425	0.0005
201	Q8VCM7	Fibrinogen gamma chain	Fgg	-2.0425	0.0005
202	P48455	Serine/threonine-protein phosphatase 2B catalytic subunit gamma isoform	Ppp3cc	-2.0380	0.0005
203	P83940	Elongin-C	Eloc	-2.0369	0.0005
204	Q8BYR5	Calcium-dependent secretion activator 2	Cadps2	-2.0364	0.0005
205	P51910	Apolipoprotein D	Apod	-2.0362	0.0005
206	P60521	Gamma-aminobutyric acid receptor-associated protein-like 2	Gabarapl2	-2.0358	0.0005
207	P61205	ADP-ribosylation factor 3	Arf3	-2.0283	0.0005
208	Q8CI71	Syndetin	Vps50	-2.0152	0.0006
209	Q9WTZ1	RING-box protein 2	Rnf7	-2.0056	0.0006
210	P61226	Ras-related protein Rap-2b	Rap2b	-2.0041	0.0006
211	Q80TJ1	Calcium-dependent secretion activator 1	Cadps	-1.9912	0.0006
212	P84078	ADP-ribosylation factor 1	Arf1	-1.9870	0.0006
213	P58252	Elongation factor 2	Eef2	-1.9862	0.0006
214	B1AQ75	Keratin type I cuticular Ha6	Krt36	-1.9854	0.0006
215	Q61166	Microtubule-associated protein RP/EB family member 1	Mapre1	-1.9853	0.0006
216	Q3UJP5	Protein C8orf37 homolog		-1.9828	0.0006
217	Q9CQ54	NADH dehydrogenase [ubiquinone] 1 subunit C2	Ndufc2	-1.9786	0.0006
218	Q7TMB8	Cytoplasmic FMR1-interacting protein 1	Cyfip1	-1.9771	0.0006
219	Q9D8S9	BolA-like protein 1	Bola1	-1.9764	0.0006
220	O35066	Kinesin-like protein KIF3C	Kif3c	-1.9711	0.0006
221	P21107	Tropomyosin alpha-3 chain	Tpm3	-1.9594	0.0006
222	P05213	Tubulin alpha-1B chain	Tuba1b	-1.9488	0.0006
223	Q8BLR2	Copine-4	Cpne4	-1.9483	0.0006
224	P19639	Glutathione S-transferase Mu 3	Gstm3	-1.9439	0.0006
225	A2ALK8	Tyrosine-protein phosphatase non-receptor type 3	Ptpn3	-1.9417	0.0006
226	Q8C8R3	Ankyrin-2	Ank2	-1.9413	0.0006
227	Q8CHU3	Epsin-2	Epn2	-1.9367	0.0006
228	Q01853	Transitional endoplasmic reticulum ATPase	Vcp	-1.9351	0.0006
229	O70589	Peripheral plasma membrane protein CASK	Cask	-1.9343	0.0006
230	P12787	Cytochrome c oxidase subunit 5A mitochondrial	Cox5a	-1.9305	0.0007
231	Q9EST1	Gasdermin-A	Gsdma	-1.9305	0.0007
232	Q9CR00	26S proteasome non-ATPase regulatory subunit 9	Psmd9	-1.9246	0.0007
233	P56480	ATP synthase subunit beta mitochondrial	Atp5f1b	-1.9126	0.0007
234	P35803	Neuronal membrane glycoprotein M6-b	Gpm6b	-1.9093	0.0007
235	Q8C0M9	Isoaspartyl peptidase/L-asparaginase	Asrgl1	-1.9048	0.0007
236	P62774	Myotrophin	Mtpn	-1.9033	0.0007
237	Q9D2P8	Myelin-associated oligodendrocyte basic protein	Mobp	-1.9024	0.0007
238	Q4FK66	Pre-mRNA-splicing factor 38A	Prpf38a	-1.9012	0.0007
239	Q62446	Peptidyl-prolyl cis-trans isomerase FKBP3	Fkbp3	-1.8907	0.0007
240	P70336	Rho-associated protein kinase 2	Rock2	-1.8826	0.0007
241	P0DP27	Calmodulin-2	Calm2	-1.8809	0.0007
242	P0DP28	Calmodulin-3	Calm3	-1.8809	0.0007
243	P0DP26	Calmodulin-1	Calm1	-1.8809	0.0007
244	Q9DBG3	AP-2 complex subunit beta	Ap2b1	-1.8780	0.0007
245	P97384	Annexin A11	Anxa11	-1.8688	0.0007
246	Q69ZH9	Rho GTPase-activating protein 23	Arhgap23	-1.8649	0.0008
247	Q9Z0E0	Neurochondrin	Ncdn	-1.8636	0.0008
248	P00015	Cytochrome c testis-specific	Cyct	-1.8594	0.0008
249	Q8CFV4	Neuritin	Nrn1	-1.8552	0.0008
250	Q9D1K2	V-type proton ATPase subunit F	Atp6v1f	-1.8552	0.0008
251	Q8BU31	Ras-related protein Rap-2c	Rap2c	-1.8514	0.0008
252	Q9DAK9	14 kDa phosphohistidine phosphatase	Phpt1	-1.8491	0.0008
253	Q3UH99	Protein shisa-6	Shisa6	-1.8471	0.0008
254	P97350	Plakophilin-1	Pkp1	-1.8466	0.0008
255	Q9D3D9	ATP synthase subunit delta mitochondrial	Atp5f1d	-1.8453	0.0008
256	Q6NXK7	Inactive dipeptidyl peptidase 10	Dpp10	-1.8447	0.0008
257	P46460	Vesicle-fusing ATPase	Nsf	-1.8441	0.0008
258	Q05816	Fatty acid-binding protein 5	Fabp5	-1.8430	0.0008
259	Q64433	10 kDa heat shock protein mitochondrial	Hspe1	-1.8424	0.0008
260	P63321	Ras-related protein Ral-A	Rala	-1.8417	0.0008
261	Q9Z1P6	NADH dehydrogenase [ubiquinone] 1 alpha subcomplex subunit 7	Ndufa7	-1.8397	0.0008
262	P56564	Excitatory amino acid transporter 1	Slc1a3	-1.8370	0.0008
263	Q8BYA0	Tubulin-specific chaperone D	Tbcd	-1.8358	0.0008
264	P06909	Complement factor H	Cfh	-1.8346	0.0008
265	Q61387	Cytochrome c oxidase subunit 7A-related protein mitochondrial	Cox7a2l	-1.8346	0.0008
266	Q9R1P1	Proteasome subunit beta type-3	Psmb3	-1.8271	0.0008

267	P70349	Histidine triad nucleotide-binding protein 1	Hint1	-1.8239	0.0008
268	Q9QYX7	Protein piccolo	Pclo	-1.8196	0.0008
269	P70704	Phospholipid-transporting ATPase IA	Atp8a1	-1.8185	0.0008
270	P26883	Peptidyl-prolyl cis-trans isomerase FKBP1A	Fkbp1a	-1.8134	0.0008
271	Q0VBF8	Protein stum homolog	Stum	-1.8110	0.0008
272	Q80X90	Filamin-B	Flnb	-1.8101	0.0008
273	Q9JLQ2	ARF GTPase-activating protein GIT2	Git2	-1.8093	0.0008
274	P70275	Semaphorin-3E	Sema3e	-1.8011	0.0009
275	Q9JI75	Ribosyldihydronicotinamide dehydrogenase [quinone]	Nqo2	-1.8003	0.0009
276	Q9WU78	Programmed cell death 6-interacting protein	Pdcd6ip	-1.8001	0.0009
277	Q9R1S8	Calpain-7	Capn7	-1.7991	0.0009
278	Q08189	Protein-glutamine gamma-glutamyltransferase E	Tgm3	-1.7886	0.0009
279	O54962	Barrier-to-autointegration factor	Banf1	-1.7886	0.0009
280	P40240	CD9 antigen	Cd9	-1.7880	0.0009
281	Q8VED5	Keratin type II cytoskeletal 79	Krt79	-1.7871	0.0009
282	E9Q401	Ryanodine receptor 2	Ryr2	-1.7865	0.0009
283	Q8C1B1	Calmodulin-regulated spectrin-associated protein 2	Camsap2	-1.7742	0.0010
284	Q5U3K5	Rab-like protein 6	Rabl6	-1.7738	0.0010
285	P62855	40S ribosomal protein S26	Rps26	-1.7723	0.0010
286	P01029	Complement C4-B	C4b	-1.7692	0.0010
287	Q9CQD1	Ras-related protein Rab-5A	Rab5a	-1.7670	0.0010
288	Q9D6G9	CKLF-like MARVEL transmembrane domain-containing protein 5	Cmtm5	-1.7654	0.0010
289	Q4PJX1	Protein odr-4 homolog	Odr4	-1.7636	0.0010
290	Q8VE19	GATOR complex protein MI	Mios	-1.7600	0.0010
291	Q922D8	C-1-tetrahydrofolate synthase cytoplasmic	Mthfd1	-1.7502	0.0010
292	Q62425	Cytochrome c oxidase subunit NDUFA4	Ndufa4	-1.7484	0.0010
293	Q05920	Pyruvate carboxylase mitochondrial	Pc	-1.7483	0.0010
294	P62242	40S ribosomal protein S8	Rps8	-1.7481	0.0010
295	P15508	Spectrin beta chain erythrocytic	Sptb	-1.7464	0.0010
296	P51881	ADP/ATP translocase 2	Slc25a5	-1.7439	0.0010
297	Q6WKZ8	E3 ubiquitin-protein ligase UBR2	Ubr2	-1.7438	0.0010
298	Q9CWU6	Ubiquinol-cytochrome-c reductase complex assembly factor 1	Uqcc1	-1.7339	0.0010
299	P62317	Small nuclear ribonucleoprotein Sm D2	Snrpd2	-1.7297	0.0011
300	Q6PIE5	Sodium/potassium-transporting ATPase subunit alpha-2	Atp1a2	-1.7295	0.0011
301	Q9Z0J4	Nitric oxide synthase brain	Nos1	-1.7211	0.0011
302	Q9JJH1	Ribonuclease 4	Rnase4	-1.7201	0.0011
303	Q9D4F2	Phospholipid phosphatase 6	Plpp6	-1.7191	0.0011
304	O08600	Endonuclease G mitochondrial	Endog	-1.7191	0.0011
305	Q60994	Adiponectin	Adipoq	-1.7191	0.0011
306	Q5U5V2	Hydroxylysine kinase	Hykk	-1.7191	0.0011
307	P00329	Alcohol dehydrogenase 1	Adh1	-1.7191	0.0011
308	P0C1Q2	Dual 3' 5'-cyclic-AMP and -GMP phosphodiesterase 11A	Pde11a	-1.7191	0.0011
309	Q8C6M1	Ubiquitin carboxyl-terminal hydrolase 20	Usp20	-1.7191	0.0011
310	Q8VCC8	Rap guanine nucleotide exchange factor 3	Rapgef3	-1.7163	0.0011
311	Q80YX1	Tenascin	Tnc	-1.7146	0.0011
312	P10922	Histone H1.0	H1-0	-1.7129	0.0011
313	Q9DAM5	Mitochondrial thiamine pyrophosphate carrier	Slc25a19	-1.7099	0.0011
314	Q9CZL5	Pterin-4-alpha-carbinolamine dehydratase 2	Pcbd2	-1.7099	0.0011
315	Q9JLR9	HIG1 domain family member 1A mitochondrial	Higd1a	-1.7099	0.0011
316	P48428	Tubulin-specific chaperone A	Tbca	-1.7099	0.0011
317	Q99JY9	Actin-related protein 3	Actr3	-1.7091	0.0011
318	Q8JZQ9	Eukaryotic translation initiation factor 3 subunit B	Eif3b	-1.7073	0.0011
319	Q7TQD2	Tubulin polymerization-promoting protein	Tppp	-1.7046	0.0011
320	Q9D1G1	Ras-related protein Rab-1B	Rab1b	-1.6951	0.0011
321	P67778	Prohibitin	Phb	-1.6900	0.0011
322	P06801	NADP-dependent malic enzyme	Me1	-1.6891	0.0011
323	Q80W21	Glutathione S-transferase Mu 7	Gstm7	-1.6870	0.0011
324	Q9CYR0	Single-stranded DNA-binding protein mitochondrial	Ssbp1	-1.6861	0.0011
325	Q2PFD7	PH and SEC7 domain-containing protein 3	Psd3	-1.6861	0.0011
326	Q02105	Complement C1q subcomponent subunit C	C1qc	-1.6839	0.0011
327	Q9Z1G3	V-type proton ATPase subunit C 1	Atp6v1c1	-1.6818	0.0011
328	Q91Z38	Tetratricopeptide repeat protein 1	Ttc1	-1.6815	0.0012
329	Q9D1N9	39S ribosomal protein L21 mitochondrial	Mrpl21	-1.6815	0.0012
330	Q8BVI4	Dihydropteridine reductase	Qdpr	-1.6813	0.0012
331	P35700	Peroxiredoxin-1	Prdx1	-1.6783	0.0012
332	Q9CZT8	Ras-related protein Rab-3B	Rab3b	-1.6775	0.0012
333	P68040	Receptor of activated protein C kinase 1	Rack1	-1.6769	0.0012

334	Q9WUE3	Transmembrane reductase CYB561D2	Cyb561d2	-1.6732	0.0012
335	Q99104	Unconventional myosin-Va	Myo5a	-1.6732	0.0012
336	P17156	Heat shock-related 70 kDa protein 2	Hspa2	-1.6674	0.0012
337	P28740	Kinesin-like protein KIF2A	Kif2a	-1.6671	0.0012
338	Q3THE2	Myosin regulatory light chain 12B	Myl12b	-1.6647	0.0012
339	O08677	Kininogen-1	Kng1	-1.6585	0.0012
340	Q9JJ69	Kv channel-interacting protein 2	Kcnip2	-1.6571	0.0013
341	Q8K097	Protein lifeguard 2	Faim2	-1.6571	0.0013
342	Q9CY00	Tetratricopeptide repeat protein 30B	Ttc30b	-1.6571	0.0013
343	Q80U35	Rho guanine nucleotide exchange factor 17	Arhgef17	-1.6562	0.0013
344	A2AGT5	Cytoskeleton-associated protein 5	Ckap5	-1.6555	0.0013
345	Q60771	Claudin-11	Cldn11	-1.6548	0.0013
346	P17710	Hexokinase-1	Hk1	-1.6515	0.0013
347	O55023	Inositol monophosphatase 1	Impa1	-1.6501	0.0013
348	Q5PR73	GTP-binding protein Di-Ras2	Diras2	-1.6491	0.0013
349	Q80XN0	D-beta-hydroxybutyrate dehydrogenase mitochondrial	Bdh1	-1.6481	0.0013
350	Q9CQI6	Coactosin-like protein	Cotl1	-1.6442	0.0013
351	P35279	Ras-related protein Rab-6A	Rab6a	-1.6407	0.0013
352	Q9QXS6	Drebrin	Dbn1	-1.6364	0.0013
353	Q3UHD1	Adhesion G protein-coupled receptor B1	Adgrb1	-1.6363	0.0013
354	Q91X97	Neurocalcin-delta	Ncald	-1.6287	0.0013
355	P21614	Vitamin D-binding protein	Gc	-1.6276	0.0013
356	Q61176	Arginase-1	Arg1	-1.6276	0.0013
357	P63242	Eukaryotic translation initiation factor 5A-1	Eif5a	-1.6268	0.0013
358	Q6ZQ38	Cullin-associated NEDD8-dissociated protein 1	Cand1	-1.6215	0.0014
359	P62748	Hippocalcin-like protein 1	Hpcal1	-1.6208	0.0014
360	Q8BHZ0	CYFIP-related Rac1 interactor A	Cyria	-1.6173	0.0014
361	Q6NVE9	Protein phosphatase PTC7 homolog	Pptc7	-1.6163	0.0014
362	Q9WVK4	EH domain-containing protein 1	Ehd1	-1.6131	0.0014
363	Q9QYR6	Microtubule-associated protein 1A	Map1a	-1.6130	0.0014
364	Q9DBG5	Perilipin-3	Plin3	-1.6107	0.0014
365	P35282	Ras-related protein Rab-21	Rab21	-1.6096	0.0014
366	P68373	Tubulin alpha-1C chain	Tuba1c	-1.6092	0.0014
367	P35278	Ras-related protein Rab-5C	Rab5c	-1.6083	0.0014
368	Q9D051	Pyruvate dehydrogenase E1 component subunit beta mitochondrial	Pdhb	-1.6068	0.0014
369	Q8BGY7	Protein FAM210A	Fam210a	-1.6028	0.0014
370	Q6ZPE2	Myotubularin-related protein 5	Sbf1	-1.6021	0.0014
371	Q9CQR2	40S ribosomal protein S21	Rps21	-1.6016	0.0014
372	Q8BKI2	Trinucleotide repeat-containing gene 6B protein	Tnrc6b	-1.6009	0.0014
373	Q3UVC0	Kinase suppressor of Ras 2	Ksr2	-1.6009	0.0014
374	Q80SY4	E3 ubiquitin-protein ligase MIB1	Mib1	-1.6009	0.0014
375	Q99JY4	TraB domain-containing protein	Trabd	-1.6009	0.0014
376	Q8BVA2	Transmembrane protein 222	Tmem222	-1.6009	0.0014
377	O88630	Golgi SNAP receptor complex member 1	Gosr1	-1.6009	0.0014
378	P62274	40S ribosomal protein S29	Rps29	-1.6009	0.0014
379	Q9CQX0	Ubiquitin thioesterase OTUB2	Otub2	-1.6009	0.0014
380	Q60675	Laminin subunit alpha-2	Lama2	-1.6002	0.0014
381	P01638	Ig kappa chain V-V region L6 (Fragment)	4 SV	-1.6002	0.0014
382	P01637	Ig kappa chain V-V region T1	4 SV	-1.6002	0.0014
383	Q9D2G5	Synaptojanin-2	Synj2	-1.6002	0.0014
384	Q58A65	C-Jun-amino-terminal kinase-interacting protein 4	Spag9	-1.5994	0.0014
385	P68369	Tubulin alpha-1A chain	Tuba1a	-1.5940	0.0015
386	Q8CF66	Ragulator complex protein LAMTOR4	Lamtor4	-1.5917	0.0015
387	P29391	Ferritin light chain 1	Ftl1	-1.5903	0.0015
388	Q9D1E6	Tubulin-folding cofactor B	Tbcb	-1.5886	0.0015
389	Q62348	Translin	Tsn	-1.5848	0.0015
390	Q99L43	Phosphatidate cytidylyltransferase 2	Cds2	-1.5811	0.0015
391	Q60936	Atypical kinase COQ8A mitochondrial	Coq8a	-1.5797	0.0015
392	O55074	A-kinase anchor protein 7 isoform alpha	Akap7	-1.5712	0.0015
393	Q7TN79	A-kinase anchor protein 7 isoform gamma	Akap7	-1.5712	0.0015
394	P32261	Antithrombin-III	Serpinc1	-1.5688	0.0015
395	Q9CPP6	NADH dehydrogenase [ubiquinone] 1 alpha subcomplex subunit 5	Ndufa5	-1.5688	0.0015
396	Q8K4J6	Myocardin-related transcription factor A	Mrtfa	-1.5600	0.0017
397	P61022	Calcineurin B homologous protein 1	Chp1	-1.5600	0.0017
398	Q8C0D4	Rho GTPase-activating protein 12	Arhgap12	-1.5600	0.0017
399	Q8BGB7	Enolase-phosphatase E1	Enoph1	-1.5600	0.0017
400	Q3TX08	tRNA (guanine(26)-N(2))-dimethyltransferase	Trmt1	-1.5600	0.0017

401	P20491	High affinity immunoglobulin epsilon receptor subunit gamma	Fcer1g	-1.5600	0.0017
402	Q9JHL1	Na(+)/H(+) exchange regulatory cofactor NHE-RF2	Slc9a3r2	-1.5586	0.0017
403	P58059	28S ribosomal protein S21 mitochondrial	Mrps21	-1.5586	0.0017
404	Q6X893	Choline transporter-like protein 1	Slc44a1	-1.5574	0.0017
405	Q62442	Vesicle-associated membrane protein 1	Vamp1	-1.5494	0.0018
406	Q9D6J6	NADH dehydrogenase [ubiquinone] flavoprotein 2 mitochondrial	Ndufv2	-1.5470	0.0018
407	Q9CQW1	Synaptobrevin homolog YKT6	Ykt6	-1.5459	0.0018
408	P42859	Huntingtin	Htt	-1.5413	0.0018
409	G3XA57	Rab11 family-interacting protein 2	Rab11fip2	-1.5381	0.0019
410	Q9CQZ5	NADH dehydrogenase [ubiquinone] 1 alpha subcomplex subunit 6	Ndufa6	-1.5376	0.0019
411	P97315	Cysteine and glycine-rich protein 1	Csrp1	-1.5369	0.0019
412	P13020	Gelsolin	Gsn	-1.5339	0.0019
413	P97333	Neuropilin-1	Nrp1	-1.5333	0.0019
414	Q6ZWU9	40S ribosomal protein S27	Rps27	-1.5328	0.0019
415	Q9JI91	Alpha-actinin-2	Actn2	-1.5326	0.0019
416	P49615	Cyclin-dependent-like kinase 5	Cdk5	-1.5305	0.0019
417	Q8CCN5	BCAS3 microtubule associated cell migration factor	Bcas3	-1.5274	0.0020
418	O35988	Syndecan-4	Sdc4	-1.5220	0.0020
419	Q3ZK22	Vezatin	Vezt	-1.5196	0.0020
420	A2AKG8	Focadhesin	Focad	-1.5196	0.0020
421	Q9EQC5	N-terminal kinase-like protein	Scyl1	-1.5192	0.0020
422	Q8BT60	Copine-3	Cpne3	-1.5160	0.0020
423	Q8BP47	Asparagine--tRNA ligase cytoplasmic	NARS1	-1.5135	0.0020
424	Q61206	Platelet-activating factor acetylhydrolase IB subunit alpha2	Pafah1b2	-1.5111	0.0020
425	P70206	Plexin-A1	Plxna1	-1.5106	0.0020
426	Q8BR63	Protein FAM177A1	Fam177a1	-1.5087	0.0020
427	Q9D1H8	39S ribosomal protein L53 mitochondrial	Mrpl53	-1.5086	0.0020
428	Q9CWW6	Peptidyl-prolyl cis-trans isomerase NIMA-interacting 4	Pin4	-1.5086	0.0020
429	P56382	ATP synthase subunit epsilon mitochondrial	Atp5f1e	-1.5086	0.0020
430	Q9CR27	WASH complex subunit 3	Washc3	-1.5086	0.0020
431	P70335	Rho-associated protein kinase 1	Rock1	-1.5084	0.0020
432	Q9DCS9	NADH dehydrogenase [ubiquinone] 1 beta subcomplex subunit 10	Ndufb10	-1.5071	0.0020
433	Q8BUH8	Sentrin-specific protease 7	Senp7	-1.5059	0.0020
434	Q00623	Apolipoprotein A-I	Apoa1	-1.5055	0.0020
435	Q8VHK5	Membrane protein MLC1	Mlc1	-1.5052	0.0020
436	Q9CPV4	Glyoxalase domain-containing protein 4	Glod4	-1.5042	0.0020
437	Q71LX4	Talin-2	Tln2	-1.4992	0.0021
438	Q3V300	Kinesin-like protein KIF22	Kif22	-1.4981	0.0021
439	G3X9J0	Signal-induced proliferation-associated 1-like protein 3	Sipa1l3	-1.4963	0.0021
440	P35979	60S ribosomal protein L12	Rpl12	-1.4944	0.0021
441	P70398	Probable ubiquitin carboxyl-terminal hydrolase FAF-X	Usp9x	-1.4883	0.0021
442	Q8BFY6	Peflin	Pef1	-1.4808	0.0021
443	Q8VCC9	Spondin-1	Spon1	-1.4795	0.0021
444	Q80W93	Hydrocephalus-inducing protein	Hydin	-1.4791	0.0022
445	Q8R0P4	Mth938 domain-containing protein	Aamdc	-1.4739	0.0022
446	Q9Z239	Phospholemman	Fxyd1	-1.4739	0.0022
447	P58771	Tropomyosin alpha-1 chain	Tpm1	-1.4676	0.0023
448	Q6R891	Neurabin-2	Ppp1r9b	-1.4665	0.0023
449	P24270	Catalase	Cat	-1.4609	0.0023
450	Q80U95	Ubiquitin-protein ligase E3C	Ube3c	-1.4595	0.0023
451	P48771	Cytochrome c oxidase subunit 7A2 mitochondrial	Cox7a2	-1.4564	0.0025
452	P20918	Plasminogen	Plg	-1.4560	0.0025
453	Q9CPT3	N-acylneuraminate-9-phosphatase	Nanp	-1.4560	0.0025
454	O88343	Electrogenic sodium bicarbonate cotransporter 1	Slc4a4	-1.4536	0.0025
455	Q8K215	LYR motif-containing protein 4	Lyrm4	-1.4534	0.0025
456	P70406	Mitochondrial uncoupling protein 2	Ucp2	-1.4534	0.0025
457	P10639	Thioredoxin	Txn	-1.4519	0.0025
458	Q8C5W0	Calmin	Clmn	-1.4470	0.0026
459	Q9WU63	Heme-binding protein 2	Hebp2	-1.4470	0.0026
460	O70404	Vesicle-associated membrane protein 8	Vamp8	-1.4470	0.0026
461	P70158	Acid sphingomyelinase-like phosphodiesterase 3a	Smpdl3a	-1.4470	0.0026
462	P56812	Programmed cell death protein 5	Pdcd5	-1.4470	0.0026
463	P13597	Intercellular adhesion molecule 1	Icam1	-1.4470	0.0026
464	P62700	Protein yippee-like 5	Ypel5	-1.4470	0.0026
465	P45878	Peptidyl-prolyl cis-trans isomerase FKBP2	Fkbp2	-1.4470	0.0026
466	P49935	Pro-cathepsin H	Ctsh	-1.4470	0.0026
467	Q7TMK6	Protein Hook homolog 2	Hook2	-1.4470	0.0026

468	Q505B7	Protein archease	Zbtb8os	-1.4470	0.0026
469	P0C6F1	Dynein axonemal heavy chain 2	Dnah2	-1.4470	0.0026
470	Q9JHW2	Omega-amidase NIT2	Nit2	-1.4386	0.0026
471	Q9DBJ1	Phosphoglycerate mutase 1	Pgam1	-1.4365	0.0027
472	P08551	Neurofilament light polypeptide	Nefl	-1.4364	0.0027
473	Q8C052	Microtubule-associated protein 1S	Map1s	-1.4318	0.0027
474	P68368	Tubulin alpha-4A chain	Tuba4a	-1.4313	0.0027
475	Q08460	Calcium-activated potassium channel subunit alpha-1	Kcnma1	-1.4274	0.0027
476	P02468	Laminin subunit gamma-1	Lamc1	-1.4261	0.0027
477	Q9WVL6	Exostosin-like 3	Extl3	-1.4253	0.0027
478	P53798	Squalene synthase	Fdft1	-1.4222	0.0027
479	Q60625	Intercellular adhesion molecule 5	Icam5	-1.4212	0.0027
480	Q9JHD1	Histone acetyltransferase KAT2B	Kat2b	-1.4188	0.0028
481	Q8VCT9	Dual specificity testis-specific protein kinase 2	Tesk2	-1.4174	0.0028
482	Q6WQJ1	Diacylglycerol lipase-alpha	Dagla	-1.4115	0.0028
483	Q9CQ92	Mitochondrial fission 1 protein	Fis1	-1.4106	0.0028
484	O35250	Exocyst complex component 7	Exoc7	-1.4104	0.0028
485	Q5EG47	5'-AMP-activated protein kinase catalytic subunit alpha-1	Prkaa1	-1.4038	0.0029
486	P59997	Lysine-specific demethylase 2A	Kdm2a	-1.4005	0.0029
487	Q9WV68	Peroxisomal 2 4-dienoyl-CoA reductase [(3E)-enoyl-CoA-producing]	Decr2	-1.4000	0.0029
488	Q9D0W5	Peptidyl-prolyl cis-trans isomerase-like 1	Ppil1	-1.3996	0.0029
489	Q8BWS5	G protein-regulated inducer of neurite outgrowth 3	Gprin3	-1.3989	0.0029
490	Q6P9K9	Neurexin-3	Nrxn3	-1.3952	0.0029
491	Q6NVE8	WD repeat-containing protein 44	Wdr44	-1.3946	0.0029
492	Q9CYG7	Mitochondrial import receptor subunit TOM34	Tomm34	-1.3944	0.0029
493	Q9Z2R6	Protein unc-119 homolog A	Unc119	-1.3943	0.0029
494	Q8R0F8	Acylpyruvase FAHD1 mitochondrial	Fahd1	-1.3943	0.0029
495	P18826	Phosphorylase b kinase regulatory subunit alpha skeletal muscle isoform	Phka1	-1.3887	0.0030
496	P47754	F-actin-capping protein subunit alpha-2	Capza2	-1.3858	0.0030
497	Q80X95	Ras-related GTP-binding protein A	Rraga	-1.3830	0.0030
498	P63017	Heat shock cognate 71 kDa protein	Hspa8	-1.3824	0.0030
499	Q99LS3	Phosphoserine phosphatase	Psph	-1.3819	0.0030
500	Q9D8Y1	Transmembrane protein 126A	Tmem126a	-1.3810	0.0030
501	P97470	Serine/threonine-protein phosphatase 4 catalytic subunit	Ppp4c	-1.3797	0.0031
502	P17182	Alpha-enolase	Eno1	-1.3797	0.0031
503	P62821	Ras-related protein Rab-1A	Rab1A	-1.3795	0.0031
504	Q8BKN5	Gamma-tubulin complex component 5	Tubgcp5	-1.3782	0.0031
505	Q9Z1Z0	General vesicular transport factor p115	Uso1	-1.3765	0.0031
506	O89112	Glutathione S-transferase LANCL1	Lancl1	-1.3757	0.0031
507	Q91Z69	SLIT-ROBO Rho GTPase-activating protein 1	Srgap1	-1.3754	0.0031
508	P09528	Ferritin heavy chain	Fth1	-1.3749	0.0031
509	Q6PH08	ERC protein 2	Erc2	-1.3744	0.0031
510	Q99KC8	von Willebrand factor A domain-containing protein 5A	Vwa5a	-1.3740	0.0031
511	P31648	Sodium- and chloride-dependent GABA transporter 1	Slc6a1	-1.3732	0.0031
512	Q8VH51	RNA-binding protein 39	Rbm39	-1.3716	0.0031
513	Q920P5	Adenylate kinase isoenzyme 5	Ak5	-1.3698	0.0031
514	P61458	Pterin-4-alpha-carbinolamine dehydratase	Pcbd1	-1.3672	0.0031
515	P97855	Ras GTPase-activating protein-binding protein 1	G3bp1	-1.3671	0.0031
516	Q64520	Guanylate kinase	Guk1	-1.3668	0.0031
517	P35123	Ubiquitin carboxyl-terminal hydrolase 4	Usp4	-1.3647	0.0032
518	Q8VE09	Tetratricopeptide repeat protein 39C	Ttc39c	-1.3624	0.0033
519	P47746	Cannabinoid receptor 1	Cnr1	-1.3624	0.0033
520	Q7TNM2	Tripartite motif-containing protein 46	Trim46	-1.3616	0.0033
521	Q9Z2X1	Heterogeneous nuclear ribonucleoprotein F	Hnrnpf	-1.3602	0.0033
522	O35239	Tyrosine-protein phosphatase non-receptor type 9	Ptpn9	-1.3565	0.0033
523	P13707	Glycerol-3-phosphate dehydrogenase [NAD(+)] cytoplasmic	Gpd1	-1.3472	0.0035
524	A2AJA9	Apical junction component 1 homolog	Ajm1	-1.3463	0.0035
525	Q1RLL3	Copine-9	Cpne9	-1.3453	0.0035
526	Q61838	Pregnancy zone protein	Pzp	-1.3446	0.0035
527	P62754	40S ribosomal protein S6	Rps6	-1.3435	0.0035
528	Q9D882	Protein FAM241B	Fam241b	-1.3434	0.0035
529	Q8BGS2	BolA-like protein 2	Bola2	-1.3434	0.0035
530	P62984	Ubiquitin-60S ribosomal protein L40	Uba52	-1.3392	0.0035
531	O70503	Very-long-chain 3-oxoacyl-CoA reductase	Hsd17b12	-1.3391	0.0035
532	P49452	Centromere protein C	Cenpc	-1.3361	0.0036
533	P31650	Sodium- and chloride-dependent GABA transporter 3	Slc6a11	-1.3338	0.0036

534	Q9Z224	Molybdopterin synthase sulfur carrier subunit	Mocs2	-1.3335	0.0036
535	Q9CQQ0	Small integral membrane protein 8	Smim8	-1.3335	0.0036
536	Q80YA3	Phospholipase DDHD1	Ddhd1	-1.3319	0.0036
537	Q8BZA9	Fructose-2 6-bisphosphatase TIGAR	Tigar	-1.3319	0.0036
538	Q8BTU1	Cilia- and flagella-associated protein 20	Cfap20	-1.3313	0.0036
539	P55200	Histone-lysine N-methyltransferase 2A	Kmt2a	-1.3312	0.0036
540	Q7TMC8	L-fucose kinase	Fcsk	-1.3294	0.0036
541	Q9CR30	Josephin-2	Josd2	-1.3254	0.0037
542	P20357	Microtubule-associated protein 2	Map2	-1.3252	0.0037
543	Q8BH59	Calcium-binding mitochondrial carrier protein Aralar1	Slc25a12	-1.3210	0.0038
544	Q9CTY5	Calcium uptake protein 3 mitochondrial	Micu3	-1.3201	0.0038
545	P56371	Ras-related protein Rab-4A	Rab4a	-1.3193	0.0038
546	Q3UMR5	Calcium uniporter protein mitochondrial	Mcu	-1.3176	0.0038
547	Q5NC83	Spermatogenesis-associated protein 48	Spata48	-1.3172	0.0038
548	Q8BYB9	Protein O-glucosyltransferase 1	Poglut1	-1.3170	0.0038
549	Q8CCJ4	APC membrane recruitment protein 2	Amer2	-1.3170	0.0038
550	Q60857	Sodium-dependent serotonin transporter	Slc6a4	-1.3170	0.0038
551	Q80VJ3	2'-deoxynucleoside 5'-phosphate N-hydrolase 1	Dnph1	-1.3170	0.0038
552	Q9D0K0	TBC1 domain family member 7	Tbc1d7	-1.3170	0.0038
553	Q9QYI4	DnaJ homolog subfamily B member 12	Dnajb12	-1.3170	0.0038
554	P11862	Growth arrest-specific protein 2	Gas2	-1.3170	0.0038
555	Q9CXV9	DCN1-like protein 5	Dcun1d5	-1.3170	0.0038
556	Q9DCL2	Cytosolic iron-sulfur assembly component 2A	Ciao2a	-1.3170	0.0038
557	Q91W53	Golgin subfamily A member 7	Golga7	-1.3170	0.0038
558	Q8VHZ7	U3 small nucleolar ribonucleoprotein protein IMP4	Imp4	-1.3170	0.0038
559	Q3UNA4	NTF2-related export protein 2	Nxt2	-1.3170	0.0038
560	Q8C460	ERI1 exoribonuclease 3	Eri3	-1.3160	0.0038
561	Q9WVQ1	Membrane-associated guanylate kinase WW and PDZ domain-containing protein 2	Magi2	-1.3141	0.0038
562	Q8CCB4	Vacuolar protein sorting-associated protein 53 homolog	Vps53	-1.3114	0.0039
563	O70496	H(+)/Cl(-) exchange transporter 7	Clcn7	-1.3080	0.0039
564	Q925N0	Sideroflexin-5	Sfxn5	-1.3060	0.0040
565	Q61809	Leucine-rich repeat neuronal protein 1	Lrrn1	-1.3051	0.0040
566	Q7M759	Alpha/beta hydrolase domain-containing protein 17B	Abhd17b	-1.3051	0.0040
567	Q3UHD9	Arf-GAP with GTPase ANK repeat and PH domain-containing protein 2	Agap2	-1.3015	0.0040
568	Q91WG2	Rab GTPase-binding effector protein 2	Rabep2	-1.2983	0.0050
569	P21271	Unconventional myosin-Vb	Myo5b	-1.2982	0.0050
570	Q921M4	Golgin subfamily A member 2	Golga2	-1.2978	0.0050
571	Q8R2Y8	Peptidyl-tRNA hydrolase 2 mitochondrial	Ptrh2	-1.2978	0.0050
572	P02463	Collagen alpha-1(IV) chain	Col4a1	-1.2978	0.0050
573	Q9D0T1	NHP2-like protein 1	Snu13	-1.2978	0.0050
574	Q9WVA2	Mitochondrial import inner membrane translocase subunit Tim8 A	Timm8a1	-1.2978	0.0050
575	O55102	Biogenesis of lysosome-related organelles complex 1 subunit 1	Bloc1s1	-1.2978	0.0050
576	Q9QZD8	Mitochondrial dicarboxylate carrier	Slc25a10	-1.2967	0.0050
577	Q61885	Myelin-oligodendrocyte glycoprotein	Mog	-1.2954	0.0051
578	Q99L45	Eukaryotic translation initiation factor 2 subunit 2	Eif2s2	-1.2944	0.0051
579	P98086	Complement C1q subcomponent subunit A	C1qa	-1.2944	0.0051
580	Q9CXZ1	NADH dehydrogenase [ubiquinone] iron-sulfur protein 4 mitochondrial	Ndufs4	-1.2944	0.0051
581	O70133	ATP-dependent RNA helicase A	Dhx9	-1.2935	0.0051
582	Q8C963	Coiled-coil domain-containing protein 159	Ccdc159	-1.2919	0.0051
583	Q9CXS4	Centromere protein V	Cenpv	-1.2898	0.0051
584	Q9QUJ7	Long-chain-fatty-acid--CoA ligase 4	Acsl4	-1.2863	0.0051
585	P28660	Nck-associated protein 1	Nckap1	-1.2862	0.0052
586	Q99LC8	Translation initiation factor eIF-2B subunit alpha	Eif2b1	-1.2836	0.0052
587	Q3UZP4	Small VCP/p97-interacting protein	Svip	-1.2836	0.0052
588	G5E829	Plasma membrane calcium-transporting ATPase 1	Atp2b1	-1.2824	0.0052
589	Q07813	Apoptosis regulator BAX	Bax	-1.2789	0.0053
590	Q3UQ44	Ras GTPase-activating-like protein IQGAP2	Iqgap2	-1.2757	0.0053
591	Q8BTY2	Sodium bicarbonate cotransporter 3	Slc4a7	-1.2754	0.0053
592	Q99K10	Neuroligin-1	Nlgn1	-1.2754	0.0053
593	B1AQJ2	Ubiquitin carboxyl-terminal hydrolase 36	Usp36	-1.2750	0.0053
594	Q9R1P4	Proteasome subunit alpha type-1	Psma1	-1.2744	0.0053
595	P12970	60S ribosomal protein L7a	Rpl7a	-1.2701	0.0054
596	Q8BJY1	26S proteasome non-ATPase regulatory subunit 5	Psmd5	-1.2701	0.0054
597	P0CG50	Polyubiquitin-C	Ubc	-1.2698	0.0055
598	P0CG49	Polyubiquitin-B	Ubb	-1.2698	0.0055
599	P47743	Metabotropic glutamate receptor 8	Grm8	-1.2672	0.0055

600	Q3UUI3	Acyl-coenzyme A thioesterase THEM4	Them4	-1.2665	0.0055
601	Q3UHK1	Proton myo-inositol cotransporter	Slc2a13	-1.2665	0.0055
602	Q3UPL0	Protein transport protein Sec31A	Sec31a	-1.2642	0.0056
603	P0C673	Immunoglobulin superfamily member 11	Igsf11	-1.2635	0.0056
604	P62315	Small nuclear ribonucleoprotein Sm D1	Snrpd1	-1.2635	0.0056
605	Q5U4C1	G-protein coupled receptor-associated sorting protein 1	Gprasp1	-1.2635	0.0056
606	Q9JK38	Glucosamine 6-phosphate N-acetyltransferase	Gnpnat1	-1.2635	0.0056
607	Q91YR9	Prostaglandin reductase 1	Ptgr1	-1.2635	0.0056
608	Q91ZW2	GDP-fucose protein O-fucosyltransferase 1	Pofut1	-1.2635	0.0056
609	Q8K245	UV radiation resistance-associated protein	Uvrag	-1.2635	0.0056
610	P28828	Receptor-type tyrosine-protein phosphatase mu	Ptprm	-1.2635	0.0056
611	Q61127	NGFI-A-binding protein 2	Nab2	-1.2635	0.0056
612	Q9WTS4	Teneurin-1	Tenm1	-1.2634	0.0056
613	Q920N7	Synaptotagmin-12	Syt12	-1.2575	0.0057
614	Q62393	Tumor protein D52	Tpd52	-1.2547	0.0057
615	Q80Y56	Rabenosyn-5	Rbsn	-1.2522	0.0058
616	Q9JKR6	Hypoxia up-regulated protein 1	Hyou1	-1.2485	0.0058
617	Q9JKC6	Cell cycle exit and neuronal differentiation protein 1	Cend1	-1.2483	0.0058
618	Q8BW96	Calcium/calmodulin-dependent protein kinase type 1D	Camk1d	-1.2461	0.0059
619	Q8VDN2	Sodium/potassium-transporting ATPase subunit alpha-1	Atp1a1	-1.2460	0.0059
620	P42932	T-complex protein 1 subunit theta	Cct8	-1.2452	0.0059
621	Q61771	Kinesin-like protein KIF3B	Kif3b	-1.2451	0.0059
622	Q9DCT2	NADH dehydrogenase [ubiquinone] iron-sulfur protein 3 mitochondrial	Ndufs3	-1.2406	0.0060
623	P62983	Ubiquitin-40S ribosomal protein S27a	Rps27a	-1.2378	0.0061
624	Q60870	Receptor expression-enhancing protein 5	Reep5	-1.2358	0.0061
625	Q91W86	Vacuolar protein sorting-associated protein 11 homolog	Vps11	-1.2325	0.0061
626	Q91ZZ3	Beta-synuclein	Sncb	-1.2325	0.0061
627	Q8C2E7	WASH complex subunit 5	Washc5	-1.2324	0.0061
628	Q7TME0	2-lysophosphatidate phosphatase PLPPR4	Plppr4	-1.2300	0.0061
629	P08226	Apolipoprotein E	Apoe	-1.2294	0.0062
630	Q8BTH8	Casein kinase I isoform gamma-1	Csnk1g1	-1.2269	0.0062
631	O70152	Dolichol-phosphate mannosyltransferase subunit 1	Dpm1	-1.2269	0.0062
632	Q5SWU9	Acetyl-CoA carboxylase 1	Acaca	-1.2265	0.0062
633	Q8BH61	Coagulation factor XIII A chain	F13a1	-1.2235	0.0062
634	O88384	Vesicle transport through interaction with t-SNAREs homolog 1B	Vti1b	-1.2235	0.0062
635	Q99J99	3-mercaptopyruvate sulfurtransferase	Mpst	-1.2235	0.0062
636	Q80UP3	Diacylglycerol kinase zeta	Dgkz	-1.2229	0.0063
637	P63213	Guanine nucleotide-binding protein G(I)/G(S)/G(O) subunit gamma-2	Gng2	-1.2217	0.0063
638	P62075	Mitochondrial import inner membrane translocase subunit Tim13	Timm13	-1.2215	0.0063
639	Q9DCJ5	NADH dehydrogenase [ubiquinone] 1 alpha subcomplex subunit 8	Ndufa8	-1.2178	0.0064
640	Q9Z351	Potassium voltage-gated channel subfamily KQT member 2	Kcnq2	-1.2137	0.0064
641	Q504M8	Ras-related protein Rab-26	Rab26	-1.2094	0.0067
642	P61957	Small ubiquitin-related modifier 2	Sumo2	-1.2079	0.0067
643	Q9Z172	Small ubiquitin-related modifier 3	Sumo3	-1.2079	0.0067
644	Q61282	Aggrecan core protein	Acan	-1.2078	0.0067
645	Q9WVA4	Transgelin-2	Tagln2	-1.2066	0.0067
646	Q8BG40	Katanin p80 WD40 repeat-containing subunit B1	Katnb1	-1.2057	0.0067
647	Q6P9R4	Rho guanine nucleotide exchange factor 18	Arhgef18	-1.2028	0.0068
648	O35215	D-dopachrome decarboxylase	Ddt	-1.2028	0.0068
649	Q61510	E3 ubiquitin/ISG15 ligase TRIM25	Trim25	-1.2028	0.0068
650	Q9Z103	Activity-dependent neuroprotector homeobox protein	Adnp	-1.2028	0.0068
651	P11103	Poly [ADP-ribose] polymerase 1	Parp1	-1.2026	0.0068
652	P53994	Ras-related protein Rab-2A	Rab2a	-1.2019	0.0068
653	Q60631	Growth factor receptor-bound protein 2	Grb2	-1.2016	0.0068
654	Q924T2	28S ribosomal protein S2 mitochondrial	Mrps2	-1.2015	0.0068
655	Q8BI72	CDKN2A-interacting protein	Cdkn2aip	-1.2015	0.0068
656	Q9CQ74	Leptin receptor overlapping transcript-like 1	Leprotl1	-1.2015	0.0068
657	P10605	Cathepsin B	Ctsb	-1.2009	0.0068
658	Q9CPY7	Cytosol aminopeptidase	Lap3	-1.2001	0.0068
659	Q8C129	Leucyl-cystinyl aminopeptidase	Lnpep	-1.1984	0.0069
660	P97371	Proteasome activator complex subunit 1	Psme1	-1.1984	0.0069
661	Q9EPR5	VPS10 domain-containing receptor SorCS2	Sorcs2	-1.1977	0.0069
662	P80318	T-complex protein 1 subunit gamma	Cct3	-1.1975	0.0069
663	Q9CQ75	NADH dehydrogenase [ubiquinone] 1 alpha subcomplex subunit 2	Ndufa2	-1.1974	0.0069
664	O35963	Ras-related protein Rab-33B	Rab33b	-1.1957	0.0069
665	Q9D338	39S ribosomal protein L19 mitochondrial	Mrpl19	-1.1953	0.0069
666	Q9D1J1	Adaptin ear-binding coat-associated protein 2	Necap2	-1.1953	0.0069

667	Q6Y685	Transforming acidic coiled-coil-containing protein 1	Tacc1	-1.1953	0.0069
668	Q3URD3	Sarcolemmal membrane-associated protein	Slmap	-1.1946	0.0069
669	Q69ZQ1	Myogenesis-regulating glycosidase	Myorg	-1.1946	0.0069
670	Q8VBX6	Multiple PDZ domain protein	Mpdz	-1.1946	0.0069
671	Q5SYL3	Protein KIAA0100	Kiaa0100	-1.1946	0.0069
672	P97363	Serine palmitoyltransferase 2	Sptlc2	-1.1946	0.0069
673	P10493	Nidogen-1	Nid1	-1.1930	0.0070
674	Q99NB9	Splicing factor 3B subunit 1	Sf3b1	-1.1915	0.0070
675	Q9CQ00	Distal membrane-arm assembly complex protein 1	Dmac1	-1.1903	0.0070
676	Q91WG7	Diacylglycerol kinase gamma	Dgkg	-1.1901	0.0070
677	Q8K0T0	Reticulon-1	Rtn1	-1.1900	0.0070
678	Q9ESE1	Lipopolysaccharide-responsive and beige-like anchor protein	Lrba	-1.1882	0.0071
679	Q9CR68	Cytochrome b-c1 complex subunit Rieske mitochondrial	Uqcrfs1	-1.1851	0.0074
680	Q8BG32	26S proteasome non-ATPase regulatory subunit 11	Psmd11	-1.1828	0.0074
681	Q9QXT0	Protein canopy homolog 2	Cnpy2	-1.1828	0.0074
682	Q5RKR3	Immunoglobulin superfamily containing leucine-rich repeat protein 2	Islr2	-1.1784	0.0075
683	P97447	Four and a half LIM domains protein 1	Fhl1	-1.1755	0.0076
684	P00405	Cytochrome c oxidase subunit 2	Mtco2	-1.1751	0.0076
685	P62852	40S ribosomal protein S25	Rps25	-1.1659	0.0086
686	O08583	THO complex subunit 4	Alyref	-1.1640	0.0087
687	Q9JJW6	Aly/REF export factor 2	Alyref2	-1.1640	0.0087
688	P28652	Calcium/calmodulin-dependent protein kinase type II subunit beta	Camk2b	-1.1639	0.0087
689	Q3UHK6	Teneurin-4	Tenm4	-1.1631	0.0087
690	Q9D6M3	Mitochondrial glutamate carrier 1	Slc25a22	-1.1628	0.0087
691	P70302	Stromal interaction molecule 1	Stim1	-1.1627	0.0087
692	Q3UFF7	Lysophospholipase-like protein 1	Lyplal1	-1.1627	0.0087
693	Q61285	ATP-binding cassette sub-family D member 2	Abcd2	-1.1627	0.0087
694	Q61290	Voltage-dependent R-type calcium channel subunit alpha-1E	Cacna1e	-1.1624	0.0087
695	Q5RJI5	Serine/threonine-protein kinase BRSK1	Brsk1	-1.1574	0.0089
696	Q78IK4	MIC complex subunit Mic27	Apool	-1.1574	0.0089
697	P50171	(3R)-3-hydroxyacyl-CoA dehydrogenase	Hsd17b8	-1.1574	0.0089
698	Q60866	Phosphotriesterase-related protein	Pter	-1.1559	0.0089
699	Q9EPL2	Calsyntenin-1	Clstn1	-1.1545	0.0090
700	P49722	Proteasome subunit alpha type-2	Psma2	-1.1505	0.0090
701	P08071	Lactotransferrin	Ltf	-1.1505	0.0090
702	Q9D7X3	Dual specificity protein phosphatase 3	Dusp3	-1.1486	0.0091
703	P14847	C-reactive protein	Crp	-1.1486	0.0091
704	Q9D9Z5	DET1- and DDB1-associated protein 1	Dda1	-1.1486	0.0091
705	P13808	Anion exchange protein 2	Slc4a2	-1.1486	0.0091
706	Q3UCQ1	Forkhead box protein K2	Foxk2	-1.1486	0.0091
707	P01639	Ig kappa chain V-V region MOPC 41	Gm5571	-1.1486	0.0091
708	Q9JM14	5'(3')-deoxyribonucleotidase cytosolic type	Nt5c	-1.1486	0.0091
709	Q8BZJ7	DCN1-like protein 2	Dcun1d2	-1.1486	0.0091
710	P01786	Ig heavy chain V region MOPC 47A		-1.1486	0.0091
711	Q9DCB8	Iron-sulfur cluster assembly 2 homolog mitochondrial	Isca2	-1.1486	0.0091
712	Q8BQZ5	Cleavage and polyadenylation specificity factor subunit 4	Cpsf4	-1.1486	0.0091
713	Q5SW75	Protein phosphatase Slingshot homolog 2	Ssh2	-1.1486	0.0091
714	Q9QUQ5	Short transient receptor potential channel 4	Trpc4	-1.1486	0.0091
715	P62073	Mitochondrial import inner membrane translocase subunit Tim10	Timm10	-1.1486	0.0091
716	Q9D1I5	Methylmalonyl-CoA epimerase mitochondrial	Mcee	-1.1486	0.0091
717	P62965	Cellular retinoic acid-binding protein 1	Crabp1	-1.1486	0.0091
718	E9PYK3	Protein mono-ADP-ribosyltransferase PARP4	Parp4	-1.1486	0.0091
719	A6X919	Probable C-mannosyltransferase DPY19L1	Dpy19l1	-1.1486	0.0091
720	O70200	Allograft inflammatory factor 1	Aif1	-1.1486	0.0091
721	Q9CR64	Protein kish-A	Tmem167a	-1.1486	0.0091
722	P61014	Cardiac phospholamban	Pln	-1.1486	0.0091
723	Q9CZ83	39S ribosomal protein L55 mitochondrial	Mrpl55	-1.1486	0.0091
724	Q61743	ATP-sensitive inward rectifier potassium channel 11	Kcnj11	-1.1486	0.0091
725	Q8VE42	Ankyrin repeat domain-containing protein 49	Ankrd49	-1.1486	0.0091
726	Q9EP69	Phosphatidylinositol-3-phosphatase SAC1	Sacm1l	-1.1480	0.0091
727	O08992	Syntenin-1	Sdcbp	-1.1470	0.0092
728	Q9CQA3	Succinate dehydrogenase [ubiquinone] iron-sulfur subunit mitochondrial	Sdhb	-1.1428	0.0093
729	Q8BLN5	Lanosterol synthase	Lss	-1.1425	0.0093
730	Q3UMF0	Cordon-bleu protein-like 1	Cobll1	-1.1412	0.0093
731	Q80TF3	Protocadherin-19	Pcdh19	-1.1412	0.0093
732	P97427	Dihydropyrimidinase-related protein 1	Crmp1	-1.1410	0.0093

733	E9PVB3	Coiled-coil domain-containing protein 175	Ccdc175	-1.1409	0.0094
734	Q8R4U7	Leucine zipper protein 1	Luzp1	-1.1408	0.0094
735	P11031	Activated RNA polymerase II transcriptional coactivator p15	Sub1	-1.1404	0.0094
736	Q9D1G5	Leucine-rich repeat-containing protein 57	Lrrc57	-1.1403	0.0094
737	E9Q557	Desmoplakin	Dsp	-1.1393	0.0095
738	P61027	Ras-related protein Rab-10	Rab10	-1.1377	0.0095
739	P46638	Ras-related protein Rab-11B	Rab11b	-1.1377	0.0095
740	Q9DB20	ATP synthase subunit O mitochondrial	Atp5po	-1.1342	0.0095
741	B1AXH1	NHS-like protein 2	Nhsl2	-1.1301	0.0096
742	Q9CR62	Mitochondrial 2-oxoglutarate/malate carrier protein	Slc25a11	-1.1299	0.0096
743	P70399	TP53-binding protein 1	Tp53bp1	-1.1260	0.0097
744	P07724	Albumin	Alb	-1.1236	0.0099
745	Q9D9V3	Ethylmalonyl-CoA decarboxylase	Echdc1	-1.1210	0.0101
746	P58774	Tropomyosin beta chain	Tpm2	-1.1182	0.0102
747	Q80XK6	Autophagy-related protein 2 homolog B	Atg2b	-1.1173	0.0102
748	Q6NS52	Diacylglycerol kinase beta	Dgkb	-1.1170	0.0102
749	Q9WTU6	Mitogen-activated protein kinase 9	Mapk9	-1.1157	0.0103
750	P83887	Tubulin gamma-1 chain	Tubg1	-1.1153	0.0103
751	Q02384	Son of sevenless homolog 2	Sos2	-1.1153	0.0103
752	P11835	Integrin beta-2	Itgb2	-1.1151	0.0103
753	Q505D1	Serine/threonine-protein phosphatase 6 regulatory ankyrin repeat subunit A	Ankrd28	-1.1151	0.0103
754	P70313	Nitric oxide synthase endothelial	Nos3	-1.1151	0.0103
755	Q8BGX2	Mitochondrial import inner membrane translocase subunit Tim29	Timm29	-1.1135	0.0103
756	Q6WVG3	BTB/POZ domain-containing protein KCTD12	Kctd12	-1.1117	0.0104
757	Q8VBV7	COP9 signalosome complex subunit 8	Cops8	-1.1117	0.0104
758	F6ZDS4	Nucleoprotein TPR	Tpr	-1.1113	0.0104
759	P98192	Dihydroxyacetone phosphate acyltransferase	Gnpat	-1.1089	0.0105
760	Q8BR90	UPF0600 protein C5orf51 homolog		-1.1089	0.0105
761	Q9DC53	Copine-8	Cpne8	-1.1084	0.0105
762	P61750	ADP-ribosylation factor 4	Arf4	-1.1060	0.0106
763	A2AN08	E3 ubiquitin-protein ligase UBR4	Ubr4	-1.1055	0.0106
764	P09671	Superoxide dismutase [Mn] mitochondrial	Sod2	-1.1052	0.0106
765	Q91ZR1	Ras-related protein Rab-4B	Rab4b	-1.1021	0.0121
766	Q8CAQ8	MIC complex subunit Mic60	Immt	-1.1020	0.0121
767	P61021	Ras-related protein Rab-5B	Rab5b	-1.1020	0.0121
768	Q8BR92	Paralemmin-2	Palm2	-1.1018	0.0122
769	Q8BGN8	Synaptoporin	Synpr	-1.1007	0.0122
770	Q9QYS2	Metabotropic glutamate receptor 3	Grm3	-1.1001	0.0122
771	Q80VP0	Tectonin beta-propeller repeat-containing protein 1	Tecpr1	-1.0982	0.0124
772	Q9JIK9	28S ribosomal protein S34 mitochondrial	Mrps34	-1.0975	0.0124
773	Q78PY7	Staphylococcal nuclease domain-containing protein 1	Snd1	-1.0968	0.0124
774	Q6S7F2	Transcription factor E2F7	E2f7	-1.0965	0.0125
775	Q8R3F5	Malonyl-CoA-acyl carrier protein transacylase mitochondrial	Mcat	-1.0961	0.0125
776	Q9QYB8	Beta-adducin	Add2	-1.0951	0.0126
777	Q6VNS1	NT-3 growth factor receptor	Ntrk3	-1.0945	0.0126
778	P62245	40S ribosomal protein S15a	Rps15a	-1.0945	0.0126
779	Q9WUU9	Germinal-center associated nuclear protein	Mcm3ap	-1.0941	0.0126
780	Q02013	Aquaporin-1	Aqp1	-1.0941	0.0126
781	Q6NTA4	Ras-related GTP-binding protein B	Rragb	-1.0929	0.0126
782	Q8BU30	Isoleucine--tRNA ligase cytoplasmic	Iars1	-1.0927	0.0126
783	O08638	Myosin-11	Myh11	-1.0922	0.0127
784	Q8CI94	Glycogen phosphorylase brain form	Pygb	-1.0910	0.0127
785	Q9D0M3	Cytochrome c1 heme protein mitochondrial	Cyc1	-1.0891	0.0127
786	P22682	E3 ubiquitin-protein ligase CBL	Cbl	-1.0887	0.0127
787	Q9ERI6	Retinol dehydrogenase 14	Rdh14	-1.0883	0.0127
788	Q9CQ19	Myosin regulatory light polypeptide 9	Myl9	-1.0883	0.0127
789	Q6P069	Sorcin	Sri	-1.0879	0.0128
790	Q6PHN9	Ras-related protein Rab-35	Rab35	-1.0866	0.0128
791	Q68ED7	CREB-regulated transcription coactivator 1	Crtc1	-1.0853	0.0129
792	G5E870	E3 ubiquitin-protein ligase TRIP12	Trip12	-1.0853	0.0129
793	P54797	Transport and Golgi organization 2 homolog	Tango2	-1.0853	0.0129
794	Q99MU3	Double-stranded RNA-specific adenosine deaminase	Adar	-1.0847	0.0129
795	P62484	Abl interactor 2	Abi2	-1.0818	0.0130
796	Q9QZF2	Glypican-1	Gpc1	-1.0810	0.0130
797	Q9CQE8	RNA transcription translation and transport factor protein	RTRAF	-1.0794	0.0130
798	Q9CQJ8	NADH dehydrogenase [ubiquinone] 1 beta subcomplex subunit 9	Ndufb9	-1.0769	0.0131
799	Q9WVQ5	Methylthioribulose-1-phosphate dehydratase	Apip	-1.0755	0.0132

800	Q8K2I4	Beta-mannosidase	Manba	-1.0755	0.0132
801	Q61599	Rho GDP-dissociation inhibitor 2	Arhgdib	-1.0755	0.0132
802	Q3U1F9	Phosphoprotein associated with glycosphingolipid-enriched microdomains 1	Pag1	-1.0755	0.0132
803	Q04519	Sphingomyelin phosphodiesterase	Smpd1	-1.0755	0.0132
804	O88745	Scrapie-responsive protein 1	Scrg1	-1.0755	0.0132
805	Q9JJI8	60S ribosomal protein L38	Rpl38	-1.0755	0.0132
806	P84089	Enhancer of rudimentary homolog	Erh	-1.0755	0.0132
807	Q9EPV8	Ubiquitin-like protein 5	Ubl5	-1.0755	0.0132
808	P01887	Beta-2-microglobulin	B2m	-1.0755	0.0132
809	O70194	Eukaryotic translation initiation factor 3 subunit D	Eif3d	-1.0739	0.0132
810	Q9JIG8	PRA1 family protein 2	Praf2	-1.0739	0.0132
811	P34022	Ran-specific GTPase-activating protein	Ranbp1	-1.0731	0.0132
812	Q9QYE9	Pleckstrin homology domain-containing family B member 1	Plekhb1	-1.0707	0.0133
813	O54950	5'-AMP-activated protein kinase subunit gamma-1	Prkag1	-1.0680	0.0134
814	Q8BRF7	Sec1 family domain-containing protein 1	Scfd1	-1.0668	0.0135
815	Q9DBT5	AMP deaminase 2	Ampd2	-1.0657	0.0135
816	Q8BSL7	ADP-ribosylation factor 2	Arf2	-1.0600	0.0138
817	P14069	Protein S100-A6	S100a6	-1.0580	0.0138
818	Q9QYC0	Alpha-adducin	Add1	-1.0572	0.0138
819	Q9QZ23	NFU1 iron-sulfur cluster scaffold homolog mitochondrial	Nfu1	-1.0557	0.0139
820	P03930	ATP synthase protein 8	Mtatp8	-1.0557	0.0139
821	P63168	Dynein light chain 1 cytoplasmic	Dynll1	-1.0556	0.0139
822	P25785	Metalloproteinase inhibitor 2	Timp2	-1.0556	0.0139
823	Q8VEB6	Zinc phosphodiesterase ELAC protein 1	Elac1	-1.0556	0.0139
824	P53808	Phosphatidylcholine transfer protein	Pctp	-1.0556	0.0139
825	Q91WN1	DnaJ homolog subfamily C member 9	Dnajc9	-1.0556	0.0139
826	Q9CQH7	Transcription factor BTF3 homolog 4	Btf3l4	-1.0556	0.0139
827	Q3V4B5	COMM domain-containing protein 6	Commd6	-1.0556	0.0139
828	Q99LY9	NADH dehydrogenase [ubiquinone] iron-sulfur protein 5	Ndufs5	-1.0556	0.0139
829	Q9D7B7	Probable glutathione peroxidase 8	Gpx8	-1.0556	0.0139
830	P58064	28S ribosomal protein S6 mitochondrial	Mrps6	-1.0556	0.0139
831	Q8BTE5	Protein CEBPZ	Cebpzos	-1.0556	0.0139
832	Q5DU37	Zinc finger FYVE domain-containing protein 26	Zfyve26	-1.0556	0.0139
833	Q7TPG7	Chemokine-like protein TAFA-2	Tafa2	-1.0556	0.0139
834	Q8K2A1	PTB domain-containing engulfment adapter protein 1	Gulp1	-1.0556	0.0139
835	Q0VBK2	Keratin type II cytoskeletal 80	Krt80	-1.0556	0.0139
836	P56873	Protein ZNRD2	Znrd2	-1.0556	0.0139
837	P11881	Inositol 1 4 5-trisphosphate receptor type 1	Itpr1	-1.0551	0.0139
838	P97449	Aminopeptidase N	Anpep	-1.0549	0.0140
839	Q9WTU3	Sodium channel protein type 8 subunit alpha	Scn8a	-1.0546	0.0140
840	P97742	Carnitine O-palmitoyltransferase 1 liver isoform	Cpt1a	-1.0546	0.0140
841	Q8CIZ8	von Willebrand factor	Vwf	-1.0546	0.0140
842	Q60932	Voltage-dependent anion-selective channel protein 1	Vdac1	-1.0546	0.0140
843	P32020	Sterol carrier protein 2	Scp2	-1.0524	0.0140
844	Q91VR2	ATP synthase subunit gamma mitochondrial	Atp5f1c	-1.0523	0.0141
845	P00493	Hypoxanthine-guanine phosphoribosyltransferase	Hprt1	-1.0511	0.0141
846	Q9Z218	Dipeptidyl aminopeptidase-like protein 6	Dpp6	-1.0505	0.0142
847	Q3UHB1	5'-nucleotidase domain-containing protein 3	Nt5dc3	-1.0484	0.0144
848	Q8BXR9	Oxysterol-binding protein-related protein 6	Osbpl6	-1.0478	0.0145
849	Q9JK23	Proteasome assembly chaperone 1	Psmg1	-1.0478	0.0145
850	Q9QZ06	Toll-interacting protein	Tollip	-1.0478	0.0145
851	P83093	Stromal interaction molecule 2	Stim2	-1.0477	0.0145
852	Q6PER3	Microtubule-associated protein RP/EB family member 3	Mapre3	-1.0471	0.0145
853	Q7TQF7	Amphiphysin	Amph	-1.0467	0.0146
854	P30999	Catenin delta-1	Ctnnd1	-1.0446	0.0146
855	Q810M5	Palmitoyltransferase ZDHHC19	Zdhhc19	-1.0441	0.0146
856	Q3TKT4	Transcription activator BRG1	Smarca4	-1.0439	0.0147
857	Q8K0C1	Importin-13	Ipo13	-1.0425	0.0147
858	Q8BGR2	Volume-regulated anion channel subunit LRRC8D	Lrrc8d	-1.0425	0.0147
859	Q8CHQ0	F-box only protein 4	Fbxo4	-1.0425	0.0147
860	Q80YV2	Nuclear-interacting partner of ALK	Zc3hc1	-1.0421	0.0147
861	Q64378	Peptidyl-prolyl cis-trans isomerase FKBP5	Fkbp5	-1.0421	0.0147
862	O35445	E3 ubiquitin-protein ligase RNF5	Rnf5	-1.0421	0.0147
863	Q91YY4	ATP synthase mitochondrial F1 complex assembly factor 2	Atpaf2	-1.0421	0.0147
864	Q8BWH0	Putative sodium-coupled neutral amino acid transporter 7	Slc38a7	-1.0421	0.0147
865	Q3UVL4	Vacuolar protein sorting-associated protein 51 homolog	Vps51	-1.0420	0.0147
866	P17225	Polypyrimidine tract-binding protein 1	Ptbp1	-1.0408	0.0149

867	Q9QXY6	EH domain-containing protein 3	Ehd3	-1.0372	0.0150
868	Q8BYM5	Neuroligin-3	Nlgn3	-1.0365	0.0151
869	O70435	Proteasome subunit alpha type-3	Psma3	-1.0333	0.0152
870	Q61207	Prosaposin	Psap	-1.0312	0.0152
871	P50518	V-type proton ATPase subunit E 1	Atp6v1e1	-1.0310	0.0152
872	Q9CR51	V-type proton ATPase subunit G 1	Atp6v1g1	-1.0310	0.0152
873	Q99K46	Ubiquitin carboxyl-terminal hydrolase 11	Usp11	-1.0305	0.0152
874	P27048	Small nuclear ribonucleoprotein-associated protein B	Snrpb	-1.0305	0.0152
875	P63163	Small nuclear ribonucleoprotein-associated protein N	Snrpn	-1.0305	0.0152
876	Q61765	Keratin type I cuticular Ha1	Krt31	-1.0305	0.0152
877	Q3UV17	Keratin type II cytoskeletal 2 oral	Krt76	-1.0303	0.0152
878	P68404	Protein kinase C beta type	Prkcb	-1.0276	0.0153
879	P59281	Rho GTPase-activating protein 39	Arhgap39	-1.0258	0.0156
880	P41105	60S ribosomal protein L28	Rpl28	-1.0257	0.0156
881	P23819	Glutamate receptor 2	Gria2	-1.0253	0.0156
882	Q9CRC9	Glucosamine-6-phosphate isomerase 2	Gnpda2	-1.0252	0.0156
883	Q9CPU0	Lactoylglutathione lyase	Glo1	-1.0246	0.0156
884	P56135	ATP synthase subunit f mitochondrial	Atp5mf	-1.0244	0.0156
885	P62900	60S ribosomal protein L31	Rpl31	-1.0238	0.0156
886	Q61941	NAD(P) transhydrogenase mitochondrial	Nnt	-1.0230	0.0158
887	Q64332	Synapsin-2	Syn2	-1.0215	0.0159
888	Q3UU96	Serine/threonine-protein kinase MRCK alpha	Cdc42bpa	-1.0212	0.0159
889	Q61753	D-3-phosphoglycerate dehydrogenase	Phgdh	-1.0204	0.0159
890	Q80YD1	ATP-dependent RNA helicase SUPV3L1 mitochondrial	Supv3l1	-1.0195	0.0160
891	P62137	Serine/threonine-protein phosphatase PP1-alpha catalytic subunit	Ppp1ca	-1.0194	0.0160
892	P60487	Chronophin	Pdxp	-1.0187	0.0160
893	Q9R112	Sulfide:quinone oxidoreductase mitochondrial	Sqor	-1.0161	0.0163
894	Q7TPS5	C2 domain-containing protein 5	C2cd5	-1.0161	0.0163
895	Q8CHG7	Rap guanine nucleotide exchange factor 2	Rapgef2	-1.0154	0.0163
896	P47963	60S ribosomal protein L13	Rpl13	-1.0151	0.0163
897	Q6PFD5	Disks large-associated protein 3	Dlgap3	-1.0132	0.0164
898	Q80VL1	Tudor and KH domain-containing protein	Tdrkh	-1.0123	0.0165
899	P08032	Spectrin alpha chain erythrocytic 1	Spta1	-1.0118	0.0165
900	P50446	Keratin type II cytoskeletal 6A	Krt6a	-1.0106	0.0165
901	Q9CWJ9	Bifunctional purine biosynthesis protein ATIC	Atic	-1.0105	0.0165
902	Q80TB8	Synaptic vesicle membrane protein VAT-1 homolog-like	Vat1l	-1.0085	0.0166
903	O55100	Synaptogyrin-1	Syngr1	-1.0077	0.0166
904	O70310	Glycylpeptide N-tetradecanoyltransferase 1	Nmt1	-1.0050	0.0168
905	P61028	Ras-related protein Rab-8B	Rab8b	-1.0034	0.0169
906	Q9QXK3	Coatomer subunit gamma-2	Copg2	-1.0034	0.0169
907	P70182	Phosphatidylinositol 4-phosphate 5-kinase type-1 alpha	Pip5k1a	-1.0030	0.0170
908	Q924M7	Mannose-6-phosphate isomerase	Mpi	-1.0026	0.0170
909	Q8BFT9	Synaptic vesicle 2-related protein	Svop	-1.0025	0.0170
910	Q91W50	Cold shock domain-containing protein E1	Csde1	-1.0004	0.0171
911	P62878	E3 ubiquitin-protein ligase RBX1	Rbx1	-0.9985	0.0171
912	D3Z7P3	Glutaminase kidney isoform mitochondrial	Gls	-0.9985	0.0171
913	Q9R1Q8	Transgelin-3	Tagln3	-0.9978	0.0171
914	Q99LI8	Hepatocyte growth factor-regulated tyrosine kinase substrate	Hgs	-0.9971	0.0171
915	Q9D0R2	Threonine--tRNA ligase 1 cytoplasmic	Tars1	-0.9971	0.0171
916	Q9DCU6	39S ribosomal protein L4 mitochondrial	Mrpl4	-0.9964	0.0172
917	P84086	Complexin-2	Cplx2	-0.9964	0.0172
918	Q45VK7	Cytoplasmic dynein 2 heavy chain 1	Dync2h1	-0.9964	0.0172
919	Q61292	Laminin subunit beta-2	Lamb2	-0.9959	0.0172
920	Q8C7R4	Ubiquitin-like modifier-activating enzyme 6	Uba6	-0.9958	0.0172
921	Q6NVF0	Inositol polyphosphate 5-phosphatase OCRL	Ocrl	-0.9956	0.0172
922	Q5H8C4	Vacuolar protein sorting-associated protein 13A	Vps13a	-0.9943	0.0173
923	Q64727	Vinculin	Vcl	-0.9896	0.0175
924	Q9CZM2	60S ribosomal protein L15	Rpl15	-0.9893	0.0175
925	Q8BMS1	Trifunctional enzyme subunit alpha mitochondrial	Hadha	-0.9882	0.0176
926	Q9D0R8	Protein LSM12 homolog	Lsm12	-0.9881	0.0176
927	Q80U30	Protein CLEC16A	Clec16a	-0.9881	0.0176
928	Q8BVI5	Syntaxin-16	Stx16	-0.9868	0.0178
929	Q8BLJ3	PI-PLC X domain-containing protein 3	Plcxd3	-0.9868	0.0178
930	Q60674	Nuclear receptor subfamily 1 group D member 2	Nr1d2	-0.9868	0.0178
931	Q9CQF0	39S ribosomal protein L11 mitochondrial	Mrpl11	-0.9860	0.0178
932	Q8VEK0	Cell cycle control protein 50A	Tmem30a	-0.9847	0.0179
933	Q8BHE3	Caytaxin	Atcay	-0.9847	0.0179

934	Q91YN0	Protein C12orf4 homolog	D6Wsu163e	-0.9845	0.0179
935	Q9CRB9	MIC complex subunit Mic19	Chchd3	-0.9802	0.0180
936	Q8VDM4	26S proteasome non-ATPase regulatory subunit 2	Psmd2	-0.9801	0.0181
937	Q9WUR9	Adenylate kinase 4 mitochondrial	Ak4	-0.9783	0.0181
938	Q9DBR1	5'-3' exoribonuclease 2	Xrn2	-0.9760	0.0182
939	Q91WG5	5'-AMP-activated protein kinase subunit gamma-2	Prkag2	-0.9757	0.0182
940	Q8BMF4	Dihydrolipoyllysine-residue acetyltransferase component of pyruvate dehydrogenase complex mitochondrial	Dlat	-0.9741	0.0183
941	P60603	Reactive oxygen species modulator 1	Romo1	-0.9728	0.0198
942	O08553	Dihydropyrimidinase-related protein 2	Dpysl2	-0.9721	0.0198
943	Q63836	Selenium-binding protein 2	Selenbp2	-0.9710	0.0200
944	P32883	GTPase KRas	Kras	-0.9710	0.0200
945	Q9EPA7	Nicotinamide/nicotinic acid mononucleotide adenylyltransferase 1	Nmnat1	-0.9709	0.0200
946	Q8BFR4	N-acetylglucosamine-6-sulfatase	Gns	-0.9709	0.0200
947	Q8VDD5	Myosin-9	Myh9	-0.9701	0.0200
948	P14602	Heat shock protein beta-1	Hspb1	-0.9661	0.0206
949	B1AXV0	DOMON domain-containing protein FRRS1L	Frrs1l	-0.9655	0.0206
950	Q8R0H9	ADP-ribosylation factor-binding protein GGA1	Gga1	-0.9651	0.0206
951	Q9CQH3	NADH dehydrogenase [ubiquinone] 1 beta subcomplex subunit 5 mitochondrial	Ndufb5	-0.9651	0.0206
952	Q8R4F1	Netrin-G2	Ntng2	-0.9648	0.0207
953	Q8VEM8	Phosphate carrier protein mitochondrial	Slc25a3	-0.9610	0.0211
954	Q8BKT7	THO complex subunit 5 homolog	Thoc5	-0.9600	0.0212
955	Q9CVB6	Actin-related protein 2/3 complex subunit 2	Arpc2	-0.9568	0.0213
956	Q91V14	Solute carrier family 12 member 5	Slc12a5	-0.9549	0.0214
957	Q6PDN3	Myosin light chain kinase smooth muscle	Mylk	-0.9537	0.0216
958	Q8CI51	PDZ and LIM domain protein 5	Pdlim5	-0.9535	0.0216
959	Q8VI36	Paxillin	Pxn	-0.9524	0.0216
960	P11679	Keratin type II cytoskeletal 8	Krt8	-0.9507	0.0219
961	P17742	Peptidyl-prolyl cis-trans isomerase A	Ppia	-0.9506	0.0219
962	Q3UFY8	tRNA methyltransferase 10 homolog C	Trmt10c	-0.9501	0.0219
963	Q62108	Disks large homolog 4	Dlg4	-0.9497	0.0223
964	O70566	Protein diaphanous homolog 2	Diaph2	-0.9487	0.0224
965	Q0VGY8	Protein TANC1	Tanc1	-0.9476	0.0225
966	P57784	U2 small nuclear ribonucleoprotein A'	Snrpa1	-0.9473	0.0225
967	Q9WV54	Acid ceramidase	Asah1	-0.9471	0.0225
968	O70325	Phospholipid hydroperoxide glutathione peroxidase	Gpx4	-0.9470	0.0225
969	Q3UVX5	Metabotropic glutamate receptor 5	Grm5	-0.9461	0.0225
970	Q9WV91	Prostaglandin F2 receptor negative regulator	Ptgfrn	-0.9454	0.0226
971	Q3UNZ8	Quinone oxidoreductase-like protein 2	Cryzl2	-0.9454	0.0226
972	Q91XY4	Protocadherin gamma-A4	Pcdhga4	-0.9454	0.0226
973	Q9ET26	E3 ubiquitin-protein ligase RNF114	Rnf114	-0.9431	0.0226
974	P68037	Ubiquitin-conjugating enzyme E2 L3	Ube2l3	-0.9423	0.0227
975	P61164	Alpha-centractin	Actr1a	-0.9405	0.0228
976	P47199	Quinone oxidoreductase	Cryz	-0.9394	0.0229
977	Q80Y81	Zinc phosphodiesterase ELAC protein 2	Elac2	-0.9352	0.0233
978	P07744	Keratin type II cytoskeletal 4	Krt4	-0.9351	0.0233
979	Q8VI75	Importin-4	Ipo4	-0.9345	0.0233
980	Q8C754	Vacuolar protein sorting-associated protein 52 homolog	Vps52	-0.9342	0.0233
981	P62761	Visinin-like protein 1	Vsnl1	-0.9314	0.0235
982	Q9JKF7	39S ribosomal protein L39 mitochondrial	Mrpl39	-0.9306	0.0236
983	Q9QUP5	Hyaluronan and proteoglycan link protein 1	Hapln1	-0.9280	0.0238
984	Q5DTN8	Janus kinase and microtubule-interacting protein 3	Jakmip3	-0.9276	0.0238
985	Q9DBC7	cAMP-dependent protein kinase type I-alpha regulatory subunit	Prkar1a	-0.9266	0.0239
986	Q8K212	Phosphofurin acidic cluster sorting protein 1	Pacs1	-0.9264	0.0239
987	Q6P2B1	Transportin-3	Tnpo3	-0.9231	0.0242
988	P63087	Serine/threonine-protein phosphatase PP1-gamma catalytic subunit	Ppp1cc	-0.9227	0.0242
989	Q8VBZ3	Cleft lip and palate transmembrane protein 1 homolog	Clptm1	-0.9226	0.0242
990	O35639	Annexin A3	Anxa3	-0.9226	0.0242
991	Q9JJ28	Protein flightless-1 homolog	Flii	-0.9201	0.0244
992	Q61301	Catenin alpha-2	Ctnna2	-0.9199	0.0244
993	Q99L13	3-hydroxyisobutyrate dehydrogenase mitochondrial	Hibadh	-0.9183	0.0245
994	Q68FM6	Protein phosphatase 1 regulatory subunit 29	Elfn2	-0.9162	0.0246
995	Q7TT50	Serine/threonine-protein kinase MRCK beta	Cdc42bpb	-0.9155	0.0247
996	P29533	Vascular cell adhesion protein 1	Vcam1	-0.9150	0.0247
997	Q9JKC8	AP-3 complex subunit mu-1	Ap3m1	-0.9150	0.0247

998	Q8VDK1	Deaminated glutathione amidase	Nit1	-0.9140	0.0247
999	Q80Y24	Prickle-like protein 2	Prickle2	-0.9115	0.0248
1000	Q78J03	Methionine-R-sulfoxide reductase B2 mitochondrial	Msrb2	-0.9115	0.0248
1001	P61804	Dolichyl-diphosphooligosaccharide--protein glycosyltransferase subunit DAD1	Dad1	-0.9115	0.0248
1002	P08030	Adenine phosphoribosyltransferase	Aprt	-0.9115	0.0248
1003	Q9D7X1	BTB/POZ domain-containing protein KCTD4	Kctd4	-0.9115	0.0248
1004	Q62422	Osteoclast-stimulating factor 1	Ostf1	-0.9115	0.0248
1005	P97298	Pigment epithelium-derived factor	Serpinf1	-0.9115	0.0248
1006	Q63810	Calcineurin subunit B type 1	Ppp3r1	-0.9115	0.0248
1007	O35955	Proteasome subunit beta type-10	Psmb10	-0.9115	0.0248
1008	Q99MI1	ELKS/Rab6-interacting/CAST family member 1	Erc1	-0.9112	0.0249
1009	P46664	Adenylosuccinate synthetase isozyme 2	Adss2	-0.9112	0.0249
1010	P47753	F-actin-capping protein subunit alpha-1	Capza1	-0.9108	0.0249
1011	P99029	Peroxiredoxin-5 mitochondrial	Prdx5	-0.9084	0.0251
1012	B9EKR1	Receptor-type tyrosine-protein phosphatase zeta	Ptprz1	-0.9069	0.0251
1013	P0C7L0	WAS/WASL-interacting protein family member 3	Wipf3	-0.9054	0.0252
1014	P06728	Apolipoprotein A-IV	Apoa4	-0.9047	0.0253
1015	Q9CYW4	Haloacid dehalogenase-like hydrolase domain-containing protein 3	Hdhd3	-0.9047	0.0253
1016	Q9R013	Cathepsin F	Ctsf	-0.9047	0.0253
1017	Q9JKK7	Tropomodulin-2	Tmod2	-0.9014	0.0255
1018	O09061	Proteasome subunit beta type-1	Psmb1	-0.8987	0.0259
1019	Q9CPR4	60S ribosomal protein L17	Rpl17	-0.8987	0.0259
1020	Q9Z2W9	Glutamate receptor 3	Gria3	-0.8981	0.0259
1021	Q80X60	EF-hand calcium-binding domain-containing protein 3	Efcab3	-0.8944	0.0261
1022	Q8K4Z5	Splicing factor 3A subunit 1	Sf3a1	-0.8940	0.0261
1023	P35550	rRNA 2'-O-methyltransferase fibrillarin	Fbl	-0.8933	0.0262
1024	P15864	Histone H1.2	H1-2	-0.8932	0.0262
1025	Q6PGH1	Protein BUD31 homolog	Bud31	-0.8907	0.0263
1026	Q91YP2	Neurolysin mitochondrial	Nln	-0.8855	0.0271
1027	Q8C8N2	Protein SCAI	Scai	-0.8844	0.0272
1028	P52332	Tyrosine-protein kinase JAK1	Jak1	-0.8843	0.0272
1029	Q60967	Bifunctional 3'-phosphoadenosine 5'-phosphosulfate synthase 1	Papss1	-0.8843	0.0272
1030	Q99M87	DnaJ homolog subfamily A member 3 mitochondrial	Dnaja3	-0.8843	0.0272
1031	O09131	Glutathione S-transferase omega-1	Gsto1	-0.8843	0.0272
1032	Q8CBW3	Abl interactor 1	Abi1	-0.8829	0.0273
1033	Q810U4	Neuronal cell adhesion molecule	Nrcam	-0.8810	0.0273
1034	Q69ZU6	Thrombospondin type-1 domain-containing protein 7A	Thsd7a	-0.8798	0.0274
1035	Q8VE80	THO complex subunit 3	Thoc3	-0.8791	0.0274
1036	Q6PDJ6	F-box only protein 42	Fbxo42	-0.8791	0.0274
1037	Q9CXY1	Endosomal/lysosomal potassium channel TMEM175	Tmem175	-0.8791	0.0274
1038	Q6ZPS6	Ankyrin repeat and IBR domain-containing protein 1	Ankib1	-0.8791	0.0274
1039	P70248	Unconventional myosin-If	Myo1f	-0.8791	0.0274
1040	P49222	Protein 4.2	Epb42	-0.8791	0.0274
1041	Q99MD6	Thioredoxin reductase 3	Txnrd3	-0.8791	0.0274
1042	Q9Z2D8	Methyl-CpG-binding domain protein 3	Mbd3	-0.8791	0.0274
1043	Q3ZT31	Sorting nexin-25	Snx25	-0.8791	0.0274
1044	Q80XS6	Protein Smaug homolog 2	Samd4b	-0.8791	0.0274
1045	Q8CHE4	PH domain leucine-rich repeat-containing protein phosphatase 1	Phlpp1	-0.8791	0.0274
1046	Q8C172	Ceramide synthase 6	Cers6	-0.8791	0.0274
1047	O88834	SH2 domain-containing adapter protein D	Shd	-0.8791	0.0274
1048	O88522	NF-kappa-B essential modulator	Ikbkg	-0.8791	0.0274
1049	Q68FF0	Uncharacterized protein KIAA1841	Kiaa1841	-0.8791	0.0274
1050	Q9QZL6	Ubiquitin carboxyl-terminal hydrolase 21	Usp21	-0.8791	0.0274
1051	Q91ZA3	Propionyl-CoA carboxylase alpha chain mitochondrial	Pcca	-0.8783	0.0274
1052	Q8BKG3	Inactive tyrosine-protein kinase 7	Ptk7	-0.8775	0.0275
1053	Q91VH2	Sorting nexin-9	Snx9	-0.8775	0.0275
1054	Q8K2P7	Sodium-coupled neutral amino acid transporter 1	Slc38a1	-0.8775	0.0275
1055	Q32M21	Gasdermin-A2	Gsdma2	-0.8775	0.0275
1056	Q9WUT3	Ribosomal protein S6 kinase alpha-2	Rps6ka2	-0.8764	0.0275
1057	P58044	Isopentenyl-diphosphate Delta-isomerase 1	Idi1	-0.8755	0.0277
1058	Q8K353	Cysteine-rich and transmembrane domain-containing protein 1	Cystm1	-0.8755	0.0277
1059	Q5M8N4	Epimerase family protein SDR39U1	Sdr39u1	-0.8755	0.0277
1060	Q9D5V5	Cullin-5	Cul5	-0.8751	0.0277
1061	P24668	Cation-dependent mannose-6-phosphate receptor	M6pr	-0.8746	0.0277
1062	Q4JIM5	Tyrosine-protein kinase ABL2	Abl2	-0.8746	0.0277
1063	O08912	Polypeptide N-acetylgalactosaminyltransferase 1	Galnt1	-0.8746	0.0277
1064	Q3UE37	Ubiquitin-conjugating enzyme E2 Z	Ube2z	-0.8721	0.0280

1065	P23818	Glutamate receptor 1	Gria1	-0.8718	0.0280
1066	Q6DFW4	Nucleolar protein 58	Nop58	-0.8709	0.0283
1067	Q8BHC1	Ras-related protein Rab-39B	Rab39b	-0.8705	0.0283
1068	Q80TL0	Protein phosphatase 1E	Ppm1e	-0.8690	0.0284
1069	Q8BL66	Early endosome antigen 1	Eea1	-0.8674	0.0286
1070	Q91ZJ5	UTP--glucose-1-phosphate uridylyltransferase	Ugp2	-0.8668	0.0286
1071	P12382	ATP-dependent 6-phosphofructokinase liver type	Pfkl	-0.8665	0.0287
1072	P80560	Receptor-type tyrosine-protein phosphatase N2	Ptprn2	-0.8652	0.0287
1073	P56528	ADP-ribosyl cyclase/cyclic ADP-ribose hydrolase 1	Cd38	-0.8652	0.0287
1074	Q9D8X1	Copper homeostasis protein cutC homolog	Cutc	-0.8652	0.0287
1075	Q61595	Kinectin	Ktn1	-0.8652	0.0287
1076	Q9DCR2	AP-3 complex subunit sigma-1	Ap3s1	-0.8652	0.0287
1077	Q60749	KH domain-containing RNA-binding signal transduction-associated protein 1	Khdrbs1	-0.8648	0.0288
1078	P62270	40S ribosomal protein S18	Rps18	-0.8629	0.0289
1079	P98084	Amyloid-beta A4 precursor protein-binding family A member 2	Apba2	-0.8628	0.0289
1080	Q91WK5	Glycine cleavage system H protein mitochondrial	Gcsh	-0.8628	0.0289
1081	Q9R0Q6	Actin-related protein 2/3 complex subunit 1A	Arpc1a	-0.8626	0.0289
1082	P21447	ATP-dependent translocase ABCB1	Abcb1a	-0.8614	0.0291
1083	P61161	Actin-related protein 2	Actr2	-0.8610	0.0292
1084	Q8BMS4	Ubiquinone biosynthesis O-methyltransferase mitochondrial	Coq3	-0.8599	0.0292
1085	Q9DD02	Protein Hikeshi	Hikeshi	-0.8599	0.0292
1086	Q91V57	N-chimaerin	Chn1	-0.8599	0.0292
1087	Q5Y4Y6	Gasdermin-A3	Gsdma3	-0.8599	0.0292
1088	Q8BHN3	Neutral alpha-glucosidase AB	Ganab	-0.8587	0.0294
1089	Q8BH74	Nuclear pore complex protein Nup107	Nup107	-0.8573	0.0295
1090	P15208	Insulin receptor	Insr	-0.8573	0.0295
1091	Q6P4S8	Integrator complex subunit 1	Ints1	-0.8573	0.0295
1092	P97461	40S ribosomal protein S5	Rps5	-0.8542	0.0297
1093	Q8R1V4	Transmembrane emp24 domain-containing protein 4	Tmed4	-0.8537	0.0297
1094	Q9CQM5	Thioredoxin domain-containing protein 17	Txndc17	-0.8537	0.0297
1095	P62320	Small nuclear ribonucleoprotein Sm D3	Snrpd3	-0.8537	0.0297
1096	Q99J36	THUMP domain-containing protein 1	Thumpd1	-0.8532	0.0298
1097	P51150	Ras-related protein Rab-7a	Rab7a	-0.8523	0.0298
1098	P28271	Cytoplasmic aconitate hydratase	Aco1	-0.8520	0.0298
1099	Q8R5C5	Beta-centractin	Actr1b	-0.8511	0.0299

Supplementary Table 3.

Number	Accession	Protein Name	Gene Name	PLGEM-STN	*p* Value
1	O70340	Neuronal pentraxin-2	*Nptx2*	3.4226	0.0001
2	Q14DL0	Ubiquilin-like protein	*Ubqlnl*	3.2976	0.0002
3	Q7TSJ2	Microtubule-associated protein 6	*Map6*	3.2974	0.0002
4	P20612	Guanine nucleotide-binding protein G(t) subunit alpha-1	*Gnat1*	3.2250	0.0002
5	P50149	Guanine nucleotide-binding protein G(t) subunit alpha-2	*Gnat2*	3.2172	0.0002
6	P04925	Major prion protein	*Prnp*	3.2049	0.0002
7	Q8CGK7	Guanine nucleotide-binding protein G(olf) subunit alpha	*Gnal*	3.1616	0.0002
8	Q3V3I2	Guanine nucleotide-binding protein G(t) subunit alpha-3	*Gnat3*	3.1521	0.0002
9	P27600	Guanine nucleotide-binding protein subunit alpha-12	*Gna12*	3.1467	0.0002
10	B2RSH2	Guanine nucleotide-binding protein G(i) subunit alpha-1	*Gnai1*	2.8841	0.0003
11	Q9DC51	Guanine nucleotide-binding protein G(i) subunit alpha-3	*Gnai3*	2.8207	0.0003
12	D3YVF0	A-kinase anchor protein 5	*Akap5*	2.8144	0.0003
13	O08599	Syntaxin-binding protein 1	*Stxbp1*	2.8054	0.0003
14	Q811Q9	Choline-phosphate cytidylyltransferase B	*Pcyt1b*	2.7984	0.0003
15	P14733	Lamin-B1	*Lmnb1*	2.7820	0.0003
16	Q922P8	Transmembrane protein 132A	*Tmem132a*	2.7385	0.0003
17	Q9WV92	Band 4.1-like protein 3	*Epb41l3*	2.6512	0.0003
18	P26645	Myristoylated alanine-rich C-kinase substrate	*Marcks*	2.5879	0.0004
19	Q811P8	Rho GTPase-activating protein 32	*Arhgap32*	2.5813	0.0004
20	B1AZI6	THO complex subunit 2	*Thoc2*	2.5587	0.0004
21	Q4FZC9	Nesprin-3	*Syne3*	2.4903	0.0004
22	Q61072	Disintegrin and metalloproteinase domain-containing protein 9	*Adam9*	2.4892	0.0004
23	P08752	Guanine nucleotide-binding protein G(i) subunit alpha-2	*Gnai2*	2.4740	0.0004
24	Q6Q477	Plasma membrane calcium-transporting ATPase 4	*Atp2b4*	2.4506	0.0004
25	E9PZ19	Protein turtle homolog B	*Igsf9b*	2.4439	0.0004
26	P48678	Prelamin-A/C	*Lmna*	2.4418	0.0004
27	Q8CFN5	Myocyte-specific enhancer factor 2C	*Mef2c*	2.3636	0.0005
28	P62881	Guanine nucleotide-binding protein subunit beta-5	*Gnb5*	2.3562	0.0005
29	Q5DTL9	Sodium-driven chloride bicarbonate exchanger	*Slc4a10*	2.3511	0.0005
30	Q3UKJ7	WD40 repeat-containing protein SMU1	*Smu1*	2.3293	0.0005
31	Q91YT0	NADH dehydrogenase [ubiquinone] flavoprotein 1 mitochondrial	*Ndufv1*	2.3192	0.0005
32	Q6R0H7	Guanine nucleotide-binding protein G(s) subunit alpha isoforms XLas	*Gnas*	2.3025	0.0005
33	P27601	Guanine nucleotide-binding protein subunit alpha-13	*Gna13*	2.2781	0.0006
34	Q5SVL6	Rap1 GTPase-activating protein 2	*Rap1gap2*	2.2711	0.0006
35	P63094	Guanine nucleotide-binding protein G(s) subunit alpha isoforms short	*Gnas*	2.2592	0.0006
36	P43276	Histone H1.5	*H1-5*	2.2263	0.0006
37	P31324	cAMP-dependent protein kinase type II-beta regulatory subunit	*Prkar2b*	2.2116	0.0006
38	Q9QWI6	SRC kinase signaling inhibitor 1	*Srcin1*	2.2066	0.0006
39	P49025	Citron Rho-interacting kinase	*Cit*	2.2043	0.0006
40	O08919	Numb-like protein	*Numbl*	2.1861	0.0006
41	Q8VIJ6	Splicing factor proline- and glutamine-rich	*Sfpq*	2.1761	0.0007
42	A2AQ25	Sickle tail protein	*Skt*	2.1616	0.0007
43	Q07409	Contactin-3	*Cntn3*	2.1175	0.0007
44	Q8JZK9	Hydroxymethylglutaryl-CoA synthase cytoplasmic	*Hmgcs1*	2.1072	0.0007
45	Q80UG5	Septin-9	*Septin9*	2.1050	0.0007
46	Q9R0K7	Plasma membrane calcium-transporting ATPase 2	*Atp2b2*	2.0905	0.0008
47	Q3UXZ6	Protein FAM81A	*Fam81a*	2.0626	0.0008
48	Q8C1B7	Septin-11	*Septin11*	2.0570	0.0008
49	O08644	Ephrin type-B receptor 6	*Ephb6*	2.0553	0.0008
50	Q99104	Unconventional myosin-Va	*Myo5a*	2.0398	0.0008
51	Q80XD1	Beta-chimaerin	*Chn2*	2.0371	0.0008
52	Q99M74	Keratin type II cuticular Hb2	*Krt82*	2.0371	0.0008
53	Q8QZY1	Eukaryotic translation initiation factor 3 subunit L	*Eif3l*	2.0284	0.0008
54	Q08091	Calponin-1	*Cnn1*	1.9997	0.0009
55	Q61001	Laminin subunit alpha-5	*Lama5*	1.9950	0.0009
56	Q8K310	Matrin-3	*Matr3*	1.9737	0.0009
57	Q8BLE7	Vesicular glutamate transporter 2	*Slc17a6*	1.9423	0.0010
58	Q3TVA9	Coiled-coil domain-containing protein 136	*Ccdc136*	1.9397	0.0010
59	P28665	Murinoglobulin-1	*Mug1*	1.9293	0.0011
60	Q9CS84	Neurexin-1	*Nrxn1*	1.9254	0.0011
61	Q8CHT1	Ephexin-1	*Ngef*	1.9241	0.0011

62	Q8K010	5-oxoprolinase	*Oplah*	1.9136	0.0011
63	Q9DBS9	Oxysterol-binding protein-related protein 3	*Osbpl3*	1.8906	0.0011
64	P12849	cAMP-dependent protein kinase type I-beta regulatory subunit	*Prkar1b*	1.8758	0.0012
65	Q8VHJ5	Serine/threonine-protein kinase MARK1	*Mark1*	1.8671	0.0012
66	O09012	Peroxisomal targeting signal 1 receptor	*Pex5*	1.8568	0.0012
67	Q62132	Receptor-type tyrosine-protein phosphatase R	*Ptprr*	1.8568	0.0012
68	Q8BYW1	Rho GTPase-activating protein 25	*Arhgap25*	1.8568	0.0012
69	Q9D486	C-Maf-inducing protein	*Cmip*	1.8568	0.0012
70	D3YZU1	SH3 and multiple ankyrin repeat domains protein 1	*Shank1*	1.8556	0.0012
71	E9PUL5	Proline-rich transmembrane protein 2	*Prrt2*	1.8513	0.0012
72	Q9QWY8	Arf-GAP with SH3 domain ANK repeat and PH domain-containing protein 1	*Asap1*	1.8460	0.0012
73	Q8BYN5	FSD1-like protein	*Fsd1l*	1.8438	0.0012
74	Q8CGI1	Protein FAM193A	*Fam193a*	1.8331	0.0012
75	Q6P4T0	Autophagy-related protein 2 homolog A	*Atg2a*	1.8215	0.0012
76	P97490	Adenylate cyclase type 8	*Adcy8*	1.8215	0.0012
77	Q9WVA3	Mitotic checkpoint protein BUB3	*Bub3*	1.8174	0.0012
78	O35954	Membrane-associated phosphatidylinositol transfer protein 1	*Pitpnm1*	1.8164	0.0012
79	Q9QZS3	Protein numb homolog	*Numb*	1.8160	0.0012
80	Q04750	DNA topoisomerase 1	*Top1*	1.8067	0.0013
81	O89053	Coronin-1A	*Coro1a*	1.8030	0.0013
82	P48318	Glutamate decarboxylase 1	*Gad1*	1.7891	0.0013
83	P20357	Microtubule-associated protein 2	*Map2*	1.7862	0.0013
84	Q6PE01	U5 small nuclear ribonucleoprotein 40 kDa protein	*Snrnp40*	1.7830	0.0013
85	Q6P9K8	Caskin-1	*Caskin1*	1.7724	0.0014
86	P18872	Guanine nucleotide-binding protein G(o) subunit alpha	*Gnao1*	1.7591	0.0014
87	Q61024	Asparagine synthetase [glutamine-hydrolyzing]	*Asns*	1.7588	0.0014
88	Q7TT37	Elongator complex protein 1	*Elp1*	1.7568	0.0015
89	A2ALS5	Rap1 GTPase-activating protein 1	*Rap1gap*	1.7544	0.0015
90	Q63912	Oligodendrocyte-myelin glycoprotein	*Omg*	1.7311	0.0015
91	Q8VD12	Zinc finger protein 385A	*Znf385a*	1.7285	0.0015
92	Q6NZJ6	Eukaryotic translation initiation factor 4 gamma 1	*Eif4g1*	1.7280	0.0015
93	Q5DU25	IQ motif and SEC7 domain-containing protein 2	*Iqsec2*	1.7203	0.0015
94	Q9JKY5	Huntingtin-interacting protein 1-related protein	*Hip1r*	1.7137	0.0015
95	Q8C7H1	Methylmalonic aciduria type A homolog mitochondrial	*Mmaa*	1.6796	0.0016
96	O88737	Protein bassoon	*Bsn*	1.6759	0.0016
97	Q570Y9	DEP domain-containing mTOR-interacting protein	*Deptor*	1.6689	0.0016
98	P63056	Noelin-3	*Olfm3*	1.6689	0.0016
99	P26231	Catenin alpha-1	*Ctnna1*	1.6630	0.0016
100	Q6QD59	Vesicle transport protein SEC20	*Bnip1*	1.6575	0.0016
101	Q6PDG5	SWI/SNF complex subunit SMARCC2	*Smarcc2*	1.6549	0.0016
102	Q7TNR6	Immunoglobulin superfamily member 21	*Igsf21*	1.6529	0.0016
103	Q9WTX6	Cullin-1	*Cul1*	1.6453	0.0017
104	Q7TQG1	Pleckstrin homology domain-containing family A member 6	*Plekha6*	1.6319	0.0017
105	Q6PIC6	Sodium/potassium-transporting ATPase subunit alpha-3	*Atp1a3*	1.6278	0.0017
106	Q80U72	Protein scribble homolog	*Scrib*	1.5995	0.0018
107	Q8C2Q3	RNA-binding protein 14	*Rbm14*	1.5926	0.0018
108	Q00422	GA-binding protein alpha chain	*Gabpa*	1.5912	0.0018
109	Q5Y4Y6	Gasdermin-A3	*Gsdma3*	1.5912	0.0018
110	Q8BM13	Noelin-2	*Olfm2*	1.5890	0.0018
111	Q6VNB8	WD repeat and FYVE domain-containing protein 3	*Wdfy3*	1.5837	0.0018
112	Q8CC35	Synaptopodin	*Synpo*	1.5826	0.0019
113	Q5DU31	Interactor protein for cytohesin exchange factors 1	*Ipcef1*	1.5813	0.0019
114	Q8K4P8	E3 ubiquitin-protein ligase HECW1	*Hecw1*	1.5813	0.0019
115	P47962	60S ribosomal protein L5	*Rpl5*	1.5770	0.0019
116	Q61137	Astrotactin-1	*Astn1*	1.5734	0.0019
117	Q8R0A7	Uncharacterized protein KIAA0513	*Kiaa0513*	1.5698	0.0019
118	Q8CG72	ADP-ribose glycohydrolase ARH3	*Adprs*	1.5648	0.0019
119	E9Q7X7	Neurexin-2	*Nrxn2*	1.5632	0.0019
120	Q62283	Tetraspanin-7	*Tspan7*	1.5543	0.0019
121	Q9DBR3	Armadillo repeat-containing protein 8	*Armc8*	1.5495	0.0019
122	Q99P47	Contactin-associated protein-like 4	*Cntnap4*	1.5488	0.0019
123	P07310	Creatine kinase M-type	*Ckm*	1.5465	0.0020
124	Q8VCN9	Tubulin-specific chaperone C	*Tbcc*	1.5300	0.0021

125	Q8R4U6	DNA topoisomerase I mitochondrial	*Top1mt*	1.5266	0.0021
126	Q149F3	Eukaryotic peptide chain release factor GTP-binding subunit ERF3B	*Gspt2*	1.5251	0.0021
127	Q61271	Activin receptor type-1B	*Acvr1b*	1.5138	0.0021
128	Q3UTZ3	Trafficking protein particle complex subunit 14	*Trappc14*	1.5038	0.0022
129	F8VPU2	FERM ARHGEF and pleckstrin domain-containing protein 1	*Farp1*	1.4974	0.0022
130	Q6PFD5	Disks large-associated protein 3	*Dlgap3*	1.4967	0.0022
131	P11983	T-complex protein 1 subunit alpha	*Tcp1*	1.4958	0.0022
132	P15533	Tripartite motif-containing protein 30A	*Trim30a*	1.4956	0.0022
133	P52800	Ephrin-B2	*Efnb2*	1.4938	0.0022
134	Q8QZV7	Integrator complex subunit 13	*IntS13*	1.4938	0.0022
135	O55131	Septin-7	*Septin7*	1.4882	0.0023
136	Q3UY34	Protein CUST	*Custos*	1.4740	0.0024
137	Q922L6	Negative elongation factor D	*Nelfcd*	1.4740	0.0024
138	Q80U49	Centrosomal protein of 170 kDa protein B	*Cep170b*	1.4732	0.0024
139	Q8BGW1	Alpha-ketoglutarate-dependent dioxygenase FTO	*Fto*	1.4719	0.0024
140	P31750	RAC-alpha serine/threonine-protein kinase	*Akt1*	1.4705	0.0024
141	O88447	Kinesin light chain 1	*Klc1*	1.4687	0.0024
142	P43006	Excitatory amino acid transporter 2	*Slc1a2*	1.4680	0.0024
143	P53986	Monocarboxylate transporter 1	*Slc16a1*	1.4661	0.0024
144	Q61301	Catenin alpha-2	*Ctnna2*	1.4648	0.0024
145	P07901	Heat shock protein HSP 90-alpha	*Hsp90aa1*	1.4623	0.0025
146	P11499	Heat shock protein HSP 90-beta	*Hsp90ab1*	1.4621	0.0025
147	P07724	Albumin	*Alb*	1.4617	0.0025
148	Q8K2K6	Arf-GAP domain and FG repeat-containing protein 1	*Agfg1*	1.4608	0.0025
149	P49586	Choline-phosphate cytidylyltransferase A	*Pcyt1a*	1.4595	0.0025
150	Q0KK59	Protein unc-79 homolog	*Unc79*	1.4595	0.0025
151	Q9JJA9	General receptor for phosphoinositides 1-associated scaffold protein	*Tamalin*	1.4511	0.0025
152	P01901	H-2 class I histocompatibility antigen K-B alpha chain	*H2-K1*	1.4489	0.0025
153	Q62165	Dystroglycan	*Dag1*	1.4487	0.0025
154	Q8VD65	Phosphoinositide 3-kinase regulatory subunit 4	*Pik3r4*	1.4478	0.0026
155	Q8CJ19	[F-actin]-monooxygenase MICAL3	*Mical3*	1.4462	0.0026
156	Q63943	Myocyte-specific enhancer factor 2D	*Mef2d*	1.4407	0.0026
157	Q80YF9	Rho GTPase-activating protein 33	*Arhgap33*	1.4393	0.0026
158	Q920Q4	Vacuolar protein sorting-associated protein 16 homolog	*Vps16*	1.4239	0.0027
159	Q8BH60	Golgi-associated PDZ and coiled-coil motif-containing protein	*Gopc*	1.4202	0.0030
160	Q8C525	Protein MB21D2	*Mb21d2*	1.4202	0.0030
161	O35926	Cyclin-dependent kinase 5 activator 2	*Cdk5r2*	1.4202	0.0030
162	P97386	DNA ligase 3	*Lig3*	1.4202	0.0030
163	A2AKX3	Probable helicase senataxin	*Setx*	1.4202	0.0030
164	Q8CJ61	CKLF-like MARVEL transmembrane domain-containing protein 4	*Cmtm4*	1.4087	0.0031
165	Q9WUA6	RAC-gamma serine/threonine-protein kinase	*Akt3*	1.4068	0.0031
166	P10852	4F2 cell-surface antigen heavy chain	*Slc3a2*	1.4045	0.0031
167	Q8BRT1	CLIP-associating protein 2	*Clasp2*	1.4013	0.0031
168	Q9D8X2	Coiled-coil domain-containing protein 124	*Ccdc124*	1.3985	0.0031
169	Q99KR3	Endoribonuclease LACTB2	*Lactb2*	1.3973	0.0032
170	Q64314	Hematopoietic progenitor cell antigen CD34	*Cd34*	1.3973	0.0032
171	P52479	Ubiquitin carboxyl-terminal hydrolase 10	*Usp10*	1.3952	0.0032
172	Q61233	Plastin-2	*Lcp1*	1.3824	0.0034
173	Q8CIP4	MAP/microtubule affinity-regulating kinase 4	*Mark4*	1.3797	0.0034
174	Q80YX1	Tenascin	*Tnc*	1.3793	0.0034
175	Q60823	RAC-beta serine/threonine-protein kinase	*Akt2*	1.3766	0.0034
176	P34914	Bifunctional epoxide hydrolase 2	*Ephx2*	1.3714	0.0035
177	P50446	Keratin type II cytoskeletal 6A	*Krt6a*	1.3665	0.0035
178	Q91ZM2	SH2B adapter protein 1	*Sh2b1*	1.3640	0.0035
179	Q9EQF6	Dihydropyrimidinase-related protein 5	*Dpysl5*	1.3608	0.0035
180	Q8K0D0	Cyclin-dependent kinase 17	*Cdk17*	1.3582	0.0036
181	Q6A009	E3 ubiquitin-protein ligase listerin	*Ltn1*	1.3552	0.0036
182	Q00560	Interleukin-6 receptor subunit beta	*Il6st*	1.3530	0.0036
183	Q6P5U7	NACHT and WD repeat domain-containing protein 2	*Nwd2*	1.3521	0.0036
184	Q9QXS1	Plectin	*Plec*	1.3496	0.0037
185	Q9JI39	ATP-binding cassette sub-family B member 10 mitochondrial	*Abcb10*	1.3485	0.0037
186	Q3TIR3	Synembryn-A	*Ric8a*	1.3447	0.0037
187	Q8JZP2	Synapsin-3	*Syn3*	1.3427	0.0037

188	O70174	Neuronal acetylcholine receptor subunit alpha-4	*Chrna4*	1.3419	0.0037
189	Q91Y63	Solute carrier family 13 member 3	*Slc13a3*	1.3419	0.0037
190	Q8C9B9	Death-inducer obliterator 1	*Dido1*	1.3419	0.0037
191	A2A8U2	Transmembrane protein 201	*Tmem201*	1.3419	0.0037
192	Q6ZQA6	Immunoglobulin superfamily member 3	*Igsf3*	1.3419	0.0037
193	Q8C5K5	Uncharacterized protein CXorf38 homolog		1.3419	0.0037
194	Q922K7	Probable 28S rRNA (cytosine-C(5))-methyltransferase	*Nop2*	1.3419	0.0037
195	Q91V17	E3 ubiquitin-protein ligase ZNRF1	*Znrf1*	1.3419	0.0037
196	Q91YT2	E3 ubiquitin-protein ligase RNF185	*Rnf185*	1.3419	0.0037
197	Q9CWX9	Probable ATP-dependent RNA helicase DDX47	*Ddx47*	1.3419	0.0037
198	P22933	Gamma-aminobutyric acid receptor subunit delta	*Gabrd*	1.3419	0.0037
199	Q91V14	Solute carrier family 12 member 5	*Slc12a5*	1.3380	0.0037
200	Q9Z2Q6	Septin-5	*Septin5*	1.3374	0.0037
201	Q8JZS0	Protein lin-7 homolog A	*Lin7a*	1.3362	0.0040
202	Q80TK0	AP2-interacting clathrin-endocytosis protein	*Kiaa1107*	1.3317	0.0040
203	Q8BUR4	Dedicator of cytokinesis protein 1	*Dock1*	1.3317	0.0040
204	O35927	Catenin delta-2	*Ctnnd2*	1.3315	0.0040
205	Q64096	Guanine nucleotide exchange factor DBS	*Mcf2l*	1.3279	0.0040
206	Q9WVR4	Fragile X mental retardation syndrome-related protein 2	*Fxr2*	1.3271	0.0040
207	Q8CFE4	SCY1-like protein 2	*Scyl2*	1.3243	0.0040
208	Q9QZB0	Regulator of G-protein signaling 17	*Rgs17*	1.3242	0.0040
209	Q5DTT2	PH and SEC7 domain-containing protein 1	*Psd*	1.3223	0.0041
210	O88643	Serine/threonine-protein kinase PAK 1	*Pak1*	1.3203	0.0041
211	Q3UTQ8	Cyclin-dependent kinase-like 5	*Cdkl5*	1.3200	0.0041
212	Q6V4S5	Protein sidekick-2	*Sdk2*	1.3177	0.0041
213	Q9QYJ3	DnaJ homolog subfamily B member 1	*Dnajb1*	1.3170	0.0041
214	E9Q912	Rap1 GTPase-GDP dissociation stimulator 1	*Rap1gds1*	1.3166	0.0041
215	P14206	40S ribosomal protein SA	*Rpsa*	1.3150	0.0041
216	Q6PDN3	Myosin light chain kinase smooth muscle	*Mylk*	1.3137	0.0042
217	Q8VEH5	EPM2A-interacting protein 1	*Epm2aip1*	1.3134	0.0042
218	Q80XL6	Acyl-CoA dehydrogenase family member 11	*Acad11*	1.3034	0.0043
219	Q3UTJ2	Sorbin and SH3 domain-containing protein 2	*Sorbs2*	1.3009	0.0043
220	Q61644	Protein kinase C and casein kinase substrate in neurons protein 1	*Pacsin1*	1.2991	0.0044
221	Q8BJ42	Disks large-associated protein 2	*Dlgap2*	1.2970	0.0044
222	P34152	Focal adhesion kinase 1	*Ptk2*	1.2947	0.0044
223	Q9JKS5	Intracellular hyaluronan-binding protein 4	*Habp4*	1.2946	0.0044
224	Q8C078	Calcium/calmodulin-dependent protein kinase kinase 2	*Camkk2*	1.2920	0.0044
225	Q91WJ8	Far upstream element-binding protein 1	*Fubp1*	1.2912	0.0044
226	Q8C4G9	Adhesion G protein-coupled receptor A1	*Adgra1*	1.2907	0.0044
227	Q8R1F1	Protein Niban 2	*Niban2*	1.2907	0.0044
228	Q8BUH8	Sentrin-specific protease 7	*Senp7*	1.2907	0.0044
229	Q05BC3	Echinoderm microtubule-associated protein-like 1	*Eml1*	1.2902	0.0044
230	O08808	Protein diaphanous homolog 1	*Diaph1*	1.2858	0.0045
231	P28867	Protein kinase C delta type	*Prkcd*	1.2855	0.0045
232	Q9QXT8	Calsenilin	*Kcnip3*	1.2855	0.0045
233	B2RQL2	Storkhead-box protein 1	*Stox1*	1.2849	0.0045
234	Q9JME5	AP-3 complex subunit beta-2	*Ap3b2*	1.2836	0.0045
235	Q6PGB6	N-alpha-acetyltransferase 50	*Naa50*	1.2791	0.0046
236	P08775	DNA-directed RNA polymerase II subunit RPB1	*Polr2a*	1.2787	0.0046
237	Q69Z98	Serine/threonine-protein kinase BRSK2	*Brsk2*	1.2774	0.0046
238	Q8C6B2	Rhotekin	*Rtkn*	1.2759	0.0046
239	P52189	Inward rectifier potassium channel 4	*Kcnj4*	1.2759	0.0046
240	Q9CZ44	NSFL1 cofactor p47	*Nsfl1c*	1.2756	0.0046
241	Q9QYB1	Chloride intracellular channel protein 4	*Clic4*	1.2737	0.0047
242	Q91V12	Cytosolic acyl coenzyme A thioester hydrolase	*Acot7*	1.2664	0.0048
243	Q9JJV5	Voltage-dependent calcium channel gamma-3 subunit	*Cacng3*	1.2597	0.0049
244	Q6PAV2	Probable E3 ubiquitin-protein ligase HERC4	*Herc4*	1.2580	0.0049
245	Q64735	Complement component receptor 1-like protein	*Cr1l*	1.2490	0.0050
246	Q91XL9	Oxysterol-binding protein-related protein 1	*Osbpl1a*	1.2441	0.0051
247	Q01147	Cyclic AMP-responsive element-binding protein 1	*Creb1*	1.2421	0.0051
248	Q80TY0	Formin-binding protein 1	*Fnbp1*	1.2416	0.0051
249	Q8CI61	BAG family molecular chaperone regulator 4	*Bag4*	1.2360	0.0053
250	Q8C7D2	Protein cereblon	*Crbn*	1.2360	0.0053

251	Q9R0N3	Synaptotagmin-11	*Syt11*	1.2360	0.0053
252	Q9CPR7	Suppressor of IKBKE 1	*Sike1*	1.2360	0.0053
253	Q8BZH4	Pogo transposable element with ZNF domain	*Pogz*	1.2360	0.0053
254	Q0PMG2	MAM domain-containing glycosylphosphatidylinositol anchor protein 1	*Mdga1*	1.2360	0.0053
255	P58158	Galactosylgalactosylxylosylprotein 3-beta-glucuronosyltransferase 3	*B3gat3*	1.2360	0.0053
256	Q6PDH0	Pleckstrin homology-like domain family B member 1	*Phldb1*	1.2360	0.0053
257	Q80U57	Regulating synaptic membrane exocytosis protein 3	*Rims3*	1.2340	0.0054
258	Q02357	Ankyrin-1	*Ank1*	1.2330	0.0054
259	P48722	Heat shock 70 kDa protein 4L	*Hspa4l*	1.2291	0.0054
260	Q7TNF0	Double C2-like domain-containing protein alpha	*Doc2a*	1.2230	0.0056
261	Q7TNG5	Echinoderm microtubule-associated protein-like 2	*Eml2*	1.2225	0.0056
262	Q8BRK8	5'-AMP-activated protein kinase catalytic subunit alpha-2	*Prkaa2*	1.2203	0.0057
263	Q8K448	Cholesterol transporter ABCA5	*Abca5*	1.2203	0.0057
264	Q02248	Catenin beta-1	*Ctnnb1*	1.2173	0.0057
265	Q3TY86	Apoptosis-inducing factor 3	*Aifm3*	1.2160	0.0057
266	P51830	Adenylate cyclase type 9	*Adcy9*	1.2100	0.0058
267	Q9JI10	Serine/threonine-protein kinase 3	*Stk3*	1.2099	0.0058
268	Q9JIS5	Synaptic vesicle glycoprotein 2A	*Sv2a*	1.2053	0.0059
269	P97819	85/88 kDa calcium-independent phospholipase A2	*Pla2g6*	1.2041	0.0059
270	Q9ERE2	Keratin type II cuticular Hb1	*Krt81*	1.2041	0.0059
271	P97861	Keratin type II cuticular Hb6	*Krt86*	1.2041	0.0059
272	Q62095	ATP-dependent RNA helicase DDX3Y	*Ddx3y*	1.2039	0.0059
273	A2A432	Cullin-4B	*Cul4b*	1.2036	0.0059
274	Q9JHU4	Cytoplasmic dynein 1 heavy chain 1	*Dync1h1*	1.2015	0.0060
275	Q920M5	Coronin-6	*Coro6*	1.1979	0.0060
276	P50608	Fibromodulin	*Fmod*	1.1977	0.0060
277	Q02257	Junction plakoglobin	*Jup*	1.1939	0.0061
278	Q99PV0	Pre-mRNA-processing-splicing factor 8	*Prpf8*	1.1903	0.0071
279	Q3UNH4	G protein-regulated inducer of neurite outgrowth 1	*Gprin1*	1.1882	0.0072
280	O88741	Ganglioside-induced differentiation-associated protein 1	*Gdap1*	1.1877	0.0073
281	Q68EF4	Metabotropic glutamate receptor 4	*Grm4*	1.1870	0.0073
282	Q9CWL8	Beta-catenin-like protein 1	*Ctnnbl1*	1.1861	0.0073
283	Q9ERB0	Synaptosomal-associated protein 29	*Snap29*	1.1861	0.0073
284	Q8CAK3	Shiftless antiviral inhibitor of ribosomal frameshifting protein homolog	*Shfl*	1.1861	0.0073
285	Q8BH24	Transmembrane 9 superfamily member 4	*Tm9sf4*	1.1861	0.0073
286	Q3TLH4	Protein PRRC2C	*Prrc2c*	1.1780	0.0077
287	Q8K124	Pleckstrin homology domain-containing family O member 2	*Plekho2*	1.1741	0.0077
288	Q9BCZ4	Selenoprotein S	*Selenos*	1.1741	0.0077
289	P70213	Friend virus susceptibility protein 1	*Fv1*	1.1741	0.0077
290	Q91YE3	Egl nine homolog 1	*Egln1*	1.1741	0.0077
291	B2RXR6	Serine/threonine-protein phosphatase 6 regulatory ankyrin repeat subunit B	*Ankrd44*	1.1741	0.0077
292	Q9WUB0	RanBP-type and C3HC4-type zinc finger-containing protein 1	*Rbck1*	1.1741	0.0077
293	Q9D6T0	Nitric oxide synthase-interacting protein	*Nosip*	1.1741	0.0077
294	Q7TSH4	Centriolar coiled-coil protein of 110 kDa	*Ccp110*	1.1741	0.0077
295	Q61324	Aryl hydrocarbon receptor nuclear translocator 2	*Arnt2*	1.1741	0.0077
296	Q5BL07	Peroxisome biogenesis factor 1	*Pex1*	1.1741	0.0077
297	Q7TN22	Thioredoxin domain-containing protein 16	*Txndc16*	1.1741	0.0077
298	Q6ZPR4	Potassium channel subfamily T member 1	*Kcnt1*	1.1741	0.0077
299	Q91VB2	Calcium/calmodulin-dependent protein kinase type 1G	*Camk1g*	1.1741	0.0077
300	Q8VHQ4	Ras-related protein Rab-40C	*Rab40c*	1.1741	0.0077
301	Q32M21	Gasdermin-A2	*Gsdma2*	1.1741	0.0077
302	P36993	Protein phosphatase 1B	*Ppm1b*	1.1732	0.0077
303	A2AJI0	MAP7 domain-containing protein 1	*Map7d1*	1.1728	0.0078
304	Q8K012	Formin-binding protein 1-like	*Fnbp1l*	1.1710	0.0078
305	P23780	Beta-galactosidase	*Glb1*	1.1660	0.0079
306	Q8BSK8	Ribosomal protein S6 kinase beta-1	*Rps6kb1*	1.1636	0.0080
307	O35841	Apoptosis inhibitor 5	*Api5*	1.1613	0.0080
308	Q8VDP4	Cell cycle and apoptosis regulator protein 2	*Ccar2*	1.1608	0.0080
309	Q9JKN6	RNA-binding protein Nova-1	*Nova1*	1.1606	0.0080
310	Q80TL4	PHD finger protein 24	*Phf24*	1.1604	0.0080
311	Q8VCT9	Dual specificity testis-specific protein kinase 2	*Tesk2*	1.1597	0.0080
312	Q9JKK8	Serine/threonine-protein kinase ATR	*Atr*	1.1563	0.0082
313	P70303	CTP synthase 2	*Ctps2*	1.1551	0.0083

314	Q922S4	cGMP-dependent 3' 5'-cyclic phosphodiesterase	*Pde2a*	1.1543	0.0083
315	E9Q6P5	Tetratricopeptide repeat protein 7B	*Ttc7b*	1.1490	0.0084
316	Q99PU5	Long-chain-fatty-acid--CoA ligase ACSBG1	*Acsbg1*	1.1456	0.0084
317	P47708	Rabphilin-3A	*Rph3a*	1.1453	0.0084
318	P70288	Histone deacetylase 2	*Hdac2*	1.1406	0.0085
319	Q8R555	Cartilage acidic protein 1	*Crtac1*	1.1406	0.0085
320	Q8CIN6	CUGBP Elav-like family member 3	*Celf3*	1.1398	0.0086
321	O54829	Regulator of G-protein signaling 7	*Rgs7*	1.1396	0.0086
322	Q8BTI8	Serine/arginine repetitive matrix protein 2	*Srrm2*	1.1381	0.0087
323	Q9CQU5	ZW10 interactor	*Zwint*	1.1346	0.0087
324	Q8R0G9	Nuclear pore complex protein Nup133	*Nup133*	1.1330	0.0088
325	Q9D6I9	Leucine rich adaptor protein 1	*Lurap1*	1.1330	0.0088
326	Q9ES00	Ubiquitin conjugation factor E4 B	*Ube4b*	1.1312	0.0090
327	P60882	Multiple epidermal growth factor-like domains protein 8	*Megf8*	1.1293	0.0091
328	P55194	SH3 domain-binding protein 1	*Sh3bp1*	1.1253	0.0092
329	O70400	PDZ and LIM domain protein 1	*Pdlim1*	1.1240	0.0092
330	Q8K004	Spermatogenesis-associated protein 2	*Spata2*	1.1222	0.0092
331	Q9D1K2	V-type proton ATPase subunit F	*Atp6v1f*	1.1222	0.0092
332	Q3U2I3	FHF complex subunit HOOK interacting protein 1B	*Fhip1b*	1.1222	0.0092
333	Q8BWT5	Disco-interacting protein 2 homolog A	*Dip2a*	1.1219	0.0093
334	A2ADY9	Protein DDI1 homolog 2	*Ddi2*	1.1205	0.0093
335	Q9D2N9	Vacuolar protein sorting-associated protein 33A	*Vps33a*	1.1205	0.0093
336	Q8BGF9	Solute carrier family 25 member 44	*Slc25a44*	1.1204	0.0093
337	P53798	Squalene synthase	*Fdft1*	1.1154	0.0094
338	Q8BM65	Neuronal tyrosine-phosphorylated phosphoinositide-3-kinase adapter 2	*Nyap2*	1.1146	0.0095
339	Q8R138	Transmembrane protein 119	*Tmem119*	1.1146	0.0095
340	Q505D9	Tripartite motif-containing protein 67	*Trim67*	1.1146	0.0095
341	Q3UYC0	Protein phosphatase 1H	*Ppm1h*	1.1128	0.0095
342	Q8BLQ9	Cell adhesion molecule 2	*Cadm2*	1.1127	0.0095
343	A2A690	Protein TANC2	*Tanc2*	1.1124	0.0095
344	P22723	Gamma-aminobutyric acid receptor subunit gamma-2	*Gabrg2*	1.1115	0.0095
345	P54763	Ephrin type-B receptor 2	*Ephb2*	1.1087	0.0096
346	Q8VEE1	LIM and cysteine-rich domains protein 1	*Lmcd1*	1.1085	0.0096
347	P70298	Homeobox protein cut-like 2	*Cux2*	1.1075	0.0096
348	Q6PIE5	Sodium/potassium-transporting ATPase subunit alpha-2	*Atp1a2*	1.1053	0.0099
349	Q8BX10	Serine/threonine-protein phosphatase PGAM5 mitochondrial	*Pgam5*	1.1020	0.0100
350	Q3UH93	Plexin-D1	*Plxnd1*	1.1005	0.0101
351	P56695	Wolframin	*Wfs1*	1.0978	0.0102
352	Q61191	Host cell factor 1	*Hcfc1*	1.0976	0.0102
353	Q8BGZ1	Hippocalcin-like protein 4	*Hpcal4*	1.0967	0.0102
354	P62812	Gamma-aminobutyric acid receptor subunit alpha-1	*Gabra1*	1.0954	0.0102
355	Q91VR5	ATP-dependent RNA helicase DDX1	*Ddx1*	1.0947	0.0102
356	Q64487	Receptor-type tyrosine-protein phosphatase delta	*Ptprd*	1.0916	0.0103
357	Q60598	Src substrate cortactin	*Cttn*	1.0876	0.0104
358	O08784	Treacle protein	*Tcof1*	1.0862	0.0105
359	O08532	Voltage-dependent calcium channel subunit alpha-2/delta-1	*Cacna2d1*	1.0835	0.0105
360	Q8VDL4	ADP-dependent glucokinase	*Adpgk*	1.0812	0.0107
361	O89001	Carboxypeptidase D	*Cpd*	1.0798	0.0107
362	Q3TCH7	Cullin-4A	*Cul4a*	1.0798	0.0107
363	Q9QUI0	Transforming protein RhoA	*Rhoa*	1.0705	0.0121
364	Q91VL8	Telomeric repeat-binding factor 2-interacting protein 1	*Terf2ip*	1.0665	0.0123
365	Q6P6J9	Thioredoxin domain-containing protein 15	*Txndc15*	1.0665	0.0123
366	Q811C2	Cysteine protease ATG4C	*Atg4c*	1.0665	0.0123
367	Q7TST3	Sterile alpha motif domain-containing protein 10	*Samd10*	1.0665	0.0123
368	Q8VDP2	STING ER exit protein	*Steep1*	1.0665	0.0123
369	D3Z4I3	RNA-binding protein 24	*Rbm24*	1.0665	0.0123
370	Q62176	RNA-binding protein 38	*Rbm38*	1.0665	0.0123
371	Q8BXL7	ADP-ribosylation factor-related protein 1	*Arfrp1*	1.0665	0.0123
372	Q9Z1B5	Mitotic spindle assembly checkpoint protein MAD2A	*Mad2l1*	1.0665	0.0123
373	Q9JIL5	Tubby-related protein 4	*Tulp4*	1.0665	0.0123
374	Q9DCI3	STARD3 N-terminal-like protein	*Stard3nl*	1.0665	0.0123
375	A2AQ19	RNA polymerase-associated protein RTF1 homolog	*Rtf1*	1.0665	0.0123
376	Q60949	TBC1 domain family member 1	*Tbc1d1*	1.0665	0.0123

377	Q9ER41	Torsin-1B	*Tor1b*	1.0665	0.0123
378	Q9R171	Cerebellin-1	*Cbln1*	1.0665	0.0123
379	Q8BGU2	Cerebellin-2	*Cbln2*	1.0665	0.0123
380	Q9CWU4	UPF0690 protein C1orf52 homolog		1.0665	0.0123
381	O70281	Protein-tyrosine sulfotransferase 1	*Tpst1*	1.0665	0.0123
382	Q6A051	Attractin-like protein 1	*Atrnl1*	1.0665	0.0123
383	P35822	Receptor-type tyrosine-protein phosphatase kappa	*Ptprk*	1.0665	0.0123
384	Q8VI93	2'-5'-oligoadenylate synthase 3	*Oas3*	1.0665	0.0123
385	P13745	Glutathione S-transferase A1	*Gsta1*	1.0651	0.0124
386	P46097	Synaptotagmin-2	*Syt2*	1.0648	0.0124
387	Q9D4V0	Ethanolamine kinase 1	*Etnk1*	1.0646	0.0124
388	P62257	Ubiquitin-conjugating enzyme E2 H	*Ube2h*	1.0646	0.0124
389	Q8BKC5	Importin-5	*Ipo5*	1.0646	0.0124
390	Q62383	Transcription elongation factor SPT6	*Supt6h*	1.0619	0.0126
391	P40336	Vacuolar protein sorting-associated protein 26A	*Vps26a*	1.0609	0.0126
392	Q9JMG3	Transmembrane and ubiquitin-like domain-containing protein 1	*Tmub1*	1.0606	0.0126
393	P23116	Eukaryotic translation initiation factor 3 subunit A	*Eif3a*	1.0604	0.0126
394	P37804	Transgelin	*Tagln*	1.0592	0.0127
395	Q62188	Dihydropyrimidinase-related protein 3	*Dpysl3*	1.0589	0.0127
396	Q9D2R0	Acetoacetyl-CoA synthetase	*Aacs*	1.0581	0.0128
397	P70205	Pituitary adenylate cyclase-activating polypeptide type I receptor	*Adcyap1r1*	1.0573	0.0128
398	Q9ERK4	Exportin-2	*Cse1l*	1.0566	0.0128
399	Q68FH0	Plakophilin-4	*Pkp4*	1.0553	0.0128
400	P97797	Tyrosine-protein phosphatase non-receptor type substrate 1	*Sirpa*	1.0512	0.0130
401	Q04899	Cyclin-dependent kinase 18	*Cdk18*	1.0439	0.0134
402	Q02819	Nucleobindin-1	*Nucb1*	1.0431	0.0134
403	Q61578	NADPH:adrenodoxin oxidoreductase mitochondrial	*Fdxr*	1.0429	0.0134
404	Q8CGF7	Transcription elongation regulator 1	*Tcerg1*	1.0428	0.0134
405	Q8VDN2	Sodium/potassium-transporting ATPase subunit alpha-1	*Atp1a1*	1.0406	0.0135
406	Q8CCT4	Transcription elongation factor A protein-like 5	*Tceal5*	1.0397	0.0135
407	Q61768	Kinesin-1 heavy chain	*Kif5b*	1.0395	0.0135
408	Q6PB44	Tyrosine-protein phosphatase non-receptor type 23	*Ptpn23*	1.0386	0.0136
409	Q8BUK6	Protein Hook homolog 3	*Hook3*	1.0360	0.0137
410	Q9Z321	DNA topoisomerase 3-beta-1	*Top3b*	1.0354	0.0138
411	Q64514	Tripeptidyl-peptidase 2	*Tpp2*	1.0335	0.0139
412	Q8C996	Transmembrane protein 163	*Tmem163*	1.0333	0.0139
413	Q0VBL3	RNA-binding protein 15	*Rbm15*	1.0330	0.0139
414	Q99J77	Sialic acid synthase	*Nans*	1.0319	0.0139
415	O08810	116 kDa U5 small nuclear ribonucleoprotein component	*Eftud2*	1.0298	0.0140
416	O88602	Voltage-dependent calcium channel gamma-2 subunit	*Cacng2*	1.0289	0.0140
417	Q8CCX5	Keratin-like protein KRT222	*Krt222*	1.0289	0.0140
418	Q8BQZ4	Ral GTPase-activating protein subunit beta	*Ralgapb*	1.0283	0.0140
419	Q99LB2	Dehydrogenase/reductase SDR family member 4	*Dhrs4*	1.0278	0.0141
420	P59823	Interleukin-1 receptor accessory protein-like 1	*Il1rapl1*	1.0245	0.0141
421	P41778	Pre-B-cell leukemia transcription factor 1	*Pbx1*	1.0245	0.0141
422	Q62178	Semaphorin-4A	*Sema4a*	1.0245	0.0141
423	Q14BI2	Metabotropic glutamate receptor 2	*Grm2*	1.0241	0.0141
424	Q8BYM8	Probable cysteine--tRNA ligase mitochondrial	*Cars2*	1.0222	0.0142
425	Q3UHE1	Membrane-associated phosphatidylinositol transfer protein 3	*Pitpnm3*	1.0222	0.0142
426	A2AFS3	Endosome/lysosome-associated apoptosis and autophagy regulator 1	*Elapor1*	1.0213	0.0143
427	Q8BTS4	Nuclear pore complex protein Nup54	*Nup54*	1.0213	0.0143
428	Q9EQS9	Immunoglobulin superfamily DCC subclass member 4	*Igdcc4*	1.0213	0.0143
429	Q8VDI7	Ubiquitin-associated domain-containing protein 1	*Ubac1*	1.0213	0.0143
430	Q61923	Potassium voltage-gated channel subfamily A member 6	*Kcna6*	1.0173	0.0145
431	Q6I6G8	E3 ubiquitin-protein ligase HECW2	*Hecw2*	1.0173	0.0145
432	Q61189	Methylosome subunit pICln	*Clns1a*	1.0173	0.0145
433	Q9D394	Protein RUFY3	*Rufy3*	1.0169	0.0145
434	Q9D6Z1	Nucleolar protein 56	*Nop56*	1.0153	0.0145
435	Q9EPE9	Endoplasmic reticulum transmembrane helix translocase	*Atp13a1*	1.0138	0.0146
436	Q8R3V5	Endophilin-B2	*Sh3glb2*	1.0097	0.0148
437	B2RY56	RNA-binding protein 25	*Rbm25*	1.0077	0.0148
438	Q8BGZ4	Cell division cycle protein 23 homolog	*Cdc23*	1.0044	0.0150
439	P07903	DNA excision repair protein ERCC-1	*Ercc1*	1.0044	0.0150

440	Q9QYS9	Protein quaking	*Qki*	1.0041	0.0150
441	Q8C854	Myelin expression factor 2	*Myef2*	1.0012	0.0152
442	Q8R0I4	TM2 domain-containing protein 2	*Tm2d2*	0.9995	0.0152
443	Q9EPW0	Inositol polyphosphate-4-phosphatase type I A	*Inpp4a*	0.9991	0.0153
444	Q811D0	Disks large homolog 1	*Dlg1*	0.9974	0.0153
445	Q99NB8	Ubiquilin-4	*Ubqln4*	0.9969	0.0153
446	Q91WG4	Elongator complex protein 2	*Elp2*	0.9964	0.0153
447	P47857	ATP-dependent 6-phosphofructokinase muscle type	*Pfkm*	0.9948	0.0154
448	Q8C729	Protein FAM126B	*Fam126b*	0.9943	0.0154
449	Q06335	Amyloid-like protein 2	*Aplp2*	0.9940	0.0154
450	O08672	Kinesin-like protein KIFC2	*Kifc2*	0.9940	0.0154
451	Q5SUE8	Ankyrin repeat domain-containing protein 40	*Ankrd40*	0.9940	0.0154
452	Q811S7	Upstream-binding protein 1	*Ubp1*	0.9940	0.0154
453	Q8BZB2	Phosphopantothenoylcysteine decarboxylase	*Ppcdc*	0.9940	0.0154
454	Q0VGB7	Serine/threonine-protein phosphatase 4 regulatory subunit 2	*Ppp4r2*	0.9940	0.0154
455	O35075	Vacuolar protein sorting-associated protein 26C	*Vps26c*	0.9940	0.0154
456	Q3UN02	Lysocardiolipin acyltransferase 1	*Lclat1*	0.9940	0.0154
457	Q5SWP3	NAC-alpha domain-containing protein 1	*Nacad*	0.9940	0.0154
458	P97298	Pigment epithelium-derived factor	*Serpinf1*	0.9940	0.0154
459	Q6PB93	Polypeptide N-acetylgalactosaminyltransferase 2	*Galnt2*	0.9940	0.0154
460	Q640M6	Glycerophosphodiester phosphodiesterase domain-containing protein 5	*Gdpd5*	0.9940	0.0154
461	Q9DBX6	Cytochrome P450 2S1	*Cyp2s1*	0.9940	0.0154
462	O35607	Bone morphogenetic protein receptor type-2	*Bmpr2*	0.9940	0.0154
463	O35166	Golgi SNAP receptor complex member 2	*Gosr2*	0.9940	0.0154
464	Q9DAI6	Protein FAM135B	*Fam135b*	0.9940	0.0154
465	Q9ERS5	Pleckstrin homology domain-containing family A member 2	*Plekha2*	0.9940	0.0154
466	Q8BP22	Protein FAM92A	*Fam92a*	0.9940	0.0154
467	O54836	Zinc finger matrin-type protein 3	*Zmat3*	0.9940	0.0154
468	Q99MJ9	ATP-dependent RNA helicase DDX50	*Ddx50*	0.9940	0.0154
469	Q9D968	Host cell factor 2	*Hcfc2*	0.9940	0.0154
470	Q8VE85	PRELI domain containing protein 3A	*Prelid3a*	0.9940	0.0154
471	O35417	Proenkephalin-B	*Pdyn*	0.9940	0.0154
472	Q99MD9	Nuclear autoantigenic sperm protein	*Nasp*	0.9940	0.0154
473	Q9EQB9	Zinc finger protein 287	*Znf287*	0.9940	0.0154
474	P08074	Carbonyl reductase [NADPH] 2	*Cbr2*	0.9940	0.0154
475	P97300	Neuroplastin	*Nptn*	0.9917	0.0155
476	Q8BG81	Polymerase delta-interacting protein 3	*Poldip3*	0.9911	0.0157
477	Q5SSL4	Active breakpoint cluster region-related protein	*Abr*	0.9847	0.0174
478	Q7M6Z0	Reticulon-4 receptor-like 2	*Rtn4rl2*	0.9846	0.0174
479	Q9ER72	Cysteine--tRNA ligase cytoplasmic	*Cars1*	0.9837	0.0177
480	O70591	Prefoldin subunit 2	*Pfdn2*	0.9836	0.0177
481	Q8JZR0	Long-chain-fatty-acid--CoA ligase 5	*Acsl5*	0.9818	0.0177
482	Q8JZR6	Electroneutral sodium bicarbonate exchanger 1	*Slc4a8*	0.9807	0.0178
483	P70257	Nuclear factor 1 X-type	*Nfix*	0.9803	0.0178
484	P70271	PDZ and LIM domain protein 4	*Pdlim4*	0.9803	0.0178
485	Q8VHQ9	Acyl-coenzyme A thioesterase 11	*Acot11*	0.9777	0.0180
486	O88532	Zinc finger RNA-binding protein	*Zfr*	0.9773	0.0182
487	Q9WVH9	Fibulin-5	*Fbln5*	0.9753	0.0183
488	Q9ERL9	Guanylate cyclase soluble subunit alpha-1	*Gucy1a1*	0.9753	0.0183
489	Q4KMM3	Oxidation resistance protein 1	*Oxr1*	0.9690	0.0187
490	Q6P4T2	U5 small nuclear ribonucleoprotein 200 kDa helicase	*Snrnp200*	0.9686	0.0187
491	Q8CHS8	Vacuolar protein sorting-associated protein 37A	*Vps37a*	0.9681	0.0187
492	Q6NSW3	A-kinase anchor protein SPHKAP	*Sphkap*	0.9664	0.0188
493	Q3TPX4	Exocyst complex component 5	*Exoc5*	0.9659	0.0188
494	Q9JJ43	RNA binding protein fox-1 homolog 1	*Rbfox1*	0.9618	0.0190
495	P06537	Glucocorticoid receptor	*Nr3c1*	0.9611	0.0190
496	Q8BIE6	FERM domain-containing protein 4A	*Frmd4a*	0.9611	0.0190
497	Q8BHK1	Magnesium transporter NIPA1	*Nipa1*	0.9611	0.0190
498	Q9Z2V5	Histone deacetylase 6	*Hdac6*	0.9598	0.0192
499	Q99K28	ADP-ribosylation factor GTPase-activating protein 2	*Arfgap2*	0.9598	0.0192
500	Q9Z2D3	Gasdermin-E	*Gsdme*	0.9589	0.0192
501	Q8C4Y3	Negative elongation factor B	*Nelfb*	0.9582	0.0194
502	O70318	Band 4.1-like protein 2	*Epb41l2*	0.9578	0.0194

503	Q61739	Integrin alpha-6	*Itga6*	0.9575	0.0194
504	Q9Z275	Retinaldehyde-binding protein 1	*Rlbp1*	0.9550	0.0197
505	Q6PGC1	ATP-dependent RNA helicase DHX29	*Dhx29*	0.9550	0.0197
506	P20029	Endoplasmic reticulum chaperone BiP	*Hspa5*	0.9546	0.0198
507	Q6NXK7	Inactive dipeptidyl peptidase 10	*Dpp10*	0.9533	0.0198
508	Q99JR1	Sideroflexin-1	*Sfxn1*	0.9533	0.0198
509	Q9D154	Leukocyte elastase inhibitor A	*Serpinb1a*	0.9518	0.0199
510	P70392	Ras-specific guanine nucleotide-releasing factor 2	*Rasgrf2*	0.9504	0.0200
511	Q9CPU4	Microsomal glutathione S-transferase 3	*Mgst3*	0.9504	0.0200
512	Q8BZF8	Phosphoglucomutase-like protein 5	*Pgm5*	0.9463	0.0205
513	P35980	60S ribosomal protein L18	*Rpl18*	0.9455	0.0206
514	Q8BLK9	Ribosomal protein S6 kinase delta-1	*Rps6kc1*	0.9452	0.0206
515	Q9D0Q7	39S ribosomal protein L45 mitochondrial	*Mrpl45*	0.9437	0.0206
516	Q5DTY9	BTB/POZ domain-containing protein KCTD16	*Kctd16*	0.9396	0.0208
517	Q8BNU0	Armadillo repeat-containing protein 6	*Armc6*	0.9387	0.0208
518	Q9WTS5	Teneurin-2	*Tenm2*	0.9384	0.0211
519	Q9CR26	Vacuolar protein sorting-associated protein VTA1 homolog	*Vta1*	0.9372	0.0211
520	Q61481	Calcium/calmodulin-dependent 3' 5'-cyclic nucleotide phosphodiesterase 1A	*Pde1a*	0.9369	0.0211
521	P16627	Heat shock 70 kDa protein 1-like	*Hspa1l*	0.9360	0.0212
522	Q8BHS3	Pre-mRNA-splicing factor RBM22	*Rbm22*	0.9341	0.0214
523	Q64467	Glyceraldehyde-3-phosphate dehydrogenase testis-specific	*Gapdhs*	0.9336	0.0214
524	A2AT37	Regulator of nonsense transcripts 2	*Upf2*	0.9332	0.0214
525	Q8C419	Probable G-protein coupled receptor 158	*Gpr158*	0.9285	0.0217
526	Q6P4S6	Serine/threonine-protein kinase SIK3	*Sik3*	0.9276	0.0218
527	P12367	cAMP-dependent protein kinase type II-alpha regulatory subunit	*Prkar2a*	0.9271	0.0218
528	Q80U56	Late secretory pathway protein AVL9 homolog	*Avl9*	0.9270	0.0219
529	Q9EPU0	Regulator of nonsense transcripts 1	*Upf1*	0.9258	0.0219
530	Q62083	PRKCA-binding protein	*Pick1*	0.9243	0.0220
531	Q9Z2H2	Regulator of G-protein signaling 6	*Rgs6*	0.9238	0.0220
532	Q78ZA7	Nucleosome assembly protein 1-like 4	*Nap1l4*	0.9202	0.0223
533	Q91YD3	mRNA-decapping enzyme 1A	*Dcp1a*	0.9196	0.0223
534	Q8VDP3	[F-actin]-monooxygenase MICAL1	*Mical1*	0.9170	0.0226
535	P60191	Regulating synaptic membrane exocytosis protein 4	*Rims4*	0.9170	0.0226
536	Q9D8P4	39S ribosomal protein L17 mitochondrial	*Mrpl17*	0.9170	0.0226
537	E9Q9D5	Rab-like protein 2A	*Rabl2*	0.9170	0.0226
538	P50516	V-type proton ATPase catalytic subunit A	*Atp6v1a*	0.9166	0.0226
539	P61460	GATOR complex protein DEPDC5	*Depdc5*	0.9165	0.0226
540	Q8C015	Serine/threonine-protein kinase PAK 5	*Pak5*	0.9155	0.0226
541	Q810B6	Rabankyrin-5	*Ankfy1*	0.9152	0.0226
542	P12023	Amyloid-beta A4 protein	*App*	0.9103	0.0229
543	Q9CXW4	60S ribosomal protein L11	*Rpl11*	0.9095	0.0229
544	Q8JZW5	SH2 domain-containing protein 5	*Sh2d5*	0.9082	0.0232
545	Q9D2N4	Dystrobrevin alpha	*Dtna*	0.9082	0.0232
546	P41241	Tyrosine-protein kinase CSK	*Csk*	0.9082	0.0232
547	A2ARP1	Inositol hexakisphosphate and diphosphoinositol-pentakisphosphate kinase 1	*Ppip5k1*	0.9081	0.0232
548	Q6TEK5	Vitamin K epoxide reductase complex subunit 1-like protein 1	*Vkorc1l1*	0.9075	0.0232
549	Q91XU0	ATPase WRNIP1	*Wrnip1*	0.9061	0.0234
550	P61620	Protein transport protein Sec61 subunit alpha isoform 1	*Sec61a1*	0.9035	0.0237
551	Q9D8W5	26S proteasome non-ATPase regulatory subunit 12	*Psmd12*	0.9016	0.0239
552	Q61315	Adenomatous polyposis coli protein	*Apc*	0.9016	0.0239
553	Q60780	Growth arrest-specific protein 7	*Gas7*	0.8982	0.0244
554	P26041	Moesin	*Msn*	0.8963	0.0246
555	Q9DBG9	Tax1-binding protein 3	*Tax1bp3*	0.8959	0.0246
556	Q4ZJN1	Complement C1q and tumor necrosis factor-related protein 9	*C1qtnf9*	0.8954	0.0246
557	Q9Z0S1	3'(2') 5'-bisphosphate nucleotidase 1	*Bpnt1*	0.8951	0.0246
558	Q3UH60	Disco-interacting protein 2 homolog B	*Dip2b*	0.8943	0.0246
559	Q9EQH3	Vacuolar protein sorting-associated protein 35	*Vps35*	0.8920	0.0260
560	P35235	Tyrosine-protein phosphatase non-receptor type 11	*Ptpn11*	0.8918	0.0260
561	Q8BHL3	TBC1 domain family member 10B	*Tbc1d10b*	0.8908	0.0263
562	O88487	Cytoplasmic dynein 1 intermediate chain 2	*Dync1i2*	0.8895	0.0265
563	Q3TCJ1	BRISC complex subunit Abraxas 2	*Abraxas2*	0.8894	0.0265
564	Q9JMB8	Contactin-6	*Cntn6*	0.8894	0.0265
565	Q99LR1	Lysophosphatidylserine lipase ABHD12	*Abhd12*	0.8891	0.0265

566	Q61699	Heat shock protein 105 kDa	*Hsph1*	0.8884	0.0265
567	Q8BIJ7	RUN and FYVE domain-containing protein 1	*Rufy1*	0.8880	0.0265
568	Q9DAW6	U4/U6 small nuclear ribonucleoprotein Prp4	*Prpf4*	0.8880	0.0265
569	Q8BIF2	RNA binding protein fox-1 homolog 3	*Rbfox3*	0.8869	0.0266
570	Q8C7R4	Ubiquitin-like modifier-activating enzyme 6	*Uba6*	0.8848	0.0272
571	Q91Z31	Polypyrimidine tract-binding protein 2	*Ptbp2*	0.8818	0.0274
572	Q4ACU6	SH3 and multiple ankyrin repeat domains protein 3	*Shank3*	0.8795	0.0275
573	Q9QXG2	Rab proteins geranylgeranyltransferase component A 1	*Chm*	0.8778	0.0276
574	Q8R574	Phosphoribosyl pyrophosphate synthase-associated protein 2	*Prpsap2*	0.8759	0.0277
575	Q9WTR1	Transient receptor potential cation channel subfamily V member 2	*Trpv2*	0.8756	0.0277
576	Q8CH09	SURP and G-patch domain-containing protein 2	*Sugp2*	0.8743	0.0277
577	Q60629	Ephrin type-A receptor 5	*Epha5*	0.8736	0.0278
578	Q9DBG7	Signal recognition particle receptor subunit alpha	*Srpra*	0.8725	0.0279
579	Q9R226	KH domain-containing RNA-binding signal transduction-associated protein 3	*Khdrbs3*	0.8719	0.0279
580	Q5F2E7	Nuclear fragile X mental retardation-interacting protein 2	*Nufip2*	0.8709	0.0280
581	Q9JHU9	Inositol-3-phosphate synthase 1	*Isyna1*	0.8700	0.0280
582	Q9QXJ1	Amyloid-beta A4 precursor protein-binding family B member 1	*Apbb1*	0.8680	0.0281
583	Q91V92	ATP-citrate synthase	*Acly*	0.8666	0.0282
584	P68181	cAMP-dependent protein kinase catalytic subunit beta	*Prkacb*	0.8666	0.0282
585	Q0KL02	Triple functional domain protein	*Trio*	0.8657	0.0283
586	Q61490	CD166 antigen	*Alcam*	0.8644	0.0284
587	Q99J39	Malonyl-CoA decarboxylase mitochondrial	*Mlycd*	0.8643	0.0285
588	Q9JJZ4	Ubiquitin-conjugating enzyme E2 J1	*Ube2j1*	0.8640	0.0285
589	Q6ZPU9	KIF-binding protein	*Kifbp*	0.8640	0.0285
590	Q5U4F6	Cytoplasmic dynein 2 intermediate chain 2	*Dync2i2*	0.8639	0.0286
591	P63137	Gamma-aminobutyric acid receptor subunit beta-2	*Gabrb2*	0.8632	0.0286
592	A2AQP0	Myosin-7B	*Myh7b*	0.8628	0.0287
593	Q6NS60	F-box only protein 41	*Fbxo41*	0.8603	0.0288
594	Q3UDE2	Tubulin--tyrosine ligase-like protein 12	*Ttll12*	0.8592	0.0288
595	Q9Z2H5	Band 4.1-like protein 1	*Epb41l1*	0.8566	0.0292
596	O89029	Matrilin-4	*Matn4*	0.8556	0.0293
597	Q8VE73	Cullin-7	*Cul7*	0.8547	0.0293
598	E9Q3L2	Phosphatidylinositol 4-kinase alpha	*Pi4ka*	0.8547	0.0293
599	Q8BWZ3	N-alpha-acetyltransferase 25 NatB auxiliary subunit	*Naa25*	0.8527	0.0295
600	P08003	Protein disulfide-isomerase A4	*Pdia4*	0.8504	0.0298

Supplementary Table 4.

Number	Accession	Protein Name	Gene Name	PLGEM-STN	*p* Value
1	P60202	Myelin proteolipid protein	*Plp1*	-11.2113	0.0000
2	Q9CWF2	Tubulin beta-2B chain	*Tubb2b*	-8.3793	0.0000
3	Q7TMM9	Tubulin beta-2A chain	*Tubb2a*	-8.1485	0.0000
4	P68372	Tubulin beta-4B chain	*Tubb4b*	-7.0781	0.0000
5	P99024	Tubulin beta-5 chain	*Tubb5*	-6.3516	0.0000
6	Q9D6F9	Tubulin beta-4A chain	*Tubb4a*	-6.2305	0.0000
7	Q922F4	Tubulin beta-6 chain	*Tubb6*	-4.6802	0.0001
8	Q0VE82	Copine-7	*Cpne7*	-4.5807	0.0001
9	P16330	2' 3'-cyclic-nucleotide 3'-phosphodiesterase	*Cnp*	-4.3320	0.0001
10	P10649	Glutathione S-transferase Mu 1	*Gstm1*	-4.3084	0.0001
11	P63158	High mobility group protein B1	*Hmgb1*	-4.2832	0.0001
12	Q9Z140	Copine-6	*Cpne6*	-4.2237	0.0001
13	P04370	Myelin basic protein	*Mbp*	-4.0933	0.0001
14	O54983	Ketimine reductase mu-crystallin	*Crym*	-3.9207	0.0001
15	P01869	Ig gamma-1 chain C region membrane-bound form	*Ighg1*	-3.6882	0.0001
16	E9PV24	Fibrinogen alpha chain	*Fga*	-3.6449	0.0001
17	Q9DB05	Alpha-soluble NSF attachment protein	*Napa*	-3.6091	0.0001
18	P01868	Ig gamma-1 chain C region secreted form	*Ighg1*	-3.5617	0.0001
19	Q80XN0	D-beta-hydroxybutyrate dehydrogenase mitochondrial	*Bdh1*	-3.3187	0.0002
20	P11798	Calcium/calmodulin-dependent protein kinase type II subunit alpha	*Camk2a*	-3.2023	0.0002
21	Q61885	Myelin-oligodendrocyte glycoprotein	*Mog*	-3.1555	0.0002
22	P12246	Serum amyloid P-component	*Apcs*	-3.1367	0.0002
23	Q9QVP9	Protein-tyrosine kinase 2-beta	*Ptk2b*	-3.1031	0.0002
24	B1AQJ2	Ubiquitin carboxyl-terminal hydrolase 36	*Usp36*	-3.0336	0.0002
25	Q9Z0E0	Neurochondrin	*Ncdn*	-2.9705	0.0002
26	P01027	Complement C3	*C3*	-2.8866	0.0002
27	Q9EQK5	Major vault protein	*Mvp*	-2.8693	0.0002
28	P59108	Copine-2	*Cpne2*	-2.8595	0.0003
29	P05784	Keratin type I cytoskeletal 18	*Krt18*	-2.8437	0.0003
30	Q0VBF8	Protein stum homolog	*Stum*	-2.7978	0.0003
31	Q9D379	Epoxide hydrolase 1	*Ephx1*	-2.7954	0.0003
32	Q9R0Q6	Actin-related protein 2/3 complex subunit 1A	*Arpc1a*	-2.7468	0.0003
33	Q4FZF3	Probable ATP-dependent RNA helicase DDX49	*Ddx49*	-2.6957	0.0003
34	Q9JJH1	Ribonuclease 4	*Rnase4*	-2.6892	0.0003
35	P00405	Cytochrome c oxidase subunit 2	*Mtco2*	-2.6539	0.0003
36	Q9QZX7	Serine racemase	*Srr*	-2.6312	0.0003
37	P48453	Serine/threonine-protein phosphatase 2B catalytic subunit beta isoform	*Ppp3cb*	-2.6280	0.0003
38	O08600	Endonuclease G mitochondrial	*Endog*	-2.6202	0.0003
39	P51881	ADP/ATP translocase 2	*Slc25a5*	-2.6031	0.0004
40	Q921I1	Serotransferrin	*Tf*	-2.5951	0.0004
41	P57746	V-type proton ATPase subunit D	*Atp6v1d*	-2.5795	0.0004
42	Q80X60	EF-hand calcium-binding domain-containing protein 3	*Efcab3*	-2.5790	0.0004
43	P68254	14-3-3 protein theta	*Ywhaq*	-2.5690	0.0004
44	Q06185	ATP synthase subunit e mitochondrial	*Atp5me*	-2.5600	0.0004
45	Q811L6	Microtubule-associated serine/threonine-protein kinase 4	*Mast4*	-2.5549	0.0004
46	P46460	Vesicle-fusing ATPase	*Nsf*	-2.5515	0.0004
47	P01029	Complement C4-B	*C4b*	-2.5336	0.0004
48	P08249	Malate dehydrogenase mitochondrial	*Mdh2*	-2.5211	0.0004
49	P40240	CD9 antigen	*Cd9*	-2.5192	0.0004
50	P51910	Apolipoprotein D	*Apod*	-2.4981	0.0004
51	P47757	F-actin-capping protein subunit beta	*Capzb*	-2.4938	0.0004
52	Q9D711	Pirin	*Pir*	-2.4892	0.0004
53	Q9ESM3	Hyaluronan and proteoglycan link protein 2	*Hapln2*	-2.4723	0.0004
54	P84075	Neuron-specific calcium-binding protein hippocalcin	*Hpca*	-2.4623	0.0004
55	P15626	Glutathione S-transferase Mu 2	*Gstm2*	-2.4548	0.0004
56	Q61282	Aggrecan core protein	*Acan*	-2.4406	0.0004
57	P35846	Folate receptor alpha	*Folr1*	-2.4347	0.0004
58	P62702	40S ribosomal protein S4 X isoform	*Rps4x*	-2.4258	0.0004
59	P68033	Actin alpha cardiac muscle 1	*Actc1*	-2.4216	0.0004
60	P55200	Histone-lysine N-methyltransferase 2A	*Kmt2a*	-2.4134	0.0004
61	P19536	Cytochrome c oxidase subunit 5B mitochondrial	*Cox5b*	-2.4079	0.0004
62	Q80VQ0	Aldehyde dehydrogenase family 3 member B1	*Aldh3b1*	-2.3677	0.0005
63	Q9DBJ1	Phosphoglycerate mutase 1	*Pgam1*	-2.3664	0.0005
64	Q9D4F2	Phospholipid phosphatase 6	*Plpp6*	-2.3636	0.0005
65	Q60931	Voltage-dependent anion-selective channel protein 3	*Vdac3*	-2.3590	0.0005
66	P05064	Fructose-bisphosphate aldolase A	*Aldoa*	-2.3422	0.0005
67	Q9D051	Pyruvate dehydrogenase E1 component subunit beta mitochondrial	*Pdhb*	-2.3153	0.0006
68	Q61646	Haptoglobin	*Hp*	-2.3139	0.0006
69	Q80W21	Glutathione S-transferase Mu 7	*Gstm7*	-2.3020	0.0006
70	Q9D7B6	Isobutyryl-CoA dehydrogenase mitochondrial	*Acad8*	-2.2881	0.0006
71	Q64332	Synapsin-2	*Syn2*	-2.2875	0.0006
72	P59764	Dedicator of cytokinesis protein 4	*Dock4*	-2.2841	0.0006
73	P54071	Isocitrate dehydrogenase [NADP] mitochondrial	*Idh2*	-2.2762	0.0006
74	P01864	Ig gamma-2A chain C region secreted form		-2.2711	0.0006
75	Q6P9Q6	FK506-binding protein 15	*Fkbp15*	-2.2408	0.0006
76	Q80WR1	Tetraspanin-18	*Tspan18*	-2.2408	0.0006
77	Q3UGR5	Haloacid dehalogenase-like hydrolase domain-containing protein 2	*Hdhd2*	-2.2357	0.0006

78	Q3URD3	Sarcolemmal membrane-associated protein	*Slmap*	-2.2353	0.0006
79	Q9DCT2	NADH dehydrogenase [ubiquinone] iron-sulfur protein 3 mitochondrial	*Ndufs3*	-2.2348	0.0006
80	P19639	Glutathione S-transferase Mu 3	*Gstm3*	-2.2332	0.0006
81	P11679	Keratin type II cytoskeletal 8	*Krt8*	-2.2264	0.0006
82	G3X9J0	Signal-induced proliferation-associated 1-like protein 3	*Sipa1l3*	-2.1856	0.0007
83	O88533	Aromatic-L-amino-acid decarboxylase	*Ddc*	-2.1762	0.0007
84	Q9QUP5	Hyaluronan and proteoglycan link protein 1	*Hapln1*	-2.1755	0.0007
85	P63318	Protein kinase C gamma type	*Prkcg*	-2.1606	0.0007
86	Q9ERD7	Tubulin beta-3 chain	*Tubb3*	-2.1528	0.0007
87	Q99J99	3-mercaptopyruvate sulfurtransferase	*Mpst*	-2.1494	0.0007
88	Q62446	Peptidyl-prolyl cis-trans isomerase FKBP3	*Fkbp3*	-2.1482	0.0007
89	P16858	Glyceraldehyde-3-phosphate dehydrogenase	*Gapdh*	-2.1396	0.0007
90	Q99K85	Phosphoserine aminotransferase	*Psat1*	-2.1336	0.0007
91	Q8R164	Valacyclovir hydrolase	*Bphl*	-2.1217	0.0007
92	P03995	Glial fibrillary acidic protein	*Gfap*	-2.1177	0.0007
93	Q9CQA3	Succinate dehydrogenase [ubiquinone] iron-sulfur subunit mitochondrial	*Sdhb*	-2.1091	0.0007
94	Q9CQ65	S-methyl-5'-thioadenosine phosphorylase	*Mtap*	-2.1022	0.0007
95	Q9D338	39S ribosomal protein L19 mitochondrial	*Mrpl19*	-2.1022	0.0007
96	Q9D710	Thioredoxin-related transmembrane protein 2	*Tmx2*	-2.0884	0.0008
97	Q9JJZ2	Tubulin alpha-8 chain	*Tuba8*	-2.0794	0.0008
98	P39061	Collagen alpha-1(XVIII) chain	*Col18a1*	-2.0553	0.0008
99	P48774	Glutathione S-transferase Mu 5	*Gstm5*	-2.0474	0.0008
100	P13808	Anion exchange protein 2	*Slc4a2*	-2.0371	0.0008
101	P01638	Ig kappa chain V-V region L6 (Fragment)	*4 SV*	-2.0371	0.0008
102	P01637	Ig kappa chain V-V region T1	*4 SV*	-2.0371	0.0008
103	Q91V41	Ras-related protein Rab-14	*Rab14*	-2.0267	0.0008
104	P48758	Carbonyl reductase [NADPH] 1	*Cbr1*	-2.0157	0.0009
105	P10605	Cathepsin B	*Ctsb*	-2.0081	0.0009
106	Q9CR00	26S proteasome non-ATPase regulatory subunit 9	*Psmd9*	-2.0048	0.0009
107	Q9QUM9	Proteasome subunit alpha type-6	*Psma6*	-1.9979	0.0009
108	P62897	Cytochrome c somatic	*Cycs*	-1.9974	0.0009
109	Q8BHB9	Chloride intracellular channel protein 6	*Clic6*	-1.9942	0.0009
110	P23242	Gap junction alpha-1 protein	*Gja1*	-1.9862	0.0009
111	O35143	ATPase inhibitor mitochondrial	*Atp5if1*	-1.9861	0.0009
112	Q64105	Sepiapterin reductase	*Spr*	-1.9855	0.0009
113	P20444	Protein kinase C alpha type	*Prkca*	-1.9847	0.0009
114	P62748	Hippocalcin-like protein 1	*Hpcal1*	-1.9794	0.0009
115	Q9D7X1	BTB/POZ domain-containing protein KCTD4	*Kctd4*	-1.9764	0.0009
116	P01837	Immunoglobulin kappa constant	*Igkc*	-1.9764	0.0009
117	Q9CZU6	Citrate synthase mitochondrial	*Cs*	-1.9697	0.0009
118	P63328	Serine/threonine-protein phosphatase 2B catalytic subunit alpha isoform	*Ppp3ca*	-1.9689	0.0009
119	P06151	L-lactate dehydrogenase A chain	*Ldha*	-1.9592	0.0010
120	Q8VEL9	GTP-binding protein REM 2	*Rem2*	-1.9546	0.0010
121	Q99KK2	N-acylneuraminate cytidylyltransferase	*Cmas*	-1.9501	0.0010
122	O35459	Delta(3 5)-Delta(2 4)-dienoyl-CoA isomerase mitochondrial	*Ech1*	-1.9482	0.0010
123	Q8BRF7	Sec1 family domain-containing protein 1	*Scfd1*	-1.9244	0.0010
124	P23819	Glutamate receptor 2	*Gria2*	-1.9119	0.0011
125	P59281	Rho GTPase-activating protein 39	*Arhgap39*	-1.9087	0.0011
126	Q9D6G9	CKLF-like MARVEL transmembrane domain-containing protein 5	*Cmtm5*	-1.9049	0.0011
127	P97447	Four and a half LIM domains protein 1	*Fhl1*	-1.9004	0.0011
128	P05202	Aspartate aminotransferase mitochondrial	*Got2*	-1.8966	0.0011
129	Q3UQ44	Ras GTPase-activating-like protein IQGAP2	*Iqgap2*	-1.8745	0.0011
130	P09470	Angiotensin-converting enzyme	*Ace*	-1.8683	0.0011
131	P70261	Paladin	*Pald1*	-1.8668	0.0011
132	Q9DB26	Phytanoyl-CoA dioxygenase domain-containing protein 1	*Phyhd1*	-1.8668	0.0011
133	Q99JY9	Actin-related protein 3	*Actr3*	-1.8643	0.0011
134	Q9CVB6	Actin-related protein 2/3 complex subunit 2	*Arpc2*	-1.8621	0.0011
135	Q61414	Keratin type I cytoskeletal 15	*Krt15*	-1.8615	0.0011
136	Q80ZJ1	Ras-related protein Rap-2a	*Rap2a*	-1.8613	0.0011
137	Q8BK63	Casein kinase I isoform alpha	*Csnk1a1*	-1.8498	0.0011
138	Q3UMR5	Calcium uniporter protein mitochondrial	*Mcu*	-1.8498	0.0011
139	P63321	Ras-related protein Ral-A	*Rala*	-1.8489	0.0011
140	Q3TRR0	Microtubule-associated protein 9	*Map9*	-1.8341	0.0012
141	Q9D8Y1	Transmembrane protein 126A	*Tmem126a*	-1.8341	0.0012
142	A2ALK8	Tyrosine-protein phosphatase non-receptor type 3	*Ptpn3*	-1.8329	0.0012
143	Q9WV68	Peroxisomal 2 4-dienoyl-CoA reductase [(3E)-enoyl-CoA-producing]	*Decr2*	-1.8303	0.0012
144	Q02013	Aquaporin-1	*Aqp1*	-1.8303	0.0012
145	Q8R059	UDP-glucose 4-epimerase	*Gale*	-1.8301	0.0012
146	Q9D287	Pre-mRNA-splicing factor SPF27	*Bcas2*	-1.8215	0.0012
147	Q5SYL3	Protein KIAA0100	*Kiaa0100*	-1.8215	0.0012
148	Q9CQR4	Acyl-coenzyme A thioesterase 13	*Acot13*	-1.8191	0.0012
149	Q8VDD5	Myosin-9	*Myh9*	-1.8113	0.0012
150	P41233	Phospholipid-transporting ATPase ABCA1	*Abca1*	-1.8103	0.0012
151	P55012	Solute carrier family 12 member 2	*Slc12a2*	-1.8040	0.0012
152	Q8CIZ8	von Willebrand factor	*Vwf*	-1.8021	0.0012
153	Q9JLC8	Sacsin	*Sacs*	-1.8015	0.0012
154	P01878	Ig alpha chain C region		-1.8007	0.0012
155	O09131	Glutathione S-transferase omega-1	*Gsto1*	-1.7987	0.0012
156	Q8BMF4	Dihydrolipoyllysine-residue acetyltransferase component of pyruvate dehydrogenase complex mitochondrial	*Dlat*	-1.7925	0.0013

157	P63242	Eukaryotic translation initiation factor 5A-1	*Eif5a*	-1.7912	0.0013
158	O88545	COP9 signalosome complex subunit 6	*Cops6*	-1.7911	0.0013
159	P47754	F-actin-capping protein subunit alpha-2	*Capza2*	-1.7886	0.0013
160	Q9WV55	Vesicle-associated membrane protein-associated protein A	*Vapa*	-1.7864	0.0013
161	Q5U4C1	G-protein coupled receptor-associated sorting protein 1	*Gprasp1*	-1.7810	0.0013
162	Q8K0E8	Fibrinogen beta chain	*Fgb*	-1.7783	0.0013
163	Q8CGF6	WD repeat-containing protein 47	*Wdr47*	-1.7666	0.0014
164	P62823	Ras-related protein Rab-3C	*Rab3c*	-1.7516	0.0014
165	P62259	14-3-3 protein epsilon	*Ywhae*	-1.7512	0.0014
166	P62754	40S ribosomal protein S6	*Rps6*	-1.7469	0.0014
167	P52825	Carnitine O-palmitoyltransferase 2 mitochondrial	*Cpt2*	-1.7412	0.0015
168	P34022	Ran-specific GTPase-activating protein	*Ranbp1*	-1.7345	0.0015
169	Q9DAM5	Mitochondrial thiamine pyrophosphate carrier	*Slc25a19*	-1.7302	0.0015
170	Q8VCM7	Fibrinogen gamma chain	*Fgg*	-1.7289	0.0015
171	Q99JP6	Homer protein homolog 3	*Homer3*	-1.7289	0.0015
172	Q9CQ75	NADH dehydrogenase [ubiquinone] 1 alpha subcomplex subunit 2	*Ndufa2*	-1.7253	0.0015
173	Q9JM52	Misshapen-like kinase 1	*Mink1*	-1.7167	0.0015
174	P01867	Ig gamma-2B chain C region	*Igh-3*	-1.7116	0.0015
175	Q8BVI4	Dihydropteridine reductase	*Qdpr*	-1.6984	0.0015
176	P68369	Tubulin alpha-1A chain	*Tuba1a*	-1.6971	0.0015
177	Q9D0M3	Cytochrome c1 heme protein mitochondrial	*Cyc1*	-1.6933	0.0016
178	O54991	Contactin-associated protein 1	*Cntnap1*	-1.6921	0.0016
179	P58871	182 kDa tankyrase-1-binding protein	*Tnks1bp1*	-1.6915	0.0016
180	Q9WTU3	Sodium channel protein type 8 subunit alpha	*Scn8a*	-1.6850	0.0016
181	P14152	Malate dehydrogenase cytoplasmic	*Mdh1*	-1.6817	0.0016
182	P63087	Serine/threonine-protein phosphatase PP1-gamma catalytic subunit	*Ppp1cc*	-1.6812	0.0016
183	P05213	Tubulin alpha-1B chain	*Tuba1b*	-1.6739	0.0016
184	Q8VDQ8	NAD-dependent protein deacetylase sirtuin-2	*Sirt2*	-1.6690	0.0016
185	P25785	Metalloproteinase inhibitor 2	*Timp2*	-1.6689	0.0016
186	Q3ZK22	Vezatin	*Vezt*	-1.6689	0.0016
187	P52840	Sulfotransferase 1A1	*Sult1a1*	-1.6689	0.0016
188	Q8BZ60	Stonin-2	*Ston2*	-1.6663	0.0016
189	Q91V64	Isochorismatase domain-containing protein 1	*Isoc1*	-1.6606	0.0016
190	Q99LS3	Phosphoserine phosphatase	*Psph*	-1.6606	0.0016
191	Q9CZB0	Succinate dehydrogenase cytochrome b560 subunit mitochondrial	*Sdhc*	-1.6460	0.0017
192	Q8BMS1	Trifunctional enzyme subunit alpha mitochondrial	*Hadha*	-1.6447	0.0017
193	P62307	Small nuclear ribonucleoprotein F	*Snrpf*	-1.6386	0.0017
194	Q920N7	Synaptotagmin-12	*Syt12*	-1.6375	0.0017
195	P63260	Actin cytoplasmic 2	*Actg1*	-1.6339	0.0017
196	P49615	Cyclin-dependent-like kinase 5	*Cdk5*	-1.6330	0.0017
197	P68373	Tubulin alpha-1C chain	*Tuba1c*	-1.6299	0.0017
198	Q6WKZ8	E3 ubiquitin-protein ligase UBR2	*Ubr2*	-1.6218	0.0017
199	Q62418	Drebrin-like protein	*Dbnl*	-1.6209	0.0017
200	Q80X95	Ras-related GTP-binding protein A	*Rraga*	-1.6148	0.0017
201	Q9R0P9	Ubiquitin carboxyl-terminal hydrolase isozyme L1	*Uchl1*	-1.6145	0.0017
202	Q5SWU9	Acetyl-CoA carboxylase 1	*Acaca*	-1.6136	0.0017
203	Q8CI71	Syndetin	*Vps50*	-1.6054	0.0018
204	Q3UNZ8	Quinone oxidoreductase-like protein 2	*Cryzl2*	-1.6039	0.0018
205	P58771	Tropomyosin alpha-1 chain	*Tpm1*	-1.5987	0.0018
206	Q9R112	Sulfide:quinone oxidoreductase mitochondrial	*Sqor*	-1.5927	0.0018
207	P18526	Ig heavy chain V region 345		-1.5912	0.0018
208	P70407	Cadherin-9	*Cdh9*	-1.5912	0.0018
209	Q64374	Regucalcin	*Rgn*	-1.5912	0.0018
210	P62700	Protein yippee-like 5	*Ypel5*	-1.5912	0.0018
211	Q9CR62	Mitochondrial 2-oxoglutarate/malate carrier protein	*Slc25a11*	-1.5881	0.0019
212	Q91XE4	N-acyl-aromatic-L-amino acid amidohydrolase (carboxylate-forming)	*Acy3*	-1.5813	0.0019
213	Q6X893	Choline transporter-like protein 1	*Slc44a1*	-1.5783	0.0019
214	Q9WV96	Mitochondrial import inner membrane translocase subunit Tim10 B	*Timm10b*	-1.5735	0.0019
215	Q9JII6	Aldo-keto reductase family 1 member A1	*Akr1a1*	-1.5694	0.0019
216	Q8BKN5	Gamma-tubulin complex component 5	*Tubgcp5*	-1.5680	0.0019
217	A2APX8	Sodium channel protein type 1 subunit alpha	*Scn1a*	-1.5655	0.0019
218	Q9DB16	Calcium-binding protein 39-like	*Cab39l*	-1.5648	0.0019
219	P07309	Transthyretin	*Ttr*	-1.5614	0.0019
220	Q99JR5	Tubulointerstitial nephritis antigen-like	*Tinagl1*	-1.5609	0.0019
221	Q91WT9	Cystathionine beta-synthase	*Cbs*	-1.5580	0.0020
222	Q99LB6	Methionine adenosyltransferase 2 subunit beta	*Mat2b*	-1.5544	0.0020
223	Q3UHB1	5'-nucleotidase domain-containing protein 3	*Nt5dc3*	-1.5524	0.0020
224	P70336	Rho-associated protein kinase 2	*Rock2*	-1.5509	0.0020
225	Q8K3J1	NADH dehydrogenase [ubiquinone] iron-sulfur protein 8 mitochondrial	*Ndufs8*	-1.5444	0.0020
226	Q80U35	Rho guanine nucleotide exchange factor 17	*Arhgef17*	-1.5415	0.0020
227	O08677	Kininogen-1	*Kng1*	-1.5403	0.0020
228	P63082	V-type proton ATPase 16 kDa proteolipid subunit	*Atp6v0c*	-1.5374	0.0020
229	Q1RLL3	Copine-9	*Cpne9*	-1.5365	0.0020
230	P29387	Guanine nucleotide-binding protein subunit beta-4	*Gnb4*	-1.5291	0.0021
231	Q9CQ69	Cytochrome b-c1 complex subunit 8	*Uqcrq*	-1.5266	0.0021
232	P23492	Purine nucleoside phosphorylase	*Pnp*	-1.5261	0.0021
233	P11835	Integrin beta-2	*Itgb2*	-1.5244	0.0021
234	Q9JJ69	Kv channel-interacting protein 2	*Kcnip2*	-1.5244	0.0021
235	Q99K23	Ufm1-specific protease 2	*Ufsp2*	-1.5202	0.0021

236	Q9CWE0	Mitochondrial fission regulator 1-like	*Mtfr1l*	-1.5145	0.0022
237	O70250	Phosphoglycerate mutase 2	*Pgam2*	-1.5142	0.0022
238	Q641K1	Cytosolic carboxypeptidase 1	*Agtpbp1*	-1.5138	0.0022
239	Q5SYD0	Unconventional myosin-Id	*Myo1d*	-1.5123	0.0022
240	Q9CQV8	14-3-3 protein beta/alpha	*Ywhab*	-1.5120	0.0022
241	P60710	Actin cytoplasmic 1	*Actb*	-1.5119	0.0022
242	P02535	Keratin type I cytoskeletal 10	*Krt10*	-1.5102	0.0022
243	P70335	Rho-associated protein kinase 1	*Rock1*	-1.5093	0.0022
244	P97384	Annexin A11	*Anxa11*	-1.5087	0.0022
245	Q91WG7	Diacylglycerol kinase gamma	*Dgkg*	-1.5052	0.0022
246	P19157	Glutathione S-transferase P 1	*Gstp1*	-1.5014	0.0022
247	B1AXH1	NHS-like protein 2	*Nhsl2*	-1.4957	0.0023
248	Q7M759	Alpha/beta hydrolase domain-containing protein 17B	*Abhd17b*	-1.4938	0.0023
249	Q9R1S8	Calpain-7	*Capn7*	-1.4938	0.0023
250	Q9CWD3	Nucleoside diphosphate-linked moiety X motif 17	*Nudt17*	-1.4938	0.0023
251	Q9EST4	Proteasome assembly chaperone 2	*Psmg2*	-1.4938	0.0023
252	Q3V4B5	COMM domain-containing protein 6	*Commd6*	-1.4938	0.0023
253	Q6NZK8	Protein tyrosine phosphatase domain-containing protein 1	*Ptpdc1*	-1.4938	0.0023
254	Q925N0	Sideroflexin-5	*Sfxn5*	-1.4931	0.0023
255	Q9DC53	Copine-8	*Cpne8*	-1.4876	0.0023
256	P70206	Plexin-A1	*Plxna1*	-1.4872	0.0023
257	O89112	Glutathione S-transferase LANCL1	*Lancl1*	-1.4860	0.0023
258	Q60994	Adiponectin	*Adipoq*	-1.4851	0.0023
259	P28828	Receptor-type tyrosine-protein phosphatase mu	*Ptprm*	-1.4851	0.0023
260	P15105	Glutamine synthetase	*Glul*	-1.4824	0.0023
261	Q8BW96	Calcium/calmodulin-dependent protein kinase type 1D	*Camk1d*	-1.4814	0.0023
262	Q8BGY7	Protein FAM210A	*Fam210a*	-1.4807	0.0023
263	P17182	Alpha-enolase	*Eno1*	-1.4794	0.0024
264	P62874	Guanine nucleotide-binding protein G(I)/G(S)/G(T) subunit beta-1	*Gnb1*	-1.4755	0.0024
265	Q9JJ61	Polypeptide N-acetylgalactosaminyltransferase 16	*Galnt16*	-1.4740	0.0024
266	P01654	Ig kappa chain V-III region PC 2880/PC 1229		-1.4740	0.0024
267	P01656	Ig kappa chain V-III region MOPC 70		-1.4740	0.0024
268	O35668	Huntingtin-associated protein 1	*Hap1*	-1.4740	0.0024
269	Q8BLS7	Ankyrin repeat domain-containing protein SOWAHA	*Sowaha*	-1.4740	0.0024
270	Q80ZW2	Protein THEM6	*Them6*	-1.4669	0.0025
271	Q80TL0	Protein phosphatase 1E	*Ppm1e*	-1.4659	0.0025
272	Q68SA9	A disintegrin and metalloproteinase with thrombospondin motifs 7	*Adamts7*	-1.4650	0.0025
273	Q8R0F8	Acylpyruvase FAHD1 mitochondrial	*Fahd1*	-1.4650	0.0025
274	Q9D1G1	Ras-related protein Rab-1B	*Rab1b*	-1.4644	0.0025
275	O08553	Dihydropyrimidinase-related protein 2	*Dpysl2*	-1.4640	0.0025
276	Q03265	ATP synthase subunit alpha mitochondrial	*Atp5f1a*	-1.4627	0.0025
277	P63101	14-3-3 protein zeta/delta	*Ywhaz*	-1.4607	0.0025
278	P17751	Triosephosphate isomerase	*Tpi1*	-1.4607	0.0025
279	P53808	Phosphatidylcholine transfer protein	*Pctp*	-1.4595	0.0025
280	P00015	Cytochrome c testis-specific	*Cyct*	-1.4595	0.0025
281	Q62288	Testican-1	*Spock1*	-1.4595	0.0025
282	Q9CQD1	Ras-related protein Rab-5A	*Rab5a*	-1.4595	0.0025
283	Q9D2P8	Myelin-associated oligodendrocyte basic protein	*Mobp*	-1.4587	0.0025
284	Q571B6	WASP homolog-associated protein with actin membranes and microtubules	*Whamm*	-1.4585	0.0025
285	P28663	Beta-soluble NSF attachment protein	*Napb*	-1.4583	0.0025
286	Q8CI94	Glycogen phosphorylase brain form	*Pygb*	-1.4553	0.0026
287	Q8BWU8	Ethanolamine-phosphate phospho-lyase	*Etnppl*	-1.4552	0.0026
288	P17225	Polypyrimidine tract-binding protein 1	*Ptbp1*	-1.4551	0.0026
289	Q9CQI6	Coactosin-like protein	*Cotl1*	-1.4547	0.0026
290	O55023	Inositol monophosphatase 1	*Impa1*	-1.4535	0.0026
291	Q9QZF2	Glypican-1	*Gpc1*	-1.4513	0.0026
292	P56371	Ras-related protein Rab-4A	*Rab4a*	-1.4496	0.0026
293	Q3UJP5	Protein C8orf37 homolog		-1.4489	0.0026
294	Q9DCC8	Mitochondrial import receptor subunit TOM20 homolog	*Tomm20*	-1.4489	0.0026
295	Q9CTY5	Calcium uptake protein 3 mitochondrial	*Micu3*	-1.4424	0.0027
296	Q64378	Peptidyl-prolyl cis-trans isomerase FKBP5	*Fkbp5*	-1.4393	0.0027
297	Q8BMS4	Ubiquinone biosynthesis O-methyltransferase mitochondrial	*Coq3*	-1.4393	0.0027
298	P62137	Serine/threonine-protein phosphatase PP1-alpha catalytic subunit	*Ppp1ca*	-1.4331	0.0027
299	P08551	Neurofilament light polypeptide	*Nefl*	-1.4326	0.0027
300	B2RUR8	OTU domain-containing protein 7B	*Otud7b*	-1.4268	0.0027
301	P70248	Unconventional myosin-If	*Myo1f*	-1.4202	0.0030
302	Q8K382	DENN domain-containing protein 1A	*Dennd1a*	-1.4202	0.0030
303	P70302	Stromal interaction molecule 1	*Stim1*	-1.4195	0.0030
304	P61021	Ras-related protein Rab-5B	*Rab5b*	-1.4158	0.0030
305	P23818	Glutamate receptor 1	*Gria1*	-1.4146	0.0031
306	Q8BT60	Copine-3	*Cpne3*	-1.4086	0.0031
307	P09528	Ferritin heavy chain	*Fth1*	-1.4044	0.0031
308	P56382	ATP synthase subunit epsilon mitochondrial	*Atp5f1e*	-1.3973	0.0032
309	Q9CXS4	Centromere protein V	*Cenpv*	-1.3971	0.0032
310	P97333	Neuropilin-1	*Nrp1*	-1.3964	0.0032
311	O70152	Dolichol-phosphate mannosyltransferase subunit 1	*Dpm1*	-1.3964	0.0032
312	Q9DCZ1	GMP reductase 1	*Gmpr*	-1.3918	0.0032
313	Q8BHS6	Armadillo repeat-containing X-linked protein 3	*Armcx3*	-1.3893	0.0033
314	P83093	Stromal interaction molecule 2	*Stim2*	-1.3864	0.0033

315	Q9CQS8	Protein transport protein Sec61 subunit beta	*Sec61b*	-1.3806	0.0033
316	Q9Z1P6	NADH dehydrogenase [ubiquinone] 1 alpha subcomplex subunit 7	*Ndufa7*	-1.3792	0.0033
317	F6SEU4	Ras/Rap GTPase-activating protein SynGAP	*Syngap1*	-1.3752	0.0034
318	Q9D1G5	Leucine-rich repeat-containing protein 57	*Lrrc57*	-1.3750	0.0034
319	G3XA57	Rab11 family-interacting protein 2	*Rab11fip2*	-1.3726	0.0034
320	P16125	L-lactate dehydrogenase B chain	*Ldhb*	-1.3674	0.0035
321	Q8C0M9	Isoaspartyl peptidase/L-asparaginase	*Asrgl1*	-1.3654	0.0035
322	Q5PR73	GTP-binding protein Di-Ras2	*Diras2*	-1.3613	0.0035
323	Q60866	Phosphotriesterase-related protein	*Pter*	-1.3598	0.0035
324	Q68ED7	CREB-regulated transcription coactivator 1	*Crtc1*	-1.3598	0.0035
325	Q8BLR2	Copine-4	*Cpne4*	-1.3581	0.0035
326	Q8K298	Anillin	*Anln*	-1.3562	0.0035
327	Q6WQJ1	Diacylglycerol lipase-alpha	*Dagla*	-1.3557	0.0036
328	Q8VE09	Tetratricopeptide repeat protein 39C	*Ttc39c*	-1.3552	0.0036
329	Q99L27	GMP reductase 2	*Gmpr2*	-1.3552	0.0036
330	Q9CPQ1	Cytochrome c oxidase subunit 6C	*Cox6c*	-1.3493	0.0036
331	P56528	ADP-ribosyl cyclase/cyclic ADP-ribose hydrolase 1	*Cd38*	-1.3485	0.0036
332	Q60770	Syntaxin-binding protein 3	*Stxbp3*	-1.3465	0.0036
333	Q61166	Microtubule-associated protein RP/EB family member 1	*Mapre1*	-1.3450	0.0036
334	P61226	Ras-related protein Rap-2b	*Rap2b*	-1.3426	0.0037
335	Q9D7X3	Dual specificity protein phosphatase 3	*Dusp3*	-1.3419	0.0037
336	Q8C5W0	Calmin	*Clmn*	-1.3419	0.0037
337	Q5U5V2	Hydroxylysine kinase	*Hykk*	-1.3419	0.0037
338	Q69Z38	Inactive tyrosine-protein kinase PEAK1	*Peak1*	-1.3419	0.0037
339	Q9JM14	5'(3')-deoxyribonucleotidase cytosolic type	*Nt5c*	-1.3419	0.0037
340	Q8BZJ7	DCN1-like protein 2	*Dcun1d2*	-1.3419	0.0037
341	Q9QUQ5	Short transient receptor potential channel 4	*Trpc4*	-1.3419	0.0037
342	Q6ZWM4	U6 snRNA-associated Sm-like protein LSm8	*Lsm8*	-1.3419	0.0037
343	O08601	Microsomal triglyceride transfer protein large subunit	*Mttp*	-1.3419	0.0037
344	Q8BJL0	SWI/SNF-related matrix-associated actin-dependent regulator of chromatin subfamily A-like protein 1	*Smarcal1*	-1.3419	0.0037
345	P62880	Guanine nucleotide-binding protein G(I)/G(S)/G(T) subunit beta-2	*Gnb2*	-1.3393	0.0037
346	O54940	BCL2/adenovirus E1B 19 kDa protein-interacting protein 2	*Bnip2*	-1.3374	0.0037
347	O70325	Phospholipid hydroperoxide glutathione peroxidase	*Gpx4*	-1.3327	0.0039
348	Q6WVG3	BTB/POZ domain-containing protein KCTD12	*Kctd12*	-1.3315	0.0040
349	Q6NTA4	Ras-related GTP-binding protein B	*Rragb*	-1.3307	0.0040
350	Q6NVE8	WD repeat-containing protein 44	*Wdr44*	-1.3277	0.0040
351	P21447	ATP-dependent translocase ABCB1	*Abcb1a*	-1.3214	0.0040
352	Q9QZ23	NFU1 iron-sulfur cluster scaffold homolog mitochondrial	*Nfu1*	-1.3213	0.0040
353	P62806	Histone H4	*H4c1*	-1.3134	0.0041
354	Q9CPV4	Glyoxalase domain-containing protein 4	*Glod4*	-1.3119	0.0042
355	P62835	Ras-related protein Rap-1A	*Rap1a*	-1.3116	0.0042
356	E9Q555	E3 ubiquitin-protein ligase RNF213	*Rnf213*	-1.3084	0.0042
357	P10922	Histone H1.0	*H1-0*	-1.3062	0.0043
358	Q9CXI0	2-methoxy-6-polyprenyl-1 4-benzoquinol methylase mitochondrial	*Coq5*	-1.3034	0.0043
359	P01942	Hemoglobin subunit alpha	*Hba*	-1.3025	0.0043
360	P97371	Proteasome activator complex subunit 1	*Psme1*	-1.2952	0.0044
361	Q80U87	Ubiquitin carboxyl-terminal hydrolase 8	*Usp8*	-1.2946	0.0044
362	Q3TCN2	Putative phospholipase B-like 2	*Plbd2*	-1.2916	0.0044
363	Q8C1B1	Calmodulin-regulated spectrin-associated protein 2	*Camsap2*	-1.2915	0.0044
364	Q5HZI9	Mitochondrial nicotinamide adenine dinucleotide transporter SLC25A51	*Slc25a51*	-1.2915	0.0044
365	P01592	Immunoglobulin J chain	*Jchain*	-1.2907	0.0044
366	P01630	Ig kappa chain V-II region 7S34.1		-1.2907	0.0044
367	Q8BHI4	Kelch repeat and BTB domain-containing protein 3	*Kbtbd3*	-1.2907	0.0044
368	Q8BTF8	RNA-binding Raly-like protein	*Ralyl*	-1.2907	0.0044
369	Q99JR6	Nicotinamide/nicotinic acid mononucleotide adenylyltransferase 3	*Nmnat3*	-1.2907	0.0044
370	Q99J47	Dehydrogenase/reductase SDR family member 7B	*Dhrs7b*	-1.2905	0.0044
371	P20152	Vimentin	*Vim*	-1.2902	0.0044
372	Q9QZD8	Mitochondrial dicarboxylate carrier	*Slc25a10*	-1.2881	0.0044
373	Q9JKC6	Cell cycle exit and neuronal differentiation protein 1	*Cend1*	-1.2870	0.0044
374	Q8BNW9	Kelch repeat and BTB domain-containing protein 11	*Kbtbd11*	-1.2861	0.0045
375	P32261	Antithrombin-III	*Serpinc1*	-1.2849	0.0045
376	Q91XC9	Peroxisomal membrane protein PEX16	*Pex16*	-1.2849	0.0045
377	P54869	Hydroxymethylglutaryl-CoA synthase mitochondrial	*Hmgcs2*	-1.2849	0.0045
378	Q924T2	28S ribosomal protein S2 mitochondrial	*Mrps2*	-1.2849	0.0045
379	Q91YY4	ATP synthase mitochondrial F1 complex assembly factor 2	*Atpaf2*	-1.2849	0.0045
380	Q9WUP7	Ubiquitin carboxyl-terminal hydrolase isozyme L5	*Uchl5*	-1.2830	0.0045
381	Q91VR2	ATP synthase subunit gamma mitochondrial	*Atp5f1c*	-1.2812	0.0045
382	Q9D880	Mitochondrial import inner membrane translocase subunit TIM50	*Timm50*	-1.2793	0.0045
383	P26443	Glutamate dehydrogenase 1 mitochondrial	*Glud1*	-1.2734	0.0046
384	Q9ERI6	Retinol dehydrogenase 14	*Rdh14*	-1.2724	0.0046
385	Q9QWL7	Keratin type I cytoskeletal 17	*Krt17*	-1.2691	0.0047
386	Q9QYE9	Pleckstrin homology domain-containing family B member 1	*Plekhb1*	-1.2683	0.0047
387	P05532	Mast/stem cell growth factor receptor Kit	*Kit*	-1.2677	0.0047
388	Q8CGP5	Histone H2A type 1-F	*Hist1h2af*	-1.2674	0.0048
389	Q8CBW3	Abl interactor 1	*Abi1*	-1.2654	0.0048
390	Q8R2Y8	Peptidyl-tRNA hydrolase 2 mitochondrial	*Ptrh2*	-1.2623	0.0049
391	Q9ES46	Beta-parvin	*Parvb*	-1.2623	0.0049
392	Q8K2T1	NmrA-like family domain-containing protein 1	*Nmral1*	-1.2615	0.0049
393	Q9EQ06	Estradiol 17-beta-dehydrogenase 11	*Hsd17b11*	-1.2615	0.0049

394	Q9DC07	LIM zinc-binding domain-containing Nebulette	*Nebl*	-1.2582	0.0049
395	P18242	Cathepsin D	*Ctsd*	-1.2579	0.0049
396	P68368	Tubulin alpha-4A chain	*Tuba4a*	-1.2559	0.0049
397	Q8CHU3	Epsin-2	*Epn2*	-1.2545	0.0050
398	Q8JZL3	Thiamine-triphosphatase	*Thtpa*	-1.2528	0.0050
399	P70280	Vesicle-associated membrane protein 7	*Vamp7*	-1.2528	0.0050
400	Q8BU31	Ras-related protein Rap-2c	*Rap2c*	-1.2501	0.0050
401	Q8CGP7	Histone H2A type 1-K	*H2ac15*	-1.2491	0.0050
402	P39053	Dynamin-1	*Dnm1*	-1.2465	0.0050
403	P05214	Tubulin alpha-3 chain	*Tuba3a*	-1.2454	0.0050
404	A1L317	Keratin type I cytoskeletal 24	*Krt24*	-1.2434	0.0050
405	Q9Z2M6	Ubiquitin-like protein 3	*Ubl3*	-1.2421	0.0050
406	P40237	CD82 antigen	*Cd82*	-1.2398	0.0051
407	P18525	Ig heavy chain V region 5-84		-1.2360	0.0052
408	Q9D0K0	TBC1 domain family member 7	*Tbc1d7*	-1.2360	0.0052
409	Q8C0D4	Rho GTPase-activating protein 12	*Arhgap12*	-1.2360	0.0052
410	Q8BWY4	Metallo-beta-lactamase domain-containing protein 1	*Mblac1*	-1.2360	0.0052
411	Q6SKR2	Methyltransferase N6AMT1	*N6amt1*	-1.2360	0.0052
412	Q810B9	SLIT and NTRK-like protein 3	*Slitrk3*	-1.2360	0.0052
413	Q9CQX0	Ubiquitin thioesterase OTUB2	*Otub2*	-1.2360	0.0052
414	Q69ZH9	Rho GTPase-activating protein 23	*Arhgap23*	-1.2349	0.0053
415	Q4V9Z5	Seizure 6-like protein 2	*Sez6l2*	-1.2254	0.0054
416	Q8R4U7	Leucine zipper protein 1	*Luzp1*	-1.2238	0.0055
417	Q62425	Cytochrome c oxidase subunit NDUFA4	*Ndufa4*	-1.2219	0.0055
418	Q61781	Keratin type I cytoskeletal 14	*Krt14*	-1.2215	0.0055
419	Q9JIK9	28S ribosomal protein S34 mitochondrial	*Mrps34*	-1.2178	0.0057
420	Q8CIQ7	Dedicator of cytokinesis protein 3	*Dock3*	-1.2159	0.0057
421	P46471	26S proteasome regulatory subunit 7	*Psmc2*	-1.2147	0.0057
422	Q9JI91	Alpha-actinin-2	*Actn2*	-1.2137	0.0057
423	Q8BLY3	Leucine-rich repeat and fibronectin type-III domain-containing protein 3	*Lrfn3*	-1.2107	0.0058
424	Q8BWR2	PITH domain-containing protein 1	*Pithd1*	-1.2102	0.0058
425	P49070	Guided entry of tail-anchored proteins factor CAMLG	*Camlg*	-1.2041	0.0059
426	P01633	Ig kappa chain V19-17	*Igk-V19-17*	-1.2041	0.0059
427	P28474	Alcohol dehydrogenase class-3	*Adh5*	-1.2032	0.0059
428	P17427	AP-2 complex subunit alpha-2	*Ap2a2*	-1.2021	0.0059
429	Q8VHW2	Voltage-dependent calcium channel gamma-8 subunit	*Cacng8*	-1.2011	0.0059
430	Q3UV17	Keratin type II cytoskeletal 2 oral	*Krt76*	-1.2005	0.0059
431	P56391	Cytochrome c oxidase subunit 6B1	*Cox6b1*	-1.2000	0.0060
432	Q91VH6	Protein MEMO1	*Memo1*	-1.1977	0.0060
433	P35278	Ras-related protein Rab-5C	*Rab5c*	-1.1968	0.0060
434	Q9CX00	IST1 homolog	*Ist1*	-1.1948	0.0060
435	P35276	Ras-related protein Rab-3D	*Rab3d*	-1.1946	0.0060
436	Q8K0T0	Reticulon-1	*Rtn1*	-1.1932	0.0060
437	O70194	Eukaryotic translation initiation factor 3 subunit D	*Eif3d*	-1.1870	0.0071
438	O35066	Kinesin-like protein KIF3C	*Kif3c*	-1.1870	0.0071
439	Q80U19	Disheveled-associated activator of morphogenesis 2	*Daam2*	-1.1870	0.0071
440	P23953	Carboxylesterase 1C	*Ces1c*	-1.1870	0.0071
441	Q9CPT3	N-acylneuraminate-9-phosphatase	*Nanp*	-1.1870	0.0071
442	P06909	Complement factor H	*Cfh*	-1.1861	0.0071
443	Q9DAS9	Guanine nucleotide-binding protein G(I)/G(S)/G(O) subunit gamma-12	*Gng12*	-1.1861	0.0071
444	Q8R0P4	Mth938 domain-containing protein	*Aamdc*	-1.1861	0.0071
445	P35282	Ras-related protein Rab-21	*Rab21*	-1.1852	0.0072
446	Q80YA9	Connector enhancer of kinase suppressor of ras 2	*Cnksr2*	-1.1796	0.0075
447	Q504M8	Ras-related protein Rab-26	*Rab26*	-1.1794	0.0075
448	Q6ZQI3	Malectin	*Mlec*	-1.1778	0.0075
449	O70503	Very-long-chain 3-oxoacyl-CoA reductase	*Hsd17b12*	-1.1762	0.0075
450	P27671	Ras-specific guanine nucleotide-releasing factor 1	*Rasgrf1*	-1.1752	0.0076
451	Q8R5I6	Glutathione S-transferase Mu 4	*Gstm4*	-1.1751	0.0076
452	Q3UFT3	GRB2-associated and regulator of MAPK protein	*Garem1*	-1.1741	0.0076
453	P97785	GDNF family receptor alpha-1	*Gfra1*	-1.1741	0.0076
454	Q8BML1	[F-actin]-monooxygenase MICAL2	*Mical2*	-1.1741	0.0076
455	P27046	Alpha-mannosidase 2	*Man2a1*	-1.1741	0.0076
456	Q99LN9	Deoxyhypusine hydroxylase	*Dohh*	-1.1741	0.0076
457	Q505B7	Protein archease	*Zbtb8os*	-1.1741	0.0076
458	P70451	Tyrosine-protein kinase Fer	*Fer*	-1.1741	0.0076
459	Q9R0E1	Multifunctional procollagen lysine hydroxylase and glycosyltransferase LH3	*Plod3*	-1.1741	0.0076
460	P70245	3-beta-hydroxysteroid-Delta(8) Delta(7)-isomerase	*Ebp*	-1.1741	0.0076
461	Q3U3W5	Protein arginine N-methyltransferase 9	*Prmt9*	-1.1741	0.0076
462	P68404	Protein kinase C beta type	*Prkcb*	-1.1734	0.0076
463	O35393	Ephrin-B3	*Efnb3*	-1.1734	0.0076
464	Q9JLZ3	Methylglutaconyl-CoA hydratase mitochondrial	*Auh*	-1.1727	0.0076
465	Q6P069	Sorcin	*Sri*	-1.1714	0.0076
466	Q9CQW1	Synaptobrevin homolog YKT6	*Ykt6*	-1.1704	0.0077
467	Q3TUH1	Phosphatidate cytidylyltransferase mitochondrial	*Tamm41*	-1.1660	0.0078
468	Q9QXX4	Calcium-binding mitochondrial carrier protein Aralar2	*Slc25a13*	-1.1655	0.0078
469	Q11011	Puromycin-sensitive aminopeptidase	*Npepps*	-1.1644	0.0078
470	Q8K0Z7	Translational activator of cytochrome c oxidase 1	*Taco1*	-1.1631	0.0078
471	P13707	Glycerol-3-phosphate dehydrogenase [NAD(+)] cytoplasmic	*Gpd1*	-1.1608	0.0079
472	Q9WVK4	EH domain-containing protein 1	*Ehd1*	-1.1582	0.0080

473	Q3UHB8	Coiled-coil domain-containing protein 177	*Ccdc177*	-1.1581	0.0080
474	Q9WTS4	Teneurin-1	*Tenm1*	-1.1553	0.0081
475	Q3UUI3	Acyl-coenzyme A thioesterase THEM4	*Them4*	-1.1551	0.0081
476	Q06138	Calcium-binding protein 39	*Cab39*	-1.1547	0.0082
477	P50396	Rab GDP dissociation inhibitor alpha	*Gdi1*	-1.1545	0.0082
478	Q9CS42	Ribose-phosphate pyrophosphokinase 2	*Prps2*	-1.1476	0.0083
479	Q6PEE3	Ribonucleoside-diphosphate reductase subunit M2 B	*Rrm2b*	-1.1453	0.0083
480	Q91WP6	Serine protease inhibitor A3N	*Serpina3n*	-1.1406	0.0084
481	Q6PHN9	Ras-related protein Rab-35	*Rab35*	-1.1353	0.0085
482	P42125	Enoyl-CoA delta isomerase 1 mitochondrial	*Eci1*	-1.1344	0.0086
483	Q91ZA3	Propionyl-CoA carboxylase alpha chain mitochondrial	*Pcca*	-1.1333	0.0086
484	Q99PT1	Rho GDP-dissociation inhibitor 1	*Arhgdia*	-1.1333	0.0086
485	Q9WUE3	Transmembrane reductase CYB561D2	*Cyb561d2*	-1.1332	0.0086
486	P61022	Calcineurin B homologous protein 1	*Chp1*	-1.1330	0.0086
487	Q8BZM1	Glomulin	*Glmn*	-1.1330	0.0086
488	O35450	FK506-binding protein-like	*Fkbpl*	-1.1330	0.0086
489	Q3UHF7	Transcription factor HIVEP2	*Hivep2*	-1.1330	0.0086
490	Q9CRC9	Glucosamine-6-phosphate isomerase 2	*Gnpda2*	-1.1329	0.0086
491	Q9D023	Mitochondrial pyruvate carrier 2	*Mpc2*	-1.1318	0.0086
492	Q4KUS2	Protein unc-13 homolog A	*Unc13a*	-1.1300	0.0089
493	O70443	Guanine nucleotide-binding protein G(z) subunit alpha	*Gnaz*	-1.1300	0.0089
494	Q60631	Growth factor receptor-bound protein 2	*Grb2*	-1.1276	0.0089
495	P01635	Ig kappa chain V-V region K2 (Fragment)		-1.1253	0.0090
496	Q9CWU6	Ubiquinol-cytochrome-c reductase complex assembly factor 1	*Uqcc1*	-1.1253	0.0090
497	Q8BJY1	26S proteasome non-ATPase regulatory subunit 5	*Psmd5*	-1.1227	0.0090
498	Q3UVC0	Kinase suppressor of Ras 2	*Ksr2*	-1.1222	0.0090
499	Q99JY4	TraB domain-containing protein	*Trabd*	-1.1222	0.0090
500	Q8BKR5	Protein phosphatase 1 regulatory subunit 37	*Ppp1r37*	-1.1222	0.0090
501	Q9CQF3	Cleavage and polyadenylation specificity factor subunit 5	*Nudt21*	-1.1216	0.0091
502	Q9CQ86	Migration and invasion enhancer 1	*Mien1*	-1.1194	0.0091
503	Q5SQM0	Echinoderm microtubule-associated protein-like 6	*Eml6*	-1.1194	0.0091
504	Q9R013	Cathepsin F	*Ctsf*	-1.1194	0.0091
505	Q8BP47	Asparagine--tRNA ligase cytoplasmic	*NARS1*	-1.1191	0.0091
506	P62962	Profilin-1	*Pfn1*	-1.1173	0.0092
507	Q9CQZ6	NADH dehydrogenase [ubiquinone] 1 beta subcomplex subunit 3	*Ndufb3*	-1.1151	0.0093
508	O88384	Vesicle transport through interaction with t-SNAREs homolog 1B	*Vti1b*	-1.1137	0.0093
509	Q9CPP6	NADH dehydrogenase [ubiquinone] 1 alpha subcomplex subunit 5	*Ndufa5*	-1.1137	0.0093
510	Q8K021	Secretory carrier-associated membrane protein 1	*Scamp1*	-1.1134	0.0093
511	P05201	Aspartate aminotransferase cytoplasmic	*Got1*	-1.1113	0.0093
512	Q9QZD9	Eukaryotic translation initiation factor 3 subunit I	*Eif3i*	-1.1112	0.0093
513	P47199	Quinone oxidoreductase	*Cryz*	-1.1087	0.0094
514	Q9JK23	Proteasome assembly chaperone 1	*Psmg1*	-1.1087	0.0094
515	Q9JKX6	ADP-sugar pyrophosphatase	*Nudt5*	-1.1087	0.0094
516	Q9QZ06	Toll-interacting protein	*Tollip*	-1.1087	0.0094
517	Q9Z2R6	Protein unc-119 homolog A	*Unc119*	-1.1085	0.0094
518	O35988	Syndecan-4	*Sdc4*	-1.1084	0.0094
519	Q3TKT4	Transcription activator BRG1	*Smarca4*	-1.1084	0.0094
520	Q91XF0	Pyridoxine-5'-phosphate oxidase	*Pnpo*	-1.1084	0.0094
521	Q9D7G0	Ribose-phosphate pyrophosphokinase 1	*Prps1*	-1.1084	0.0094
522	Q8BFY6	Peflin	*Pef1*	-1.1082	0.0094
523	Q9R0N7	Synaptotagmin-7	*Syt7*	-1.1069	0.0094
524	Q80UG2	Plexin-A4	*Plxna4*	-1.1048	0.0097
525	Q9DC69	NADH dehydrogenase [ubiquinone] 1 alpha subcomplex subunit 9 mitochondrial	*Ndufa9*	-1.1035	0.0098
526	Q925I1	ATPase family AAA domain-containing protein 3	*Atad3*	-1.1014	0.0098
527	P62821	Ras-related protein Rab-1A	*Rab1A*	-1.1004	0.0099
528	P12960	Contactin-1	*Cntn1*	-1.0993	0.0099
529	Q8BHW2	Protein CP1	*Oscp1*	-1.0987	0.0099
530	O35381	Acidic leucine-rich nuclear phosphoprotein 32 family member A	*Anp32a*	-1.0987	0.0099
531	Q60900	ELAV-like protein 3	*Elavl3*	-1.0976	0.0100
532	Q922Q1	Mitochondrial amidoxime reducing component 2	*Mtarc2*	-1.0976	0.0100
533	Q6PER3	Microtubule-associated protein RP/EB family member 3	*Mapre3*	-1.0972	0.0100
534	Q78IK4	MIC complex subunit Mic27	*Apool*	-1.0968	0.0100
535	Q9CPR5	39S ribosomal protein L15 mitochondrial	*Mrpl15*	-1.0929	0.0101
536	Q3TES0	IQ motif and SEC7 domain-containing protein 3	*Iqsec3*	-1.0920	0.0101
537	P63024	Vesicle-associated membrane protein 3	*Vamp3*	-1.0909	0.0102
538	Q9Z0R6	Intersectin-2	*Itsn2*	-1.0905	0.0102
539	P35550	rRNA 2'-O-methyltransferase fibrillarin	*Fbl*	-1.0849	0.0103
540	P68510	14-3-3 protein eta	*Ywhah*	-1.0846	0.0103
541	Q8QZS1	3-hydroxyisobutyryl-CoA hydrolase mitochondrial	*Hibch*	-1.0813	0.0105
542	P97820	Mitogen-activated protein kinase kinase kinase kinase 4	*Map4k4*	-1.0803	0.0105
543	Q8BP40	Lysophosphatidic acid phosphatase type 6	*Acp6*	-1.0798	0.0105
544	Q9CQE8	RNA transcription translation and transport factor protein	*RTRAF*	-1.0798	0.0105
545	Q9R0Q9	Mannose-P-dolichol utilization defect 1 protein	*Mpdu1*	-1.0787	0.0105
546	P62317	Small nuclear ribonucleoprotein Sm D2	*Snrpd2*	-1.0779	0.0106
547	Q8C0L0	Thioredoxin-related transmembrane protein 4	*Tmx4*	-1.0758	0.0106
548	Q91X72	Hemopexin	*Hpx*	-1.0726	0.0107
549	Q8BR92	Paralemmin-2	*Palm2*	-1.0687	0.0121
550	Q8BZA9	Fructose-2 6-bisphosphatase TIGAR	*Tigar*	-1.0665	0.0122
551	Q9R0L7	A-kinase anchor protein 8-like	*Akap8l*	-1.0665	0.0122

552	P14847	C-reactive protein	*Crp*	-1.0665	0.0122
553	Q9D0P8	Intraflagellar transport protein 27 homolog	*Ift27*	-1.0665	0.0122
554	Q61016	Guanine nucleotide-binding protein G(I)/G(S)/G(O) subunit gamma-7	*Gng7*	-1.0665	0.0122
555	P56812	Programmed cell death protein 5	*Pdcd5*	-1.0665	0.0122
556	P01639	Ig kappa chain V-V region MOPC 41	*Gm5571*	-1.0665	0.0122
557	P35831	Tyrosine-protein phosphatase non-receptor type 12	*Ptpn12*	-1.0665	0.0122
558	P01786	Ig heavy chain V region MOPC 47A		-1.0665	0.0122
559	Q80XC2	tRNA (adenine(58)-N(1))-methyltransferase catalytic subunit TRMT61A	*Trmt61a*	-1.0665	0.0122
560	Q91ZW2	GDP-fucose protein O-fucosyltransferase 1	*Pofut1*	-1.0665	0.0122
561	Q8BQZ5	Cleavage and polyadenylation specificity factor subunit 4	*Cpsf4*	-1.0665	0.0122
562	Q9CZJ0	Metallophosphoesterase MPPED2	*Mpped2*	-1.0665	0.0122
563	Q91ZG2	Metallophosphoesterase domain-containing protein 1	*Mpped1*	-1.0665	0.0122
564	Q9R0G7	Zinc finger E-box-binding homeobox 2	*Zeb2*	-1.0665	0.0122
565	Q7TQA1	Immunoglobulin superfamily member 1	*Igsf1*	-1.0665	0.0122
566	Q62087	Serum paraoxonase/lactonase 3	*Pon3*	-1.0665	0.0122
567	P01632	Ig kappa chain V-I region S107A	*Igkv7-33*	-1.0665	0.0122
568	P01657	Ig kappa chain V-III region PC 2413		-1.0665	0.0122
569	P01634	Ig kappa chain V-V region MOPC 21		-1.0665	0.0122
570	P61014	Cardiac phospholamban	*Pln*	-1.0665	0.0122
571	Q8VEG6	CCR4-NOT transcription complex subunit 6-like	*Cnot6l*	-1.0665	0.0122
572	P06328	Ig heavy chain V region 1-72	*Ighv1-72*	-1.0665	0.0122
573	P01754	Ig heavy chain V region 1-62-3	*Ighv1-62-3*	-1.0665	0.0122
574	P01753	Ig heavy chain V region 186-1	*4 SV*	-1.0665	0.0122
575	Q76KF0	Semaphorin-6D	*Sema6d*	-1.0665	0.0122
576	Q8VBY2	Calcium/calmodulin-dependent protein kinase kinase 1	*Camkk1*	-1.0653	0.0122
577	P0C8K7	Small integral membrane protein 1	*Smim1*	-1.0651	0.0122
578	Q9R118	Serine protease HTRA1	*Htra1*	-1.0646	0.0122
579	O08709	Peroxiredoxin-6	*Prdx6*	-1.0642	0.0123
580	Q62465	Synaptic vesicle membrane protein VAT-1 homolog	*Vat1*	-1.0609	0.0124
581	Q9DB25	Dolichyl-phosphate beta-glucosyltransferase	*Alg5*	-1.0606	0.0124
582	Q8R4F1	Netrin-G2	*Ntng2*	-1.0603	0.0125
583	Q922J3	CAP-Gly domain-containing linker protein 1	*Clip1*	-1.0601	0.0125
584	P61264	Syntaxin-1B	*Stx1b*	-1.0579	0.0126
585	Q3UHD9	Arf-GAP with GTPase ANK repeat and PH domain-containing protein 2	*Agap2*	-1.0536	0.0127
586	P27048	Small nuclear ribonucleoprotein-associated protein B	*Snrpb*	-1.0533	0.0127
587	Q9R1V6	Disintegrin and metalloproteinase domain-containing protein 22	*Adam22*	-1.0523	0.0128
588	O55234	Proteasome subunit beta type-5	*Psmb5*	-1.0493	0.0130
589	P0DP60	Ly-6/neurotoxin-like protein 1	*Lynx1*	-1.0488	0.0131
590	P56564	Excitatory amino acid transporter 1	*Slc1a3*	-1.0480	0.0131
591	P00493	Hypoxanthine-guanine phosphoribosyltransferase	*Hprt1*	-1.0439	0.0132
592	Q91YJ3	Thymocyte nuclear protein 1	*Thyn1*	-1.0431	0.0132
593	Q9DBG5	Perilipin-3	*Plin3*	-1.0428	0.0132
594	O08992	Syntenin-1	*Sdcbp*	-1.0428	0.0132
595	Q9CXZ1	NADH dehydrogenase [ubiquinone] iron-sulfur protein 4 mitochondrial	*Ndufs4*	-1.0428	0.0132
596	Q9CPU0	Lactoylglutathione lyase	*Glo1*	-1.0414	0.0133
597	Q8BTH8	Casein kinase I isoform gamma-1	*Csnk1g1*	-1.0397	0.0133
598	Q8CH25	SAFB-like transcription modulator	*Sltm*	-1.0393	0.0133
599	P02089	Hemoglobin subunit beta-2	*Hbb-b2*	-1.0380	0.0135
600	O35409	Glutamate carboxypeptidase 2	*Folh1*	-1.0377	0.0135
601	P61294	Ras-related protein Rab-6B	*Rab6b*	-1.0376	0.0135
602	P62715	Serine/threonine-protein phosphatase 2A catalytic subunit beta isoform	*Ppp2cb*	-1.0362	0.0135
603	Q8C2E7	WASH complex subunit 5	*Washc5*	-1.0342	0.0135
604	P10639	Thioredoxin	*Txn*	-1.0335	0.0136
605	Q8VDD8	WASH complex subunit 1	*Washc1*	-1.0333	0.0136
606	Q9WVQ5	Methylthioribulose-1-phosphate dehydratase	*Apip*	-1.0330	0.0136
607	Q9CQV7	Mitochondrial import inner membrane translocase subunit TIM14	*Dnajc19*	-1.0330	0.0136
608	Q8VCC8	Rap guanine nucleotide exchange factor 3	*Rapgef3*	-1.0330	0.0136
609	P46660	Alpha-internexin	*Ina*	-1.0325	0.0136
610	Q9QZ88	Vacuolar protein sorting-associated protein 29	*Vps29*	-1.0319	0.0136
611	P62484	Abl interactor 2	*Abi2*	-1.0314	0.0137
612	Q8BHC1	Ras-related protein Rab-39B	*Rab39b*	-1.0300	0.0137
613	P63216	Guanine nucleotide-binding protein G(I)/G(S)/G(O) subunit gamma-3	*Gng3*	-1.0286	0.0137
614	Q61102	Iron-sulfur clusters transporter ABCB7 mitochondrial	*Abcb7*	-1.0283	0.0138
615	P28660	Nck-associated protein 1	*Nckap1*	-1.0278	0.0138
616	Q9CXJ1	Probable glutamate--tRNA ligase mitochondrial	*Ears2*	-1.0245	0.0139
617	Q8K097	Protein lifeguard 2	*Faim2*	-1.0245	0.0139
618	B0F2B4	Neuroligin 4-like	*Nlgn4l*	-1.0245	0.0139
619	Q62422	Osteoclast-stimulating factor 1	*Ostf1*	-1.0245	0.0139
620	P01647	Ig kappa chain V-V region HP 124E1		-1.0245	0.0139
621	P01645	Ig kappa chain V-V region HP 93G7		-1.0245	0.0139
622	P01644	Ig kappa chain V-V region HP R16.7		-1.0245	0.0139
623	P01648	Ig kappa chain V-V region HP 91A3		-1.0245	0.0139
624	Q64433	10 kDa heat shock protein mitochondrial	*Hspe1*	-1.0239	0.0139
625	P19253	60S ribosomal protein L13a	*Rpl13a*	-1.0226	0.0139
626	P35279	Ras-related protein Rab-6A	*Rab6a*	-1.0225	0.0139
627	Q78IK2	ATP synthase membrane subunit K mitochondrial	*Atp5mk*	-1.0218	0.0140
628	Q9Z2V6	Histone deacetylase 5	*Hdac5*	-1.0213	0.0140
629	Q9CZH3	Proteasome assembly chaperone 3	*Psmg3*	-1.0213	0.0140
630	Q9Z2A0	3-phosphoinositide-dependent protein kinase 1	*Pdpk1*	-1.0208	0.0140

631	Q62059	Versican core protein	*Vcan*	-1.0185	0.0142
632	Q9QYY8	Spastin	*Spast*	-1.0181	0.0142
633	Q9DB50	AP-1 complex subunit sigma-2	*Ap1s2*	-1.0181	0.0142
634	Q9CXT8	Mitochondrial-processing peptidase subunit beta	*Pmpcb*	-1.0173	0.0142
635	Q9JKB1	Ubiquitin carboxyl-terminal hydrolase isozyme L3	*Uchl3*	-1.0144	0.0143
636	P62631	Elongation factor 1-alpha 2	*Eef1a2*	-1.0099	0.0145
637	B1AXV0	DOMON domain-containing protein FRRS1L	*Frrs1l*	-1.0077	0.0146
638	P47911	60S ribosomal protein L6	*Rpl6*	-1.0071	0.0146
639	Q91ZR1	Ras-related protein Rab-4B	*Rab4b*	-1.0050	0.0147
640	P62077	Mitochondrial import inner membrane translocase subunit Tim8 B	*Timm8b*	-1.0044	0.0148
641	A2AGL3	Ryanodine receptor 3	*Ryr3*	-1.0023	0.0149
642	Q9Z280	Phospholipase D1	*Pld1*	-1.0014	0.0149
643	P53026	60S ribosomal protein L10a	*Rpl10a*	-1.0006	0.0150
644	Q3UMF0	Cordon-bleu protein-like 1	*Cobll1*	-0.9995	0.0150
645	Q8R238	Serine dehydratase-like	*Sdsl*	-0.9995	0.0150
646	Q9D0B0	Serine/arginine-rich splicing factor 9	*Srsf9*	-0.9995	0.0150
647	Q8BK75	Elongator complex protein 6	*Elp6*	-0.9995	0.0150
648	Q9WVA2	Mitochondrial import inner membrane translocase subunit Tim8 A	*Timm8a1*	-0.9995	0.0150
649	Q9JME7	Trafficking protein particle complex subunit 2-like protein	*Trappc2l*	-0.9995	0.0150
650	Q60932	Voltage-dependent anion-selective channel protein 1	*Vdac1*	-0.9974	0.0150
651	A2AJA9	Apical junction component 1 homolog	*Ajm1*	-0.9966	0.0151
652	P57784	U2 small nuclear ribonucleoprotein A'	*Snrpa1*	-0.9955	0.0151
653	P56399	Ubiquitin carboxyl-terminal hydrolase 5	*Usp5*	-0.9949	0.0151
654	Q8K4J6	Myocardin-related transcription factor A	*Mrtfa*	-0.9940	0.0152
655	Q8CCJ4	APC membrane recruitment protein 2	*Amer2*	-0.9940	0.0152
656	Q3TX08	tRNA (guanine(26)-N(2))-dimethyltransferase	*Trmt1*	-0.9940	0.0152
657	B9EJI9	Transmembrane protein 229A	*Tmem229a*	-0.9940	0.0152
658	Q91WI7	KICSTOR complex protein ITFG2	*Itfg2*	-0.9940	0.0152
659	Q91WN1	DnaJ homolog subfamily C member 9	*Dnajc9*	-0.9940	0.0152
660	Q9D2X5	MAU2 chromatid cohesion factor homolog	*Mau2*	-0.9940	0.0152
661	Q9CQH7	Transcription factor BTF3 homolog 4	*Btf3l4*	-0.9940	0.0152
662	P58064	28S ribosomal protein S6 mitochondrial	*Mrps6*	-0.9940	0.0152
663	Q6P5H6	FERM domain-containing protein 5	*Frmd5*	-0.9940	0.0152
664	Q9QYB8	Beta-adducin	*Add2*	-0.9920	0.0153
665	P35486	Pyruvate dehydrogenase E1 component subunit alpha somatic form mitochondrial	*Pdha1*	-0.9917	0.0153
666	P84078	ADP-ribosylation factor 1	*Arf1*	-0.9902	0.0156
667	Q58A65	C-Jun-amino-terminal kinase-interacting protein 4	*Spag9*	-0.9869	0.0171
668	Q9Z2W8	Glutamate receptor 4	*Gria4*	-0.9862	0.0172
669	Q01405	Protein transport protein Sec23A	*Sec23a*	-0.9855	0.0172
670	Q5EBJ4	Ermin	*Ermn*	-0.9854	0.0172
671	Q9R1P1	Proteasome subunit beta type-3	*Psmb3*	-0.9854	0.0172
672	Q9CZT8	Ras-related protein Rab-3B	*Rab3b*	-0.9850	0.0172
673	Q3UFF7	Lysophospholipase-like protein 1	*Lyplal1*	-0.9836	0.0175
674	P11031	Activated RNA polymerase II transcriptional coactivator p15	*Sub1*	-0.9832	0.0175
675	Q62393	Tumor protein D52	*Tpd52*	-0.9818	0.0175
676	A2AN08	E3 ubiquitin-protein ligase UBR4	*Ubr4*	-0.9805	0.0176
677	Q91YL3	Uridine-cytidine kinase-like 1	*Uckl1*	-0.9803	0.0176
678	Q791T5	Mitochondrial carrier homolog 1	*Mtch1*	-0.9764	0.0180
679	Q8JZW4	Copine-5	*Cpne5*	-0.9755	0.0180
680	P98084	Amyloid-beta A4 precursor protein-binding family A member 2	*Apba2*	-0.9753	0.0181
681	Q9CYW4	Haloacid dehalogenase-like hydrolase domain-containing protein 3	*Hdhd3*	-0.9753	0.0181
682	P61804	Dolichyl-diphosphooligosaccharide--protein glycosyltransferase subunit DAD1	*Dad1*	-0.9753	0.0181
683	P13020	Gelsolin	*Gsn*	-0.9733	0.0182
684	P97855	Ras GTPase-activating protein-binding protein 1	*G3bp1*	-0.9708	0.0183
685	Q9D7A8	Armadillo repeat-containing protein 1	*Armc1*	-0.9708	0.0183
686	Q6ZPE2	Myotubularin-related protein 5	*Sbf1*	-0.9697	0.0184
687	O88844	Isocitrate dehydrogenase [NADP] cytoplasmic	*Idh1*	-0.9688	0.0184
688	Q99KC8	von Willebrand factor A domain-containing protein 5A	*Vwa5a*	-0.9665	0.0185
689	P61205	ADP-ribosylation factor 3	*Arf3*	-0.9657	0.0186
690	Q3UVL4	Vacuolar protein sorting-associated protein 51 homolog	*Vps51*	-0.9644	0.0186
691	Q61097	Kinase suppressor of Ras 1	*Ksr1*	-0.9618	0.0188
692	Q3UHX2	28 kDa heat- and acid-stable phosphoprotein	*Pdap1*	-0.9618	0.0188
693	P97346	Nucleoredoxin	*Nxn*	-0.9611	0.0188
694	E9PZJ8	Activating signal cointegrator 1 complex subunit 3	*Ascc3*	-0.9611	0.0188
695	Q99LC8	Translation initiation factor eIF-2B subunit alpha	*Eif2b1*	-0.9606	0.0188
696	Q8BI72	CDKN2A-interacting protein	*Cdkn2aip*	-0.9598	0.0189
697	Q9DCS2	Methyltransferase-like 26	*Mettl26*	-0.9598	0.0189
698	Q8C460	ERI1 exoribonuclease 3	*Eri3*	-0.9598	0.0189
699	Q8BQM8	Echinoderm microtubule-associated protein-like 5	*Eml5*	-0.9598	0.0189
700	Q3THW5	Histone H2A.V	*H2az2*	-0.9596	0.0189
701	P0C0S6	Histone H2A.Z	*H2az1*	-0.9596	0.0189
702	O88958	Glucosamine-6-phosphate isomerase 1	*Gnpda1*	-0.9591	0.0189
703	P29391	Ferritin light chain 1	*Ftl1*	-0.9589	0.0189
704	Q8CHG7	Rap guanine nucleotide exchange factor 2	*Rapgef2*	-0.9579	0.0191
705	Q99KP3	Lambda-crystallin homolog	*Cryl1*	-0.9555	0.0194
706	Q8VE47	Ubiquitin-like modifier-activating enzyme 5	*Uba5*	-0.9555	0.0194
707	O08579	Emerin	*Emd*	-0.9555	0.0194
708	Q9D1E6	Tubulin-folding cofactor B	*Tbcb*	-0.9476	0.0200
709	Q810U3	Neurofascin	*Nfasc*	-0.9465	0.0201

710	P39054	Dynamin-2	*Dnm2*	-0.9464	0.0201
711	Q7TQF7	Amphiphysin	*Amph*	-0.9460	0.0203
712	P62878	E3 ubiquitin-protein ligase RBX1	*Rbx1*	-0.9427	0.0204
713	P69566	Ran-binding protein 9	*Ranbp9*	-0.9395	0.0205
714	Q80XI4	Phosphatidylinositol 5-phosphate 4-kinase type-2 beta	*Pip4k2b*	-0.9392	0.0205
715	O08638	Myosin-11	*Myh11*	-0.9392	0.0205
716	Q61595	Kinectin	*Ktn1*	-0.9387	0.0206
717	Q99KI3	ER membrane protein complex subunit 3	*Emc3*	-0.9387	0.0206
718	Q8R2H9	Phosphoethanolamine/phosphocholine phosphatase	*Phospho1*	-0.9387	0.0206
719	P02088	Hemoglobin subunit beta-1	*Hbb-b1*	-0.9374	0.0207
720	Q8VEJ9	Vacuolar protein sorting-associated protein 4A	*Vps4a*	-0.9367	0.0208
721	O70252	Heme oxygenase 2	*Hmox2*	-0.9364	0.0208
722	Q920P5	Adenylate kinase isoenzyme 5	*Ak5*	-0.9363	0.0208
723	P35288	Ras-related protein Rab-23	*Rab23*	-0.9347	0.0210
724	P62996	Transformer-2 protein homolog beta	*Tra2b*	-0.9341	0.0210
725	Q99JY8	Phospholipid phosphatase 3	*Plpp3*	-0.9334	0.0210
726	O70492	Sorting nexin-3	*Snx3*	-0.9321	0.0211
727	Q924M7	Mannose-6-phosphate isomerase	*Mpi*	-0.9315	0.0211
728	P63163	Small nuclear ribonucleoprotein-associated protein N	*Snrpn*	-0.9315	0.0211
729	P55066	Neurocan core protein	*Ncan*	-0.9303	0.0212
730	P08226	Apolipoprotein E	*Apoe*	-0.9303	0.0212
731	P22682	E3 ubiquitin-protein ligase CBL	*Cbl*	-0.9293	0.0213
732	Q9D855	Cytochrome b-c1 complex subunit 7	*Uqcrb*	-0.9279	0.0214
733	Q6PGN3	Serine/threonine-protein kinase DCLK2	*Dclk2*	-0.9277	0.0214
734	Q8BP48	Methionine aminopeptidase 1	*Metap1*	-0.9240	0.0216
735	P21550	Beta-enolase	*Eno3*	-0.9188	0.0220
736	Q8BH04	Phosphoenolpyruvate carboxykinase [GTP] mitochondrial	*Pck2*	-0.9180	0.0220
737	P60487	Chronophin	*Pdxp*	-0.9173	0.0221
738	P01636	Ig kappa chain V-V region MOPC 149		-0.9170	0.0221
739	O54692	Centromere/kinetochore protein zw10 homolog	*Zw10*	-0.9170	0.0221
740	Q8BVI5	Syntaxin-16	*Stx16*	-0.9170	0.0221
741	Q8BG94	COMM domain-containing protein 7	*Commd7*	-0.9170	0.0221
742	Q9ER35	Fructosamine-3-kinase	*Fn3k*	-0.9165	0.0221
743	Q99LX0	Parkinson disease protein 7 homolog	*Park7*	-0.9140	0.0223
744	P60521	Gamma-aminobutyric acid receptor-associated protein-like 2	*Gabarapl2*	-0.9108	0.0225
745	P50518	V-type proton ATPase subunit E 1	*Atp6v1e1*	-0.9105	0.0225
746	P61290	Proteasome activator complex subunit 3	*Psme3*	-0.9095	0.0225
747	Q9WTL7	Acyl-protein thioesterase 2	*Lypla2*	-0.9095	0.0225
748	P03899	NADH-ubiquinone oxidoreductase chain 3	*Mtnd3*	-0.9087	0.0226
749	Q8BYK4	Retinol dehydrogenase 12	*Rdh12*	-0.9075	0.0229
750	Q61792	LIM and SH3 domain protein 1	*Lasp1*	-0.9030	0.0233
751	Q8K2X3	CST complex subunit STN1	*Stn1*	-0.9026	0.0233
752	Q9DBR1	5'-3' exoribonuclease 2	*Xrn2*	-0.9001	0.0236
753	Q8BLN5	Lanosterol synthase	*Lss*	-0.8997	0.0236
754	O88738	Baculoviral IAP repeat-containing protein 6	*Birc6*	-0.8991	0.0237
755	Q8CFV4	Neuritin	*Nrn1*	-0.8985	0.0237
756	Q80UU9	Membrane-associated progesterone receptor component 2	*Pgrmc2*	-0.8971	0.0241
757	Q8BIZ1	Ankyrin repeat and sterile alpha motif domain-containing protein 1B	*Anks1b*	-0.8966	0.0241
758	Q61147	Ceruloplasmin	*Cp*	-0.8959	0.0242
759	P67984	60S ribosomal protein L22	*Rpl22*	-0.8921	0.0254
760	Q8BGB7	Enolase-phosphatase E1	*Enoph1*	-0.8909	0.0257
761	P35505	Fumarylacetoacetase	*Fah*	-0.8908	0.0257
762	Q8VEM8	Phosphate carrier protein mitochondrial	*Slc25a3*	-0.8895	0.0258
763	Q3UH99	Protein shisa-6	*Shisa6*	-0.8894	0.0258
764	O70589	Peripheral plasma membrane protein CASK	*Cask*	-0.8889	0.0259
765	Q9CQA1	Trafficking protein particle complex subunit 5	*Trappc5*	-0.8880	0.0259
766	Q8BFR4	N-acetylglucosamine-6-sulfatase	*Gns*	-0.8880	0.0259
767	Q9D1N9	39S ribosomal protein L21 mitochondrial	*Mrpl21*	-0.8880	0.0259
768	P63011	Ras-related protein Rab-3A	*Rab3a*	-0.8879	0.0259
769	P35283	Ras-related protein Rab-12	*Rab12*	-0.8865	0.0265
770	P70349	Histidine triad nucleotide-binding protein 1	*Hint1*	-0.8860	0.0265
771	Q8BFZ9	Erlin-2	*Erlin2*	-0.8848	0.0266
772	O88322	Nidogen-2	*Nid2*	-0.8848	0.0266
773	P97412	Lysosomal-trafficking regulator	*Lyst*	-0.8843	0.0266
774	Q9CYR0	Single-stranded DNA-binding protein mitochondrial	*Ssbp1*	-0.8825	0.0267
775	O09117	Synaptophysin-like protein 1	*Sypl1*	-0.8788	0.0269
776	P45376	Aldo-keto reductase family 1 member B1	*Akr1b1*	-0.8781	0.0270
777	O08583	THO complex subunit 4	*Alyref*	-0.8778	0.0270
778	Q6NVE9	Protein phosphatase PTC7 homolog	*Pptc7*	-0.8743	0.0271
779	Q810B7	SLIT and NTRK-like protein 5	*Slitrk5*	-0.8725	0.0272
780	P19001	Keratin type I cytoskeletal 19	*Krt19*	-0.8724	0.0273
781	P54797	Transport and Golgi organization 2 homolog	*Tango2*	-0.8709	0.0274
782	Q8K1R7	Serine/threonine-protein kinase Nek9	*Nek9*	-0.8706	0.0274
783	P49722	Proteasome subunit alpha type-2	*Psma2*	-0.8706	0.0274
784	P97433	Rho guanine nucleotide exchange factor 28	*Arhgef28*	-0.8706	0.0274
785	P08071	Lactotransferrin	*Ltf*	-0.8706	0.0274
786	Q9JI75	Ribosyldihydronicotinamide dehydrogenase [quinone]	*Nqo2*	-0.8706	0.0274
787	P97470	Serine/threonine-protein phosphatase 4 catalytic subunit	*Ppp4c*	-0.8705	0.0274
788	Q922Q8	Leucine-rich repeat-containing protein 59	*Lrrc59*	-0.8705	0.0274

789	Q922J6	Tetraspanin-2	*Tspan2*	-0.8705	0.0274
790	Q9QY42	Prosaposin receptor GPR37	*Gpr37*	-0.8700	0.0274
791	Q9DCU6	39S ribosomal protein L4 mitochondrial	*Mrpl4*	-0.8700	0.0274
792	P46467	Vacuolar protein sorting-associated protein 4B	*Vps4b*	-0.8669	0.0276
793	Q9CRB9	MIC complex subunit Mic19	*Chchd3*	-0.8666	0.0276
794	Q91YP2	Neurolysin mitochondrial	*Nln*	-0.8659	0.0277
795	Q9CQJ8	NADH dehydrogenase [ubiquinone] 1 beta subcomplex subunit 9	*Ndufb9*	-0.8659	0.0277
796	Q8K4I3	Rho guanine nucleotide exchange factor 6	*Arhgef6*	-0.8640	0.0281
797	Q60610	Rho guanine nucleotide exchange factor TIAM1	*Tiam1*	-0.8640	0.0281
798	P21460	Cystatin-C	*Cst3*	-0.8635	0.0281
799	Q9JM63	ATP-sensitive inward rectifier potassium channel 10	*Kcnj10*	-0.8628	0.0281
800	Q9JMG7	Hepatoma-derived growth factor-related protein 3	*Hdgfl3*	-0.8623	0.0281
801	Q99JI6	Ras-related protein Rap-1b	*Rap1b*	-0.8620	0.0282
802	Q8VHL0	Urea transporter 1	*Slc14a1*	-0.8610	0.0282
803	Q3THE2	Myosin regulatory light chain 12B	*Myl12b*	-0.8607	0.0282
804	Q6PDY2	2-aminoethanethiol dioxygenase	*Ado*	-0.8571	0.0285
805	Q6GYP7	Ral GTPase-activating protein subunit alpha-1	*Ralgapa1*	-0.8568	0.0287
806	Q8C522	Endonuclease domain-containing 1 protein	*Endod1*	-0.8562	0.0287
807	Q7TQD2	Tubulin polymerization-promoting protein	*Tppp*	-0.8558	0.0287
808	P23591	GDP-L-fucose synthase	*Gfus*	-0.8527	0.0290
809	P00920	Carbonic anhydrase 2	*Ca2*	-0.8527	0.0290
810	O70493	Sorting nexin-12	*Snx12*	-0.8527	0.0290
811	Q62348	Translin	*Tsn*	-0.8520	0.0292
812	Q925T6	Glutamate receptor-interacting protein 1	*Grip1*	-0.8520	0.0292
813	Q8BK08	Transmembrane protein 11 mitochondrial	*Tmem11*	-0.8520	0.0292
814	Q8BMF3	NADP-dependent malic enzyme mitochondrial	*Me3*	-0.8505	0.0293
815	Q91V24	ATP-binding cassette sub-family A member 7	*Abca7*	-0.8495	0.0295
816	P17710	Hexokinase-1	*Hk1*	-0.8486	0.0296
817	Q9WVA4	Transgelin-2	*Tagln2*	-0.8470	0.0298
818	Q91YH5	Atlastin-3	*Atl3*	-0.8447	0.0299
819	Q61772	Ephrin type-A receptor 7	*Epha7*	-0.8445	0.0299
820	Q8BH59	Calcium-binding mitochondrial carrier protein Aralar1	*Slc25a12*	-0.8441	0.0299
821	Q8BH74	Nuclear pore complex protein Nup107	*Nup107*	-0.8435	0.0300
822	Q8C0Z1	Protein FAM234A	*Fam234a*	-0.8435	0.0300
823	Q8BGR2	Volume-regulated anion channel subunit LRRC8D	*Lrrc8d*	-0.8435	0.0300
824	P84099	60S ribosomal protein L19	*Rpl19*	-0.8435	0.0300
825	Q9CYH5	Glucose-fructose oxidoreductase domain-containing protein 2	*Gfod2*	-0.8435	0.0300
826	Q6P4S8	Integrator complex subunit 1	*Ints1*	-0.8435	0.0300
827	Q8BGS7	Choline/ethanolaminephosphotransferase 1	*Cept1*	-0.8435	0.0300
828	O55126	Protein NipSnap homolog 2	*Nipsnap2*	-0.8434	0.0300
829	O70433	Four and a half LIM domains protein 2	*Fhl2*	-0.8434	0.0300
830	Q6ZQ82	Rho GTPase-activating protein 26	*Arhgap26*	-0.8434	0.0300
831	Q8BFZ3	Beta-actin-like protein 2	*Actbl2*	-0.6308	0.0812
832	P62737	Actin aortic smooth muscle	*Acta2*	-0.5124	0.1249
