# Supplementary material for: Hippocampal dentate gyri proteomics reveals Wnt signaling involvement in the behavioral impairment in the THRSP-overexpressing ADHD mouse model
Source: Commun Biol. 2023 Jan 16;6:55. doi: 10.1038/s42003-022-04387-5 (PMC9842619; doi:10.1038/s42003-022-04387-5)
Supplement: Supplementary file 2 — Description of Additional Supplementary Files [file 42003_2022_4387_MOESM2_ESM.pdf]

Description of Additional Supplementary Files

File Name: Supplementary Video 1

Description: Sample mouse treadmill experiment
