# Supplementary material for: Hippocampal dentate gyri proteomics reveals Wnt signaling involvement in the behavioral impairment in the THRSP-overexpressing ADHD mouse model
Source: Commun Biol. 2023 Jan 16;6:55. doi: 10.1038/s42003-022-04387-5 (PMC9842619; doi:10.1038/s42003-022-04387-5)
Supplement: Supplementary file 4 — Reporting Summary [file 42003_2022_4387_MOESM4_ESM.pdf]

nature portfolio

Corresponding author(s): DBPR COMMSBIO-22-3082C

Last updated by author(s): Nov 12, 2022

Reporting Summary

Nature Portfolio wishes to improve the reproducibility of the work that we publish. This form provides structure for consistency and transparency in reporting. For further information on Nature Portfolio policies, see our Editorial Policies and the Editorial Policy Checklist.

Statistics

For all statistical analyses, confirm that the following items are present in the figure legend, table legend, main text, or Methods section.

n/a	Confirmed	
☐	☒	The exact sample size (*n*) for each experimental group/condition, given as a discrete number and unit of measurement
☐	☒	A statement on whether measurements were taken from distinct samples or whether the same sample was measured repeatedly
☐	☒	The statistical test(s) used AND whether they are one- or two-sided<br>*Only common tests should be described solely by name; describe more complex techniques in the Methods section.*
☐	☒	A description of all covariates tested
☐	☒	A description of any assumptions or corrections, such as tests of normality and adjustment for multiple comparisons
☐	☒	A full description of the statistical parameters including central tendency (e.g. means) or other basic estimates (e.g. regression coefficient) AND variation (e.g. standard deviation) or associated estimates of uncertainty (e.g. confidence intervals)
☐	☐	For null hypothesis testing, the test statistic (e.g. *F*, *t*, *r*) with confidence intervals, effect sizes, degrees of freedom and *P* value noted<br>*Give P values as exact values whenever suitable.*
☐	☒	For Bayesian analysis, information on the choice of priors and Markov chain Monte Carlo settings
☐	☒	For hierarchical and complex designs, identification of the appropriate level for tests and full reporting of outcomes
☐	☒	Estimates of effect sizes (e.g. Cohen's *d*, Pearson's *r*), indicating how they were calculated

Our web collection on statistics for biologists contains articles on many of the points above.

Software and code

Policy information about availability of computer code

Data collection	No software was used
Data analysis	GraphPad Prism version 9.4.1 (681)

For manuscripts utilizing custom algorithms or software that are central to the research but not yet described in published literature, software must be made available to editors and reviewers. We strongly encourage code deposition in a community repository (e.g. GitHub). See the Nature Portfolio guidelines for submitting code & software for further information.

Data

Policy information about availability of data

All manuscripts must include a data availability statement. This statement should provide the following information, where applicable:

- Accession codes, unique identifiers, or web links for publicly available datasets
- A description of any restrictions on data availability
- For clinical datasets or third party data, please ensure that the statement adheres to our policy

The authors declare that the data supporting the findings of this study are available within the paper and its supplementary information files.

Human research participants

Policy information about studies involving human research participants and Sex and Gender in Research.

Reporting on sex and gender	No human participants were included in this research.
Population characteristics	*Describe the covariate-relevant population characteristics of the human research participants (e.g. age, genotypic information, past and current diagnosis and treatment categories). If you filled out the behavioural & social sciences study design questions and have nothing to add here, write "See above."*
Recruitment	*Describe how participants were recruited. Outline any potential self-selection bias or other biases that may be present and how these are likely to impact results.*
Ethics oversight	*Identify the organization(s) that approved the study protocol.*

Note that full information on the approval of the study protocol must also be provided in the manuscript.

Field-specific reporting

Please select the one below that is the best fit for your research. If you are not sure, read the appropriate sections before making your selection.

☒ Life sciences ☐ Behavioural & social sciences ☐ Ecological, evolutionary & environmental sciences

For a reference copy of the document with all sections, see nature.com/documents/nr-reporting-summary-flat.pdf

Life sciences study design

All studies must disclose on these points even when the disclosure is negative.

Sample size	For graphical purposes, data are presented as mean±standard error of the mean, and all statistical analyses were conducted on raw data tested for normal (Gaussian) distribution using the D'Agostino-Pearson omnibus. The animal numbers and recorded data points are indicated in all figures. The results were analyzed using either one-way or two-way analysis of variance with or without repeated measures, followed by Tukey's multiple comparison test. A level of probability of $p \leq 0.05$ was defined as the threshold for statistical significance. Experiments were replicated at least three times.
Data exclusions	No data were excluded from the analysis.
Replication	Preliminary experiments were conducted and all attempts at replication were successful.
Randomization	Samples allocation were randomly selected.
Blinding	All investigators were blinded to group allocation during data collection and/or analysis.

Reporting for specific materials, systems and methods

We require information from authors about some types of materials, experimental systems and methods used in many studies. Here, indicate whether each material, system or method listed is relevant to your study. If you are not sure if a list item applies to your research, read the appropriate section before selecting a response.

Materials & experimental systems

n/a	Involved in the study	
☐	☒	Antibodies
☒	☐	Eukaryotic cell lines
☒	☐	Palaeontology and archaeology
☐	☒	Animals and other organisms
☒	☐	Clinical data
☒	☐	Dual use research of concern

Methods

n/a	Involved in the study	
☒	☐	ChIP-seq
☒	☐	Flow cytometry
☒	☐	MRI-based neuroimaging

Antibodies

Antibodies used	1. rabbit polyclonal anti-phosphorylated-GSK3B (1/1000)

Antibodies used	2. rabbit monoclonal anti-GSK3B (1/1000)<br>3. rabbit polyclonal anti-CSNK1E (1/1000)<br>4. rabbit polyclonal anti-GFAP (1/1000)<br>5. mouse monoclonal anti-NEU-N (1/1000)<br>6. mouse monoclonal anti-ACTB (1/5000)<br>7. HRP-conjugated anti-rabbit (1/3000)<br>8. HRP-conjugated anti-mouse (1/5000)
Validation	1. rabbit polyclonal anti-phosphorylated-GSK3B (Cell Signaling Technology Cat# 9336, RRID:AB_331405)<br>2. rabbit monoclonal anti-GSK3B (Cell Signaling Technology Cat# 9336, RRID:AB_331405)<br>3. rabbit polyclonal anti-CSNK1E (Cell Signaling Technology Cat# 12448, RRID:AB_2797919)<br>4. rabbit polyclonal anti-GFAP (LSBio, catalog number: LS-B15993)<br>5. mouse monoclonal anti-NEU-N (LSBio [LifeSpan] Cat# LS-C312122, RRID:AB_2827517)<br>6. mouse monoclonal anti-ACTB (Sigma-Aldrich Cat# A5441, RRID:AB_476744)<br>7. HRP-conjugated anti-rabbit (Bio-Rad Cat# 170-6515, RRID:AB_11125142)<br>8. HRP-conjugated anti-mouse (Bio-Rad Cat# 170-6516, RRID:AB_11125547)

Animals and other research organisms

Policy information about studies involving animals; ARRIVE guidelines recommended for reporting animal research, and Sex and Gender in Research

Laboratory animals	1. C57BL/6N (used as a background strain for all transgenic animals).<br>2. Wild-type male litter-mates (used as control).<br>3. THRSP-overexpressing (THRSP OE) male mice.<br>4. THRSP KO (homozygous) male mice.
Wild animals	The study did not involve wild animals.
Reporting on sex	To preserve the TG mouse lines and maintain a significant number of samples for our experiments, female mice of each strain followed continuous breeding, and male mice experimented.
Field-collected samples	The study did not involve samples collected in the field.
Ethics oversight	All standard animal care and procedures were performed following the Principles of Laboratory Animal Care (NIH Publication No. 85-23, revised 1985), the Animal Ethics Review Board of Sahmyook University, South Korea (SYUIACUC2020-010), and in compliance with the 3Rs framework and ARRIVE guidelines recommended by Communications Biology.

Note that full information on the approval of the study protocol must also be provided in the manuscript.
